# Supplementary material for: Pharmacogenomics of steroid-induced ocular hypertension: relationship to high-tension glaucomas and new pathophysiologic insight
Source: medRxiv. 2025 Aug 13:2025.08.11.25333245. Preprint. [Version 1] doi: 10.1101/2025.08.11.25333245 (PMC12363710; doi:10.1101/2025.08.11.25333245)
Supplement: Supplement 1 — Figure S1. Q-Q plots for the Indianapolis-1 discovery cohort Figure S2. Q-Q and Manhattan plots for the Indianapolis-2 replication cohort Figure S3. Aqueous outflow pathway (AOP) cell type expression of top prioritized target genes and prioritized target genes of replicated SNPs. Figure S4. Effects of sQTLs identified in the COL11A1 gene associated with SIOH or other high-tension ocular phenotypes. [file media-1.pdf]

Supplementary Figure S1. Q-Q plots for the Indianapolis-1 discovery cohort.

Quantile-quantile (Q-Q) plots showing the distribution of the observed P values from the logistic regression analysis for the GWAS scan against the expected distribution under the null hypothesis. The two quantitative traits (QTs) used are indicated. The x-axis of QQ-plot reports the expected −log10 (p-value) and the y-axis reports the observed −log10 (p -value). The genomic control parameters (λ), which are close to 1, indicate that population stratification is addressed properly.

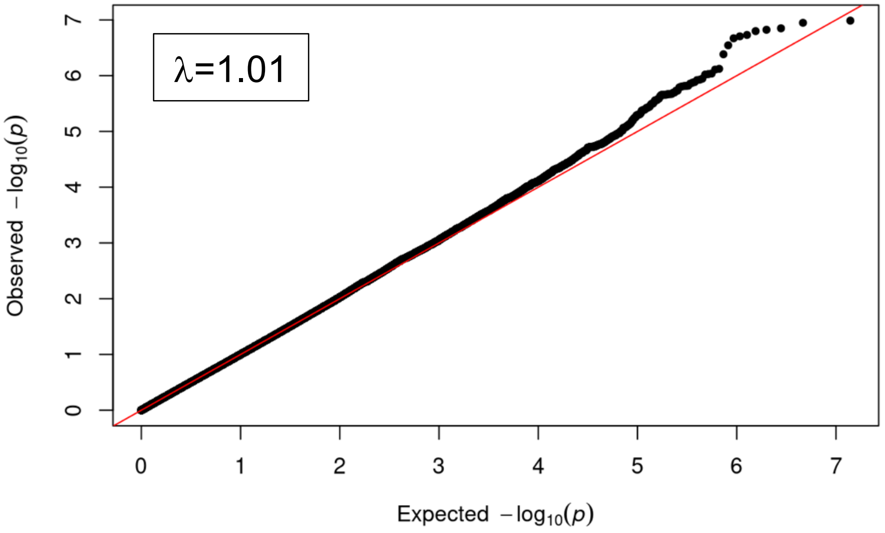

12 month QT

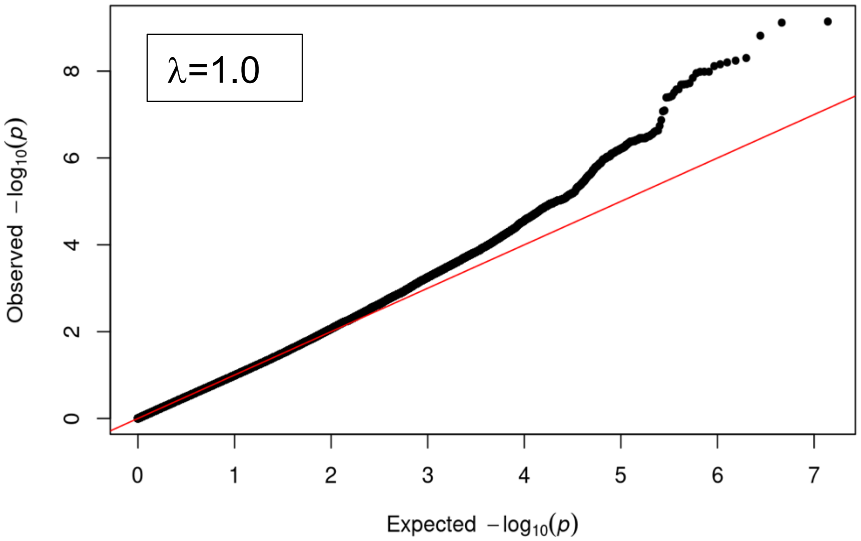

3 month QT

Supplementary Figure S2. Q-Q and Manhattan plots for the Indianapolis-2 replication cohort.

Quantile-quantile (Q-Q)-plot (left) and Manhattan plot (right) of the GWAS conducted for the Indianapolis-2 cohort with the quantitative trait (QT) as indicated. The x-axis of Q-Q-plot reports the expected −log10 (p-value) and the y-axis reports the observed −log10 (p -value). The x-axis of Manhattan plot reports chromosomes and coordinates within chromosomes. The y-axis reports the −log10 (p -value). The genomic control parameter (λ), which is close to 1, indicates that population stratification is addressed properly.

12 month QT

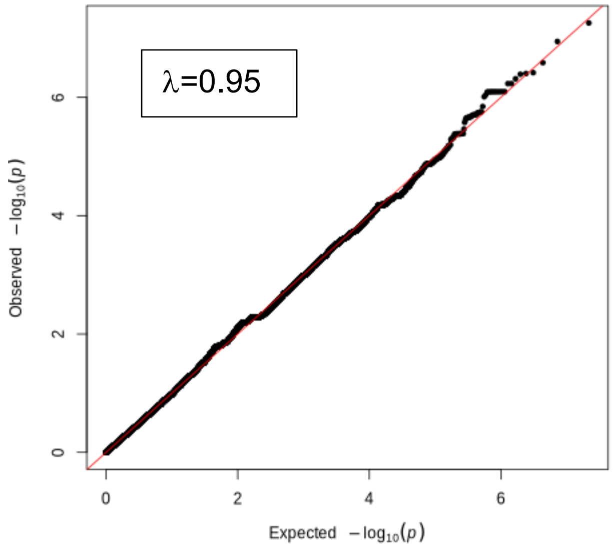

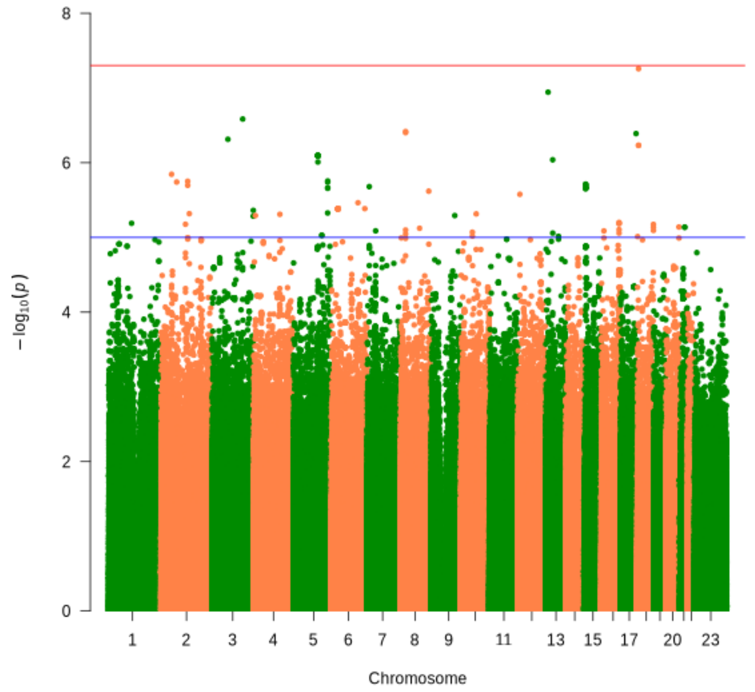

3 month QT

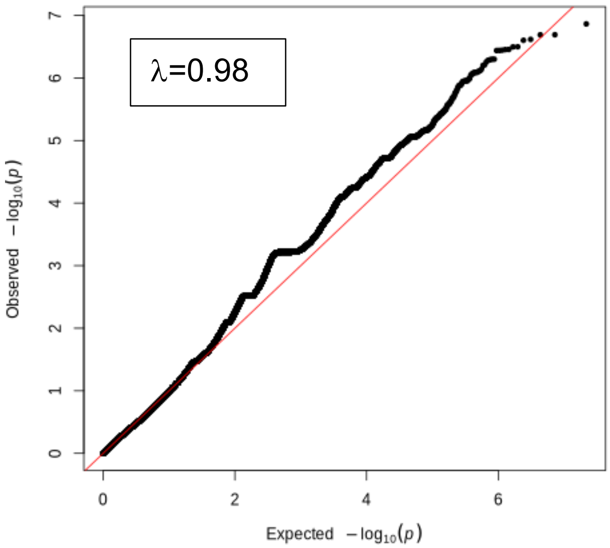

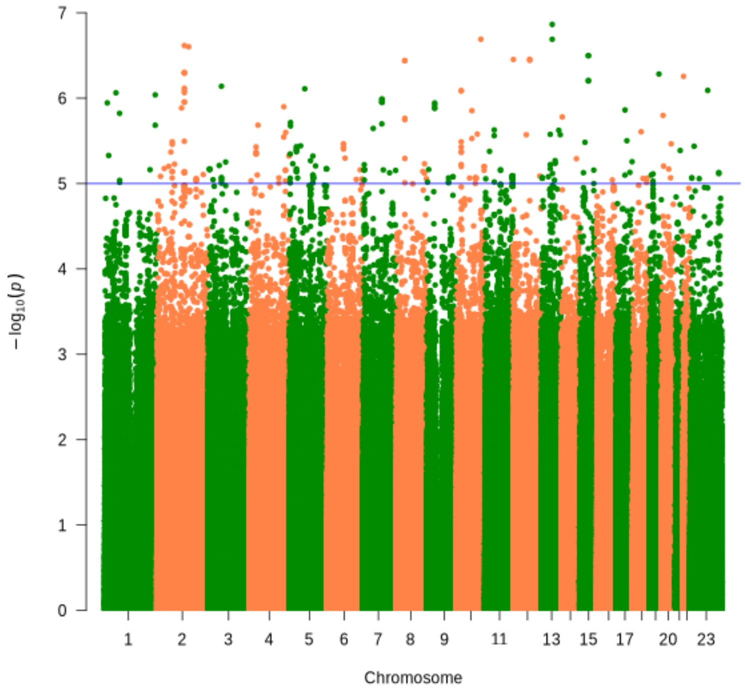

Supplementary Figure S3. Aqueous outflow pathway (AOP) cell type expression of top prioritized target genes and prioritized target genes of replicated SNPs.

Gene expression heat map as viewed in the van Zyl human scRNA-seq dataset hosted on Spectacle. The top prioritized target genes are in red. The 3 prioritized target genes of replicated SNPs are in blue.

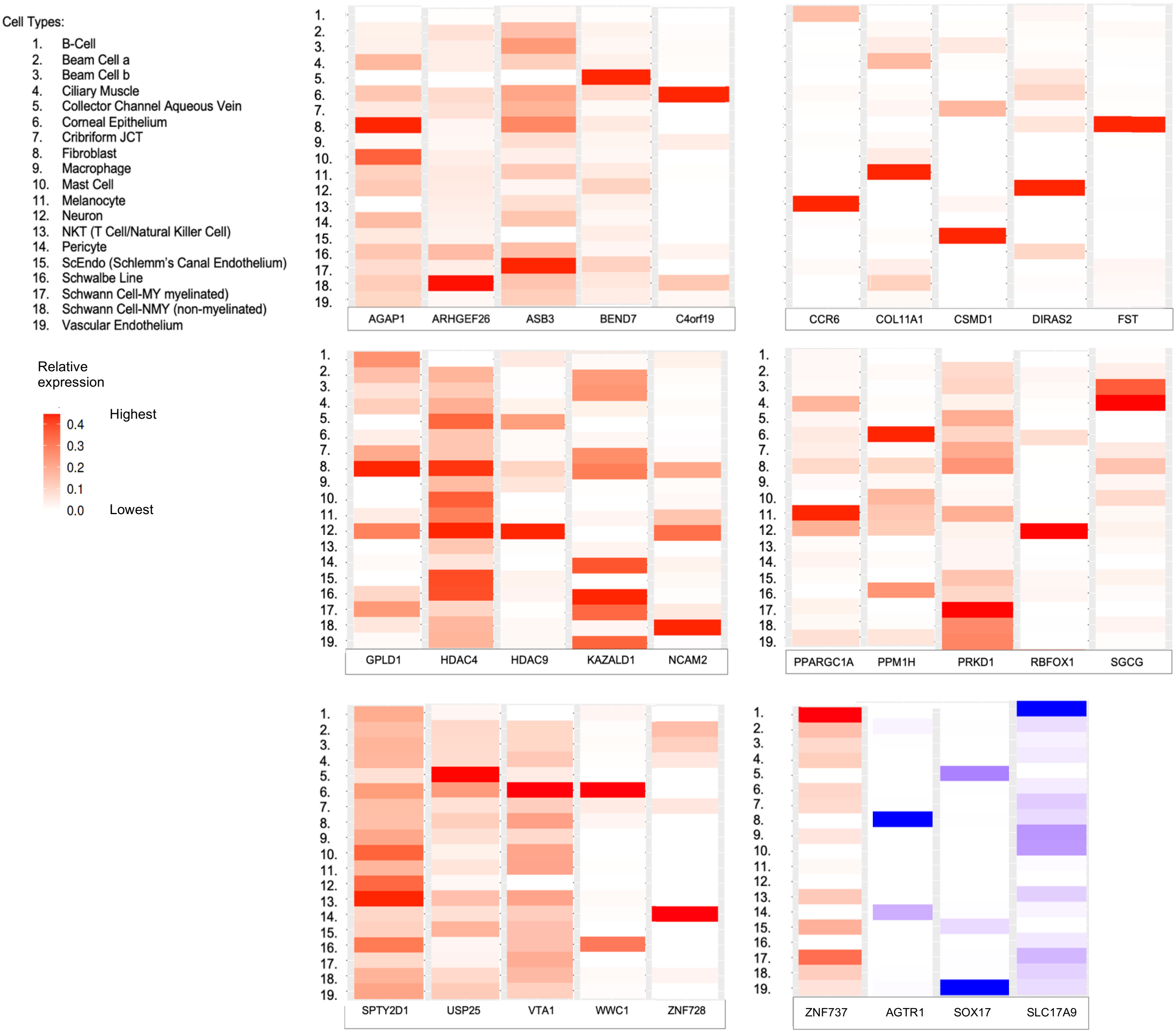

Supplementary Figure S4. Effects of sQTLs identified in the *COL11A1* gene associated with SIOH or other high-tension ocular phenotypes. Each of the indicated SNPs was searched on the GTEx portal. Shown are QTL violin plots depicting the effect of the minor allele on splicing (tissue type: testis).

COL11A1 gene: 66 introns and 67 exons

Positional Information from Genome Build GRCh38:

- Chr1:102,876,473-103,108,521 = 232,049 bp
- Chr1: 102,979,612-102,984,138 = 8,885 bp = alternative splicing, intron 31
- Chr1: 102,946,956-102,961,866 = 14,911 bp = intron 41
- Chr1: 102,883,341-102,886,807 = 3,467 bp = intron 49
- Chr1: 102,888,311-102,886,807 = 3,497 bp = intron 62

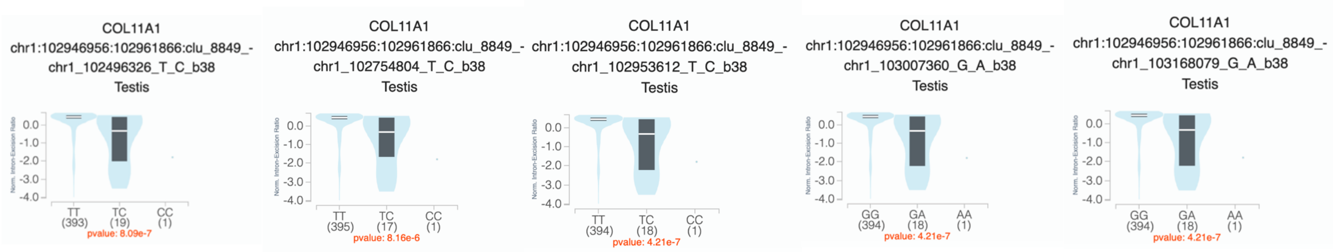

SIOH rs77180278<br>negative effect	SIOH rs112351653<br>negative effect	SIOH rs114413507<br>negative effect	SIOH rs116672066<br>negative effect	SIOH rs111928960<br>negative effect

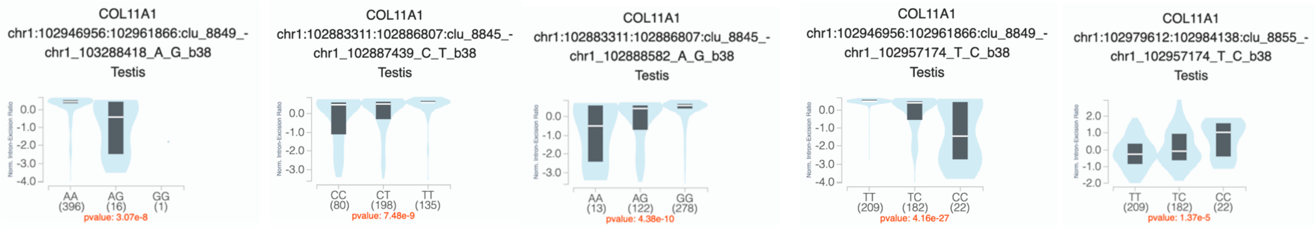

SIOH rs113221952<br>negative effect	PACG/POAG rs11164630<br>positive effect	PACG rs1676486<br>positive effect	PEXS rs3102053<br>negative effect	PEXS rs3102053<br>positive effect
