# Supplementary material for: Pharmacogenomics of steroid-induced ocular hypertension: relationship to high-tension glaucomas and new pathophysiologic insight
Source: medRxiv. 2025 Aug 13:2025.08.11.25333245. Preprint. [Version 1] doi: 10.1101/2025.08.11.25333245 (PMC12363710; doi:10.1101/2025.08.11.25333245)
Supplement: Supplement 2 — Table S1. GWAS Results SNPs Indianapolis-1 Discovery Cohort [file media-2.pdf]

Supplementary Table S1. GWAS Results SNPs Indianapolis-1 Discovery Cohort
12 month quantitative trait (QT)

Headers

QT: quantitiative trait; rsid: reference SNP cluster ID, chr: chromosome; pos_38, position of SNP on GRCh38 reference panel; Imputation_Rsq (if <0.8); REF and ALT, reference allele and alternate allele; n.obs: number of observations;
caf: common (major) allele frequency; MAC, minor allele count; Score: p-values from Score test; Score.SE; Score.Stat; Score.pval; EST; EST.SE; Func.refGene: SNP location with respect to nearest gene;
Gene.refGene; nearest gene upstream and downstream; GeneDetail.refGene: distance to nearest gene upstream and downstream; GeneDetail.refGene: distance to nearest gene upstream and downstream

Notes

Imputation Rsq listed only if less than 0.8

QT	rsid	chr	pos_38	Imputation_Rsq	REF	ALT	n.obs	caf	MAC	Score	Score.SE	Score.Stat	Score.pval	Est	Est.SE	Func.refGene	Gene.refGene	GeneDetail.refGene
12 mo	rs142934021	5	53366842	0.721159995	G	A	439	0.0079727	7	1.70971028	0.301367945	5.673165672	1.40E-08	18.82471499	3.318202936	intergenic	LOC257396;FST	dist=251716;dist=113787
12 mo	rs138164904	16	6808238		C	T	439	0.0034169	3	1.270258009	0.230575889	5.509066939	3.61E-08	23.89264096	4.336966899	intronic	RBFOX1	.
12 mo	rs138138661	3	147940027	0.736020029	C	T	439	0.0045558	4	1.27663259	0.23490951	5.434571772	5.49E-08	23.13474571	4.256958355	intergenic	LOC440982;LINC02032	dist=430117;dist=138132
12 mo	rs147669485	18	29023686		G	T	439	0.0034169	4	1.284305415	0.241008458	5.3288811	9.88E-08	22.11076388	4.149231981	intergenic	CDH2;MIR302F	dist=846556;dist=1275226
12 mo	rs148997617	3	148037789		C	T	439	0.0045558	4	1.354154489	0.254680453	5.317072728	1.05E-07	20.87742767	3.926489017	intergenic	LOC440982;LINC02032	dist=527879;dist=40370
12 mo	rs74572772	22	34096660		A	G	439	0.0182232	16	2.712607909	0.515127295	5.265898221	1.40E-07	10.22251833	1.941267739	intergenic	LARGE1;LINC02885	dist=173837;dist=660007
12 mo	rs148157126	20	32361036		C	T	439	0.0045558	5	1.399252324	0.26592767	5.261777862	1.43E-07	19.78650011	3.760421027	intronic	ASXL1	.
12 mo	rs80019988	22	34097658		G	A	439	0.0182232	16	2.739120335	0.52174256	5.249946134	1.52E-07	10.06233062	1.916654069	intergenic	LARGE1;LINC02885	dist=174835;dist=659009
12 mo	rs186792608	2	42869559		A	G	439	0.0034169	3	1.224622659	0.233550544	5.243501632	1.58E-07	22.45124992	4.281728413	intergenic	HAAO;LINC01819	dist=76976;dist=158293
12 mo	rs9974985	21	39935648	0.79768002	G	A	439	0.8861048	114	-6.320045974	1.205919394	-5.240852753	1.60E-07	-4.345939521	0.829242821	intergenic	PCP4;DSCAM	dist=6256;dist=75353
12 mo	rs113154814	2	68062365	0.753430009	T	C	439	0.0056948	5	1.38111384	0.263901723	5.233440026	1.66E-07	19.83101879	3.789289395	intronic	C1D	.
12 mo	rs1005412	21	39937023	0.782010019	A	G	439	0.8872437	112	-6.127850017	1.173197527	-5.223204001	1.76E-07	-4.452109624	0.852371384	intergenic	PCP4;DSCAM	dist=7631;dist=73978
12 mo	rs193041547	20	32184077		T	C	439	0.0045558	4	1.384000945	0.265194908	5.218806627	1.80E-07	19.67913585	3.770811462	intergenic	TM9SF4;TSPY26P	dist=16819;dist=5069
12 mo	rs77297738	22	34077554		C	T	439	0.0182232	16	2.636152158	0.507182742	5.197637737	2.02E-07	10.24805718	1.971675922	intergenic	LARGE1;LINC02885	dist=154731;dist=679113
12 mo	rs146249289	20	32094428		C	T	439	0.0045558	4	1.377562106	0.265036842	5.197624967	2.02E-07	19.6109527	3.773060354	intronic	HCK	.
12 mo	rs9636964	21	39932840	0.797720015	A	G	439	0.8849658	115	-6.21374108	1.208541419	-5.141520995	2.73E-07	-4.254319226	0.827443714	intergenic	PCP4;DSCAM	dist=3448;dist=78161
12 mo	rs76356799	3	179875980		G	A	439	0.0045558	4	1.36392794	0.265929638	5.128905337	2.91E-07	19.28670071	3.760393191	intronic	PEX5L	.
12 mo	rs7275595	21	39935998	0.794510007	G	A	439	0.8883827	113	-6.12484339	1.198927666	-5.108601265	3.25E-07	-4.260975379	0.834078676	intergenic	PCP4;DSCAM	dist=6606;dist=75003
12 mo	rs192855100	20	32520938		G	A	439	0.0045558	4	1.26803868	0.250556407	5.060891062	4.17E-07	20.19860967	3.99111726	intronic	NOL4L	.
12 mo	rs2427460	20	62959430	0.922999978	T	C	439	0.4863326	420	-9.771088627	1.948334389	-5.015098375	5.30E-07	-2.574043965	0.513258918	intronic	SLC17A9	.
12 mo	rs562831582	19	50731053	0.742709994	A	C	439	0.0034169	4	1.186136633	0.237177678	5.001046654	5.70E-07	21.08565484	4.216248377	intergenic	CLEC11A;GPR32	dist=5345;dist=39411
12 mo	rs149859280	20	32090367	0.777029991	C	T	439	0.0045558	5	1.36598448	0.273167403	5.000539835	5.72E-07	18.30577068	3.660758895	intronic	HCK	.
12 mo	rs138733283	20	32087884	0.776130021	C	T	439	0.0045558	5	1.365481855	0.273350907	4.995344152	5.87E-07	18.27447439	3.658301378	intronic	HCK	.
12 mo	rs80212581	16	6362966		C	T	439	0.0034169	3	1.113691785	0.22359493	4.980845435	6.33E-07	22.27620028	4.472373329	intronic	RBFOX1	.
12 mo	rs148248743	3	136415753	0.708679974	C	T	439	0.0022779	3	0.909706546	0.183031941	4.970206509	6.69E-07	27.15485873	5.463527255	intronic	STAG1	.
12 mo	rs188353596	17	48001236		C	T	439	0.0034169	3	1.130738257	0.227716806	4.965545927	6.85E-07	21.80579474	4.391419406	intergenic	CDK5RAP3;COPZ2	dist=19450;dist=24931
12 mo	rs113791989	13	61344755	0.787899971	G	A	439	0.0022779	3	0.975147098	0.196694436	4.957675046	7.13E-07	25.20495822	5.084027893	intergenic	MIR3169;PCDH20	dist=144874;dist=64931
12 mo	rs56324718	15	39987872		G	A	439	0.0102506	9	1.956402255	0.395430594	4.947523752	7.52E-07	12.5117374	2.5288888	intronic	EIF2AK4	.
12 mo	rs191298981	2	67882914	0.724789977	C	T	439	0.0045558	3	1.017681042	0.205887042	4.942909629	7.70E-07	24.00787141	4.857032236	intergenic	LINC01812;C1D	dist=57352;dist=158216
12 mo	rs146919974	2	114495286	0.777989984	T	C	439	0.0045558	5	1.23575568	0.250278916	4.93751411	7.91E-07	19.72804656	3.995542316	intronic	DPP10	.
12 mo	rs9305683	21	39933795	0.793929994	G	A	439	0.8895216	112	-5.884975803	1.19568078	-4.921862005	8.57E-07	-4.116367918	0.836343627	intergenic	PCP4;DSCAM	dist=4403;dist=77206
12 mo	rs145791959	20	32134626	0.783770025	G	A	439	0.0056948	5	1.351424877	0.274699531	4.919647552	8.67E-07	17.90919529	3.640341121	intronic	TM9SF4	.
12 mo	rs142956968	10	23201236		C	T	439	0.0159453	14	2.319717767	0.47190049	4.915692647	8.85E-07	10.41679921	2.119090829	downstream	C10orf67	dist=680
12 mo	rs532269430	13	73480315		T	C	439	0.0045558	5	1.291806032	0.263414349	4.904083761	9.39E-07	18.61737516	3.796300403	intergenic	KLF5;LINC00392	dist=402772;dist=83929
12 mo	rs113063005	4	23507444	0.763409972	T	C	439	0.0068337	7	1.445039344	0.294886773	4.900319289	9.57E-07	16.61762999	3.391132089	intergenic	GBA3;PPARGC1A	dist=687872;dist=284577
12 mo	rs147944608	10	108663951		C	T	439	0.0125285	11	2.135371399	0.436244671	4.894893947	9.84E-07	11.22052434	2.292291613	intergenic	LINC01435;XPNPEP1	dist=594658;dist=1200815
12 mo	rs2606194	17	79214741		A	G	439	0.9487472	44	-4.201206996	0.858842579	-4.891707862	1.00E-06	-5.695697885	1.164357735	intronic	RBFOX3	.
12 mo	rs113537164	2	68007798		A	C	439	0.0045558	4	1.171984248	0.239752135	4.888316203	1.02E-06	20.38904135	4.17097432	intergenic	LINC01812;C1D	dist=182236;dist=33332
12 mo	rs138055631	20	32192841		G	A	439	0.0056948	5	1.347966833	0.276239343	4.879706185	1.06E-06	17.66477622	3.620049149	UTR3	PLAGL2	NM_002657:c.*36110>0
12 mo	rs566724618	13	22889700		C	T	439	0.0034169	3	1.064880575	0.218311246	4.877809059	1.07E-06	22.34337048	4.580616053	ncRNA_intronic	LINC00621	.
12 mo	rs145764464	10	23312918		G	A	439	0.0170843	15	2.47340447	0.507675165	4.872021796	1.10E-06	9.596730604	1.969763479	intronic	C10orf67	.
12 mo	rs9981433	21	39937640	0.77809	G	T	439	0.881549	116	-5.743602038	1.180422615	-4.865716705	1.14E-06	-4.122012439	0.84715422	intergenic	PCP4;DSCAM	dist=8248;dist=73361
12 mo	rs139816293	20	32333540	0.77214998	C	T	439	0.0056948	6	1.378989447	0.283657989	4.861451108	1.17E-06	17.13842475	3.525372234	UTR3	KIF3B	NM_004798:c.*22210>0
12 mo	rs117699122	12	451096	0.758159995	T	C	439	0.0034169	4	1.10182785	0.226781885	4.858535554	1.18E-06	21.42382563	4.40952328	intergenic	CCDC77;B4GALNT3	dist=8456;dist=8843
12 mo	rs529110230	7	117535853		T	C	439	0.0034169	3	1.126856399	0.232431066	4.848131618	1.25E-06	20.85836332	4.302350877	intronic	CFTR	.
12 mo	rs12114488	8	61760164		G	A	439	0.3052392	266	8.910223585	1.843056308	4.834482563	1.33E-06	2.623079144	0.54257702	intergenic	MIR4470;LINC02155	dist=45305;dist=129675
12 mo	rs182868205	3	177994660		C	T	439	0.0034169	3	1.020592047	0.21132014	4.829601415	1.37E-06	22.85443031	4.732156621	intergenic	LINC02015;LINC01014	dist=95436;dist=424541
12 mo	rs144138711	9	97160137		T	C	439	0.0455581	40	3.827539823	0.793072242	4.826218371	1.39E-06	6.085471305	1.260919179	ncRNA_intronic	ANKRD18CP	.
12 mo	rs372194899	13	73783373		A	G	439	0.0034169	3	1.092725742	0.226492304	4.824560146	1.40E-06	21.30121009	4.41516106	intronic	KLF12	.
12 mo	rs368187808	13	73783371		A	C	439	0.0034169	3	1.092690402	0.226499726	4.82424601	1.41E-06	21.2991251	4.415016369	intronic	KLF12	.
12 mo	rs145804766	1	202153371		C	T	439	0.0102506	9	1.845977123	0.382967209	4.820196299	1.43E-06	12.58644653	2.611189618	intronic	PTPN7	.
12 mo	rs182211730	7	116363561		G	A	439	0.0045558	4	1.236134718	0.256594603	4.817461878	1.45E-06	18.7746033	3.897198104	intergenic	LOC102724434;CAV2	dist=76834;dist=136177
12 mo	rs558784715	7	117403240		A	G	439	0.0034169	3	1.113953973	0.231647992	4.80882206	1.52E-06	20.75917871	4.316894751	intronic	ASZ1	.
12 mo	rs189709453	3	177856520		G	A	439	0.0034169	3	0.994705176	0.207058897	4.803972151	1.56E-06	23.20099361	4.829543736	ncRNA_intronic	LINC02015	.
12 mo	rs142721557	7	117578274		A	C	439	0.0034169	3	1.1096866	0.231349797	4.796574767	1.61E-06	20.73299749	4.322458941	intronic	CFTR	.
12 mo	rs576047962	7	117468967		A	G	439	0.0034169	3	1.109863163	0.231406391	4.796164697	1.62E-06	20.72615487	4.321401824	intergenic	ASZ1;CFTR	dist=41474;dist=11058
12 mo	rs142215699	7	117559820		T	C	439	0.0034169	3	1.109487618	0.231350082	4.795708764	1.62E-06	20.72922868	4.322453614	ncRNA_intronic	CFTR-AS1	.

12 mo	rs143281973	7	116367942		C	T	439	0.0045558	4	1.258303619	0.262399562	4.795372412	1.62E-06	18.27507784	3.810981978	intergenic	LOC102724434;CAV2	dist=81215;dist=131796
12 mo	rs17127656	1	65477788		C	T	439	0.0558087	49	4.531431604	0.945352558	4.793377416	1.64E-06	5.070465376	1.057806414	intronic	LEPR	.
12 mo	rs201355675	7	117585727		G	A	439	0.0034169	3	1.108886281	0.231349354	4.7931246	1.64E-06	20.71812396	4.322467219	intronic	CFTR	.
12 mo	rs186767531	3	177921989		T	C	439	0.0034169	3	0.994608422	0.207613329	4.790677095	1.66E-06	23.07499768	4.81664642	intergenic	LINC02015;LINC01014	dist=22765;dist=497212
12 mo	rs7518849	1	65483108		T	C	439	0.0558087	49	4.51937809	0.943712555	4.788935003	1.68E-06	5.074569555	1.059644692	intronic	LEPR	.
12 mo	rs181132315	4	128726214	0.779959977	C	T	439	0.0034169	3	1.035727795	0.216325589	4.787819148	1.69E-06	22.13246791	4.62266164	intergenic	LINC02615;JADE1	dist=206818;dist=83486
12 mo	rs75082290	2	67604021		T	G	439	0.0785877	69	4.880252144	1.019651405	4.786196655	1.70E-06	4.693953865	0.98072733	intergenic	ETAA1;LINC01812	dist=191932;dist=192033
12 mo	rs145421321	20	32274323		C	T	439	0.0056948	6	1.344334892	0.281033355	4.783542121	1.72E-06	17.02126111	3.558296484	intergenic	POFUT1;KIF3B	dist=35665;dist=3328
12 mo	rs143432612	20	32304972	0.798579991	C	T	439	0.0056948	6	1.349254459	0.282232222	4.780653489	1.75E-06	16.93872319	3.543181539	intronic	KIF3B	.
12 mo	rs140276610	16	6383734	0.729809999	C	T	439	0.0034169	4	1.029963075	0.215488262	4.779671375	1.76E-06	22.18065773	4.640624008	intronic	RBFOX1	.
12 mo	rs559152067	18	68362375	0.792789996	G	A	439	0.0022779	3	0.920959359	0.192922126	4.773736308	1.81E-06	24.74436915	5.183438622	intergenic	LOC643542;TMX3	dist=462756;dist=311313
12 mo	rs147221953	13	22911583		G	A	439	0.0034169	3	1.00672997	0.211147151	4.767906961	1.86E-06	22.58096755	4.736033596	ncRNA_intronic	LINC00621	.
12 mo	rs556274646	11	439012		C	T	439	0.0102506	9	1.757323369	0.369211146	4.759670415	1.94E-06	12.89145917	2.708477277	intronic	ANO9	.
12 mo	rs190251199	14	105124240	0.736329973	T	C	439	0.0034169	3	0.998973364	0.210064697	4.755550928	1.98E-06	22.63850613	4.760438165	intergenic	LINC02298;JAG2	dist=24744;dist=16758
12 mo	rs188993522	7	117634677		T	C	439	0.0034169	3	1.061817851	0.22328044	4.755534576	1.98E-06	21.29848267	4.478672657	intronic	CFTR	.
12 mo	rs568321148	2	29647482		T	G	439	0.0034169	3	1.087769414	0.22901495	4.749774698	2.04E-06	20.74002026	4.366527169	intronic	ALK	.
12 mo	rs148815783	11	1013992		C	T	439	0.0079727	8	1.656962032	0.349074609	4.746727463	2.07E-06	13.59803132	2.864717097	exonic	MUC6	.
12 mo	rs76220567	2	12919819	0.745050013	T	C	439	0.0034169	4	1.089141003	0.22947558	4.746217455	2.07E-06	20.68288684	4.357762163	intergenic	TRIB2;LOC100506474	dist=177087;dist=46963
12 mo	rs35145334	1	23138629		A	G	439	0.0170843	14	2.111856339	0.445194762	4.743668432	2.10E-06	10.65526559	2.246207918	intronic	LUZP1	.
12 mo	rs11579567	1	65491458		C	A	439	0.0558087	49	4.468235403	0.942530351	4.740680657	2.13E-06	5.029737931	1.060973792	intronic	LEPR	.
12 mo	rs114067899	1	84725361		G	A	439	0.0079727	7	1.628650291	0.343835958	4.736707304	2.17E-06	13.77606734	2.908363649	intergenic	SSX2IP;LPAR3	dist=34893;dist=86241
12 mo	rs56821264	6	148382765		C	T	439	0.0068337	6	1.520993492	0.321110203	4.736671324	2.17E-06	14.75092126	3.114195656	intronic	SASH1	.
12 mo	rs536781978	2	29510815		A	G	439	0.0034169	3	1.090050785	0.230277801	4.733633818	2.21E-06	20.55618824	4.342580992	intronic	ALK	.
12 mo	rs147267707	4	43024541		G	A	439	0.0045558	4	1.241836175	0.262592348	4.729140754	2.25E-06	18.00943854	3.808184082	intronic	GRXCR1	.
12 mo	rs77618729	4	43048951		T	C	439	0.0045558	4	1.241458345	0.26252633	4.728890791	2.26E-06	18.01301525	3.809141731	intergenic	GRXCR1;LINC02383	dist=18293;dist=408583
12 mo	rs547186621	20	6126094	0.76117003	A	G	439	0.0034169	4	1.03230976	0.218862053	4.716714238	2.40E-06	21.55108289	4.569088099	intergenic	FERMT1;CASC20	dist=3064;dist=300638
12 mo	rs200198574	20	32358851		G	A	439	0.0056948	6	1.343845487	0.285141824	4.712902058	2.44E-06	16.52827354	3.50702674	intronic	ASXL1	.
12 mo	rs532464521	4	43569022		T	C	439	0.0045558	4	1.233962678	0.261903215	4.711521696	2.46E-06	17.98955275	3.818204376	intergenic	LINC02383;LINC02475	dist=76479;dist=447839
12 mo	rs79252854	3	157805368		C	T	439	0.0045558	5	1.264269707	0.26842653	4.709928283	2.48E-06	17.54643357	3.72541417	intergenic	SLC66A1L;SHOX2	dist=204274;dist=290537
12 mo	rs79935606	2	176473907		T	C	439	0.0056948	5	1.354071441	0.2878717	4.703732401	2.55E-06	16.33968329	3.473769742	intergenic	MTX2;MIR1246	dist=135882;dist=127073
12 mo	rs556435089	4	43549016		G	A	439	0.0045558	4	1.230811842	0.261936344	4.698896777	2.62E-06	17.93907904	3.817721456	intergenic	LINC02383;LINC02475	dist=56473;dist=467845
12 mo	rs62192733	20	1947511	0.746280015	A	G	439	0.0068337	7	1.395720916	0.297737289	4.687759871	2.76E-06	15.74461797	3.358665632	ncRNA_exonic	PDYN-AS1	.
12 mo	rs7534177	1	65500037		A	G	439	0.0569476	49	4.416633642	0.943519417	4.681020403	2.85E-06	4.961233778	1.059861601	intronic	LEPR	.
12 mo	rs139390630	1	84400513	0.718169987	G	A	439	0.0113895	13	1.872384943	0.40008221	4.680000507	2.87E-06	11.69759713	2.499486296	intronic	DNASE2B	.
12 mo	rs149706477	3	66162553		A	G	439	0.0034169	3	1.043481199	0.223002578	4.679233802	2.88E-06	20.98286869	4.484253102	intronic	SLC25A26	.
12 mo	rs189094663	4	11621826	0.791639984	G	A	439	0.0022779	3	0.91574426	0.195762636	4.677829629	2.90E-06	23.895416	5.108227082	intergenic	HS3ST1;LINC02360	dist=192932;dist=119125
12 mo	rs181182636	7	116014504		A	G	439	0.0034169	3	0.996739233	0.213108408	4.677146454	2.91E-06	21.94726388	4.692447434	intronic	TFEC	.
12 mo	rs76098744	1	111806750		C	T	439	0.0159453	14	2.284999945	0.488674498	4.675914038	2.93E-06	9.568565696	2.046351926	intronic	KCND3	.
12 mo	rs4896997	6	148362788		C	T	439	0.0068337	6	1.509673329	0.323427425	4.667734442	3.05E-06	14.43209229	3.091883755	intronic	SASH1	.
12 mo	rs17078283	6	148375784		C	T	439	0.0068337	6	1.509561069	0.323435684	4.667268152	3.05E-06	14.43028205	3.091804795	intronic	SASH1	.
12 mo	rs73202425	7	109515659		T	C	439	0.0125285	12	2.024115362	0.433684884	4.667249048	3.05E-06	10.76184396	2.305821662	intergenic	C7orf66;EIF3IP1	dist=631072;dist=443568
12 mo	rs4131286	6	148367827		G	T	439	0.0068337	6	1.509448809	0.323443998	4.666801106	3.06E-06	14.42846716	3.091725323	intronic	SASH1	.
12 mo	rs74683551	1	111811796		G	A	439	0.0159453	14	2.279782265	0.488661396	4.665361915	3.08E-06	9.547228317	2.046406793	intronic	KCND3	.
12 mo	rs139055031	3	158869060	0.75673002	T	C	439	0.0056948	6	1.332817863	0.286079347	4.658909756	3.18E-06	16.28537606	3.4955337	intergenic	MFSD1;IQCJ	dist=39341;dist=200192
12 mo	rs138873576	7	116385305		G	T	439	0.0045558	4	1.230673437	0.264318756	4.656020093	3.22E-06	17.61517106	3.78331079	intergenic	LOC102724434;CAV2	dist=98578;dist=114433
12 mo	rs148483098	3	157664262		C	A	439	0.0056948	5	1.375665606	0.29567339	4.652652729	3.28E-06	15.73578443	3.382110237	intergenic	SLC66A1L;SHOX2	dist=63168;dist=431643
12 mo	rs187281112	15	32821186		C	T	439	0.0056948	5	1.468174325	0.315662894	4.651083021	3.30E-06	14.73433563	3.167936491	intronic	FMN1	.
12 mo	rs2902021	2	67598553		C	T	439	0.0797267	71	4.743601835	1.019942721	4.650851208	3.31E-06	4.559914114	0.980447215	intergenic	ETAA1;LINC01812	dist=186464;dist=197501
12 mo	rs189912648	5	135429749		C	T	439	0.0034169	4	1.088415104	0.234100744	4.649344921	3.33E-06	19.8604449	4.271665199	intergenic	MACROH2A1;DCANP1	dist=29862;dist=14465
12 mo	rs557276277	4	19012075	0.731580019	T	C	439	0.0056948	5	1.135923633	0.244416532	4.647491003	3.36E-06	19.01463443	4.091376275	intergenic	LCORL;SLIT2	dist=990200;dist=1239830
12 mo	rs147869916	1	227119951		A	G	439	0.0056948	5	1.227452207	0.264112954	4.647451732	3.36E-06	17.59645507	3.78625881	intronic	CDC42BPA	.
12 mo	rs367732718	19	35427102	0.764100015	G	A	439	0.0022779	3	0.903832269	0.194554137	4.645659467	3.39E-06	23.87849231	5.139957519	intergenic	LINC01531;FFAR2	dist=10262;dist=21155
12 mo	rs62515436	8	56228644		G	T	439	0.0170843	15	2.32339754	0.500488447	4.642260083	3.45E-06	9.275459023	1.998048118	intergenic	CHCHD7;SDR16C5	dist=9835;dist=71004
12 mo	rs189234695	3	147940371	0.726230025	T	C	439	0.0034169	3	0.898361941	0.193595051	4.640417897	3.48E-06	23.96971341	5.165421292	intergenic	LOC440982;LINC02032	dist=430461;dist=137788
12 mo	rs186893139	7	116456916		C	T	439	0.0045558	4	1.155381378	0.249215977	4.636064639	3.55E-06	18.60259803	4.012583835	intergenic	LOC102724434;CAV2	dist=170189;dist=42822
12 mo	rs73384619	6	20457948		C	T	439	0.022779	21	2.554663139	0.55175035	4.630106969	3.65E-06	8.391670204	1.812413894	intronic	E2F3	.
12 mo	rs114280794	3	133123359		G	A	439	0.0159453	14	2.436845188	0.527337114	4.621038659	3.82E-06	8.762968775	1.896320161	intronic	TMEM108	.
12 mo	rs112148840	17	68237633	0.760460019	C	T	439	0.0022779	3	0.88865431	0.192328123	4.620511518	3.83E-06	24.02410753	5.199447601	intronic	AMZ2	.
12 mo	rs147455971	4	140010558	0.772499979	T	C	439	0.0079727	5	1.238787979	0.268245339	4.618115576	3.87E-06	17.21601424	3.727930571	intronic	MAML3	.
12 mo	rs144788248	3	19545253		T	C	439	0.0113895	10	1.790726259	0.387778578	4.617909184	3.88E-06	11.90862374	2.578791237	intergenic	KCNH8;EFHB	dist=9611;dist=334221
12 mo	rs111512950	5	154300867		C	T	439	0.0091116	8	1.5632807	0.33944505	4.605401379	4.12E-06	13.56744305	2.945984928	intronic	GALNT10	.
12 mo	rs149616342	12	98785035		C	T	439	0.0113895	10	1.875050964	0.407388894	4.602606968	4.17E-06	11.2978214	2.454656997	intronic	ANKS1B	.
12 mo	rs17543620	4	169013574		T	C	439	0.0501139	44	3.899464938	0.847586222	4.600670513	4.21E-06	5.427967555	1.179820972	intergenic	CBR4;SH3RF1	dist=3319;dist=80685
12 mo	rs190354334	7	116435792		G	A	439	0.0045558	5	1.218759267	0.265120744	4.596997007	4.29E-06	17.33925809	3.7718663	intergenic	LOC102724434;CAV2	dist=149065;dist=63946
12 mo	rs17615362	4	169012936		G	A	439	0.0501139	44	3.897763553	0.84804389	4.596181398	4.30E-06	5.419744721	1.179184251	intergenic	CBR4;SH3RF1	dist=2681;dist=81323
12 mo	rs117591241	3	190866890	0.774309993	A	G	439	0.0034169	4	1.059847527	0.230770266	4.592652012	4.38E-06	19.90140277	4.333313893	intergenic	GMNC;SNAR-I	dist=4196;dist=11040
12 mo	rs61918041	12	6572702		C	T	439	0.023918	21	2.644372798	0.575917252	4.591584621	4.40E-06	7.972646426	1.736360556	intronic	CHD4	.
12 mo	rs113244573	12	6575219		G	A	439	0.023918	21	2.639855196	0.575486058	4.587174889	4.49E-06	7.970957457	1.737661556	intronic	CHD4	.
12 mo	rs62515405	8	56143419		G	A	439	0.023918	21	2.59481628	0.56680103	4.578002058	4.69E-06	8.076912036	1.76428755	intergenic	MOS;PLAG1	dist=29437;dist=17490
12 mo	rs116897913	17	16935848		C	T	439	0.0034169	3	1.038882394	0.22712082	4.574139844	4.78E-06	20.13967647	4.402942883	intergenic	TBC1D27P;TNFRSF13B	dist=2672;dist=3233

12 mo	rs143686474	7	16252374		A	C	439	0.0125285	11	1.923890516	0.421029259	4.569493631	4.89E-06	10.85314984	2.375131845	ncRNA_intronic	CRPPA-AS1	.
12 mo	rs143669489	3	159675096	0.739700019	G	A	439	0.0034169	3	0.861115457	0.188536714	4.567362191	4.94E-06	24.22531986	5.304006744	intronic	IQCJ-SCHIP1;SCHIP1	.

Supplementary Table S1. GWAS Results SNPs Indianapolis-1 Discovery Cohort
3 month quantitative trait (QT)

Headers

QT: quantitiative trait; rsid: reference SNP cluster ID, chr: chromosome; pos_38, position of SNP on GRCh38 reference panel; Imputation_Rsq (if <0.8); REF and ALT, reference allele and alternate allele; n.obs: number of observations;
caf: common (major) allele frequency; MAC, minor allele count; Score: p-values from Score test; Score.SE; Score.Stat; Score.pval; EST; EST.SE; Func.refGene: SNP location with respect to nearest gene;
Gene.refGene; nearest gene upstream and downstream; GeneDetail.refGene: distance to nearest gene upstream and downstream; GeneDetail.refGene: distance to nearest gene upstream and downstream

Notes

Imputation Rsq listed only if less than 0.8

QT	rsid	chr	pos_38	Imputation_Rsq	REF	ALT	n.obs	caf	MAC	Score	Score.SE	Score.Stat	Score.pval	Est	Est.SE	Func.refGene	Gene.refGene	GeneDetail.refGene
3 month	rs113063005	4	23507444	0.763409972	T	C	421	0.005938	6	2.130262689	0.316937024	6.721406875	1.80E-11	21.2073894	3.155200957	intergenic	GBA3;PPARGC1A	dist=687872;dist=284577
3 month	rs142106992	6	141948748		C	A	421	0.003563	4	1.689946394	0.272044449	6.212023076	5.23E-10	22.83458862	3.675869896	intergenic	MIR4465;NMBR	dist=1264865;dist=125736
3 month	rs181217257	2	239067423		C	T	421	0.004751	4	1.788144205	0.287937999	6.210170973	5.29E-10	21.56773677	3.472969885	intronic	HDAC4	.
3 month	rs188076929	2	239072023		T	C	421	0.004751	4	1.865485168	0.305579814	6.104739516	1.03E-09	19.97756143	3.272467463	intronic	HDAC4	.
3 month	rs117998251	10	13455976		C	T	421	0.003563	3	1.610926128	0.264914355	6.080931816	1.19E-09	22.95433112	3.774804885	intronic	BEND7	.
3 month	rs184458518	10	13429195		T	G	421	0.003563	3	1.603233248	0.265164814	6.046176429	1.48E-09	22.80157891	3.771239424	intergenic	SEPHS1;BEND7	dist=80897;dist=9286
3 month	rs184425183	10	13415520		A	G	421	0.003563	3	1.590271029	0.264865828	6.004062666	1.92E-09	22.66831744	3.775496477	intergenic	SEPHS1;BEND7	dist=67222;dist=22961
3 month	rs148153037	6	167087898		G	A	421	0.008314	8	2.445317826	0.413004518	5.920801636	3.20E-09	14.33592462	2.421281019	intergenic	CEP43;CCR6	dist=35180;dist=23909
3 month	rs111285015	19	22940396	0.738849998	G	A	421	0.003563	3	1.280187265	0.216410877	5.915540303	3.31E-09	27.33476425	4.62083983	intergenic	ZNF723;ZNF728	dist=81729;dist=34487
3 month	rs111928960	1	103168079		G	A	421	0.028504	22	4.045287237	0.68574164	5.899141891	3.65E-09	8.602572083	1.45827516	intergenic	COL11A1;LOC101928436	dist=59557;dist=325967
3 month	rs187520610	2	53132903		G	A	421	0.004751	4	1.843352227	0.313121747	5.887014379	3.93E-09	18.80103964	3.193645952	intergenic	MIR4431;ASB3	dist=430288;dist=537076
3 month	rs545428520	5	168394261		T	C	421	0.003563	4	1.606350972	0.272919814	5.885798274	3.96E-09	21.56603503	3.664079879	intronic	WWC1	.
3 month	rs116672066	1	103007360		G	A	421	0.027316	23	4.276024986	0.728927557	5.866186486	4.46E-09	8.047694764	1.37187844	intronic	COL11A1	.
3 month	rs192134381	21	22078395		T	C	421	0.003563	3	1.565034889	0.268006444	5.83954202	5.23E-09	21.78881202	3.731253572	ncRNA_intronic	LINC01687	.
3 month	rs150586237	6	24491120		C	T	421	0.003563	3	1.542166452	0.264483938	5.830851057	5.51E-09	22.04614425	3.780947933	intergenic	GPLD1;ALDH5A1	dist=1542;dist=3849
3 month	rs528404963	5	168425020		T	C	421	0.003563	4	1.58410842	0.273744969	5.786803767	7.17E-09	21.13939767	3.653035168	intronic	WWC1	.
3 month	rs76526501	7	18391487		G	A	421	0.007126	6	2.233180137	0.387925975	5.75671721	8.58E-09	14.83973124	2.577811398	intronic	HDAC9	.
3 month	rs74455595	7	18392161		A	G	421	0.007126	6	2.233180137	0.387925975	5.75671721	8.58E-09	14.83973124	2.577811398	intronic	HDAC9	.
3 month	rs75090694	7	18407813		A	G	421	0.008314	7	2.37298061	0.41420374	5.729017827	1.01E-08	13.83140053	2.414270813	intronic	HDAC9	.
3 month	rs575473987	8	5719972		C	T	421	0.003563	3	1.471553256	0.257316966	5.718834929	1.07E-08	22.22486534	3.886257536	intergenic	CSMD1;LOC100287015	dist=725058;dist=683583
3 month	rs545690161	9	90268417	0.734099984	G	A	421	0.005938	6	1.900812423	0.332979511	5.708496652	1.14E-08	17.14368742	3.003187786	intergenic	MIR4290HG;LINC01508	dist=226918;dist=32479
3 month	rs77141817	4	37052137		T	C	421	0.003563	3	1.553761456	0.274563203	5.659030198	1.52E-08	20.61102923	3.642148656	intergenic	LINC02616;MIR4801	dist=31431;dist=189773
3 month	rs190822761	4	37097734		G	T	421	0.003563	3	1.550650175	0.274463961	5.649740562	1.61E-08	20.58463536	3.643465595	intergenic	LINC02616;MIR4801	dist=77028;dist=144176
3 month	rs10279777	7	18401966		G	A	421	0.009501	8	2.476234721	0.438670193	5.644866606	1.65E-08	12.86813351	2.279616935	intronic	HDAC9	.
3 month	rs10486295	7	18407184		G	A	421	0.009501	8	2.488096085	0.441539202	5.635051363	1.75E-08	12.76229003	2.264804562	intronic	HDAC9	.
3 month	rs147559909	2	236142879		T	C	421	0.005938	5	1.858263542	0.329805301	5.634425932	1.76E-08	17.08409752	3.032091952	intergenic	AGAP1;GBX2	dist=11086;dist=22356
3 month	rs17169602	7	18407118		G	A	421	0.009501	8	2.49198241	0.442489656	5.631730316	1.78E-08	12.72737168	2.259939835	intronic	HDAC9	.
3 month	rs139598422	13	23312875	0.783299983	A	G	421	0.003563	4	1.544940466	0.274599144	5.626166351	1.84E-08	20.48865215	3.641671944	intronic	SGCG	.
3 month	rs74704551	14	29692681		C	T	421	0.003563	3	1.302308212	0.231983741	5.613790894	1.98E-08	24.19907048	4.31064693	intronic	PRKD1	.
3 month	rs77300464	7	18369138		A	G	421	0.007126	6	2.141507953	0.382123995	5.604222657	2.09E-08	14.66597943	2.616951597	intronic	HDAC9	.
3 month	rs75606013	7	18374990		G	A	421	0.007126	6	2.140929626	0.382127995	5.602650559	2.11E-08	14.66171186	2.616924205	intronic	HDAC9	.
3 month	rs541653703	11	18680239		G	A	421	0.004751	4	1.710453669	0.305582922	5.597347058	2.18E-08	18.31694986	3.272434185	intergenic	SPTY2D1;TMEM86A	dist=45897;dist=18540
3 month	rs117913371	10	101158729		G	A	421	0.024941	21	3.968654489	0.710300363	5.587290525	2.31E-08	7.866095548	1.407855116	intergenic	TLX1NB;LINC01514	dist=17463;dist=17593
3 month	rs191053292	12	63051500	0.719120026	T	C	421	0.003563	3	1.354341299	0.243048963	5.572298207	2.51E-08	22.9266488	4.114397317	intergenic	PPM1H;AVPR1A	dist=116350;dist=91259
3 month	rs112351653	1	102754804		T	C	421	0.028504	24	4.178844629	0.750261996	5.569847131	2.55E-08	7.423869471	1.332867724	intergenic	OLFM3;COL11A1	dist=757570;dist=121663
3 month	rs114413507	1	102953612		T	C	421	0.028504	24	4.177369718	0.750215949	5.568223023	2.57E-08	7.42216029	1.332949535	intronic	COL11A1	.
3 month	rs138414342	11	18657851		G	A	421	0.004751	5	1.685610798	0.304678389	5.532426519	3.16E-08	18.15825054	3.282149429	intergenic	SPTY2D1;TMEM86A	dist=23509;dist=40928
3 month	rs16823323	3	153939413		G	A	421	0.016627	14	3.214602568	0.582174532	5.521716236	3.36E-08	9.484640653	1.717697949	intergenic	LINC02006;ARHGEF26-AS	dist=176887;dist=84988
3 month	rs183737367	9	90567765		T	C	421	0.003563	3	1.295690086	0.235587115	5.499835526	3.80E-08	23.34523057	4.244714311	ncRNA_intronic	LINC01501	.
3 month	rs560206697	19	20546292	0.700380027	C	T	421	0.002375	3	1.205675879	0.219610964	5.490053226	4.02E-08	24.99899431	4.553506728	intronic	ZNF737	.
3 month	rs79486609	21	15872687		G	A	421	0.003563	3	1.447442576	0.264672828	5.468799292	4.53E-08	20.66248857	3.77824957	intronic	USP25	.
3 month	rs151115079	11	18634194	0.792460024	T	C	421	0.004751	5	1.669234911	0.305995633	5.455093905	4.89E-08	17.8273587	3.268020498	intronic	SPTY2D1	.
3 month	rs185510569	2	222950143		G	A	421	0.003563	3	1.507358682	0.277399453	5.433892049	5.51E-08	19.58869056	3.604909775	intergenic	ACSL3;KCNE4	dist=5505;dist=102047
3 month	rs187213609	9	90653183		C	T	421	0.003563	3	1.269834136	0.233965931	5.427431802	5.72E-08	23.19753039	4.274126556	intergenic	DIRAS2;SYK	dist=10359;dist=148417
3 month	rs117280553	21	15834844		T	C	421	0.003563	3	1.465128514	0.27006967	5.425002059	5.80E-08	20.08741695	3.702748263	intronic	USP25	.
3 month	rs183816745	5	92336071		A	G	421	0.005938	5	1.692149037	0.312309144	5.418186024	6.02E-08	17.34879088	3.201955563	intergenic	ARRDC3-AS1;NR2F1-AS1	dist=915356;dist=1113174
3 month	rs180828621	10	122773893		G	A	421	0.007126	6	1.931459198	0.357196912	5.407267342	6.40E-08	15.13805735	2.799576273	ncRNA_intronic	DMBT1L1	.
3 month	rs142684595	3	55285273	0.77293998	T	C	421	0.005938	5	1.730096674	0.321166729	5.386911268	7.17E-08	16.77294307	3.113647549	intergenic	LINC02017;WNT5A	dist=96059;dist=180442
3 month	rs184785969	21	15799112		C	A	421	0.003563	3	1.446338922	0.269771603	5.361346058	8.26E-08	19.87364864	3.706839369	intronic	USP25	.
3 month	rs77180278	1	102496326		T	C	421	0.028504	24	3.885721783	0.727780259	5.339141503	9.34E-08	7.336199959	1.374041118	intergenic	OLFM3;COL11A1	dist=499092;dist=380141
3 month	rs189709453	3	177856520		G	A	421	0.003563	3	1.314463975	0.246356303	5.335621454	9.52E-08	21.65814876	4.05916142	ncRNA_intronic	LINC02015	.
3 month	rs111846247	5	95775939		T	C	421	0.003563	3	1.3787786	0.259425442	5.314739335	1.07E-07	20.48657718	3.854672052	intronic	RHOBTB3	.
3 month	rs145875128	4	32071514	0.719099998	G	A	421	0.003563	3	1.153690598	0.217089638	5.314351295	1.07E-07	24.47998599	4.606392132	ncRNA_intronic	LINC02506	.
3 month	rs75848314	5	95762636		T	C	421	0.003563	3	1.401525911	0.264060948	5.307584942	1.11E-07	20.0998481	3.787004507	intronic	RHOBTB3	.
3 month	rs190193113	2	52858640		G	A	421	0.003563	3	1.409901074	0.265819171	5.303985667	1.13E-07	19.95336019	3.761955902	intergenic	MIR4431;ASB3	dist=156025;dist=811339
3 month	rs183378658	2	52336233		C	T	421	0.003563	3	1.421941101	0.268359175	5.298649105	1.17E-07	19.74461693	3.726349214	ncRNA_intronic	LOC730100	.
3 month	rs143371352	13	46809699		C	T	421	0.003563	3	1.364496992	0.257779154	5.29327904	1.20E-07	20.53416252	3.879289636	intergenic	ESD;HTR2A	dist=12538;dist=21847
3 month	rs75186966	13	46821623		A	C	421	0.003563	3	1.377358459	0.260942954	5.278389159	1.30E-07	20.22813447	3.832255231	intergenic	ESD;HTR2A	dist=24462;dist=9923
3 month	rs532416695	2	176622062		G	A	421	0.003563	3	1.400161077	0.265436681	5.274934396	1.33E-07	19.87266561	3.767376828	intergenic	MIR1246;LINC01116	dist=21010;dist=7519
3 month	rs145080832	2	48246004		G	A	421	0.004751	4	1.617719031	0.30702202	5.269065159	1.37E-07	17.16184772	3.257095367	intergenic	FBXO11;FOXN2	dist=339506;dist=68295
3 month	rs182868205	3	177994660		C	T	421	0.003563	3	1.323927501	0.251356167	5.267137522	1.39E-07	20.95487681	3.978418395	intergenic	LINC02015;LINC01014	dist=95436;dist=424541
3 month	rs143538552	18	31470299		A	G	421	0.003563	3	1.44755014	0.274967228	5.264446053	1.41E-07	19.14572179	3.636797033	intronic	DSG3	.

3 month	rs7822082	8	54777660		T	C	421	0.330166	277	11.70263936	2.228199285	5.252061357	1.50E-07	2.357087802	0.448792891	intronic	RP1	.
3 month	rs72986533	6	142290121	0.786499977	T	C	421	0.008314	8	2.07386378	0.395157536	5.248194939	1.54E-07	13.28127256	2.530636288	intergenic	VTA1;ADGRG6	dist=65437;dist=11798
3 month	rs373746073	18	31478421		C	A	421	0.003563	3	1.441999195	0.274877768	5.245965155	1.55E-07	19.08471968	3.637980642	UTR3	DSG3	NM_001944:c.*21610>0
3 month	rs181193202	2	52300216		T	C	421	0.003563	3	1.397666205	0.26682604	5.238117712	1.62E-07	19.63120884	3.747760153	ncRNA_intronic	LOC730100	.
3 month	rs186767531	3	177921989		T	C	421	0.003563	3	1.293967205	0.247047316	5.237730262	1.63E-07	21.20132426	4.047807581	intergenic	LINC02015;LINC01014	dist=22765;dist=497212
3 month	rs140797780	8	22230279		C	T	421	0.003563	3	1.415632732	0.270894217	5.225776871	1.73E-07	19.29083956	3.691477848	intronic	PHYHIP	.
3 month	rs137880949	12	62912517	0.737010002	T	C	421	0.004751	3	1.263235635	0.24178583	5.224605748	1.75E-07	21.60840343	4.135891677	intronic	PPM1H	.
3 month	rs148532212	6	165086368		T	C	421	0.003563	3	1.406500453	0.26958124	5.217352852	1.81E-07	19.35354569	3.709456929	intergenic	MEAT6;C6orf118	dist=264305;dist=193296
3 month	rs141754456	12	19998198	0.798650026	T	C	421	0.007126	7	2.240311418	0.429474084	5.21640654	1.82E-07	12.14603335	2.328429207	intergenic	AEBP2;LINC02398	dist=475971;dist=16487
3 month	rs117498042	6	165100792		C	T	421	0.003563	3	1.405430664	0.269604623	5.212932361	1.86E-07	19.33547097	3.70913521	intergenic	MEAT6;C6orf118	dist=278729;dist=178872
3 month	rs184487573	6	167099983		A	G	421	0.007126	7	2.008477086	0.385701261	5.207338654	1.92E-07	13.50096351	2.592680141	intergenic	CEP43;CCR6	dist=47265;dist=11824
3 month	rs149298750	4	126237546		A	C	421	0.003563	3	1.219140188	0.234426329	5.200525875	1.99E-07	22.18405204	4.265732461	intergenic	MIR2054;INTU	dist=730239;dist=1395411
3 month	rs140277951	10	80599344		G	A	421	0.008314	6	1.967344821	0.378309358	5.200359916	1.99E-07	13.74631584	2.643339318	intronic	SH2D4B	.
3 month	rs558553658	2	15542600	0.761030018	C	T	421	0.004751	3	1.579685756	0.303851567	5.198873167	2.01E-07	17.10991069	3.291080613	intronic	NBAS	.
3 month	rs112679237	4	138219706	0.778339982	T	C	421	0.017815	18	3.098512369	0.596371661	5.195606321	2.04E-07	8.712027524	1.676806707	intronic	SLC7A11	.
3 month	rs116862847	14	63674959	0.740740001	C	T	421	0.007126	7	1.757642639	0.338759972	5.188460225	2.12E-07	15.3160369	2.951942625	intergenic	WDR89;SGPP1	dist=33088;dist=9258
3 month	rs528809914	13	112386942		G	A	421	0.003563	3	1.373302637	0.265345521	5.17552597	2.27E-07	19.50485519	3.768671108	intronic	SPACA7	.
3 month	rs112007361	11	99317649		A	C	421	0.030879	26	4.213518738	0.814161472	5.175286336	2.28E-07	6.356584678	1.228257582	intronic	CNTN5	.
3 month	rs546144116	19	19452530	0.741270006	C	T	421	0.002375	3	1.157037115	0.22363222	5.173839072	2.29E-07	23.13548147	4.471627577	intronic	GATAD2A	.
3 month	rs9643828	8	54616513		C	T	421	0.67696	279	-11.04343772	2.134612444	-5.173509483	2.30E-07	-2.423629403	0.468469114	intronic	RP1	.
3 month	rs17510814	12	28316036		A	C	421	0.007126	6	1.905807603	0.368446684	5.17254649	2.31E-07	14.03879235	2.714096892	intronic	CCDC91	.
3 month	rs113167689	12	28283029		C	T	421	0.007126	6	1.892111617	0.365850586	5.171815182	2.32E-07	14.13641354	2.733356286	intronic	CCDC91	.
3 month	rs140788628	20	15877856		C	A	421	0.010689	8	2.265436379	0.438089306	5.171174799	2.33E-07	11.80392839	2.282639604	intronic	MACROD2	.
3 month	rs141756120	12	28358163		A	C	421	0.008314	6	1.868183897	0.361717894	5.164753879	2.41E-07	14.27840304	2.764585375	intronic	CCDC91	.
3 month	rs117991215	12	28358540		T	C	421	0.008314	6	1.855759228	0.359817411	5.15750259	2.50E-07	14.33366601	2.77918736	intronic	CCDC91	.
3 month	rs191423619	4	125595472	0.741770029	G	T	421	0.002375	3	1.225954306	0.23771753	5.157189324	2.51E-07	21.69461097	4.206673365	intergenic	MIR2054;INTU	dist=88165;dist=2037485
3 month	rs113651406	4	997179		C	T	421	0.003563	3	1.369997433	0.265704278	5.156098506	2.52E-07	19.40540264	3.763582603	intronic	IDUA	.
3 month	rs187942235	18	29450465		C	T	421	0.003563	3	1.294384565	0.251912631	5.138228126	2.77E-07	20.39686581	3.969630251	intergenic	CDH2;MIR302F	dist=1273336;dist=848446
3 month	rs16920698	8	54765874		G	A	421	0.330166	277	11.42894943	2.226765953	5.132532863	2.86E-07	2.304926953	0.449081772	intronic	RP1	.
3 month	rs4737676	8	54766986		G	A	421	0.330166	277	11.42891023	2.226765217	5.132516953	2.86E-07	2.304920569	0.44908192	intronic	RP1	.
3 month	rs4737674	8	54749094		C	A	421	0.330166	277	11.42523599	2.226081333	5.132443196	2.86E-07	2.305595541	0.449219885	intronic	RP1	.
3 month	rs13277510	8	54761589		G	A	421	0.330166	277	11.42815338	2.22667847	5.132377007	2.86E-07	2.304947516	0.449099416	intronic	RP1	.
3 month	rs983248	8	54768232		C	T	421	0.330166	277	11.43612477	2.228588003	5.131556283	2.87E-07	2.302604284	0.448714611	intronic	RP1	.
3 month	rs1391463	8	54769316		T	G	421	0.330166	277	11.43612548	2.228595636	5.131539026	2.87E-07	2.302588654	0.448713075	intronic	RP1	.
3 month	rs4737201	8	54778898		C	T	421	0.330166	277	11.43417402	2.228701352	5.130420012	2.89E-07	2.301977341	0.44869179	intronic	RP1	.
3 month	rs549931083	12	20363352		A	C	421	0.003563	3	1.829003959	0.357281438	5.119224688	3.07E-07	14.32826937	2.798913946	intergenic	LINC02468;PDE3A	dist=235451;dist=5185
3 month	rs141281289	11	123822316	0.733070016	A	G	421	0.005938	6	1.657109443	0.323734372	5.118731856	3.08E-07	15.81151802	3.088952198	intergenic	OR6M1;TMEM225	dist=15967;dist=60603
3 month	rs184220112	2	48404604		C	A	421	0.004751	4	1.587567329	0.310301123	5.116215225	3.12E-07	16.48790432	3.22267606	intergenic	FOXN2;PPP1R21	dist=25309;dist=36162
3 month	rs11987234	8	54757269		A	G	421	0.328979	276	11.39664661	2.227939022	5.115331478	3.13E-07	2.295992587	0.448845319	intronic	RP1	.
3 month	rs13276543	8	54775614		G	T	421	0.328979	276	11.41118163	2.230992831	5.114844597	3.14E-07	2.29263157	0.448230934	intronic	RP1	.
3 month	rs111838310	2	74446364	0.780420005	C	A	421	0.004751	5	1.465896314	0.287002463	5.107608836	3.26E-07	17.79639376	3.48429066	intergenic	RTKN;INO80B-WBP1	dist=4427;dist=8659
3 month	rs80156375	7	18403592		A	C	421	0.009501	8	2.260074467	0.442534058	5.107119835	3.27E-07	11.5406255	2.25971308	intronic	HDAC9	.
3 month	rs1561297	8	54765978		A	C	421	0.332542	279	11.40464743	2.234979191	5.102798037	3.35E-07	2.28315237	0.447431459	intronic	RP1	.
3 month	rs112983626	2	74470023	0.782959998	G	A	421	0.004751	4	1.464072657	0.286917293	5.102768952	3.35E-07	17.78480792	3.485324945	intergenic	MOGS;MRPL53	dist=4641;dist=1959
3 month	rs12548593	8	54762057		G	T	421	0.332542	279	11.39766972	2.234516943	5.100730946	3.38E-07	2.282699606	0.447524018	intronic	RP1	.
3 month	rs10105693	8	54727912		C	T	421	0.328979	276	11.34015363	2.223938019	5.099132049	3.41E-07	2.292839101	0.449652819	intronic	RP1	.
3 month	rs13278605	8	54775611		C	T	421	0.328979	277	11.43432709	2.242496155	5.09892829	3.42E-07	2.273773481	0.445931645	intronic	RP1	.
3 month	rs2083123	8	54767758		C	T	421	0.332542	279	11.40261082	2.236761629	5.097821184	3.44E-07	2.279107938	0.447074908	intronic	RP1	.
3 month	rs17017794	4	90904734		T	C	421	0.039192	33	4.452559757	0.873737599	5.095991934	3.47E-07	5.83240545	1.144508375	intronic	CCSER1	.
3 month	rs74521112	11	99218416		G	T	421	0.032067	27	4.149638813	0.814414575	5.095241344	3.48E-07	6.256323868	1.227875864	intronic	CNTN5	.
3 month	rs116651654	4	162317591	0.708450019	C	T	421	0.007126	6	1.661626207	0.326131851	5.094952243	3.49E-07	15.62236937	3.066244515	intergenic	FSTL5;MIR4454	dist=153557;dist=775983
3 month	rs565682685	9	90460046		T	C	421	0.004751	4	1.368085345	0.268772597	5.090122138	3.58E-07	18.93839699	3.720617399	intergenic	LINC01508;LINC01501	dist=26557;dist=2386
3 month	rs144541665	1	103768107		G	A	421	0.002375	3	1.181121027	0.232374488	5.082834329	3.72E-07	21.87346108	4.303398392	intergenic	AMY1C;LOC100129138	dist=9415;dist=304916
3 month	rs193253461	15	58937154	0.781300008	A	G	421	0.013064	12	2.449615619	0.482149005	5.080619466	3.76E-07	10.53744676	2.074047629	intergenic	SLTM;RNF111	dist=3475;dist=50509
3 month	rs77867199	7	18402652		G	T	421	0.010689	9	2.377239679	0.468684022	5.072158572	3.93E-07	10.82212821	2.13363365	intronic	HDAC9	.
3 month	rs189360484	12	1761344		A	G	421	0.004751	4	1.530180886	0.301727521	5.071399785	3.95E-07	16.8078794	3.314248553	intronic	ADIPOR2	.
3 month	rs79182806	7	18394204		T	C	421	0.008314	7	2.11859485	0.418485528	5.062528352	4.14E-07	12.09726027	2.389568893	intronic	HDAC9	.
3 month	rs189890455	2	48428258		C	T	421	0.005938	5	1.690230255	0.334051451	5.059790188	4.20E-07	15.14673914	2.993550834	intergenic	FOXN2;PPP1R21	dist=48963;dist=12508
3 month	rs190294315	9	83330550		C	T	421	0.005938	5	1.754632626	0.347069598	5.055564181	4.29E-07	14.56642764	2.881266486	intronic	FRMD3	.
3 month	rs185819304	18	29421615		G	A	421	0.003563	3	1.281236388	0.25348417	5.05450257	4.32E-07	19.94011134	3.945019527	intergenic	CDH2;MIR302F	dist=1244486;dist=877296
3 month	rs148433854	19	30605571		G	A	421	0.003563	3	1.369429098	0.271144709	5.050547011	4.41E-07	18.62675851	3.688067545	intronic	ZNF536	.
3 month	rs576124203	3	142122354		T	G	421	0.003563	3	1.313230925	0.260150702	5.047962263	4.47E-07	19.4039925	3.843925823	intronic	TFDP2	.
3 month	rs143597860	1	103614521		A	G	421	0.002375	3	1.179951068	0.233861351	5.045515482	4.52E-07	21.57481541	4.276037896	intergenic	AMY2B;AMY2A	dist=34987;dist=2811
3 month	rs140782222	9	81413979	0.778829992	T	C	421	0.002375	3	1.1680569	0.231519821	5.045170192	4.53E-07	21.79152597	4.31928461	intergenic	LINC01507;TLE1	dist=1379424;dist=169704
3 month	rs1856085	1	103571923		G	A	421	0.002375	3	1.181208876	0.234330387	5.040784048	4.64E-07	21.51143997	4.267478979	intronic	AMY2B	.
3 month	rs186768950	19	18695314		C	A	421	0.002375	3	1.117877431	0.221839436	5.039128522	4.68E-07	22.71520611	4.507764788	intronic	CRTC1	.
3 month	rs182303755	15	59342593		A	C	421	0.013064	12	2.406496876	0.477647778	5.038224785	4.70E-07	10.54799166	2.093592905	intronic	MYO1E	.
3 month	rs545552231	3	142145508		C	T	421	0.003563	3	1.275833022	0.253432385	5.034214646	4.80E-07	19.8641332	3.945825635	intronic	TFDP2	.
3 month	rs78547898	22	32428291	0.730069995	G	A	421	0.003563	3	1.210956763	0.240651719	5.03198883	4.85E-07	20.90983951	4.155382737	intronic	BPIFC	.
3 month	rs78907958	7	18385394		T	G	421	0.008314	7	2.090002735	0.415774551	5.026769271	4.99E-07	12.09013217	2.405149614	intronic	HDAC9	.
3 month	rs113751774	4	23427231	0.70836997	C	T	421	0.007126	7	1.694246649	0.337089114	5.026109061	5.01E-07	14.91032743	2.966574592	intergenic	GBA3;PPARGC1A	dist=607659;dist=364790
3 month	rs10958428	8	54773081		A	G	421	0.333729	280	11.27671394	2.243728439	5.025881807	5.01E-07	2.239968848	0.445686734	intronic	RP1	.
3 month	rs17776100	7	6386848		G	A	421	0.029691	25	3.798893519	0.756560221	5.021270496	5.13E-07	6.63697397	1.321771845	intronic	RAC1	.

3 month	rs185464792	19	18686561		C	T	421	0.002375	3	1.114587174	0.222127708	5.017776414	5.23E-07	22.58960154	4.501914727	intronic	CRTC1	.
3 month	rs117025967	10	19918123		C	A	421	0.013064	11	2.602875018	0.518823163	5.016882829	5.25E-07	9.669735635	1.927439002	intronic	PLXDC2	.
3 month	rs188028357	7	101025144		C	T	421	0.002375	3	1.150125852	0.229467369	5.012154263	5.38E-07	21.84255775	4.357918094	intronic	MUC17	.
3 month	rs148556485	9	81408911	0.779910028	A	C	421	0.002375	3	1.17470554	0.234401563	5.011508991	5.40E-07	21.38001525	4.266183157	intergenic	LINC01507;TLE1	dist=1374356;dist=174772
3 month	rs184613584	17	50430860		A	C	421	0.007126	6	1.77626107	0.354456155	5.011229298	5.41E-07	14.13779737	2.821223402	intronic	ACSF2	.
3 month	rs528288879	2	65648796	0.785229981	C	T	421	0.005938	6	1.697856008	0.338843001	5.010745399	5.42E-07	14.78780847	2.951219288	intergenic	SPRED2;MIR4778	dist=216197;dist=709451
3 month	rs1437782	8	54720202		C	T	421	0.330166	278	11.10161994	2.217354849	5.006695229	5.54E-07	2.2579585	0.450987807	exonic	RP1	.
3 month	rs534845494	13	57639730		A	G	421	0.004751	5	1.571808362	0.313987509	5.005958262	5.56E-07	15.94317648	3.184840074	intronic	PCDH17	.
3 month	rs1008091735	19	30599192		T	C	421	0.003563	3	1.355816116	0.270944036	5.004044884	5.61E-07	18.46892428	3.690799086	intronic	ZNF536	.
3 month	rs567982164	5	25631188	0.711179972	G	A	421	0.002375	3	1.14494858	0.228986639	5.000067194	5.73E-07	21.8356286	4.367067031	intergenic	LINC02211;CDH9	dist=328908;dist=1249409
3 month	rs79213709	11	99222724		G	A	421	0.032067	26	4.048879944	0.810020577	4.998490236	5.78E-07	6.17081884	1.23453654	intronic	CNTN5	.
3 month	rs559008174	19	18765249	0.769429982	C	T	421	0.004751	5	1.594517191	0.319680825	4.987841211	6.11E-07	15.60256615	3.12812006	intronic	CRTC1	.
3 month	rs541288561	19	18758635	0.77651	T	G	421	0.004751	5	1.591688559	0.31930164	4.984905675	6.20E-07	15.61190124	3.131834835	intronic	CRTC1	.
3 month	rs190251199	14	105124240	0.736329973	T	C	421	0.003563	3	1.240668633	0.248987137	4.982862353	6.27E-07	20.01252919	4.016271726	intergenic	LINC02298;JAG2	dist=24744;dist=16758
3 month	rs138215817	14	22173619		A	G	421	0.004751	4	1.521988034	0.305467544	4.982486896	6.28E-07	16.31101896	3.273670217	intergenic	OR4E1;LOC105370401	dist=502284;dist=206292
3 month	rs111407636	5	95744325		C	T	421	0.003563	3	1.331610247	0.26725959	4.98246012	6.28E-07	18.64277396	3.741680518	intronic	RHOBTB3	.
3 month	rs73085348	3	42669729		A	G	421	0.011876	11	2.448275953	0.491389455	4.982353469	6.28E-07	10.13931704	2.035045707	intergenic	ZBTB47;KLHL40	dist=2149;dist=15808
3 month	rs2375537	8	54706948		C	T	421	0.332542	280	11.08575521	2.225246111	4.981810848	6.30E-07	2.238768477	0.449388495	intronic	RP1	.
3 month	rs1437781	8	54717292		T	C	421	0.332542	280	11.08371192	2.225151228	4.981105007	6.32E-07	2.23854673	0.449407657	intronic	RP1	.
3 month	rs2274997	1	229668899		A	G	421	0.041568	35	4.42362483	0.889790308	4.971536315	6.64E-07	5.587312281	1.123860297	intergenic	URB2;LINC01682	dist=8699;dist=206651
3 month	rs147032554	1	186179732		T	G	421	0.003563	3	1.360299196	0.273670575	4.970571635	6.68E-07	18.16260897	3.654028209	intronic	HMCN1	.
3 month	rs111676272	5	95758594		C	A	421	0.003563	3	1.327188035	0.267072797	4.969386812	6.72E-07	18.6068625	3.744297477	intronic	RHOBTB3	.
3 month	rs2274996	1	229668791		C	T	421	0.041568	35	4.420411647	0.88962935	4.968823976	6.74E-07	5.585274332	1.124063633	intergenic	URB2;LINC01682	dist=8591;dist=206759
3 month	rs2891865	1	229670621		A	G	421	0.041568	35	4.419830983	0.88984663	4.966958165	6.80E-07	5.581813763	1.123789164	intergenic	URB2;LINC01682	dist=10421;dist=204929
3 month	rs2385790	1	229671745		C	T	421	0.041568	35	4.418931852	0.88972149	4.966646197	6.81E-07	5.582248215	1.123947226	intergenic	URB2;LINC01682	dist=11545;dist=203805
3 month	rs12024557	1	229676610		A	C	421	0.042755	35	4.42434228	0.891299524	4.963923079	6.91E-07	5.569309691	1.121957291	intergenic	URB2;LINC01682	dist=16410;dist=198940
3 month	rs78296164	9	132391328		C	T	421	0.008314	7	2.121725249	0.427501046	4.963087859	6.94E-07	11.60953385	2.339175566	intronic	TTF1	.
3 month	rs570407448	19	18769220	0.776769996	G	A	421	0.004751	5	1.587863075	0.320089569	4.960683605	7.02E-07	15.4977984	3.124125552	intronic	CRTC1	.
3 month	rs76777840	2	48085811		G	A	421	0.005938	5	1.714828291	0.345687108	4.960637102	7.03E-07	14.35007842	2.892789399	intergenic	FBXO11;FOXN2	dist=179313;dist=228488
3 month	rs528609331	11	125972300		C	T	421	0.003563	3	1.256070429	0.253271825	4.959376858	7.07E-07	19.58124182	3.948327054	intronic	CDON	.
3 month	rs182437250	12	63214686	0.713500023	T	C	421	0.004751	4	1.307028829	0.263613284	4.958129612	7.12E-07	18.80834508	3.793435539	intergenic	AVPR1A;DPY19L2	dist=63485;dist=344227
3 month	rs147601511	8	22464806		A	G	421	0.004751	4	1.494160188	0.301407236	4.957280417	7.15E-07	16.44711814	3.317770381	intronic	PPP3CC	.
3 month	rs858397	8	54702130		A	G	421	0.331354	278	11.01774054	2.2226993	4.956919066	7.16E-07	2.230134803	0.449903412	intronic	RP1	.
3 month	rs75334617	10	101196395		G	A	421	0.038005	32	4.196585386	0.847261284	4.953118316	7.30E-07	5.846034048	1.180273451	intergenic	LINC01514;LBX1	dist=2248;dist=30581
3 month	rs4562666	1	229689023		T	C	421	0.042755	36	4.44283225	0.897203314	4.951867853	7.35E-07	5.519225993	1.114574572	intergenic	URB2;LINC01682	dist=28823;dist=186527
3 month	rs382476	8	54678415		G	A	421	0.666271	288	-10.90475865	2.202374434	-4.951364528	7.37E-07	-2.24819379	0.454055398	intronic	RP1	.
3 month	rs384543	8	54679049		G	A	421	0.666271	288	-10.90475865	2.202374434	-4.951364528	7.37E-07	-2.24819379	0.454055398	intronic	RP1	.
3 month	rs446222	8	54662400		G	A	421	0.666271	288	-10.90656484	2.202740432	-4.951361803	7.37E-07	-2.247819003	0.453979954	intronic	RP1	.
3 month	rs384127	8	54684929		G	A	421	0.666271	288	-10.90492469	2.202409773	-4.951360471	7.37E-07	-2.248155875	0.454048112	intronic	RP1	.
3 month	rs12045643	1	229698303		C	T	421	0.042755	36	4.472324416	0.903269355	4.951263308	7.37E-07	5.481491517	1.107089479	intergenic	URB2;LINC01682	dist=38103;dist=177247
3 month	rs147630370	4	86529522		T	C	421	0.004751	4	1.530518883	0.309272178	4.948776482	7.47E-07	16.00136328	3.23339786	intergenic	MAPK10;MIR4452	dist=76327;dist=12960
3 month	rs532513136	8	134801505		C	A	421	0.003563	3	1.227599638	0.248065954	4.948682463	7.47E-07	19.94905941	4.031185989	upstream	MIR30B	dist=898
3 month	rs184265355	13	107359004		A	C	421	0.005938	6	1.694033349	0.342665495	4.94369399	7.67E-07	14.42717187	2.918297916	intronic	FAM155A	.
3 month	rs148248743	3	136415753	0.708679974	C	T	421	0.002375	3	1.069833829	0.216429281	4.943110391	7.69E-07	22.83937904	4.620446891	intronic	STAG1	.
3 month	rs72983831	6	141985963	0.75375998	T	G	421	0.005938	6	1.556807425	0.31516421	4.939670734	7.83E-07	15.67332386	3.17294911	intergenic	MIR4465;NMBR	dist=1302080;dist=88521
3 month	rs80292573	15	59142887		T	G	421	0.034442	30	3.721656224	0.753598905	4.938510655	7.87E-07	6.553234912	1.326965834	intronic	MYO1E	.
3 month	rs111927235	2	74256827		A	G	421	0.004751	4	1.423024126	0.288923909	4.925255689	8.43E-07	17.04689553	3.461118894	intronic	SLC4A5	.
3 month	rs369623	8	54659380		A	C	421	0.666271	287	-10.8407396	2.201888659	-4.923382276	8.51E-07	-2.235981486	0.45415557	intronic	RP1	.
3 month	rs188720948	3	150352093		T	C	421	0.003563	3	1.368098189	0.277991774	4.921362129	8.59E-07	17.7032653	3.597228742	intergenic	LINC01214;TSC22D2	dist=28346;dist=56205
3 month	rs148998974	22	40224526		A	G	421	0.005938	5	1.667947442	0.339010604	4.920045046	8.65E-07	14.51295325	2.949760239	intronic	TNRC6B	.
3 month	rs144954214	16	76145464		A	G	421	0.002375	3	1.106696587	0.225254382	4.913096823	8.96E-07	21.81132631	4.439425294	intergenic	CPHXL;CNTNAP4	dist=418974;dist=131937
3 month	rs145439370	15	58587566		T	C	421	0.030879	26	3.500007735	0.712559708	4.911879937	9.02E-07	6.893288912	1.403391166	intergenic	LIPC;ADAM10	dist=17722;dist=1243
3 month	rs75689761	7	18366950		C	T	421	0.008314	7	2.0345491	0.414387092	4.909779146	9.12E-07	11.84829172	2.413202584	intronic	HDAC9	.
3 month	rs113221952	1	103288418		A	G	421	0.021378	19	3.040841012	0.619495467	4.908576695	9.17E-07	7.923507043	1.614216816	intergenic	COL11A1;LOC101928436	dist=179896;dist=205628
3 month	rs61434999	7	18378728		A	G	421	0.008314	7	2.033933624	0.414449316	4.907556958	9.22E-07	11.84115106	2.412840272	intronic	HDAC9	.
3 month	rs433324	8	54652049		A	G	421	0.666271	287	-10.71178821	2.182726874	-4.907525693	9.22E-07	-2.248346209	0.458142524	intronic	RP1	.
3 month	rs528140343	11	125849332		A	C	421	0.003563	3	1.315991741	0.268284941	4.905201681	9.33E-07	18.28355207	3.727380292	intergenic	PATE4;HYLS1	dist=9260;dist=34282
3 month	rs34270375	1	88905019		G	A	421	0.026128	21	3.119890277	0.63606437	4.904991419	9.34E-07	7.711470175	1.572167924	intergenic	GTF2B;KYAT3	dist=13452;dist=30754
3 month	rs75773869	7	18371222		G	T	421	0.008314	7	2.02750066	0.413389996	4.904571175	9.36E-07	11.86427156	2.41902322	intronic	HDAC9	.
3 month	rs79602997	7	18370627		G	A	421	0.008314	7	2.026922666	0.413391185	4.903158896	9.43E-07	11.86082112	2.419016265	intronic	HDAC9	.
3 month	rs56224400	6	97644799		T	C	421	0.016627	14	2.845683986	0.580546566	4.901732531	9.50E-07	8.443306398	1.722514712	ncRNA_intronic	LOC101927314	.
3 month	rs186532456	1	18621834		C	T	421	0.003563	3	1.189953152	0.242873045	4.899486277	9.61E-07	20.17303433	4.117377453	intergenic	KLHDC7A;PAX7	dist=135848;dist=9012
3 month	rs118093638	11	18696777		C	T	421	0.005938	5	1.681215257	0.343292823	4.897321308	9.72E-07	14.26572587	2.912965064	intergenic	SPTY2D1;TMEM86A	dist=62435;dist=2002
3 month	rs77353774	12	28095919		G	A	421	0.007126	6	1.82528489	0.373198242	4.890925749	1.00E-06	13.1054362	2.679541026	intergenic	PTHLH;LOC729291	dist=123186;dist=89706
3 month	rs532730683	9	1784492		G	T	421	0.003563	3	1.275657724	0.260996603	4.887641107	1.02E-06	18.72683804	3.831467497	intergenic	DMRT2;SMARCA2	dist=726938;dist=230855
3 month	rs185139807	22	40198777		G	A	421	0.005938	5	1.65498418	0.338624126	4.887379825	1.02E-06	14.43305263	2.953126858	intronic	TNRC6B	.
3 month	rs146333745	18	57830225	0.779890001	C	T	421	0.003563	4	1.368613476	0.280282935	4.882971117	1.04E-06	17.42157837	3.567823349	intergenic	ATP8B1;NEDD4L	dist=26910;dist=214001
3 month	rs541680196	22	40132086		G	A	421	0.005938	5	1.654258232	0.339093792	4.878468054	1.07E-06	14.38678079	2.949036589	intronic	TNRC6B	.
3 month	rs12036586	1	229690631		G	A	421	0.047506	40	4.551491816	0.933583971	4.875289164	1.09E-06	5.222121756	1.071140927	intergenic	URB2;LINC01682	dist=30431;dist=184919
3 month	rs529523094	16	77681654		A	G	421	0.003563	3	1.307832421	0.26837178	4.873211417	1.10E-06	18.15843462	3.726174193	intergenic	ADAMTS18;NUDT7	dist=246620;dist=40838
3 month	rs562032622	1	18632839		A	C	421	0.003563	3	1.126818759	0.231494862	4.867575676	1.13E-06	21.0267115	4.319750303	intronic	PAX7	.
3 month	rs78225611	7	18367841		A	C	421	0.009501	8	2.147570798	0.441262518	4.866877908	1.13E-06	11.02943875	2.266224663	intronic	HDAC9	.
3 month	rs77346868	7	18366976		A	G	421	0.009501	8	2.148608551	0.441537806	4.866193838	1.14E-06	11.02101286	2.264811725	intronic	HDAC9	.

3 month	rs184098071	2	176251692		G	A	421	0.003563	3	1.695892029	0.34869637	4.863520744	1.15E-06	13.94772404	2.86782452	intergenic	HOXD1;MTX2	dist=60785;dist=17750
3 month	rs118183140	21	34105187		C	T	421	0.02019	17	3.113677552	0.640523765	4.861142898	1.17E-06	7.589324801	1.561222322	UTR3	SLC5A3	NM_006933:c.*78320>0
3 month	rs113625788	22	19981659		C	T	421	0.008314	7	2.000739147	0.411621625	4.860626906	1.17E-06	11.80848286	2.429415606	exonic	ARVCF	.
3 month	rs116189766	2	125636287	0.723290026	T	C	421	0.002375	3	1.114549479	0.229338184	4.85985133	1.17E-06	21.19076397	4.360372887	intergenic	CNTNAP5;LINC01941	dist=715069;dist=473813
3 month	rs76098744	1	111806750		C	T	421	0.016627	14	2.811615503	0.579024995	4.855775707	1.20E-06	8.386124522	1.727041163	intronic	KCND3	.
3 month	rs74683551	1	111811796		G	A	421	0.016627	14	2.810351968	0.579019309	4.853641189	1.21E-06	8.38252043	1.727058121	intronic	KCND3	.
3 month	rs185855183	12	101111348		C	T	421	0.003563	3	1.256842073	0.258975211	4.853136594	1.22E-06	18.73977272	3.861373435	intronic	ANO4	.
3 month	rs405226	8	54679776		A	G	421	0.662708	291	-10.75682372	2.217202771	-4.851529081	1.23E-06	-2.188130533	0.45101874	intronic	RP1	.
3 month	rs3098298	8	54670278		C	T	421	0.662708	291	-10.75741494	2.217551047	-4.851033737	1.23E-06	-2.187563503	0.450947906	intronic	RP1	.
3 month	rs367179	8	54675056		T	C	421	0.662708	291	-10.75741494	2.217551047	-4.851033737	1.23E-06	-2.187563503	0.450947906	intronic	RP1	.
3 month	rs432393	8	54667738		C	T	421	0.662708	291	-10.75654812	2.217474543	-4.850810194	1.23E-06	-2.187538166	0.450963464	intronic	RP1	.
3 month	rs117185941	21	37394182		G	A	421	0.005938	5	1.529223098	0.31531516	4.849824212	1.24E-06	15.38087864	3.171430132	intronic	DYRK1A	.
3 month	rs139360368	5	74076284		A	C	421	0.003563	4	1.280137576	0.264060375	4.847897286	1.25E-06	18.35904869	3.78701272	intergenic	ARHGEF28;LINC01335	dist=134291;dist=230126
3 month	rs184117160	15	59112107	0.770810008	C	T	421	0.014252	11	2.271648443	0.468677492	4.846933088	1.25E-06	10.34172362	2.133663377	intronic	CCNB2	.
3 month	rs145676540	3	2002951		C	T	421	0.005938	5	1.68032939	0.346683687	4.846866048	1.25E-06	13.98065798	2.884473769	intergenic	CNTN6;CNTN4	dist=598734;dist=95852
3 month	rs556293455	2	176085692		G	A	421	0.003563	3	1.630922146	0.336702149	4.843812709	1.27E-06	14.38604633	2.969984018	intergenic	EVX2;HOXD13	dist=1730;dist=7029
3 month	rs150027952	9	100623134	0.704400003	A	G	421	0.002375	3	1.072594942	0.221501288	4.842386925	1.28E-06	21.86166485	4.514646431	intergenic	CAVIN4;PLPPR1	dist=34747;dist=405593
3 month	rs138217865	15	93849653		C	T	421	0.004751	4	1.402299902	0.28969226	4.840653664	1.29E-06	16.70964097	3.451938959	intergenic	LOC105370980;LINC02207	dist=641605;dist=6907
3 month	rs112475378	3	1584977	0.749329984	T	C	421	0.016627	14	2.445371748	0.506000821	4.832742644	1.35E-06	9.550859286	1.976281377	intergenic	CNTN6;CNTN4	dist=180760;dist=513826
3 month	rs146007933	3	28185932		T	C	421	0.021378	18	3.108745063	0.643323442	4.832320511	1.35E-06	7.511494525	1.554428045	intergenic	LINC01980;CMC1	dist=325607;dist=55687
3 month	rs192443987	3	135813503		G	A	421	0.004751	4	1.31410558	0.27206124	4.830183004	1.36E-06	17.75402845	3.675643022	intergenic	EPHB1;PPP2R3A	dist=553038;dist=152225
3 month	rs149493615	1	79414404		G	A	421	0.008314	8	1.98260972	0.410759865	4.826688019	1.39E-06	11.750632	2.434512435	intergenic	ADGRL4;LINC01781	dist=407674;dist=1121351
3 month	rs140706881	10	94457778		G	A	421	0.004751	4	1.226743807	0.254265992	4.824647601	1.40E-06	18.97480496	3.932889306	intronic	TBC1D12	.
3 month	rs142894171	2	150581757		G	T	421	0.003563	3	1.287888043	0.266960796	4.82425907	1.41E-06	18.07103941	3.74586836	intergenic	LINC01920;LINC02612	dist=9536;dist=47140
3 month	rs17746486	2	95056864	0.71280998	C	T	421	0.032067	29	3.355977065	0.696974974	4.815061071	1.47E-06	6.908513578	1.434771746	intergenic	MAL;MRPS5	dist=2872;dist=28507
3 month	rs180764936	12	101104843		T	C	421	0.003563	3	1.197190428	0.248754746	4.812734014	1.49E-06	19.34730528	4.020023799	intronic	ANO4	.
3 month	rs190806532	12	48468364	0.728540003	G	T	421	0.002375	3	1.082541559	0.225298273	4.804926128	1.55E-06	21.32695498	4.438560431	intergenic	ZNF641;ANP32D	dist=117118;dist=4195
3 month	rs147393020	10	123019758		A	G	421	0.005938	5	1.619245544	0.337859644	4.792657467	1.65E-06	14.1853505	2.959808958	intronic	ACADSB	.
3 month	rs79539453	11	125396457		C	T	421	0.002375	3	1.102705134	0.230127137	4.791721423	1.65E-06	20.82206158	4.345424064	intronic	PKNOX2	.
3 month	rs371245624	5	163453950		T	C	421	0.003563	3	1.17843308	0.245958889	4.791179069	1.66E-06	19.47959308	4.065720108	UTR3	NUDCD2	NM_001329991:c.*170>0;NM
3 month	rs536803366	8	121989172	0.708639979	T	C	421	0.002375	3	1.194921552	0.249406377	4.791062547	1.66E-06	19.20986385	4.009520573	intergenic	HAS2-AS1;SMILR	dist=343847;dist=425155
3 month	rs150077525	13	57405415	0.75563997	A	G	421	0.007126	7	1.765848692	0.368578785	4.790966716	1.66E-06	12.99848746	2.713124141	intergenic	PRR20E;PCDH17	dist=235197;dist=226329
3 month	rs371879555	12	22863028	0.786769986	T	C	421	0.003563	3	1.139634583	0.238093921	4.786491727	1.70E-06	20.10337646	4.200023233	intergenic	ETNK1;LOC101928441	dist=172363;dist=312608
3 month	rs571986619	6	84969114	0.767470002	A	G	421	0.002375	3	1.102004226	0.230254532	4.786026206	1.70E-06	20.78580673	4.343019831	intergenic	TBX18;LINC02535	dist=204516;dist=418105
3 month	rs183586634	21	37390730		G	A	421	0.005938	5	1.598766374	0.334213375	4.783669629	1.72E-06	14.31322017	2.992100476	intronic	DYRK1A	.
3 month	rs143811231	1	79502446		T	C	421	0.008314	8	1.975347907	0.413110429	4.78164619	1.74E-06	11.57474094	2.420660266	intergenic	ADGRL4;LINC01781	dist=495716;dist=1033309
3 month	rs2365739	1	62018790		G	A	421	0.021378	18	3.317349453	0.694985007	4.773267649	1.81E-06	6.868159166	1.43887996	intronic	PATJ	.
3 month	rs146526206	4	90071867		T	C	421	0.017815	15	2.815290022	0.58984771	4.772909977	1.82E-06	8.091766558	1.695352855	intergenic	MMRN1;CCSER1	dist=117257;dist=55527
3 month	rs567383525	8	114017265		C	T	421	0.003563	3	1.282541719	0.268838431	4.770678479	1.84E-06	17.74552267	3.719706274	intergenic	CSMD3;TRPS1	dist=580326;dist=1391230
3 month	rs149425014	15	58659461	0.716620028	T	C	421	0.026128	22	3.013015463	0.631629727	4.770224288	1.84E-06	7.552247919	1.583206043	intronic	ADAM10	.
3 month	rs559559983	1	246813830		C	A	421	0.007126	5	1.645339674	0.345154188	4.76697004	1.87E-06	13.81113197	2.897255878	intergenic	LINC01341;AHCTF1	dist=22344;dist=25268
3 month	rs117166500	7	17013154		G	T	421	0.007126	7	1.813506071	0.380670761	4.763975219	1.90E-06	12.51468647	2.626941975	intergenic	AGR3;AHR	dist=131171;dist=285498
3 month	rs145766563	1	185160370		G	A	421	0.003563	4	1.34508864	0.282384562	4.76332215	1.90E-06	16.86821019	3.541270076	intronic	SWT1	.
3 month	rs540065886	9	2770228	0.797429979	T	C	421	0.003563	3	1.13736568	0.23877586	4.763319367	1.90E-06	19.94891509	4.188028045	intergenic	KCNV2;PUM3	dist=40191;dist=33927
3 month	rs118084887	21	37491518		T	C	421	0.005938	5	1.518241868	0.31880157	4.762341255	1.91E-06	14.93826162	3.136747415	intronic	DYRK1A	.
3 month	rs140420703	1	102337928	0.700510025	T	G	421	0.004751	5	1.375529427	0.288838786	1.375529427	1.91E-06	16.48765399	3.462138915	intergenic	OLFM3;COL11A1	dist=340694;dist=538539
3 month	rs568321148	2	29647482		T	G	421	0.003563	3	1.279867135	0.268808164	4.761265858	1.92E-06	17.71250463	3.720125102	intronic	ALK	.
3 month	rs55844051	7	23320744		T	C	421	0.003563	3	1.232383167	0.25888714	4.760310483	1.93E-06	18.3875896	3.862687038	intronic	IGF2BP3	.
3 month	rs187236873	5	92234630		G	A	421	0.007126	6	1.81660653	0.381998785	4.755529603	1.98E-06	12.44906998	2.617809375	intergenic	ARRDC3-AS1;NR2F1-AS1	dist=813915;dist=1214615
3 month	rs1812506	8	54763541		A	G	421	0.345606	291	10.64127813	2.239050947	4.752584188	2.01E-06	2.122588678	0.446617796	intronic	RP1	.
3 month	rs539713344	7	100877165	0.772019982	G	A	421	0.002375	3	1.125805228	0.236990667	4.750420101	2.03E-06	20.04475604	4.219575451	intronic	SRRT	.
3 month	rs536781978	2	29510815		A	G	421	0.003563	3	1.282866581	0.270080884	4.749934759	2.03E-06	17.58708236	3.702594509	intronic	ALK	.
3 month	rs76327548	12	100789188		G	A	421	0.013064	11	2.458077916	0.517503975	4.749872531	2.04E-06	9.178427144	1.932352307	intergenic	GAS2L3;ANO4	dist=160900;dist=5588
3 month	rs149421869	2	53256291		G	T	421	0.008314	8	1.92083422	0.404523583	4.748386252	2.05E-06	11.73821862	2.472043763	intergenic	MIR4431;ASB3	dist=553676;dist=413688
3 month	rs75024143	21	21784226		G	T	421	0.014252	12	2.293452396	0.483452813	4.743901232	2.10E-06	9.812542411	2.068454196	ncRNA_intronic	LINC01425	.
3 month	rs2375536	8	54728162		T	C	421	0.347981	292	10.56754692	2.229301935	4.740294148	2.13E-06	2.126358065	0.448570911	intronic	RP1	.
3 month	rs141326851	6	134511989		A	C	421	0.016627	14	2.616968305	0.552323641	4.738106631	2.16E-06	8.578496884	1.810532677	intergenic	LINC01010;LOC101928304	dist=7969;dist=13329
3 month	rs187518659	1	98990189		T	G	421	0.009501	8	1.921562765	0.405573328	4.737892339	2.16E-06	11.68196232	2.465645372	intronic	PLPPR5	.
3 month	rs146479102	2	65598625	0.738789976	G	A	421	0.005938	5	1.448302352	0.305828916	4.735661917	2.18E-06	15.48467677	3.269801992	intergenic	SPRED2;MIR4778	dist=166026;dist=759622
3 month	rs16850124	1	229695584		T	C	421	0.043943	37	4.326094288	0.91378287	4.73426941	2.20E-06	5.180956622	1.094351878	intergenic	URB2;LINC01682	dist=35384;dist=179966
3 month	rs115348382	1	9595845		G	A	421	0.003563	3	1.230366386	0.259928152	4.733486451	2.21E-06	18.21074945	3.847216981	intronic	TMEM201	.
3 month	rs184200893	2	69033781		C	T	421	0.003563	3	1.121211249	0.237019909	4.730451775	2.24E-06	19.95803558	4.219054866	intronic	ANTXR1	.
3 month	rs148781275	11	103769875	0.774079978	A	G	421	0.004751	5	1.423809766	0.301038131	4.729665848	2.25E-06	15.71118528	3.321838325	intergenic	DYNC2H1;MIR4693	dist=290012;dist=80031
3 month	rs139943877	3	155733500		G	A	421	0.007126	7	1.713092922	0.362264246	4.728849011	2.26E-06	13.0535902	2.760415942	intronic	PLCH1	.
3 month	rs546409459	11	125885094		A	G	421	0.003563	3	1.180777655	0.249820856	4.726497517	2.28E-06	18.91954737	4.002868361	intronic	HYLS1	.
3 month	rs186649043	3	175214503	0.769129992	C	T	421	0.003563	3	1.162234876	0.245948741	4.725516671	2.30E-06	19.21342086	4.065887859	intronic	NAALADL2	.
3 month	rs80203220	3	123003484		C	T	421	0.005938	6	1.662045235	0.351841788	4.723842626	2.31E-06	13.42604201	2.842186558	intronic	SEMA5B	.
3 month	rs541508507	2	169166755		G	A	421	0.004751	4	1.442660176	0.305446461	4.723119638	2.32E-06	15.46300334	3.273896179	intronic	LRP2	.
3 month	rs529011661	10	20083387		G	A	421	0.004751	4	1.425272643	0.301883703	4.721263948	2.34E-06	15.63934688	3.312533901	intronic	PLXDC2	.
3 month	rs182959028	22	45427152		T	C	421	0.008314	7	1.863907765	0.395028955	4.718407959	2.38E-06	11.94446103	2.531460004	intronic	RIBC2	.
3 month	rs146207930	9	126280057		A	G	421	0.007126	6	1.87537986	0.397610416	4.716626584	2.40E-06	11.86243214	2.515024654	intergenic	LOC101929116;MVB12B	dist=4142;dist=46772
3 month	rs290120	5	163841238		T	G	421	0.007126	6	1.664921355	0.353031552	4.716069562	2.40E-06	13.3587764	2.832608007	intergenic	MAT2B;LINC02143	dist=321884;dist=607184
3 month	rs531769270	4	153489891	0.744459987	T	C	421	0.003563	3	1.339792194	0.284142497	4.715212285	2.41E-06	16.59453383	3.519360917	intronic	TMEM131L	.

3 month	rs185620578	12	48175616	0.752129972	C	T	421	0.002375	3	1.072824946	0.227528468	4.715124023	2.42E-06	20.72322668	4.39505442	intergenic	ASB8;CCDC184	dist=18101;dist=8028
3 month	rs62447184	7	36534898	0.730589986	G	A	421	0.042755	43	4.329544629	0.918817438	4.712083651	2.45E-06	5.128422094	1.088355486	intronic	AOAH	.
3 month	rs118184666	12	20271815		G	A	421	0.007126	6	2.026245701	0.430149631	4.710560123	2.47E-06	10.95098026	2.324772421	intergenic	LINC02468;PDE3A	dist=143914;dist=96722
3 month	rs10494861	1	205362746		G	A	421	0.003563	3	1.156337638	0.245524493	4.709663065	2.48E-06	19.18204982	4.072913404	intergenic	KLHDC8A;LEMD1-AS1	dist=5656;dist=10506
3 month	rs183817723	16	59268871	0.79569	C	T	421	0.003563	4	1.261174136	0.267795557	4.709466243	2.48E-06	17.5860507	3.734191901	intergenic	GOT2;APOOP5	dist=534555;dist=485270
3 month	rs12502861	4	2424578		T	C	421	0.010689	9	1.987294963	0.422008794	4.709131639	2.49E-06	11.15884718	2.369618867	intronic	CFAP99	.
3 month	rs191792521	3	195919734	0.757189989	G	A	421	0.008314	6	1.587589638	0.337332296	4.706307863	2.52E-06	13.95154842	2.964435992	intergenic	TNK2-AS1;SDHAP1	dist=6470;dist=40187
3 month	rs149949098	11	95366702		G	A	421	0.016627	13	2.587327209	0.549803064	4.705916312	2.53E-06	8.559276254	1.818833079	intergenic	LOC100129203;FAM76B	dist=132298;dist=402251
3 month	rs74343174	5	162066176	0.766919971	C	A	421	0.005938	5	1.498573008	0.318559187	4.704221604	2.55E-06	14.76718237	3.139134082	intergenic	LINC01202;GABRG2	dist=64980;dist=1289
3 month	rs189765693	4	4323066		T	C	421	0.004751	4	1.425732565	0.30309955	4.70384257	2.55E-06	15.51913413	3.299246074	intergenic	ZBTB49;NSG1	dist=1283;dist=63466
3 month	rs189912648	5	135429749		C	T	421	0.003563	4	1.303529678	0.277126356	4.703737665	2.55E-06	16.97325991	3.608462274	intergenic	MACROH2A1;DCANP1	dist=29862;dist=14465
3 month	rs151323346	12	20859090		T	C	421	0.005938	4	1.39062978	0.295827471	4.700813538	2.59E-06	15.89038883	3.380348678	intronic	SLCO1B3;SLCO1B3-SLCO1B7	.
3 month	rs536023430	7	147167980	0.788429976	T	C	421	0.003563	3	1.137129093	0.242081012	4.697308079	2.64E-06	19.40386834	4.130848564	intronic	CNTNAP2	.
3 month	rs144026361	15	40956471	0.714779973	C	T	421	0.004751	5	1.365790883	0.290922074	4.694696631	2.67E-06	16.13729945	3.437346588	UTR3	CHAC1	NM_001142776:c.*6970>0;NM
3 month	rs186142189	2	67475575		G	A	421	0.007126	6	1.7290309	0.36845255	4.692682681	2.70E-06	12.73619271	2.714053683	intergenic	ETAA1;LINC01812	dist=63486;dist=320479
3 month	rs142549310	2	169173996		C	T	421	0.004751	4	1.443948619	0.307707803	4.692596694	2.70E-06	15.25017126	3.249836338	exonic	LRP2	.
3 month	rs72832764	5	170577669		G	A	421	0.003563	3	1.208783888	0.257601337	4.692459687	2.70E-06	18.21597569	3.881967434	intronic	KCNIP1	.
3 month	rs183180157	1	181277985		A	C	421	0.009501	7	1.744207622	0.371856251	4.690542692	2.72E-06	12.61385999	2.689211211	intergenic	LINC01699;CACNA1E	dist=39381;dist=205532
3 month	rs73227413	21	21764653		G	A	421	0.034442	28	3.687754962	0.78647158	4.688986931	2.75E-06	5.962055143	1.271501762	ncRNA_intronic	LINC01425	.
3 month	rs185874707	8	18173165		C	T	421	0.011876	10	2.405517016	0.513034555	4.688801158	2.75E-06	9.139347655	1.949186444	intronic	NAT1	.
3 month	rs423841	8	54643509		G	A	421	0.662708	291	-10.22664529	2.181175145	-4.688594272	2.75E-06	-2.149572575	0.458468456	intronic	RP1	.
3 month	rs191271637	17	54045899	0.762179971	A	G	421	0.003563	3	1.195597009	0.255047807	4.687736869	2.76E-06	18.379836	3.920833552	intergenic	KIF2B;TOM1L1	dist=220706;dist=854792
3 month	rs187978759	7	11672218		G	A	421	0.003563	3	1.07420489	0.229159549	4.687585114	2.76E-06	20.4555522	4.3637719	intronic	THSD7A	.
3 month	rs193153124	3	148612923		A	G	421	0.005938	5	1.529063164	0.32624874	4.686801743	2.78E-06	14.36573132	3.065145937	intergenic	LINC02046;AGTR1	dist=212967;dist=84948
3 month	rs1877768	6	16534692		C	T	421	0.017815	15	2.650393922	0.565512508	4.686711405	2.78E-06	8.287546847	1.768307483	intronic	ATXN1	.
3 month	rs76617932	1	180961288		T	C	421	0.011876	9	2.08220138	0.444364012	4.685801107	2.79E-06	10.54496084	2.250407262	intergenic	KIAA1614-AS1;STX6	dist=6401;dist=11426
3 month	rs188034471	9	83322799		G	A	421	0.004751	4	1.487832555	0.317535808	4.68555835	2.79E-06	14.75599992	3.149251127	intronic	FRMD3	.
3 month	rs1595406	8	54718055		A	G	421	0.345606	291	10.45008066	2.230645943	4.684777831	2.80E-06	2.100188892	0.448300638	intronic	RP1	.
3 month	rs147627638	6	98725240		A	G	421	0.007126	6	1.675781082	0.357955603	4.681533316	2.85E-06	13.07853062	2.793642539	intergenic	MIR2113;PNKY	dist=700621;dist=104901
3 month	rs151272830	2	67455592		G	T	421	0.007126	6	1.643645432	0.351116105	4.681202055	2.85E-06	13.33234787	2.848060758	intergenic	ETAA1;LINC01812	dist=43503;dist=340462
3 month	rs141169929	3	165090674	0.77117002	A	G	421	0.004751	5	1.473704308	0.315071386	4.677366376	2.91E-06	14.84541783	3.173883899	intergenic	SI;SLITRK3	dist=12178;dist=96046
3 month	rs720372	8	54716077		G	A	421	0.346793	292	10.41667706	2.227853301	4.675656629	2.93E-06	2.098727338	0.448862589	intronic	RP1	.
3 month	rs567080482	13	94423151		T	C	421	0.004751	4	1.409440819	0.301549206	4.67399944	2.95E-06	15.49995607	3.316208371	intergenic	GPC6;DCT	dist=15132;dist=13660
3 month	rs546286713	13	90758825		G	A	421	0.004751	4	1.389978902	0.297519732	4.671888118	2.98E-06	15.70278409	3.361121605	intergenic	LINC01049;LINC00410	dist=223484;dist=132129
3 month	rs142311947	5	177957468	0.768029988	G	A	421	0.007126	8	1.813233127	0.388171459	4.671217019	2.99E-06	12.03390127	2.576181159	ncRNA_intronic	LOC100128340	.
3 month	rs185155853	15	40951902	0.723249972	C	T	421	0.004751	5	1.370089738	0.293393972	4.669795111	3.02E-06	15.91646574	3.408386314	intergenic	DLL4;CHAC1	dist=12829;dist=1569
3 month	rs550763536	2	7312216		T	G	421	0.005938	5	1.504113282	0.322217093	4.66801208	3.04E-06	14.48716465	3.103497679	intergenic	LOC101929452;LOC1005062	dist=234336;dist=109045
3 month	rs555040883	22	40235472		G	A	421	0.004751	4	1.412000774	0.302532759	4.667265721	3.05E-06	15.42730692	3.305427169	intronic	TNRC6B	.
3 month	rs2375219	8	54785735		C	T	421	0.393112	330	10.58980707	2.26895928	4.667253025	3.05E-06	2.057001668	0.440730695	intronic	RP1	.
3 month	rs140062526	13	58711899		G	A	421	0.005938	5	1.544633202	0.331067832	4.665609433	3.08E-06	14.09260875	3.020529034	intergenic	LINC00374;DIAPH3	dist=478782;dist=953688
3 month	rs572961122	13	107360637	0.793820024	C	T	421	0.005938	6	1.632302386	0.349861236	4.665570858	3.08E-06	13.33548956	2.858276075	intronic	FAM155A	.
3 month	rs544042801	11	68692775		G	A	421	0.004751	4	1.370120555	0.293669353	4.665521075	3.08E-06	15.88698661	3.405190192	intergenic	GAL;TESMIN	dist=1600;dist=14665
3 month	rs142993106	4	90036221		G	A	421	0.017815	15	2.691736025	0.577013528	4.664944401	3.09E-06	8.084636111	1.733061622	intergenic	MMRN1;CCSER1	dist=81611;dist=91173
3 month	rs569916471	14	75424639	0.771009982	G	A	421	0.004751	5	1.390580812	0.298176949	4.663609368	3.11E-06	15.64040878	3.353713303	intergenic	LINC01220;JDP2	dist=128231;dist=3085
3 month	rs7104959	11	129976231		C	T	421	0.003563	3	1.212990302	0.260168118	4.66233262	3.13E-06	17.92046105	3.843668505	intronic	PRDM10	.
3 month	rs559228693	20	15982684	0.798879981	G	A	421	0.005938	5	1.512274875	0.324468321	4.660778189	3.15E-06	14.36435511	3.081964969	ncRNA_intronic	LOC613266	.
3 month	rs187384541	8	1684075	0.760779977	A	G	421	0.035629	26	3.153891836	0.676696813	4.660716257	3.15E-06	6.887451174	1.477766677	intronic	DLGAP2	.
3 month	rs141127122	22	40208435		G	A	421	0.004751	4	1.411818313	0.302953602	4.660179993	3.16E-06	15.38248751	3.30083549	intronic	TNRC6B	.
3 month	rs193093906	10	125016920		G	A	421	0.009501	9	2.062968829	0.442790343	4.659019469	3.18E-06	10.52195366	2.25840517	intronic	CTBP2	.
3 month	rs77871739	9	135660463		G	A	421	0.004751	4	1.439297375	0.309017583	4.657655271	3.20E-06	15.07246035	3.236061811	intergenic	GLT6D1;LCN9	dist=20923;dist=2859
3 month	rs117816016	8	102739034		C	T	421	0.003563	3	1.090578449	0.234213862	4.656336053	3.22E-06	19.88070227	4.269602116	intergenic	LOC101927245;GASAL1	dist=52311;dist=67788
3 month	rs775626702	5	163203061		A	C	421	0.003563	3	1.143758046	0.245686137	4.655362562	3.23E-06	18.94841366	4.070233717	intergenic	GABRG2;CCNG1	dist=1047522;dist=234510
3 month	rs146048121	6	141877818		G	A	421	0.002375	3	1.082392071	0.232511623	4.655217038	3.24E-06	20.02143793	4.300860255	intergenic	MIR4465;NMBR	dist=1193935;dist=196666
3 month	rs139493286	18	31236056		G	A	421	0.003563	3	1.241116041	0.266612329	4.655133716	3.24E-06	17.46030927	3.750764282	intergenic	DSC1;DSG1	dist=73200;dist=82104
3 month	rs146442492	15	58689916	0.740549982	C	T	421	0.027316	24	3.06908876	0.65936392	4.654620409	3.25E-06	7.059258577	1.516613162	intronic	ADAM10	.
3 month	rs181259864	7	97859511		C	A	421	0.003563	3	1.263697226	0.271559968	4.653473908	3.26E-06	17.13608209	3.682427888	ncRNA_intronic	CZ1P-ASNS	.
3 month	rs150946694	22	46457283		T	C	421	0.004751	4	1.413199859	0.303835874	4.651194872	3.30E-06	15.30824787	3.291250592	intronic	CELSR1	.
3 month	rs181933850	5	92169830		A	G	421	0.008314	7	1.957149723	0.420974257	4.64909597	3.33E-06	11.04365858	2.375442162	intergenic	ARRDC3-AS1;NR2F1-AS1	dist=749115;dist=1279415
3 month	rs113006316	2	74575233	0.706809998	A	G	421	0.002375	3	0.943984037	0.203082246	4.648284397	3.35E-06	22.88867923	4.924113346	intronic	M1AP	.
3 month	rs191006910	5	154836449		G	A	421	0.003563	3	1.226165352	0.263817655	4.647775955	3.36E-06	17.61738031	3.790496891	intronic	FAXDC2	.
3 month	rs176786	14	45813767		T	C	421	0.32304	268	9.449422953	2.033680348	4.64646421	3.38E-06	2.284756409	0.49171936	intergenic	LINC02303;LINC00871	dist=98165;dist=250392
3 month	rs187047882	3	164567833		G	A	421	0.003563	3	1.161853087	0.250171732	4.644222095	3.41E-06	18.56413613	3.997254169	intergenic	MIR1263;LINC01324	dist=396277;dist=146262
3 month	rs73057656	3	33899079	0.799390018	A	G	421	0.036817	33	3.638911487	0.783762258	4.642876649	3.44E-06	5.923832897	1.27589711	intergenic	PDCD6IP;LOC101928135	dist=29372;dist=976718
3 month	rs112557251	2	187510673	0.726930022	T	C	421	0.002375	3	1.043551353	0.224794821	4.642239298	3.45E-06	20.65100643	4.448501059	intronic	TFPI	.
3 month	rs190190051	5	92179668		G	A	421	0.008314	7	1.947160785	0.419607459	4.640434158	3.48E-06	11.05898873	2.383179753	intergenic	ARRDC3-AS1;NR2F1-AS1	dist=758953;dist=1269577
3 month	rs180989936	1	193075048		A	G	421	0.004751	4	1.253423246	0.270112952	4.640367065	3.48E-06	17.17935787	3.702154942	intronic	RO60	.
3 month	rs188415494	8	25755782	0.735019982	C	T	421	0.003563	3	1.162251387	0.250533229	4.639110717	3.50E-06	18.51694779	3.991486498	intergenic	CDCA2;EBF2	dist=247865;dist=85943
3 month	rs1686289	14	45791779		G	A	421	0.678147	277	-9.537609426	2.056514006	-4.637755637	3.52E-06	-2.255153927	0.486259757	intergenic	LINC02303;LINC00871	dist=76177;dist=272380
3 month	rs76904423	12	100794966		G	A	421	0.010689	10	2.190673587	0.472659243	4.634784191	3.57E-06	9.805762313	2.115689082	UTR5	ANO4	NM_001286615:c.-1068200>0
3 month	rs183466664	12	26668754		A	G	421	0.004751	4	1.341044738	0.289344304	4.634771518	3.57E-06	16.01818822	3.456090156	intronic	ITPR2	.
3 month	rs999769259	4	61646247	0.702899992	G	A	421	0.003563	3	0.969956079	0.209381164	4.632489677	3.61E-06	22.12467252	4.775978806	intronic	ADGRL3	.
3 month	rs553840536	16	25686574		A	G	421	0.003563	3	1.093708141	0.236100775	4.632378455	3.61E-06	19.62034417	4.23547954	intergenic	ZKSCAN2;HS3ST4	dist=428729;dist=5385
3 month	rs183962155	4	21258020		A	C	421	0.005938	6	1.557852883	0.336419694	4.630682783	3.64E-06	13.76460079	2.972477589	intronic	KCNIP4	.

3 month	rs138480898	1	184986525		C	T	421	0.003563	3	1.261421145	0.272500077	4.629067106	3.67E-06	16.9873974	3.669723728	intergenic	NIBAN1;LINC01633	dist=12017;dist=15002
3 month	rs185158855	2	222785307	0.73951	C	A	421	0.004751	4	1.284200145	0.277572279	4.626543222	3.72E-06	16.66788644	3.602665239	intergenic	MOGAT1;ACSL3	dist=75377;dist=75728
3 month	rs180926150	1	102760770	0.792620003	C	T	421	0.002375	3	1.088498575	0.235408416	4.62387281	3.77E-06	19.64191801	4.247936485	intergenic	OLFM3;COL11A1	dist=763536;dist=115697
3 month	rs146728064	17	19362127		G	A	421	0.007126	6	1.69020067	0.365674681	4.622143005	3.80E-06	12.64004111	2.734671147	intronic	B9D1	.
3 month	rs111900874	7	89887355		G	A	421	0.014252	12	2.536039096	0.548671755	4.622142612	3.80E-06	8.424240128	1.822583342	ncRNA_intronic	STEAP2-AS1	.
3 month	rs574076561	7	49505151		A	G	421	0.003563	3	1.086373837	0.235169216	4.619541003	3.85E-06	19.64347663	4.25225723	intergenic	CDC14C;VWC2	dist=577697;dist=268487
3 month	rs76554191	2	95301880		G	A	421	0.04038	34	3.727552	0.807156314	4.618129021	3.87E-06	5.721480386	1.238917397	intronic	KCNIP3	.
3 month	rs176783	14	45811710		A	G	421	0.321853	267	9.35224324	2.025260115	4.617798558	3.88E-06	2.280101466	0.493763736	intergenic	LINC02303;LINC00871	dist=96108;dist=252449
3 month	rs140352232	2	107421656	0.797200024	G	A	421	0.002375	3	1.01264036	0.219291932	4.617772993	3.88E-06	21.05765113	4.560131294	intergenic	MIR548AU;LINC01886	dist=72131;dist=107763
3 month	rs139062456	8	13394482		C	T	421	0.017815	15	2.721124769	0.589318868	4.617406495	3.89E-06	7.835158086	1.696874229	intronic	DLC1	.
3 month	rs558614420	15	41518672	0.745689988	C	T	421	0.005938	6	1.524477016	0.330280733	4.615700711	3.92E-06	13.97508315	3.02772732	intronic	RPAP1	.
3 month	rs12266995	10	24563854		T	C	421	0.030879	26	3.568935699	0.773323681	4.615060663	3.93E-06	5.967825345	1.293119589	intergenic	KIAA1217;ARHGAP21	dist=16006;dist=19760
3 month	rs192750513	7	97948518		A	G	421	0.003563	3	1.238714279	0.26844613	4.614386809	3.94E-06	17.18924692	3.725142178	ncRNA_intronic	CZ1P-ASNS	.
3 month	rs56302696	12	47899047	0.778989971	G	A	421	0.002375	3	1.068148373	0.231704225	4.60996503	4.03E-06	19.89590409	4.315847075	intronic	VDR	.
3 month	rs151015676	5	177963936	0.785820007	T	G	421	0.002375	3	1.054373875	0.228793957	4.608399149	4.06E-06	20.14213671	4.370744819	intergenic	LOC100128340;PROP1	dist=4136;dist=28299
3 month	rs12315614	12	64527177		C	A	421	0.07601	64	5.614667617	1.219220913	4.605127386	4.12E-06	3.777106623	0.820195905	intergenic	TBK1;RASSF3	dist=25064;dist=83318
3 month	rs72837643	5	170761951	0.782980025	T	C	421	0.003563	4	1.251913633	0.271928683	4.60383075	4.15E-06	16.93028738	3.677434793	intergenic	KCNIP1;GABRP	dist=25319;dist=21768
3 month	rs428110	14	45825457		A	C	421	0.317102	265	9.275749143	2.014875505	4.603633881	4.15E-06	2.284822992	0.49630858	intergenic	LINC02303;LINC00871	dist=109855;dist=238702
3 month	rs111391231	7	89867731		T	C	421	0.014252	12	2.546596314	0.553340001	4.602227036	4.18E-06	8.317177547	1.807207138	intergenic	ZNF804B;STEAP2-AS1	dist=529203;dist=14622
3 month	rs180765647	13	113724338		G	T	421	0.002375	3	1.05190176	0.228624832	4.600995217	4.20E-06	20.12465213	4.373978059	intronic	GRK1	.
3 month	rs568658857	12	49460215		G	A	421	0.005938	5	1.486911933	0.32318174	4.600853791	4.21E-06	14.23611926	3.094234224	intronic	SPATS2	.
3 month	rs182531466	5	92234256		C	A	421	0.007126	6	1.668434681	0.362732486	4.599628498	4.23E-06	12.68049781	2.756852606	intergenic	ARRDC3-AS1;NR2F1-AS1	dist=813541;dist=1214989
3 month	rs181812512	11	66898258		C	T	421	0.003563	3	1.08407707	0.235726848	4.598869755	4.25E-06	19.50931683	4.242198164	intronic	PC	.
3 month	rs556680896	13	100950161		C	T	421	0.003563	3	1.24171677	0.270060937	4.597913279	4.27E-06	17.0254659	3.702867989	ncRNA_intronic	NALCN-AS1	.
3 month	rs142928734	13	100948828		G	A	421	0.003563	3	1.242180957	0.270237412	4.59662838	4.29E-06	17.00959296	3.700449886	ncRNA_intronic	NALCN-AS1	.
3 month	rs143287889	4	35570658	0.79956001	C	T	421	0.002375	3	1.058108262	0.230199399	4.596485777	4.30E-06	19.96741	4.34406	intergenic	LINC02484;ARAP2	dist=1300911;dist=495346
3 month	rs543844012	9	30107025		C	T	421	0.003563	3	1.164274175	0.253510467	4.592607905	4.38E-06	18.11604845	3.944610301	intergenic	LINGO2;LINC01242	dist=893424;dist=281910
3 month	rs566018180	10	84995296	0.773450017	C	T	421	0.003563	3	1.136146605	0.247392568	4.592484792	4.38E-06	18.56355199	4.04215862	intergenic	CCSER2;LINC01519	dist=476775;dist=198125
3 month	rs147171192	4	88214436		A	G	421	0.007126	5	1.584612591	0.345114993	4.591549547	4.40E-06	13.30440472	2.89758492	intronic	ABCG2	.
3 month	rs2876414	20	15833059		G	T	421	0.022565	19	3.090964158	0.673260548	4.591037107	4.41E-06	6.819109065	1.485309072	intronic	MACROD2	.
3 month	rs137873790	5	97751337		A	G	421	0.013064	11	2.280553097	0.497276469	4.586086892	4.52E-06	9.222408811	2.010953789	intergenic	LINC01340;LINC02234	dist=80286;dist=89421
3 month	rs752259256	10	103165562	0.747950017	T	C	421	0.003563	3	1.029573618	0.224511445	4.58584023	4.52E-06	20.42586391	4.454115907	intronic	NT5C2	.
3 month	rs185771987	5	73989664	0.738900006	T	C	421	0.003563	3	1.153019969	0.251461612	4.585272329	4.53E-06	18.23448239	3.976750144	intergenic	ARHGEF28;LINC01335	dist=47671;dist=316746
3 month	rs557092705	20	35601989		C	T	421	0.003563	3	1.079320791	0.235404492	4.584962596	4.54E-06	19.47695457	4.248007298	ncRNA_exonic	FER1L4	.
3 month	rs140642138	15	41832967		G	A	421	0.005938	6	1.547446255	0.337525343	4.584681679	4.55E-06	13.58322205	2.962740492	intronic	JMJD7;JMJD7-PLA2G4B	.
3 month	rs139877408	2	128848699	0.79351002	A	G	421	0.002375	3	1.034497038	0.225706887	4.583364964	4.58E-06	20.30671294	4.430524974	intergenic	HS6ST1;LOC101927881	dist=529831;dist=15901
3 month	rs2327968	20	15832846		C	T	421	0.024941	21	3.410130247	0.744098505	4.582901624	4.59E-06	6.158998564	1.343908089	intronic	MACROD2	.
3 month	rs191986449	5	25745578		C	T	421	0.004751	3	1.135135845	0.247721071	4.582314459	4.60E-06	18.49787925	4.036798308	intergenic	LINC02211;CDH9	dist=443298;dist=1135019
3 month	rs11690187	2	67338777		A	C	421	0.005938	5	1.539986371	0.336119518	4.581663041	4.61E-06	13.63105325	2.975132201	intergenic	LINC01828;ETAA1	dist=49533;dist=58556
3 month	rs118040657	10	3430654	0.777450025	C	T	421	0.008314	8	1.732520372	0.378165204	4.581384943	4.62E-06	12.11477125	2.644346939	ncRNA_intronic	LOC105376360	.
3 month	rs138109686	15	41759244		A	G	421	0.005938	6	1.543778108	0.336970687	4.581342434	4.62E-06	13.59567051	2.96761718	intronic	MGA	.
3 month	rs563167766	1	102400809		G	A	421	0.002375	3	1.07877255	0.235715413	1.07877255	4.73E-06	19.41573898	4.242403955	intergenic	OLFM3;COL11A1	dist=403575;dist=475658
3 month	rs545550279	8	114540200		G	T	421	0.003563	3	1.091458604	0.238542228	4.57553622	4.75E-06	19.18124208	4.192129875	intergenic	CSMD3;TRPS1	dist=1103261;dist=868295
3 month	rs1498183	8	54804345		C	T	421	0.394299	332	10.33728121	2.259624491	4.574778355	4.77E-06	2.024574602	0.442551408	intronic	RP1	.
3 month	rs375790303	1	184561348		G	A	421	0.008314	7	1.831959409	0.400449797	4.574754241	4.77E-06	11.42403934	2.497191924	intronic	C1orf21	.
3 month	rs1396896	8	54782750		A	G	421	0.397862	334	10.46979793	2.288829589	4.574302074	4.78E-06	1.998533266	0.436904523	intronic	RP1	.
3 month	rs7843693	8	54779552		G	A	421	0.397862	334	10.46760966	2.288570748	4.573863258	4.79E-06	1.998567561	0.436953938	intronic	RP1	.
3 month	rs1391462	8	54787221		C	A	421	0.397862	334	10.46683006	2.288537601	4.573588854	4.79E-06	1.998476605	0.436960267	intronic	RP1	.
3 month	rs150539922	21	41856807		T	C	421	0.003563	3	1.254834519	0.274365448	4.57358799	4.79E-06	16.66969372	3.64477381	intronic	PRDM15	.
3 month	rs145896760	15	41827024		G	A	421	0.005938	6	1.564221999	0.342059488	4.572953105	4.81E-06	13.36888249	2.923468092	UTR3	MAPKBP1	NM_014994:c.*15880>0;NM_
3 month	rs181415102	4	111768110	0.716539979	T	C	421	0.003563	4	1.144945045	0.250383121	4.572772469	4.81E-06	18.26310193	3.993879436	intergenic	MIR297;FAM241A	dist=907463;dist=377344
3 month	rs6080	15	58545734	0.79065001	C	A	421	0.043943	36	3.688174426	0.80689065	4.570847892	4.86E-06	5.664767454	1.239325304	intronic	LIPC	.
3 month	rs138249376	10	61756071		T	G	421	0.004751	4	1.271516185	0.278189815	4.57067843	4.86E-06	16.43007109	3.594667912	intronic	CABCOCO1	.
3 month	rs529345909	11	67343381		A	G	421	0.003563	3	1.116845445	0.244375672	4.570198967	4.87E-06	18.70153001	4.092060355	ncRNA_intronic	LOC100130987	.
3 month	rs191930622	12	47890872	0.790120006	G	A	421	0.002375	3	1.061255347	0.232228501	4.569875551	4.88E-06	19.67835791	4.306103677	intronic	VDR	.
3 month	rs145116559	4	111756440	0.70095998	T	C	421	0.003563	4	1.144113716	0.250422362	4.568736218	4.91E-06	18.24412236	3.993253602	intergenic	MIR297;FAM241A	dist=895793;dist=389014
3 month	rs12678939	8	54792461		A	G	421	0.394299	331	10.32905196	2.261417548	4.567512076	4.94E-06	2.019756183	0.442200513	intronic	RP1	.
3 month	rs73586304	6	142518288		C	T	421	0.003563	3	1.14329832	0.250357885	4.566655922	4.96E-06	18.24051164	3.99428202	intergenic	ADGRG6;LOC153910	dist=72022;dist=8167
3 month	rs113767990	14	81251219		G	A	421	0.004751	4	1.429622227	0.313123218	4.565685793	4.98E-06	14.58111547	3.193630952	intergenic	LOC101928504;STON2	dist=27839;dist=9431

Supplementary Table S1. GWAS Results SNPs Indianapolis-1 Discovery Cohort
Overlap of 12 month and 3 month quantitative traits (QT)

Headers

QT: quantitiative trait; rsid: reference SNP cluster ID, chr: chromosome; pos_38, position of SNP on GRCh38 reference panel; REF and ALT, reference allele and alternate allele;
caf.12m: common (major) allele frequency, 12 month QT; Score.12m: p-values from Score test, 12 month QT; Score.SE.12m; Score.pval.12m
caf.3m: common (major) allele frequency, 3 month QT; Score.3m: p-values from Score test, 3 month QT; Score.SE.3m; Score.pval.3m

rsid	chr	pos_38	REF	ALT		caf.12m	Score.12m	Score.SE.12m	Score.pval.12m		caf.3m	Score.3m	Score.SE.3m	Score.pval.3m
rs76098744	1	111806750	C	T		0.01594533	2.284999945	0.488674498	2.93E-06		0.016627078	2.811615503	0.579024995	1.20E-06
rs74683551	1	111811796	G	A		0.01594533	2.279782265	0.488661396	3.08E-06		0.016627078	2.810351968	0.579019309	1.21E-06
rs536781978	2	29510815	A	G		0.003416856	1.090050785	0.230277801	2.21E-06		0.003562945	1.282866581	0.270080884	2.03E-06
rs568321148	2	29647482	T	G		0.003416856	1.087769414	0.22901495	2.04E-06		0.003562945	1.279867135	0.268808164	1.92E-06
rs148248743	3	136415753	C	T		0.002277904	0.909706546	0.183031941	6.69E-07		0.002375297	1.069833829	0.216429281	7.69E-07
rs189709453	3	177856520	G	A		0.003416856	0.994705176	0.207058897	1.56E-06		0.003562945	1.314463975	0.246356303	9.52E-08
rs186767531	3	177921989	T	C		0.003416856	0.994608422	0.207613329	1.66E-06		0.003562945	1.293967205	0.247047316	1.63E-07
rs182868205	3	177994660	C	T		0.003416856	1.020592047	0.21132014	1.37E-06		0.003562945	1.323927501	0.251356167	1.39E-07
rs113063005	4	23507444	T	C		0.006833713	1.445039344	0.294886773	9.57E-07		0.005938242	2.130262689	0.316937024	1.80E-11
rs189912648	5	135429749	C	T		0.003416856	1.088415104	0.234100744	3.33E-06		0.003562945	1.303529678	0.277126356	2.55E-06
rs190251199	14	105124240	T	C		0.003416856	0.998973364	0.210064697	1.98E-06		0.003562945	1.240668633	0.248987137	6.27E-07
