# Supplementary material for: Pharmacogenomics of steroid-induced ocular hypertension: relationship to high-tension glaucomas and new pathophysiologic insight
Source: medRxiv. 2025 Aug 13:2025.08.11.25333245. Preprint. [Version 1] doi: 10.1101/2025.08.11.25333245 (PMC12363710; doi:10.1101/2025.08.11.25333245)
Supplement: Supplement 3 — Table S2. GWAS Results Risk Loci Indianapolis-1 Discovery Cohort [file media-3.pdf]

Supplementary Table S2. GWAS Results Risk Loci Indianapolis-1 Discovery Cohort
Merged loci count and quantitative trait (QT) overlap

Notes
Top SNPs identified with the 12M and 3M QTs are merged, then clustered into risk loci by sorting by chromosomal position, then by chromosome
SNPs duplicated between 12 month and 3 month QT are boxed. Loci that cluster SNPs from both the 12 and 3 month QTs are boxed and highlighted in blue.
SNPs of genome-wide significance are highlighted in gray (column rsid) as are their p-values (column Score.pval).
Risk loci into which multiple SNPs cluster are boxed (column Gene.refGene) and those containing SNPs of genome-wide significance are shaded in gray
Count risk loci: columns adding up all risk loci broken down by p-value and QT, totals at bottom; 12+3 rep: risk loci clustering both 12M+3M QT SNPs that are duplicates;
12+3 diff: risk loci clustering both 12M+3M QT SNPs for which the 2 QTs identify different SNPs
Headers
QT: quantitative trait; rsid: reference SNP cluster ID, chr: chromosome; pos_38: position of SNP on GRCh38 reference panel; Score.pval: p-value; Est: effect size (mm Hg);
Func.refGene: SNP location with respect to nearest gene(s); Gene.refGene: nearest gene upstream and downstream; GeneDetail.refGene: distance to nearest gene upstream and downstream

										Count risk loci					
QT	rsid	chr	pos_38	MAC	Score.pval	Est	Func.refGene	Gene.refGene	GeneDetail.refGene	5E-08 12M	5E-08 3M	5E-06 12M	5E-06 3M	12+3 rep	12+3 diff
3M	rs115348382	1	9595845	3	2.20695702237544e-06	18.2107495	intronic	TMEM201	.				1		
3M	rs186532456	1	18621834	3	9.60875608166652e-07	20.1730343	intergenic	KLHDC7A;PAX7	dist=135848;dist=9012				1		
3M	rs562032622	1	18632839	3	1.12975575994446e-06	21.0267115	intronic	PAX7	.						
12M	rs35145334	1	23138629	14	2.09882278975154e-06	10.6552656	intronic	LUZP1	.			1			
3M	rs2365739	1	62018790	18	1.81260643542989e-06	6.86815917	intronic	PATJ	.				1		
12M	rs17127656	1	65477788	49	1.63996682822861e-06	5.07046538	intronic	LEPR	.			1			
12M	rs7518849	1	65483108	49	1.67668772877101e-06	5.07456956	intronic	LEPR	.						
12M	rs11579567	1	65491458	49	2.1300143111827e-06	5.02973793	intronic	LEPR	.						
12M	rs7534177	1	65500037	49	2.85450525546462e-06	4.96123378	intronic	LEPR	.						
3M	rs149493615	1	79414404	8	1.38822331670427e-06	11.750632	intergenic	ADGRL4;LINC01781	dist=407674;dist=1121351				1		
3M	rs143811231	1	79502446	8	1.738654939087e-06	11.5747409	intergenic	ADGRL4;LINC01781	dist=495716;dist=1033309						
12M	rs139390630	1	84400513	13	2.86874207705929e-06	11.6975971	intronic	DNASE2B	.			1			
12M	rs114067899	1	84725361	7	2.17218533160816e-06	13.7760673	intergenic	SSX2IP;LPAR3	dist=34893;dist=86241			1			
3M	rs34270375	1	88905019	21	9.34314238699532e-07	7.71147017	intergenic	GTF2B;KYAT3	dist=13452;dist=30754				1		
3M	rs187518659	1	98990189	8	2.15952482477188e-06	11.6819623	intronic	PLPPR5	.				1		
3M	rs140420703	1	102337928	5	1.91423564672595e-06	16.487654	intergenic	OLFM3;COL11A1	dist=340694;dist=538539						
3M	rs563167766	1	102400809	3	4.72618849175033e-06	19.415739	intergenic	OLFM3;COL11A1	dist=403575;dist=475658						
3M	rs77180278	1	102496326	24	9.33877360643494e-08	7.33619996	intergenic	OLFM3;COL11A1	dist=499092;dist=380141						
3M	rs112351653	1	102754804	24	2.54962936189828e-08	7.42386947	intergenic	OLFM3;COL11A1	dist=757570;dist=121663		1				
3M	rs180926150	1	102760770	3	3.76640865110522e-06	19.641918	intergenic	OLFM3;COL11A1	dist=763536;dist=115697						
3M	rs114413507	1	102953612	24	2.57350301189632e-08	7.42216029	intronic	COL11A1	.						
3M	rs116672066	1	103007360	23	4.45932234807969e-09	8.04769476	intronic	COL11A1	.						
3M	rs111928960	1	103168079	22	3.65396910662882e-09	8.60257208	intergenic	COL11A1;LOC101928436	dist=59557;dist=325967						
3M	rs113221952	1	103288418	19	9.173975220783e-07	7.92350704	intergenic	COL11A1;LOC101928436	dist=179896;dist=205628						
3M	rs1856085	1	103571923	3	4.63628412408432e-07	21.51144	intronic	AMY2B	.				1		
3M	rs143597860	1	103614521	3	4.52300265269257e-07	21.5748154	intergenic	AMY2B;AMY2A	dist=34987;dist=2811						
3M	rs144541665	1	103768107	3	3.71844012409788e-07	21.8734611	intergenic	AMY1C;LOC100129138	dist=9415;dist=304916						
3M	rs76098744	1	111806750	14	1.19916376071606e-06	8.38612452	intronic	KCND3	.				1	1	
12M	rs76098744	1	111806750	14	2.92647184366499E-06	9.5685657	intronic	KCND3	.						
3M	rs74683551	1	111811796	14	1.2121497054257e-06	8.38252043	intronic	KCND3	.						
12M	rs74683551	1	111811796	14	3.08074322019196E-06	9.54722832	intronic	KCND3	.						
3M	rs76617932	1	180961288	9	2.78866978177701e-06	10.5449608	intergenic	KIAA1614;STX6	dist=6401;dist=11426				1		
3M	rs183180157	1	181277985	7	2.7248136592675E-06	12.61386	intergenic	LINC01699;CACNA1E	dist=39381;dist=205532						
3M	rs375790303	1	184561348	7	4.76779314562933e-06	11.4240393	intronic	C1orf21	.				1		
3M	rs138480898	1	184986525	3	3.67316732025395e-06	16.9873974	intergenic	NIBAN1;LINC01633	dist=12017;dist=15002						

3M	rs145766563	1	185160370	4	1.9043145410504e-06	16.8682102	intronic	SWT1	.					
3M	rs147032554	1	186179732	3	6.67557909700165e-07	18.162609	intronic	HMCN1	.					
3M	rs180989936	1	193075048	4	3.47790847768381E-06	17.1793579	intronic	RO60	.			1		
12M	rs145804766	1	202153371	9	1.43417032447652e-06	12.5864465	intronic	PTPN7	.		1			
3M	rs10494861	1	205362746	3	2.48126631931009e-06	19.1820498	intergenic	KLHDC8A;LEMD1	dist=5656;dist=10506			1		
12M	rs147869916	1	227119951	5	3.36060694276516e-06	17.5964551	intronic	CDC42BPA	.		1			
3M	rs2274996	1	229668791	35	6.73601821695854e-07	5.58527433	intergenic	URB2;LINC01682	dist=8591;dist=206759			1		
3M	rs2274997	1	229668899	35	6.64244186267036e-07	5.58731228	intergenic	URB2;LINC01682	dist=8699;dist=206651					
3M	rs2891865	1	229670621	35	6.80112526815562e-07	5.58181376	intergenic	URB2;LINC01682	dist=10421;dist=204929					
3M	rs2385790	1	229671745	35	6.81207031237396e-07	5.58224822	intergenic	URB2;LINC01682	dist=11545;dist=203805					
3M	rs12024557	1	229676610	35	6.90833131537333e-07	5.56930969	intergenic	URB2;LINC01682	dist=16410;dist=198940					
3M	rs4562666	1	229689023	36	7.35045143592774e-07	5.51922599	intergenic	URB2;LINC01682	dist=28823;dist=186527					
3M	rs12036586	1	229690631	40	1.08649183321501e-06	5.22212176	intergenic	URB2;LINC01682	dist=30431;dist=184919					
3M	rs16850124	1	229695584	37	2.19845550423288e-06	5.18095662	intergenic	URB2;LINC01682	dist=35384;dist=179966					
3M	rs12045643	1	229698303	36	7.37332600021845e-07	5.48149152	intergenic	URB2;LINC01682	dist=38103;dist=177247					
3M	rs559559983	1	246813830	5	1.8701710647004e-06	13.811132	intergenic	LINC01341;AHCTF1	dist=22344;dist=25268			1		
3M	rs550763536	2	7312216	5	3.04128022440287e-06	14.4871647	intergenic	LOC101929452;LOC100506274	dist=234336;dist=109045			1		
12M	rs76220567	2	12919819	4	2.07255893928856e-06	20.6828868	intergenic	TRIB2;LOC100506474	dist=177087;dist=46963		1			
3M	rs558553658	2	15542600	3	2.00500271573415e-07	17.1099107	intronic	NBAS	.			1		
3M	rs536781978	2	29510815	3	2.20535447064362e-06	20.5561882	intronic	ALK	.			1	1	
12M	rs536781978	2	29510815	3	2.20535447064362E-06	20.5561882	intronic	ALK						
3M	rs568321148	2	29647482	3	2.03643401922156e-06	20.7400203	intronic	ALK	.					
12M	rs568321148	2	29647482	3	2.03643401922156E-06	20.7400203	intronic	ALK						
12M	rs186792608	2	42869559	3	1.57557414570447e-07	22.4512499	intergenic	HAAO;LINC01819	dist=76976;dist=158293		1			
3M	rs76777840	2	48085811	5	7.02623491851785e-07	14.3500784	intergenic	FBXO11;FOXN2	dist=179313;dist=228488			1		
3M	rs145080832	2	48246004	4	1.37120287877638e-07	17.1618477	intergenic	FBXO11;FOXN2	dist=339506;dist=68295					
3M	rs184220112	2	48404604	4	3.11727344916156e-07	16.4879043	intergenic	FOXN2;PPP1R21	dist=25309;dist=36162					
3M	rs189890455	2	48428258	5	4.1971806049668e-07	15.1467391	intergenic	FOXN2;PPP1R21	dist=48963;dist=12508					
3M	rs181193202	2	52300216	3	1.62222587830176e-07	19.6312088	ncRNA_intronic	LOC730100	.	1				
3M	rs183378658	2	52336233	3	1.16662586966659e-07	19.7446169	ncRNA_intronic	LOC730100	.					
3M	rs190193113	2	52858640	3	1.13301212531008e-07	19.9533602	intergenic	MIR4431;ASB3	dist=156025;dist=811339					
3M	rs187520610	2	53132903	4	3.93234514941546e-09	18.8010396	intergenic	MIR4431;ASB3	dist=430288;dist=537076					
3M	rs149421869	2	53256291	8	2.05046156638639e-06	11.7382186	intergenic	MIR4431;ASB3	dist=553676;dist=413688					
3M	rs146479102	2	65598625	5	2.18341304839895e-06	15.4846768	intergenic	SPRED2;MIR4778	dist=166026;dist=759622			1		
3M	rs528288879	2	65648796	6	5.421960882917e-07	14.7878085	intergenic	SPRED2;MIR4778	dist=216197;dist=709451					
3M	rs11690187	2	67338777	5	4.61292753502872e-06	13.6310532	intergenic	LINC01828;ETAA1	dist=49533;dist=58556		1	1		1
3M	rs151272830	2	67455592	6	2.85197668301622e-06	13.3323479	intergenic	ETAA1;LINC01812	dist=43503;dist=340462					
3M	rs186142189	2	67475575	6	2.69645566407293e-06	12.7361927	intergenic	ETAA1;LINC01812	dist=63486;dist=320479					
12M	rs2902021	2	67598553	71	3.30567771883221e-06	4.55991411	intergenic	ETAA1;LINC01812	dist=186464;dist=197501					
12M	rs75082290	2	67604021	69	1.699715195267e-06	4.69395387	intergenic	ETAA1;LINC01812	dist=191932;dist=192033					
12M	rs191298981	2	67882914	3	7.69651662192921e-07	24.0078714	intergenic	LINC01812;C1D	dist=57352;dist=158216		1			
12M	rs113537164	2	68007798	4	1.017020860936e-06	20.3890413	intergenic	LINC01812;C1D	dist=182236;dist=33332					
12M	rs113154814	2	68062365	5	1.66384008676077e-07	19.8310188	intronic	C1D	.					
3M	rs184200893	2	69033781	3	2.24020739771759E-06	19.9580356	intronic	ANTXR1	.			1		
3M	rs111927235	2	74256827	4	8.4250114725101e-07	17.0468955	intronic	SLC4A5	.			1		
3M	rs111838310	2	74446364	5	3.26261179053175e-07	17.7963938	intergenic	RTKN;INO80B-WBP1	dist=4427;dist=8659			1		
3M	rs112983626	2	74470023	4	3.34719559082237e-07	17.7848079	intergenic	MOGS;MRPL53	dist=4641;dist=1959			1		
3M	rs113006316	2	74575233	3	3.34707225210704e-06	22.8886792	intronic	M1AP	.			1		
3M	rs17746486	2	95056864	29	1.47154878644706e-06	6.90851358	intergenic	MAL;MRPS5	dist=2872;dist=28507			1		
3M	rs76554191	2	95301880	34	3.87215481208204e-06	5.72148039	intronic	KCNIP3	.			1		
3M	rs140352232	2	107421656	3	3.8788022972212e-06	21.0576511	intergenic	MIR548AU;LINC01886	dist=72131;dist=107763			1		
12M	rs146919974	2	114495286	5	7.91246832646777e-07	19.7280466	intronic	DPP10	.		1			
3M	rs116189766	2	125636287	3	1.17473937061964e-06	21.190764	intergenic	CNTNAP5;LINC01941	dist=715069;dist=473813			1		

3M	rs139877408	2	128848699	3	4.57552408670287e-06	20.3067129	intergenic	HS6ST1;LOC101927881	dist=529831;dist=15901			1		
3M	rs142894171	2	150581757	3	1.40524666465064e-06	18.0710394	intergenic	LINC01920;LINC02612	dist=9536;dist=47140			1		
3M	rs541508507	2	169166755	4	2.32254126928461e-06	15.4630033	intronic	LRP2	.			1		
3M	rs142549310	2	169173996	4	2.69758962759472e-06	15.2501713	exonic	LRP2	.					
3M	rs556293455	2	176085692	3	1.27371002978549e-06	14.3860463	intergenic	EVX2;HOXD13	dist=1730;dist=7029			1		
3M	rs184098071	2	176251692	3	1.15315934031841e-06	13.947724	intergenic	HOXD1;MTX2	dist=60785;dist=17750		1	1		1
3M	rs79935606	2	176473907	5	2.55448017015286e-06	16.3396833	intergenic	MTX2;MIR1246	dist=135882;dist=127073					
12M	rs532416695	2	176622062	3	1.32803586650991e-07	19.8726656	intergenic	MIR1246;LINC01116	dist=21010;dist=7519					
3M	rs112557251	2	187510673	3	3.44653364518888e-06	20.6510064	intronic	TFPI	.			1		
3M	rs185158855	2	222785307	4	3.71819301386344e-06	16.6678864	intergenic	MOGAT1;ACSL3	dist=75377;dist=75728			1		
3M	rs185510569	2	222950143	3	5.51379458113093e-08	19.5886906	intergenic	ACSL3;KCNE4	dist=5505;dist=102047			1		
3M	rs147559909	2	236142879	5	1.75642429015382e-08	17.0840975	intergenic	AGAP1;GBX2	dist=11086;dist=22356	1				
3M	rs181217257	2	239067423	4	5.29269845950619e-10	21.5677368	intronic	HDAC4	.	1				
3M	rs188076929	2	239072023	4	1.0296847211524e-09	19.9775614	intronic	HDAC4	.					
3M	rs112475378	3	1584977	14	1.34664824685029e-06	9.55085929	intergenic	CNTN6;CNTN4	dist=180760;dist=513826			1		
3M	rs145676540	3	2002951	5	1.25427052978558e-06	13.980658	intergenic	CNTN6;CNTN4	dist=598734;dist=95852					
12M	rs144788248	3	19545253	10	3.87625815195203e-06	11.9086237	intergenic	KCNH8;EFHB	dist=9611;dist=334221		1			
3M	rs146007933	3	28185932	18	1.34950762676418e-06	7.51149453	intergenic	LINC01980;CMC1	dist=325607;dist=55687			1		
3M	rs73057656	3	33899079	33	3.43591498795888e-06	5.9238329	intergenic	PDCD6IP;LOC101928135	dist=29372;dist=976718			1		
3M	rs73085348	3	42669729	11	6.28155549798072e-07	10.139317	intergenic	ZBTB47;KLHL40	dist=2149;dist=15808			1		
3M	rs142684595	3	55285273	5	7.16787983460343e-08	16.7729431	intergenic	LINC02017;WNT5A	dist=96059;dist=180442			1		
12M	rs149706477	3	66162553	3	2.87948941747911e-06	20.9828687	intronic	SLC25A26	.		1			
3M	rs80203220	3	123003484	6	2.31429533714517e-06	13.426042	intronic	SEMA5B	.			1		
12M	rs114280794	3	133123359	14	3.81823609520308e-06	8.76296877	intronic	TMEM108	.			1		
3M	rs192443987	3	135813503	4	1.3640761999881e-06	17.7540285	intergenic	EPHB1;PPP2R3A	dist=553038;dist=152225			1		
3M	rs148248743	3	136415753	3	7.68859185089755e-07	22.839379	intronic	STAG1	.		1	1	1	
12M	rs148248743	3	136415753	3	6.68816287673531E-07	27.1548587	intronic	STAG1	.					
3M	rs576124203	3	142122354	3	4.46547319819514e-07	19.4039925	intronic	TFDP2	.			1		
3M	rs545552231	3	142145508	3	4.79811814113124e-07	19.8641332	intronic	TFDP2	.					
12M	rs138138661	3	147940027	4	5.4928185119564e-08	23.1347457	intergenic	LOC440982;LINC02032	dist=430117;dist=138132		1			
12M	rs189234695	3	147940371	3	3.47705303907978e-06	23.9697134	intergenic	LOC440982;LINC02032	dist=430461;dist=137788					
12M	rs148997617	3	148037789	4	1.05449927842458e-07	20.8774277	intergenic	LOC440982;LINC02032	dist=527879;dist=40370		1			
3M	rs193153124	3	148612923	5	2.77507554510464e-06	14.3657313	intergenic	LINC02046;AGTR1	dist=212967;dist=84948			1		
3M	rs188720948	3	150352093	3	8.59439366670513e-07	17.7032653	intergenic	LINC01214;TSC22D2	dist=28346;dist=56205			1		
3M	rs16823323	3	153939413	14	3.35704338797875e-08	9.48464065	intergenic	LINC02006;ARHGEF26	dist=176887;dist=84988	1				
3M	rs139943877	3	155733500	7	2.25796218142194e-06	13.0535902	intronic	PLCH1	.			1		
12M	rs148483098	3	157664262	5	3.27691859100501e-06	15.7357844	intergenic	SLC66A1L;SHOX2	dist=63168;dist=431643		1			
12M	rs79252854	3	157805368	5	2.4780397246083e-06	17.5464336	intergenic	SLC66A1L;SHOX2	dist=204274;dist=290537					
12M	rs139055031	3	158869060	6	3.17888515002842e-06	16.2853761	intergenic	MFSD1;IQCJ	dist=39341;dist=200192		1			
12M	rs143669489	3	159675096	3	4.93900179409246e-06	24.2253199	intronic	IQCJ-SCHIP1;SCHIP1	.					
3M	rs187047882	3	164567833	3	3.41360195160902e-06	18.5641361	intergenic	MIR1263;LINC01324	dist=396277;dist=146262			1		
3M	rs141169929	3	165090674	5	2.90582805983588e-06	14.8454178	intergenic	SI;SLITRK3	dist=12178;dist=96046					
3M	rs186649043	3	175214503	3	2.29531002487815e-06	19.2134209	intronic	NAALADL2	.			1		
3M	rs189709453	3	177856520	3	1.55548473782376e-06	23.2009936	ncRNA_intronic	LINC02015	.		1	1	1	
12M	rs189709453	3	177856520	3	1.55548473782376E-06	23.2009936	ncRNA_intronic	LINC02015	.					
3M	rs186767531	3	177921989	3	1.62563417403378E-07	21.2013243	intergenic	LINC02015;LINC01014	dist=22765;dist=497212					
12M	rs186767531	3	177921989	3	1.66219440747609E-06	23.0749977	intergenic	LINC02015;LINC01014	dist=22765;dist=497212					
3M	rs182868205	3	177994660	3	1.36806623320862e-06	22.8544303	intergenic	LINC02015;LINC01014	dist=95436;dist=424541					
12M	rs182868205	3	177994660	3	1.36806623320862E-06	22.8544303	intergenic	LINC02015;LINC01014	dist=95436;dist=424541					
12M	rs76356799	3	179875980	4	2.9143183363234e-07	19.2867007	intronic	PEX5L	.		1			
12M	rs117591241	3	190866890	4	4.37648501942309e-06	19.9014028	intergenic	GMNC;SNAR-I	dist=4196;dist=11040		1			
3M	rs191792521	3	195919734	6	2.52243498533689e-06	13.9515484	intergenic	TNK2;SDHAP1	dist=6470;dist=40187			1		

3M	rs113651406	4	997179	3	2.52148284014823e-07	19.4054026	intronic	IDUA	.				1	
3M	rs12502861	4	2424578	9	2.48774370313747e-06	11.1588472	intronic	CFAP99	.				1	
3M	rs189765693	4	4323066	4	2.55310143124462e-06	15.5191341	intergenic	ZBTB49;NSG1	dist=1283;dist=63466				1	
12M	rs189094663	4	11621826	3	2.89927274380224e-06	23.895416	intergenic	HS3ST1;LINC02360	dist=192932;dist=119125			1		
12M	rs557276277	4	19012075	5	3.35996743882432e-06	19.0146344	intergenic	LCORL;SLIT2	dist=990200;dist=1239830			1		
3M	rs183962155	4	21258020	6	3.64461882511902e-06	13.7646008	intronic	KCNIP4	.				1	
3M	rs113751774	4	23427231	7	5.00531306271799e-07	14.9103274	intergenic	GBA3;PPARGC1A	dist=607659;dist=364790		1	1		1
3M	rs113063005	4	23507444	6	1.79978248668523e-11	21.2073894	intergenic	GBA3;PPARGC1A	dist=687872;dist=284577					
12M	rs113063005	4	23507444	7	9.56810303773154E-07	16.61763	intergenic	GBA3;PPARGC1A						
3M	rs145875128	4	32071514	3	1.07037949708586e-07	24.479986	ncRNA_intronic	LINC02506	.				1	
3M	rs143287889	4	35570658	3	4.29676290263636e-06	19.96741	intergenic	LINC02484;ARAP2	dist=1300911;dist=495346					
3M	rs77141817	4	37052137	3	1.52230770368993e-08	20.6110292	intergenic	LINC02616;MIR4801	dist=31431;dist=189773		1			
3M	rs190822761	4	37097734	3	1.6069016808543e-08	20.5846354	intergenic	LINC02616;MIR4801	dist=77028;dist=144176					
12M	rs147267707	4	43024541	4	2.25472034099615e-06	18.0094385	intronic	GRXCR1	.			1		
12M	rs77618729	4	43048951	4	2.25749765325168e-06	18.0130153	intergenic	GRXCR1;LINC02383	dist=18293;dist=408583					
12M	rs556435089	4	43549016	4	2.61570608345655e-06	17.939079	intergenic	LINC02383;LINC02475	dist=56473;dist=467845					
12M	rs532464521	4	43569022	4	2.45873915171385e-06	17.9895528	intergenic	LINC02383;LINC02475	dist=76479;dist=447839					
3M	rs999769259	4	61646247	3	3.61294355616724e-06	22.1246725	intronic	ADGRL3	.				1	
3M	rs147630370	4	86529522	4	7.46814493303135e-07	16.0013633	intergenic	MAPK10;MIR4452	dist=76327;dist=12960				1	
3M	rs147171192	4	88214436	5	4.39967162056646e-06	13.3044047	intronic	ABCG2	.				1	
3M	rs142993106	4	90036221	15	3.08700493831608e-06	8.08463611	intergenic	MMRN1;CCSER1	dist=81611;dist=91173				1	
3M	rs146526206	4	90071867	15	1.81582969378392e-06	8.09176656	intergenic	MMRN1;CCSER1	dist=117257;dist=55527					
3M	rs17017794	4	90904734	33	3.46919898165651e-07	5.83240545	intronic	CCSER1	.					
3M	rs145116559	4	111756440	4	4.90673849934313e-06	18.2441224	intergenic	MIR297;FAM241A	dist=895793;dist=389014				1	
3M	rs181415102	4	111768110	4	4.81312717634891e-06	18.2631019	intergenic	MIR297;FAM241A	dist=907463;dist=377344					
3M	rs191423619	4	125595472	3	2.50684302750887e-07	21.694611	intergenic	MIR2054;INTU	dist=88165;dist=2037485				1	
3M	rs149298750	4	126237546	3	1.98725449883812e-07	22.184052	intergenic	MIR2054;INTU	dist=730239;dist=1395411					
12M	rs181132315	4	128726214	3	1.68603480568751e-06	22.1324679	intergenic	LINC02615;JADE1	dist=206818;dist=83486			1		
3M	rs112679237	4	138219706	18	2.04053672228041e-07	8.71202752	intronic	SLC7A11	.				1	
12M	rs147455971	4	140010558	5	3.87240564796723e-06	17.2160142	intronic	MAML3	.			1		
3M	rs531769270	4	153489891	3	2.4145887031028e-06	16.5945338	intronic	TMEM131L	.				1	
3M	rs116651654	4	162317591	6	3.48829177722726e-07	15.6223694	intergenic	FSTL5;MIR4454	dist=153557;dist=775983				1	
12M	rs17615362	4	169012936	44	4.30304118178725e-06	5.41974472	intergenic	CBR4;SH3RF1	dist=2681;dist=81323			1		
12M	rs17543620	4	169013574	44	4.21133121953689e-06	5.42796755	intergenic	CBR4;SH3RF1	dist=3319;dist=80685					
3M	rs567982164	5	25631188	3	5.7310337989489e-07	21.8356286	intergenic	LINC02211;CDH9	dist=328908;dist=1249409				1	
3M	rs191986449	5	25745578	3	4.59857674938651e-06	18.4978793	intergenic	LINC02211;CDH9	dist=443298;dist=1135019					
12M	rs142934021	5	53366842	7	1.40182538744286e-08	18.824715	intergenic	LOC257396;FST	dist=251716;dist=113787	1				
3M	rs185771987	5	73989664	3	4.53395085864185e-06	18.2344824	intergenic	ARHGEF28;LINC01335	dist=47671;dist=316746				1	
3M	rs139360368	5	74076284	4	1.24776973080952e-06	18.3590487	intergenic	ARHGEF28;LINC01335	dist=134291;dist=230126				1	
3M	rs181933850	5	92169830	7	3.33393070013435e-06	11.0436586	intergenic	ARRDC3;NR2F1	dist=749115;dist=1279415				1	
3M	rs190190051	5	92179668	7	3.47677941958718e-06	11.0589887	intergenic	ARRDC3;NR2F1	dist=758953;dist=1269577					
3M	rs182531466	5	92234256	6	4.23245053141191e-06	12.6804978	intergenic	ARRDC3;NR2F1	dist=813541;dist=1214989					
3M	rs187236873	5	92234630	6	1.97926852689169e-06	12.44907	intergenic	ARRDC3;NR2F1	dist=813915;dist=1214615					
3M	rs183816745	5	92336071	5	6.02067560021534e-08	17.3487909	intergenic	ARRDC3;NR2F1	dist=915356;dist=1113174					
3M	rs111407636	5	95744325	3	6.27809322802765e-07	18.642774	intronic	RHOBTB3	.				1	
3M	rs111676272	5	95758594	3	6.71649637293761e-07	18.6068625	intronic	RHOBTB3	.					
3M	rs75848314	5	95762636	3	1.11087241977704e-07	20.0998481	intronic	RHOBTB3	.					
3M	rs111846247	5	95775939	3	1.06810112370498e-07	20.4865772	intronic	RHOBTB3	.					
3M	rs137873790	5	97751337	11	4.51630701305597e-06	9.22240881	intergenic	LINC01340;LINC02234	dist=80286;dist=89421				1	
3M	rs189912648	5	135429749	4	2.55441427995549e-06	16.9732599	intergenic	MACROH2A1;DCANP1	dist=29862;dist=14465			1	1	1
12M	rs189912648	5	135429749	4	3.32990944802665E-06	19.8604449	intergenic	MACROH2A1;DCANP1	dist=29862;dist=14465					
12M	rs111512950	5	154300867	8	4.11671051184833e-06	13.567443	intronic	GALNT10	.			1		

3M	rs191006910	5	154836449	3	3.35533057793161e-06	17.6173803	intronic	FAXDC2	.			1
3M	rs74343174	5	162066176	5	2.54836335504837e-06	14.7671824	intergenic	LINC01202;GABRG2	dist=64980;dist=1289			1
3M	rs775626702	5	163203061	3	3.23411085027866e-06	18.9484137	intergenic	GABRG2;CCNG1	dist=1047522;dist=234510			
3M	rs371245624	5	163453950	3	1.65804063735814e-06	19.4795931	UTR3	NUDCD2	NM_001329991:c.*170>0;NM_145266:c.*170>0			1
3M	rs290120	5	163841238	6	2.40444258455957e-06	13.3587764	intergenic	MAT2B;LINC02143	dist=321884;dist=607184			1
3M	rs545428520	5	168394261	4	3.96137253437646e-09	21.566035	intronic	WWC1	.	1		
3M	rs528404963	5	168425020	4	7.1738299793335e-09	21.1393977	intronic	WWC1	.			
3M	rs72832764	5	170577669	3	2.69939738977966e-06	18.2159757	intronic	KCNIP1	.			1
3M	rs72837643	5	170761951	4	4.147895992623e-06	16.9302874	intergenic	KCNIP1;GABRP	dist=25319;dist=21768			
3M	rs142311947	5	177957468	8	2.99420386474277e-06	12.0339013	ncRNA_intronic	LOC100128340	.			1
3M	rs151015676	5	177963936	3	4.05781112465234e-06	20.1421367	intergenic	LOC100128340;PROP1	dist=4136;dist=28299			
3M	rs1877768	6	16534692	15	2.77630022916084e-06	8.28754685	intronic	ATXN1	.			1
12M	rs73384619	6	20457948	21	3.65476878017977e-06	8.3916702	intronic	E2F3	.		1	
3M	rs150586237	6	24491120	3	5.5145390836127e-09	22.0461443	intergenic	GPLD1;ALDH5A1	dist=1542;dist=3849	1		
3M	rs571986619	6	84969114	3	1.70115854486702e-06	20.7858067	intergenic	TBX18;LINC02535	dist=204516;dist=418105			1
3M	rs56224400	6	97644799	14	9.49951178557744e-07	8.4433064	ncRNA_intronic	LOC101927314	.			1
3M	rs147627638	6	98725240	6	2.84737109324869e-06	13.0785306	intergenic	MIR2113;PNKY	dist=700621;dist=104901			
3M	rs141326851	6	134511989	14	2.157242978017e-06	8.57849688	intergenic	LINC01010;LOC101928304	dist=7969;dist=13329			1
3M	rs146048121	6	141877818	3	3.23639603147815e-06	20.0214379	intergenic	MIR4465;NMBR	dist=1193935;dist=196666	1		
3M	rs142106992	6	141948748	4	5.23067431501407e-10	22.8345886	intergenic	MIR4465;NMBR	dist=1264865;dist=125736			
3M	rs72983831	6	141985963	6	7.82545989102806e-07	15.6733239	intergenic	MIR4465;NMBR	dist=1302080;dist=88521			
3M	rs72986533	6	142290121	8	1.53596722070896e-07	13.2812726	intergenic	VTA1;ADGRG6	dist=65437;dist=11798			
3M	rs73586304	6	142518288	3	4.95566451287885e-06	18.2405116	intergenic	ADGRG6;LOC153910	dist=72022;dist=8167			
12M	rs4896997	6	148362788	6	3.04539162547005e-06	14.4320923	intronic	SASH1	.		1	
12M	rs4131286	6	148367827	6	3.05925201460773e-06	14.4284672	intronic	SASH1	.			
12M	rs17078283	6	148375784	6	3.05230865558584e-06	14.4302821	intronic	SASH1	.			
12M	rs56821264	6	148382765	6	2.17257084724272e-06	14.7509213	intronic	SASH1	.			
3M	rs148532212	6	165086368	3	1.8149830669263e-07	19.3535457	intergenic	MEAT6;C6orf118	dist=264305;dist=193296			1
3M	rs117498042	6	165100792	3	1.85878685547635e-07	19.335471	intergenic	MEAT6;C6orf118	dist=278729;dist=178872			
3M	rs148153037	6	167087898	8	3.20376100252789e-09	14.3359246	intergenic	CEP43;CCR6	dist=35180;dist=23909	1		
3M	rs184487573	6	167099983	7	1.91568282565546e-07	13.5009635	intergenic	CEP43;CCR6	dist=47265;dist=11824			
3M	rs17776100	7	6386848	25	5.13308111425872e-07	6.63697397	intronic	RAC1	.			1
3M	rs187978759	7	11672218	3	2.76447739355703e-06	20.4555522	intronic	THSD7A	.			1
12M	rs143686474	7	16252374	11	4.88904023817646e-06	10.8531498	ncRNA_intronic	CRPPA	.		1	
3M	rs117166500	7	17013154	7	1.89815826168549e-06	12.5146865	intergenic	AGR3;AHR	dist=131171;dist=285498			1
3M	rs75689761	7	18366950	7	9.11790229535791e-07	11.8482917	intronic	HDAC9	.	1		
3M	rs77346868	7	18366976	8	1.13767941006556e-06	11.0210129	intronic	HDAC9	.			
3M	rs78225611	7	18367841	8	1.13375019479557e-06	11.0294387	intronic	HDAC9	.			
3M	rs77300464	7	18369138	6	2.09191655072049e-08	14.6659794	intronic	HDAC9	.			
3M	rs79602997	7	18370627	7	9.43076371109133e-07	11.8608211	intronic	HDAC9	.			
3M	rs75773869	7	18371222	7	9.36316662267224e-07	11.8642716	intronic	HDAC9	.			
3M	rs75606013	7	18374990	6	2.11098533218711e-08	14.6617119	intronic	HDAC9	.			
3M	rs61434999	7	18378728	7	9.22178787748122e-07	11.8411511	intronic	HDAC9	.			
3M	rs78907958	7	18385394	7	4.98811901973441e-07	12.0901322	intronic	HDAC9	.			
3M	rs76526501	7	18391487	6	8.57654056311165e-09	14.8397312	intronic	HDAC9	.			
3M	rs74455595	7	18392161	6	8.57654056311165e-09	14.8397312	intronic	HDAC9	.			
3M	rs79182806	7	18394204	7	4.13732512224196e-07	12.0972603	intronic	HDAC9	.			
3M	rs10279777	7	18401966	8	1.65309369490778e-08	12.8681335	intronic	HDAC9	.			
3M	rs77867199	7	18402652	9	3.93328414300688e-07	10.8221282	intronic	HDAC9	.			
3M	rs80156375	7	18403592	8	3.27106315853648e-07	11.5406255	intronic	HDAC9	.			
3M	rs17169602	7	18407118	8	1.78410513894506e-08	12.7273717	intronic	HDAC9	.			
3M	rs10486295	7	18407184	8	1.75006169477667e-08	12.76229	intronic	HDAC9	.			

3M	rs75090694	7	18407813	7	1.01013780102531e-08	13.8314005	intronic	HDAC9	.			
3M	rs55844051	7	23320744	3	1.93295366566624e-06	18.3875896	intronic	IGF2BP3	.			1
3M	rs62447184	7	36534898	43	2.45196683172423e-06	5.12842209	intronic	AOAH	.			
3M	rs574076561	7	49505151	3	3.8458987487847e-06	19.6434766	intergenic	CDC14C;VWC2	dist=577697;dist=268487			1
3M	rs111391231	7	89867731	12	4.17997193738163e-06	8.31717755	intergenic	ZNF804B;STEAP2	dist=529203;dist=14622			1
3M	rs111900874	7	89887355	12	3.79796761664437e-06	8.42424013	ncRNA_intronic	STEAP2	.			
3M	rs181259864	7	97859511	3	3.26388920997597e-06	17.1360821	ncRNA_intronic	CZ1P-ASNS	.			1
3M	rs192750513	7	97948518	3	3.9425756746568e-06	17.1892469	ncRNA_intronic	CZ1P-ASNS	.			
3M	rs539713344	7	100877165	3	2.02994489815685e-06	20.044756	intronic	SRRT	.			1
3M	rs188028357	7	101025144	3	5.38240258932335e-07	21.8425578	intronic	MUC17	.			1
12M	rs73202425	7	109515659	12	3.05259237348811e-06	10.761844	intergenic	C7orf66;EIF3IP1	dist=631072;dist=443568		1	
12M	rs181182636	7	116014504	3	2.90894506747647e-06	21.9472639	intronic	TFEC	.		1	
12M	rs182211730	7	116363561	4	1.45395858607717e-06	18.7746033	intergenic	LOC102724434;CAV2	dist=76834;dist=136177		1	
12M	rs143281973	7	116367942	4	1.62372890091138e-06	18.2750778	intergenic	LOC102724434;CAV2	dist=81215;dist=131796			
12M	rs138873576	7	116385305	4	3.22380485845586e-06	17.6151711	intergenic	LOC102724434;CAV2	dist=98578;dist=114433			
12M	rs190354334	7	116435792	5	4.2862377340545e-06	17.3392581	intergenic	LOC102724434;CAV2	dist=149065;dist=63946			
12M	rs186893139	7	116456916	4	3.55104961576984e-06	18.602598	intergenic	LOC102724434;CAV2	dist=170189;dist=42822			
12M	rs558784715	7	117403240	3	1.5182227326648e-06	20.7591787	intronic	ASZ1	.		1	
12M	rs576047962	7	117468967	3	1.61732320840498e-06	20.7261549	intergenic	ASZ1;CFTR	dist=41474;dist=11058			
12M	rs529110230	7	117535853	3	1.24629705452557e-06	20.8583633	intronic	CFTR	.			
12M	rs142215699	7	117559820	3	1.6210064953756e-06	20.7292287	ncRNA_intronic	CFTR	.			
12M	rs142721557	7	117578274	3	1.61401730397805e-06	20.7329975	intronic	CFTR	.			
12M	rs201355675	7	117585727	3	1.64203568676087e-06	20.718124	intronic	CFTR	.			
12M	rs188993522	7	117634677	3	1.9792198058251e-06	21.2984827	intronic	CFTR	.			
3M	rs536023430	7	147167980	3	2.63612685649085e-06	19.4038683	intronic	CNTNAP2	.			1
3M	rs187384541	8	1684075	26	3.15110880283135e-06	6.88745117	intronic	DLGAP2	.			1
3M	rs575473987	8	5719972	3	1.07256928472273e-08	22.2248653	intergenic	CSMD1;LOC100287015	dist=725058;dist=683583	1		
3M	rs139062456	8	13394482	15	3.88565669511608e-06	7.83515809	intronic	DLC1	.			1
3M	rs185874707	8	18173165	10	2.74810255839378e-06	9.13934765	intronic	NAT1	.			1
3M	rs140797780	8	22230279	3	1.73425396214273e-07	19.2908396	intronic	PHYHIP	.			1
3M	rs147601511	8	22464806	4	7.14867637986866e-07	16.4471181	intronic	PPP3CC	.			1
3M	rs188415494	8	25755782	3	3.49911568838158e-06	18.5169478	intergenic	CDCA2;EBF2	dist=247865;dist=85943			1
3M	rs9643828	8	54616513	279	2.29737457097614e-07	-2.4236294	intronic	RP1	.			1
3M	rs423841	8	54643509	291	2.7508818251862e-06	-2.1495726	intronic	RP1	.			
3M	rs433324	8	54652049	287	9.22325760191311e-07	-2.2483462	intronic	RP1	.			
3M	rs369623	8	54659380	287	8.50610562336969e-07	-2.2359815	intronic	RP1	.			
3M	rs446222	8	54662400	288	7.36959451875675e-07	-2.247819	intronic	RP1	.			
3M	rs432393	8	54667738	291	1.22958170848882e-06	-2.1875382	intronic	RP1	.			
3M	rs3098298	8	54670278	291	1.22819650607779e-06	-2.1875635	intronic	RP1	.			
3M	rs367179	8	54675056	291	1.22819650607779e-06	-2.1875635	intronic	RP1	.			
3M	rs382476	8	54678415	288	7.36949131111935e-07	-2.2481938	intronic	RP1	.			
3M	rs384543	8	54679049	288	7.36949131111935e-07	-2.2481938	intronic	RP1	.			
3M	rs405226	8	54679776	291	1.2251324121268e-06	-2.1881305	intronic	RP1	.			
3M	rs384127	8	54684929	288	7.36964497256014e-07	-2.2481559	intronic	RP1	.			
3M	rs858397	8	54702130	278	7.161979275107e-07	2.2301348	intronic	RP1	.			
3M	rs2375537	8	54706948	280	6.2991993988762e-07	2.23876848	intronic	RP1	.			
3M	rs720372	8	54716077	292	2.93014534156507e-06	2.09872734	intronic	RP1	.			
3M	rs1437781	8	54717292	280	6.32222203401203e-07	2.23854673	intronic	RP1	.			
3M	rs1595406	8	54718055	291	2.80263765648492e-06	2.10018889	intronic	RP1	.			
3M	rs1437782	8	54720202	278	5.53724964407086e-07	2.2579585	exonic	RP1	.			
3M	rs10105693	8	54727912	276	3.41214459494352e-07	2.2928391	intronic	RP1	.			
3M	rs2375536	8	54728162	292	2.13408172799341e-06	2.12635806	intronic	RP1	.			
3M	rs4737674	8	54749094	277	2.86005059848221e-07	2.30559554	intronic	RP1	.			

3M	rs11987234	8	54757269	276	3.13190459972498e-07	2.29599259	intronic	RP1	.			
3M	rs13277510	8	54761589	277	2.8610568625199e-07	2.30494752	intronic	RP1	.			
3M	rs12548593	8	54762057	279	3.38344249342945e-07	2.28269961	intronic	RP1	.			
3M	rs1812506	8	54763541	291	2.00833101672028e-06	2.12258868	intronic	RP1	.			
3M	rs16920698	8	54765874	277	2.85868793626322e-07	2.30492695	intronic	RP1	.			
3M	rs1561297	8	54765978	279	3.34668102178151e-07	2.28315237	intronic	RP1	.			
3M	rs4737676	8	54766986	277	2.8589296796158e-07	2.30492057	intronic	RP1	.			
3M	rs2083123	8	54767758	279	3.4358513898515e-07	2.27910794	intronic	RP1	.			
3M	rs983248	8	54768232	277	2.87356270418993e-07	2.30260428	intronic	RP1	.			
3M	rs1391463	8	54769316	277	2.87382622216009e-07	2.30258865	intronic	RP1	.			
3M	rs10958428	8	54773081	280	5.0112447137886e-07	2.23996885	intronic	RP1	.			
3M	rs13278605	8	54775611	277	3.41581916245031e-07	2.27377348	intronic	RP1	.			
3M	rs13276543	8	54775614	276	3.13999361335922e-07	2.29263157	intronic	RP1	.			
3M	rs7822082	8	54777660	277	1.5040633792954e-07	2.3570878	intronic	RP1	.			
3M	rs4737201	8	54778898	277	2.89096388651053e-07	2.30197734	intronic	RP1	.			
3M	rs7843693	8	54779552	334	4.78812399757755e-06	1.99856756	intronic	RP1	.			
3M	rs1396896	8	54782750	334	4.77810054425197e-06	1.99853327	intronic	RP1	.			
3M	rs2375219	8	54785735	330	3.05253330469401e-06	2.05700167	intronic	RP1	.			
3M	rs1391462	8	54787221	334	4.79440218462524e-06	1.9984766	intronic	RP1	.			
3M	rs12678939	8	54792461	331	4.93547251213243e-06	2.01975618	intronic	RP1	.			
3M	rs1498183	8	54804345	332	4.76724404445351e-06	2.0245746	intronic	RP1	.			
12M	rs62515405	8	56143419	21	4.69438076015372e-06	8.07691204	intergenic	MOS;PLAG1	dist=29437;dist=17490		1	
12M	rs62515436	8	56228644	15	3.44618686083172e-06	9.27545902	intergenic	CHCHD7;SDR16C5	dist=9835;dist=71004		1	
12M	rs12114488	8	61760164	266	1.33492404675942e-06	2.62307914	intergenic	MIR4470;LINC02155	dist=45305;dist=129675		1	
3M	rs117816016	8	102739034	3	3.21886378098173e-06	19.8807023	intergenic	LOC101927245;GASAL1	dist=52311;dist=67788			1
3M	rs567383525	8	114017265	3	1.83606414606387e-06	17.7455227	intergenic	CSMD3;TRPS1	dist=580326;dist=1391230			1
3M	rs545550279	8	114540200	3	4.75001774825929e-06	19.1812421	intergenic	CSMD3;TRPS1	dist=1103261;dist=868295			
3M	rs536803366	8	121989172	3	1.65900394985684e-06	19.2098639	intergenic	HAS2;SMILR	dist=343847;dist=425155			1
3M	rs532513136	8	134801505	3	7.47175269151994e-07	19.9490594	upstream	MIR30B	dist=898			1
3M	rs532730683	9	1784492	3	1.02051348042702e-06	18.726838	intergenic	DMRT2;SMARCA2	dist=726938;dist=230855			1
3M	rs540065886	9	2770228	3	1.90434082268377e-06	19.9489151	intergenic	KCNV2;PUM3	dist=40191;dist=33927			1
3M	rs543844012	9	30107025	3	4.37741040782571e-06	18.1160485	intergenic	LINGO2;LINC01242	dist=893424;dist=281910			1
3M	rs148556485	9	81408911	3	5.40048598404711e-07	21.3800152	intergenic	LINC01507;TLE1	dist=1374356;dist=174772			1
3M	rs140782222	9	81413979	3	4.53117855536307e-07	21.791526	intergenic	LINC01507;TLE1	dist=1379424;dist=169704			
3M	rs188034471	9	83322799	4	2.79197739516285e-06	14.7559999	intronic	FRMD3	.			1
3M	rs190294315	9	83330550	5	4.29120280825722e-07	14.5664276	intronic	FRMD3	.			
3M	rs545690161	9	90268417	6	1.13978419923937e-08	17.1436874	intergenic	MIR4290HG;LINC01508	dist=226918;dist=32479	1		
3M	rs565682685	9	90460046	4	3.57832955336558e-07	18.938397	intergenic	LINC01508;LINC01501	dist=26557;dist=2386			
3M	rs183737367	9	90567765	3	3.8014567877404e-08	23.3452306	ncRNA_intronic	LINC01501	.			
3M	rs187213609	9	90653183	3	5.71707003010382e-08	23.1975304	intergenic	DIRAS2;SYK	dist=10359;dist=148417			
12M	rs144138711	9	97160137	40	1.39149931661618e-06	6.08547131	ncRNA_intronic	ANKRD18CP	.		1	
3M	rs150027952	9	100623134	3	1.2828864296083e-06	21.8616648	intergenic	CAVIN4;PLPPR1	dist=34747;dist=405593			1
3M	rs146207930	9	126280057	6	2.39787202381566e-06	11.8624321	intergenic	LOC101929116;MVB12B	dist=4142;dist=46772			1
3M	rs78296164	9	132391328	7	6.93811773227571e-07	11.6095338	intronic	TTF1	.			1
3M	rs77871739	9	135660463	4	3.19831184275799e-06	15.0724603	intergenic	GLT6D1;LCN9	dist=20923;dist=2859			1
3M	rs118040657	10	3430654	8	4.61906712585886e-06	12.1147712	ncRNA_intronic	LOC105376360	.			1
3M	rs184425183	10	13415520	3	1.92440370103676e-09	22.6683174	intergenic	SEPHS1;BEND7	dist=67222;dist=22961	1		
3M	rs184458518	10	13429195	3	1.48323670154931e-09	22.8015789	intergenic	SEPHS1;BEND7	dist=80897;dist=9286			
3M	rs117998251	10	13455976	3	1.19486099712372e-09	22.9543311	intronic	BEND7	.			
3M	rs117025967	10	19918123	11	5.25165736443477e-07	9.66973563	intronic	PLXDC2	.			1
3M	rs529011661	10	20083387	4	2.34383539239577e-06	15.6393469	intronic	PLXDC2	.			
12M	rs142956968	10	23201236	14	8.84691036334147e-07	10.4167992	downstream	C10orf67	dist=680		1	
12M	rs145764464	10	23312918	15	1.10461979254428e-06	9.5967306	intronic	C10orf67	.			

3M	rs12266995	10	24563854	26	3.92980514429013e-06	5.96782535	intergenic	KIAA1217;ARHGAP21	dist=16006;dist=19760			1
3M	rs138249376	10	61756071	4	4.86147789321271e-06	16.4300711	intronic	CABCOCO1	.			1
3M	rs140277951	10	80599344	6	1.98902982335697e-07	13.7463158	intronic	SH2D4B	.			1
3M	rs566018180	10	84995296	3	4.3799943792317e-06	18.563552	intergenic	CCSER2;LINC01519	dist=476775;dist=198125			1
3M	rs140706881	10	94457778	4	1.40251021425162e-06	18.974805	intronic	TBC1D12	.			1
3M	rs117913371	10	101158729	21	2.30639701253664e-08	7.86609555	intergenic	TLX1NB;LINC01514	dist=17463;dist=17593	1		
3M	rs75334617	10	101196395	32	7.30335368747825e-07	5.84603405	intergenic	LINC01514;LBX1	dist=2248;dist=30581			
3M	rs752259256	10	103165562	3	4.52164287436037e-06	20.4258639	intronic	NT5C2	.			1
12M	rs147944608	10	108663951	11	9.83587566974791e-07	11.2205243	intergenic	LINC01435;XPNPEP1	dist=594658;dist=1200815		1	
3M	rs180828621	10	122773893	6	6.39935885478647e-08	15.1380574	ncRNA_intronic	DMBT1L1	.			1
3M	rs147393020	10	123019758	5	1.64586496415273e-06	14.1853505	intronic	ACADSB	.			1
3M	rs193093906	10	125016920	9	3.17719154902332e-06	10.5219537	intronic	CTBP2	.			1
12M	rs556274646	11	439012	9	1.93909342324625e-06	12.8914592	intronic	ANO9	.		1	
12M	rs148815783	11	1013992	8	2.06734210421034e-06	13.5980313	exonic	MUC6	.			1
3M	rs151115079	11	18634194	5	4.89469717121508e-08	17.8273587	intronic	SPTY2D1	.	1		
3M	rs138414342	11	18657851	5	3.15830767684997e-08	18.1582505	intergenic	SPTY2D1;TMEM86A	dist=23509;dist=40928			
3M	rs541653703	11	18680239	4	2.1765671670172e-08	18.3169499	intergenic	SPTY2D1;TMEM86A	dist=45897;dist=18540			
3M	rs118093638	11	18696777	5	9.7151911784911e-07	14.2657259	intergenic	SPTY2D1;TMEM86A	dist=62435;dist=2002			
3M	rs181812512	11	66898258	3	4.24789238469822e-06	19.5093168	intronic	PC	.			1
3M	rs529345909	11	67343381	3	4.87261379674454e-06	18.70153	ncRNA_intronic	LOC100130987	.			1
3M	rs544042801	11	68692775	4	3.07835940417967e-06	15.8869866	intergenic	GAL;TESMIN	dist=1600;dist=14665			1
3M	rs149949098	11	95366702	13	2.52728186016237e-06	8.55927625	intergenic	LOC100129203;FAM76B	dist=132298;dist=402251			1
3M	rs74521112	11	99218416	27	3.48297260681022e-07	6.25632387	intronic	CNTN5	.			1
3M	rs79213709	11	99222724	26	5.77809319512938e-07	6.17081884	intronic	CNTN5	.			
3M	rs112007361	11	99317649	26	2.27561716225072e-07	6.35658468	intronic	CNTN5	.			
3M	rs148781275	11	103769875	5	2.2488967712367e-06	15.7111853	intergenic	DYNC2H1;MIR4693	dist=290012;dist=80031			1
3M	rs141281289	11	123822316	6	3.07596924681855e-07	15.811518	intergenic	OR6M1;TMEM225	dist=15967;dist=60603			1
3M	rs79539453	11	125396457	3	1.65356394618456e-06	20.8220616	intronic	PKNOX2	.			1
3M	rs528140343	11	125849332	3	9.33313906476653e-07	18.2835521	intergenic	PATE4;HYLS1	dist=9260;dist=34282			1
3M	rs546409459	11	125885094	3	2.2842558352674e-06	18.9195474	intronic	HYLS1	.			
3M	rs528609331	11	125972300	3	7.07196590950882e-07	19.5812418	intronic	CDON	.			1
3M	rs7104959	11	129976231	3	3.12645341825951e-06	17.9204611	intronic	PRDM10	.			1
12M	rs117699122	12	451096	4	1.18257175082115e-06	21.4238256	intergenic	CCDC77;B4GALNT3	dist=8456;dist=8843		1	
3M	rs189360484	12	1761344	4	3.94900202113111e-07	16.8078794	intronic	ADIPOR2	.			1
12M	rs61918041	12	6572702	21	4.39893214125342e-06	7.97264643	intronic	CHD4	.		1	
12M	rs113244573	12	6575219	21	4.49284300484012e-06	7.97095746	intronic	CHD4	.			
3M	rs141754456	12	19998198	7	1.82427554715572e-07	12.1460333	intergenic	AEBP2;LINC02398	dist=475971;dist=16487			1
3M	rs118184666	12	20271815	6	2.47036908420995e-06	10.9509803	intergenic	LINC02468;PDE3A	dist=143914;dist=96722			1
3M	rs549931083	12	20363352	3	3.06794275866128e-07	14.3282694	intergenic	LINC02468;PDE3A	dist=235451;dist=5185			
3M	rs151323346	12	20859090	4	2.59127051015029e-06	15.8903888	intronic	SLCO1B3;SLCO1B3-SLCO1B7	.			
3M	rs371879555	12	22863028	3	1.69721932161597e-06	20.1033765	intergenic	ETNK1;LOC101928441	dist=172363;dist=312608			1
3M	rs183466664	12	26668754	4	3.57331936763708e-06	16.0181882	intronic	ITPR2	.			1
3M	rs77353774	12	28095919	6	1.00362822173041e-06	13.1054362	intergenic	PTHLH;LOC729291	dist=123186;dist=89706			1
3M	rs113167689	12	28283029	6	2.31830826822625e-07	14.1364135	intronic	CCDC91	.			1
3M	rs17510814	12	28316036	6	2.30925017950923e-07	14.0387924	intronic	CCDC91	.			
3M	rs141756120	12	28358163	6	2.4075549984719e-07	14.278403	intronic	CCDC91	.			
3M	rs117991215	12	28358540	6	2.50265389686764e-07	14.333666	intronic	CCDC91	.			
3M	rs191930622	12	47890872	3	4.88013920852721e-06	19.6783579	intronic	VDR	.			1
3M	rs56302696	12	47899047	3	4.02736708601484e-06	19.8959041	intronic	VDR	.			
3M	rs185620578	12	48175616	3	2.41563563938621e-06	20.7232267	intergenic	ASB8;CCDC184	dist=18101;dist=8028			1
3M	rs190806532	12	48468364	3	1.54808652729946e-06	21.326955	intergenic	ZNF641;ANP32D	dist=117118;dist=4195			1
3M	rs568658857	12	49460215	5	4.20762702895843e-06	14.2361193	intronic	SPATS2	.			1

3M	rs137880949	12	62912517	3	1.74526564511423e-07	21.6084034	intronic	PPM1H	.	1				
3M	rs191053292	12	63051500	3	2.51400610111527e-08	22.9266488	intergenic	PPM1H;AVPR1A	dist=116350;dist=91259					
3M	rs182437250	12	63214686	4	7.11750755969757e-07	18.8083451	intergenic	AVPR1A;DPY19L2	dist=63485;dist=344227					
3M	rs12315614	12	64527177	64	4.12213451917224e-06	3.77710662	intergenic	TBK1;RASSF3	dist=25064;dist=83318			1		
12M	rs149616342	12	98785035	10	4.17235147721501e-06	11.2978214	intronic	ANKS1B	.		1			
3M	rs76327548	12	100789188	11	2.03544908278967e-06	9.17842714	intergenic	GAS2L3;ANO4	dist=160900;dist=5588			1		
3M	rs76904423	12	100794966	10	3.57310045627432e-06	9.80576231	UTR5	ANO4	NM_001286615:c.-1068200>0;NM_178826:c.-1068200>0					
3M	rs180764936	12	101104843	3	1.48879381343194e-06	19.3473053	intronic	ANO4	.					
3M	rs185855183	12	101111348	3	1.21523926305084e-06	18.7397727	intronic	ANO4	.					
12M	rs566724618	13	22889700	3	1.0727068950386e-06	22.3433705	ncRNA_intronic	LINC00621	.	1	1			1
12M	rs147221953	13	22911583	3	1.86149707525764e-06	22.5809676	ncRNA_intronic	LINC00621	.					
3M	rs139598422	13	23312875	4	1.84258662797026e-08	20.4886522	intronic	SGCG	.					
3M	rs143371352	13	46809699	3	1.20142384300403e-07	20.5341625	intergenic	ESD;HTR2A	dist=12538;dist=21847			1		
3M	rs75186966	13	46821623	3	1.30324459401267e-07	20.2281345	intergenic	ESD;HTR2A	dist=24462;dist=9923					
3M	rs150077525	13	57405415	7	1.65979661476121e-06	12.9984875	intergenic	PRR20E;PCDH17	dist=235197;dist=226329			1		
3M	rs534845494	13	57639730	5	5.5584801687088e-07	15.9431765	intronic	PCDH17	.					
3M	rs140062526	13	58711899	5	3.07703679852045e-06	14.0926088	intergenic	LINC00374;DIAPH3	dist=478782;dist=953688			1		
12M	rs113791989	13	61344755	3	7.13417559871126e-07	25.2049582	intergenic	MIR3169;PCDH20	dist=144874;dist=64931		1			
12M	rs532269430	13	73480315	5	9.38644328296013e-07	18.6173752	intergenic	KLF5;LINC00392	dist=402772;dist=83929		1			
12M	rs368187808	13	73783371	3	1.40533873966635e-06	21.2991251	intronic	KLF12	.		1			
12M	rs372194899	13	73783373	3	1.40312572084772e-06	21.3012101	intronic	KLF12	.					
3M	rs546286713	13	90758825	4	2.98443520289082e-06	15.7027841	intergenic	LINC01049;LINC00410	dist=223484;dist=132129			1		
3M	rs567080482	13	94423151	4	2.95390135279034e-06	15.4999561	intergenic	GPC6;DCT	dist=15132;dist=13660			1		
3M	rs142928734	13	100948828	3	4.29382451218382e-06	17.009593	ncRNA_intronic	NALCN	.			1		
3M	rs556680896	13	100950161	3	4.26743541179837e-06	17.0254659	ncRNA_intronic	NALCN	.					
3M	rs184265355	13	107359004	6	7.66559973381936e-07	14.4271719	intronic	FAM155A	.			1		
3M	rs572961122	13	107360637	6	3.07761414918354e-06	13.3354896	intronic	FAM155A	.					
3M	rs528809914	13	112386942	3	2.27269814316061e-07	19.5048552	intronic	SPACA7	.			1		
3M	rs180765647	13	113724338	3	4.20477084480749e-06	20.1246521	intronic	GRK1	.			1		
3M	rs138215817	14	22173619	4	6.27722428502198e-07	16.311019	intergenic	OR4E1;LOC105370401	dist=502284;dist=206292			1		
3M	rs74704551	14	29692681	3	1.97941397505543e-08	24.1990705	intronic	PRKD1	.	1				
3M	rs1686289	14	45791779	277	3.52212847732742e-06	-2.2551539	intergenic	LINC02303;LINC00871	dist=76177;dist=272380			1		
3M	rs176783	14	45811710	267	3.87832460630423e-06	2.28010147	intergenic	LINC02303;LINC00871	dist=96108;dist=252449					
3M	rs176786	14	45813767	268	3.37672684888795e-06	2.28475641	intergenic	LINC02303;LINC00871	dist=98165;dist=250392					
3M	rs428110	14	45825457	265	4.15182083903445e-06	2.28482299	intergenic	LINC02303;LINC00871	dist=109855;dist=238702					
3M	rs116862847	14	63674959	7	2.1204003875388e-07	15.3160369	intergenic	WDR89;SGPP1	dist=33088;dist=9258			1		
3M	rs569916471	14	75424639	5	3.10710931450174e-06	15.6404088	intergenic	LINC01220;JDP2	dist=128231;dist=3085			1		
3M	rs113767990	14	81251219	4	4.97864018338844e-06	14.5811155	intergenic	LOC101928504;STON2	dist=27839;dist=9431			1		
3M	rs190251199	14	105124240	3	6.2650518901636e-07	20.0125292	intergenic	LINC02298;JAG2	dist=24744;dist=16758		1	1	1	
12M	rs190251199	14	105124240	3	1.9790595982044E-06	22.6385061	intergenic	LINC02298;JAG2	dist=24744;dist=16758					
12M	rs187281112	15	32821186	5	3.3019635783423e-06	14.7343356	intronic	FMN1	.		1			
12M	rs56324718	15	39987872	9	7.5163534768267e-07	12.5117374	intronic	EIF2AK4	.		1			
3M	rs185155853	15	40951902	5	3.01500290127566e-06	15.9164657	intergenic	DLL4;CHAC1	dist=12829;dist=1569			1		
3M	rs144026361	15	40956471	5	2.67002672054584e-06	16.1372994	UTR3	CHAC1	NM_001142776:c.*6970>0;NM_024111:c.*6970>0					
3M	rs558614420	15	41518672	6	3.91771199682616e-06	13.9750831	intronic	RPAP1	.			1		
3M	rs138109686	15	41759244	6	4.62000628076245e-06	13.5956705	intronic	MGA	.			1		
3M	rs145896760	15	41827024	6	4.80897800219791e-06	13.3688825	UTR3	MAPKBP1	NM_014994:c.*15880>0;NM_001128608:c.*15880>			1		
3M	rs140642138	15	41832967	6	4.54678591939158e-06	13.5832221	intronic	JMJD7;JMJD7-PLA2G4B	.			1		
3M	rs6080	15	58545734	36	4.85754782149583e-06	5.66476745	intronic	LIPC	.			1		
3M	rs145439370	15	58587566	26	9.02072868608363e-07	6.89328891	intergenic	LIPC;ADAM10	dist=17722;dist=1243					
3M	rs149425014	15	58659461	22	1.84020904634255e-06	7.55224792	intronic	ADAM10	.					
3M	rs146442492	15	58689916	24	3.24578114232023e-06	7.05925858	intronic	ADAM10	.					

3M	rs193253461	15	58937154	12	3.76206055565965e-07	10.5374468	intergenic	SLTM;RNF111	dist=3475;dist=50509				1	
3M	rs184117160	15	59112107	11	1.25384693259541e-06	10.3417236	intronic	CCNB2	.				1	
3M	rs80292573	15	59142887	30	7.87214777000235e-07	6.55323491	intronic	MYO1E	.				1	
3M	rs182303755	15	59342593	12	4.69869428218951e-07	10.5479917	intronic	MYO1E	.					
3M	rs138217865	15	93849653	4	1.29412742467123e-06	16.709641	intergenic	LOC105370980;LINC02207	dist=641605;dist=6907				1	
12M	rs80212581	16	6362966	3	6.33070894509269e-07	22.2762003	intronic	RBFOX1	.	1				
12M	rs140276610	16	6383734	4	1.75581952039286e-06	22.1806577	intronic	RBFOX1	.					
12M	rs138164904	16	6808238	3	3.60740717035677e-08	23.892641	intronic	RBFOX1	.					
3M	rs553840536	16	25686574	3	3.61488566678171e-06	19.6203442	intergenic	ZKSCAN2;HS3ST4	dist=428729;dist=5385				1	
3M	rs183817723	16	59268871	4	2.48366342700424e-06	17.5860507	intergenic	GOT2;APOOP5	dist=534555;dist=485270				1	
3M	rs144954214	16	76145464	3	8.96489764315735e-07	21.8113263	intergenic	CPHXL;CNTNAP4	dist=418974;dist=131937				1	
3M	rs529523094	16	77681654	3	1.09798611725829e-06	18.1584346	intergenic	ADAMTS18;NUDT7	dist=246620;dist=40838				1	
12M	rs116897913	17	16935848	3	4.78180386603079e-06	20.1396765	intergenic	TBC1D27P;TNFRSF13B	dist=2672;dist=3233			1		
3M	rs146728064	17	19362127	6	3.79796041699169e-06	12.6400411	intronic	B9D1	.				1	
12M	rs188353596	17	48001236	3	6.85080774831596e-07	21.8057947	intergenic	CDK5RAP3;COPZ2	dist=19450;dist=24931			1		
3M	rs184613584	17	50430860	6	5.40834238559884e-07	14.1377974	intronic	ACSF2	.				1	
3M	rs191271637	17	54045899	3	2.76242881040307e-06	18.379836	intergenic	KIF2B;TOM1L1	dist=220706;dist=854792				1	
12M	rs112148840	17	68237633	3	3.82795090389498e-06	24.0241075	intronic	AMZ2	.			1		
12M	rs2606194	17	79214741	44	9.99647458522552e-07	-5.6956979	intronic	RBFOX3	.			1		
12M	rs147669485	18	29023686	4	9.8819660331789e-08	22.1107639	intergenic	CDH2;MIR302F	dist=846556;dist=1275226			1	1	1
3M	rs185819304	18	29421615	3	4.31513948321026e-07	19.9401113	intergenic	CDH2;MIR302F	dist=1244486;dist=877296					
3M	rs187942235	18	29450465	3	2.77341075652478e-07	20.3968658	intergenic	CDH2;MIR302F	dist=1273336;dist=848446					
3M	rs139493286	18	31236056	3	3.23770514196012e-06	17.4603093	intergenic	DSC1;DSG1	dist=73200;dist=82104				1	
3M	rs143538552	18	31470299	3	1.40612679764676e-07	19.1457218	intronic	DSG3	.					
3M	rs373746073	18	31478421	3	1.55466285461099e-07	19.0847197	UTR3	DSG3	NM_001944:c.*21610>0					
3M	rs146333745	18	57830225	4	1.04499182739545e-06	17.4215784	intergenic	ATP8B1;NEDD4L	dist=26910;dist=214001				1	
12M	rs559152067	18	68362375	3	1.80839131277764e-06	24.7443692	intergenic	LOC643542;TMX3	dist=462756;dist=311313			1		
3M	rs185464792	19	18686561	3	5.22729611714582e-07	22.5896015	intronic	CRTC1	.				1	
3M	rs186768950	19	18695314	3	4.67656376928778e-07	22.7152061	intronic	CRTC1	.					
3M	rs541288561	19	18758635	5	6.19920480169239e-07	15.6119012	intronic	CRTC1	.					
3M	rs559008174	19	18765249	5	6.10577196408926e-07	15.6025661	intronic	CRTC1	.					
3M	rs570407448	19	18769220	5	7.02455289936315e-07	15.4977984	intronic	CRTC1	.					
3M	rs546144116	19	19452530	3	2.29332366104571e-07	23.1354815	intronic	GATAD2A	.				1	
3M	rs560206697	19	20546292	3	4.01812636832912e-08	24.9989943	intronic	ZNF737	.		1			
3M	rs111285015	19	22940396	3	3.3078789506342e-09	27.3347642	intergenic	ZNF723;ZNF728	dist=81729;dist=34487		1			
3M	rs1008091735	19	30599192	3	5.61396767442543e-07	18.4689243	intronic	ZNF536	.				1	
3M	rs148433854	19	30605571	3	4.40546670794092e-07	18.6267585	intronic	ZNF536	.					
12M	rs367732718	19	35427102	3	3.38991794276911e-06	23.8784923	intergenic	LINC01531;FFAR2	dist=10262;dist=21155			1		
12M	rs562831582	19	50731053	4	5.70199111188332e-07	21.0856548	intergenic	CLEC11A;GPR32	dist=5345;dist=39411			1		
12M	rs62192733	20	1947511	7	2.76211842636971e-06	15.744618	ncRNA_exonic	PDYN	.			1		
12M	rs547186621	20	6126094	4	2.39683963851735e-06	21.5510829	intergenic	FERMT1;CASC20	dist=3064;dist=300638			1		
3M	rs2327968	20	15832846	21	4.58567809597369e-06	6.15899856	intronic	MACROD2	.				1	
3M	rs2876414	20	15833059	19	4.41048907235012e-06	6.81910907	intronic	MACROD2	.					
3M	rs140788628	20	15877856	8	2.32626831948532e-07	11.8039284	intronic	MACROD2	.					
3M	rs559228693	20	15982684	5	3.15016069009906e-06	14.3643551	ncRNA_intronic	LOC613266	.					
12M	rs138733283	20	32087884	5	5.87309369634527e-07	18.2744744	intronic	HCK	.			1		
12M	rs149859280	20	32090367	5	5.71700141373349e-07	18.3057707	intronic	HCK	.					
12M	rs146249289	20	32094428	4	2.01850841331451e-07	19.6109527	intronic	HCK	.					
12M	rs145791959	20	32134626	5	8.67001886662025e-07	17.9091953	intronic	TM9SF4	.			1		
12M	rs193041547	20	32184077	4	1.80079654856719e-07	19.6791358	intergenic	TM9SF4;TSPY26P	dist=16819;dist=5069					
12M	rs138055631	20	32192841	5	1.06243999550455e-06	17.6647762	UTR3	PLAGL2	NM_002657:c.*36110>0					
12M	rs145421321	20	32274323	6	1.72232779371331e-06	17.0212611	intergenic	POFUT1;KIF3B	dist=35665;dist=3328					

12M	rs143432612	20	32304972	6	1.74726298083525e-06	16.9387232	intronic	KIF3B	.			
12M	rs139816293	20	32333540	6	1.16528363587405e-06	17.1384248	UTR3	KIF3B	NM_004798:c.*22210>0			
12M	rs200198574	20	32358851	6	2.44213593266147e-06	16.5282735	intronic	ASXL1	.		1	
12M	rs148157126	20	32361036	5	1.42669073977956e-07	19.7865001	intronic	ASXL1	.			
12M	rs192855100	20	32520938	4	4.17301607264236e-07	20.1986097	intronic	NOL4L	.		1	
3M	rs557092705	20	35601989	3	4.54067714932956e-06	19.4769546	ncRNA_exonic	FER1L4	.			1
12M	rs2427460	20	62959430	420	5.30063377285508e-07	-2.574044	intronic	SLC17A9	.		1	
3M	rs184785969	21	15799112	3	8.26040996676763e-08	19.8736486	intronic	USP25	.	1		
3M	rs117280553	21	15834844	3	5.79538719308743e-08	20.087417	intronic	USP25	.			
3M	rs79486609	21	15872687	3	4.53094466952756e-08	20.6624886	intronic	USP25	.			
3M	rs73227413	21	21764653	28	2.74560920737872e-06	5.96205514	ncRNA_intronic	LINC01425	.			
3M	rs75024143	21	21784226	12	2.0964109268482e-06	9.81254241	ncRNA_intronic	LINC01425	.			
3M	rs192134381	21	22078395	3	5.23445134161758e-09	21.788812	ncRNA_intronic	LINC01687	.	1		
3M	rs118183140	21	34105187	17	1.16709964426479e-06	7.5893248	UTR3	SLC5A3	NM_006933:c.*78320>0			1
3M	rs183586634	21	37390730	5	1.72123504106205e-06	14.3132202	intronic	DYRK1A	.			1
3M	rs117185941	21	37394182	5	1.2357093777619e-06	15.3808786	intronic	DYRK1A	.			
3M	rs118084887	21	37491518	5	1.91359719468201e-06	14.9382616	intronic	DYRK1A	.			
12M	rs9636964	21	39932840	115	2.72523192147064e-07	-4.2543192	intergenic	PCP4;DSCAM	dist=3448;dist=78161		1	
12M	rs9305683	21	39933795	112	8.57246540213203e-07	-4.1163679	intergenic	PCP4;DSCAM	dist=4403;dist=77206			
12M	rs9974985	21	39935648	114	1.59836223443899e-07	-4.3459395	intergenic	PCP4;DSCAM	dist=6256;dist=75353			
12M	rs7275595	21	39935998	113	3.24552446398997e-07	-4.2609754	intergenic	PCP4;DSCAM	dist=6606;dist=75003			
12M	rs1005412	21	39937023	112	1.75853470115381e-07	-4.4521096	intergenic	PCP4;DSCAM	dist=7631;dist=73978			
12M	rs9981433	21	39937640	116	1.14042775714184e-06	-4.1220124	intergenic	PCP4;DSCAM	dist=8248;dist=73361			
3M	rs150539922	21	41856807	3	4.79442196264294e-06	16.6696937	intronic	PRDM15	.			1
3M	rs113625788	22	19981659	7	1.17014602462676e-06	11.8084829	exonic	ARVCF	.			1
3M	rs78547898	22	32428291	3	4.85417555255089e-07	20.9098395	intronic	BPIFC	.			1
12M	rs77297738	22	34077554	16	2.01836979224915e-07	10.2480572	intergenic	LARGE1;LINC02885	dist=154731;dist=679113		1	
12M	rs74572772	22	34096660	16	1.39505561568774e-07	10.2225183	intergenic	LARGE1;LINC02885	dist=173837;dist=660007			
12M	rs80019988	22	34097658	16	1.52143693387141e-07	10.0623306	intergenic	LARGE1;LINC02885	dist=174835;dist=659009			
3M	rs541680196	22	40132086	5	1.06912975962765e-06	14.3867808	intronic	TNRC6B	.			1
3M	rs185139807	22	40198777	5	1.02186831937043e-06	14.4330526	intronic	TNRC6B	.			
3M	rs141127122	22	40208435	4	3.1593298841889e-06	15.3824875	intronic	TNRC6B	.			
3M	rs148998974	22	40224526	5	8.65242968473446e-07	14.5129532	intronic	TNRC6B	.			
3M	rs555040883	22	40235472	4	3.05234476142884e-06	15.4273069	intronic	TNRC6B	.			
3M	rs182959028	22	45427152	7	2.37697466245549e-06	11.944461	intronic	RIBC2	.			1
3M	rs150946694	22	46457283	4	3.3001729159907e-06	15.3082479	intronic	CELSR1	.			1

Combined Results				
GENOME-WIDE RISK LOCI	2	24		
SUGGESTIVE RISK LOCI (not genome-wide)			74	200
SUGGESTIVE RISK LOCI (including genome-wide)			76	224
TOTAL GENOME-WIDE & SUGGESTIVE RISK LOCI				274
MERGED (Sum minus overlap)		26		263

Overlapping Risk Loci

ALK, DCANP1, JAG2, KCND3, PPARGC1A, STAG1, TBLXR1	OVERLAPPING RISK LOCI	7
AGTR1, CDH2, ETAA1, MTX2, SGCG		4

GENOME-WIDE SIGNIFICANCE SNPS	45
SUGGESTIVE SNPS	498
TOTAL SNPS	543
SNPS REPEATED 12&3 MO	11
Risk loci that cluster these 11 SNPs	7
