# Supplementary material for: Pharmacogenomics of steroid-induced ocular hypertension: relationship to high-tension glaucomas and new pathophysiologic insight
Source: medRxiv. 2025 Aug 13:2025.08.11.25333245. Preprint. [Version 1] doi: 10.1101/2025.08.11.25333245 (PMC12363710; doi:10.1101/2025.08.11.25333245)
Supplement: Supplement 4 — Table S3. Co-localization with Other High-Tension Ocular Phenotypes [file media-4.pdf]

Supplementary Table S3. Co-localization with Other High-Tension Ocular Phenotypes

Abbreviation or Acronym		Reference number
POAG MA	primary open angle glaucoma, multi-ancestry	Han et al. (72); Lo Faro et al. (83)
POAG AF	primary open angle glaucoma, African ancestry	Verma et al. (73)
PACG	primary angle closure glaucoma	Vithana et al. (84)
PEXS/G	pseudoexfoliation syndrome/glaucoma	Zagajewska et al. (86); Krumbiegel et al. (87)
IOP	intraocular pressure	Han et al. (72)
CCT	central corneal thickness	Iglesias et al. (74); Choquet et al. (77); Igo et al. (162)
Stickler	Type II Stickler Syndrome	

Footnotes

*subthreshold P value

**protective effect

Count

30.77%	risk loci containing SNPs of genome-wide significance
18.70%	total risk loci

Gene	P value for top SNP of genome-wide significance in discovery cohort	High-tension ocular phenotype					Endophenotype	
		POAG MA	POAG AF	PACG	PEXS/G	Stickler	IOP	CCT
ADGRL3		X	*X				*X	
AHCTF1			*X					
ALK		X					X	
ANO4			*X					
AP1AR			*X					
ARAP2			*X					
ARHGAP21		X	*X					
ATXN1								X
B4GALNT3			*X					
BEND7	1.19E-09		*X					
CAV2		X	*X				X	
CCSER1			*X					
CFTR		X					X	
CNTNAP2					X			
CNTN4			*X					
CNTN5			*X					
COL11A1	3.65E-09	X		X	*X	X	X	
CRPPA			*X					
CSMD1	1.07E-08		*X					
EIF2AK4			*X					
EYA2		X						
FST	1.40E-08							X
GMNC			*X					
HMCN1		X						
INTU		X					*X	
ITPR2		X						
KCNIP4			*X					
KLF5		X					X	
LARGE1			*X					
MACROD2			*X					
MAML3			*X					
MVB12B		X					X	
NALCN			*X					
NCAM2			*X					
NIBAN1			*X					
NRXN1			*X					
PLXDC2		X	*X				X	
PPM1H	2.51E-08		*X					
RBFOX1	3.60E-08		*X		**X			
RBFOX3			*X					
SCHIP1			*X					
SGCG	1.84E-08							X
SPRED2		X					X	
STAG1								X
RASSF3			*X					
TFEC		X						
THSD7A		X	*X				X	
TNARC6B			*X					
TRIB2		X					X	
WWC1	3.96E-09		*X					
