# Supplementary material for: Pharmacogenomics of steroid-induced ocular hypertension: relationship to high-tension glaucomas and new pathophysiologic insight
Source: medRxiv. 2025 Aug 13:2025.08.11.25333245. Preprint. [Version 1] doi: 10.1101/2025.08.11.25333245 (PMC12363710; doi:10.1101/2025.08.11.25333245)
Supplement: Supplement 5 — Table S4. Target Gene Prioritization [file media-5.pdf]

Supplementary Table S4. Target Gene Prioritization
SNP-associated genes that are aqueous outflow pathway-expressed

NOTES

Top SNPs identified with the 12M and 3M QTs are merged, then clustered into risk loci by sorting by chromosomal position, then by chromosome
SNPs duplicated between 12 month and 3 month QT are boxed. Loci that cluster SNPs from both the 12 and 3 month QTs are boxed and highlighted in blue
SNPs of genome-wide significance are highlighted in gray (column rsid) as are their p-values (column Score.pval)
Risk loci into which multiple SNPs cluster are boxed (column Gene.refGene) and those containing SNPs of genome-wide significance are shaded in gray

HEADERS

QT: quantitative trait; rsid: reference SNP cluster ID; chr: chromosome; pos_38: position of SNP on GRCh38 reference panel; Score.pval: p-value; Est: effect size (mm Hg);
Func.refGene: SNP location with respect to nearest gene(s); Gene.refGene: nearest gene upstream and downstream; GeneDetail.refGene: distance to nearest gene upstream and downstream;
Gene.refGene AOPe: Gene.refGenes that are aqueous outflow pathway (AOP)-expressed (as viewed on Spectacle);
up;downstream AOPe: next closest aqueous outflow pathway expressed gene(s) located upstream and downstream of the closest gene(s) were not AOPe;
e/sQTL AOPe: target gene(s) of an eQTL or sQTL if also AOPe; Count GC-regulated genes: columns adding up all AOPe genes for which evidence was found that they are GC-regulated, totals at bottom;
GC-DEG TM cells: glucocorticoid-regulated gene in TM cell dataset; GC-Regulated in silico: column adding up all glucocorticoid-regulated gene in DAVID dataset, total at bottom
Count new: Genes in DAVID dataset not already identified in TM cell dataset; Count overlap: column adding up all glucocorticoid-regulated genes in both TM cell and DAVID dataset, total at bottom

QT	rsid	chr	pos_38	Score.pval	Est	Func.refGene	Gene.refGene	GeneDetail.refGene	Gene.refGene AOP-expressed	up;downstream AOP-expressed
3M	rs115348382	1	9595845	2.20695702237544e-06	18.2107495	intronic	TMEM201	.	TMEM201	
3M	rs186532456	1	18621834	9.60875608166652e-07	20.1730343	intergenic	KLHDC7A;PAX7	dist=135848;dist=9012	KLHDC7A	
3M	rs562032622	1	18632839	1.12975575994446e-06	21.0267115	intronic	PAX7	.		
12M	rs35145334	1	23138629	2.09882278975154e-06	10.6552656	intronic	LUZP1	.	LUZP1	
3M	rs2365739	1	62018790	1.81260643542989e-06	6.86815917	intronic	PATJ	.		TM2D1;L1TD1
12M	rs17127656	1	65477788	1.63996682822861e-06	5.07046538	intronic	LEPR	.	LEPR	
12M	rs7518849	1	65483108	1.67668772877101e-06	5.07456956	intronic	LEPR	.		
12M	rs11579567	1	65491458	2.1300143111827e-06	5.02973793	intronic	LEPR	.		
12M	rs7534177	1	65500037	2.85450525546462e-06	4.96123378	intronic	LEPR	.		
3M	rs149493615	1	79414404	1.38822331670427e-06	11.750632	intergenic	ADGRL4;LINC01781	dist=407674;dist=1121351	ADGRL4	
3M	rs143811231	1	79502446	1.738654939087e-06	11.5747409	intergenic	ADGRL4;LINC01781	dist=495716;dist=1033309		
12M	rs139390630	1	84400513	2.86874207705929e-06	11.6975971	intronic	DNASE2B	.	DNASE2B	
12M	rs114067899	1	84725361	2.17218533160816e-06	13.7760673	intergenic	SSX2IP;LPAR3	dist=34893;dist=86241	SSX2IP;LPAR3	
3M	rs34270375	1	88905019	9.34314238699532e-07	7.71147017	intergenic	GTF2B;KYAT3	dist=13452;dist=30754	GTF2B	
3M	rs187518659	1	98990189	2.15952482477188e-06	11.6819623	intronic	PLPPR5	.	PLPPR5	
3M	rs140420703	1	102337928	1.91423564672595e-06	16.487654	intergenic	OLFM3;COL11A1	dist=340694;dist=538539	OLFM3;COL11A1	
3M	rs563167766	1	102400809	4.72618849175033e-06	19.415739	intergenic	OLFM3;COL11A1	dist=403575;dist=475658		
3M	rs77180278	1	102496326	9.33877360643494e-08	7.33619996	intergenic	OLFM3;COL11A1	dist=499092;dist=380141		
3M	rs112351653	1	102754804	2.54962936189828e-08	7.42386947	intergenic	OLFM3;COL11A1	dist=757570;dist=121663		
3M	rs180926150	1	102760770	3.76640865110522e-06	19.641918	intergenic	OLFM3;COL11A1	dist=763536;dist=115697		
3M	rs114413507	1	102953612	2.57350301189632e-08	7.42216029	intronic	COL11A1	.		
3M	rs116672066	1	103007360	4.45932234807969e-09	8.04769476	intronic	COL11A1	.		
3M	rs111928960	1	103168079	3.65396910662882e-09	8.60257208	intergenic	COL11A1;LOC101928436	dist=59557;dist=325967		
3M	rs113221952	1	103288418	9.173975220783e-07	7.92350704	intergenic	COL11A1;LOC101928436	dist=179896;dist=205628		
3M	rs1856085	1	103571923	4.63628412408432e-07	21.51144	intronic	AMY2B	.		
3M	rs143597860	1	103614521	4.52300265269257e-07	21.5748154	intergenic	AMY2B;AMY2A	dist=34987;dist=2811	AMY2B;AMY2A	
3M	rs144541665	1	103768107	3.71844012409788e-07	21.8734611	intergenic	AMY1C;LOC100129138	dist=9415;dist=304916		
3M	rs76098744	1	111806750	1.19916376071606e-06	8.38612452	intronic	KCND3	.	KCND3	
12M	rs76098744	1	111806750	2.92647184366499E-06	9.5685657	intronic	KCND3	.		
3M	rs74683551	1	111811796	1.2121497054257e-06	8.38252043	intronic	KCND3	.		
12M	rs74683551	1	111811796	3.08074322019196E-06	9.54722832	intronic	KCND3			
3M	rs76617932	1	180961288	2.78866978177701e-06	10.5449608	intergenic	KIAA1614;STX6	dist=6401;dist=11426	KIAA1614;STX6	
3M	rs183180157	1	181277985	2.7248136592675E-06	12.61386	intergenic	LINC01699;CACNA1E	dist=39381;dist=205532		
3M	rs375790303	1	184561348	4.76779314562933e-06	11.4240393	intronic	C1orf21	.	C1orf21	
3M	rs138480898	1	184986525	3.67316732025395e-06	16.9873974	intergenic	NIBAN1;LINC01633	dist=12017;dist=15002		EDEM3;RNF2
3M	rs145766563	1	185160370	1.9043145410504e-06	16.8682102	intronic	SWT1	.	SWT1	
3M	rs147032554	1	186179732	6.67557909700165e-07	18.162609	intronic	HMCN1	.	HMCN1	
3M	rs180989936	1	193075048	3.47790847768381E-06	17.1793579	intronic	RO60	.		
12M	rs145804766	1	202153371	1.43417032447652e-06	12.5864465	intronic	PTPN7	.	PTPN7	
3M	rs10494861	1	205362746	2.48126631931009e-06	19.1820498	intergenic	KLHDC8A;LEMD1	dist=5656;dist=10506	KLHDC8A;LEMD1	
12M	rs147869916	1	227119951	3.36060694276516e-06	17.5964551	intronic	CDC42BPA	.	CDC42BPA	
3M	rs2274996	1	229668791	6.73601821695854e-07	5.58527433	intergenic	URB2;LINC01682	dist=8591;dist=206759	URB2	
3M	rs2274997	1	229668899	6.64244186267036e-07	5.58731228	intergenic	URB2;LINC01682	dist=8699;dist=206651		
3M	rs2891865	1	229670621	6.80112526815562e-07	5.58181376	intergenic	URB2;LINC01682	dist=10421;dist=204929		
3M	rs2385790	1	229671745	6.81207031237396e-07	5.58224822	intergenic	URB2;LINC01682	dist=11545;dist=203805		
3M	rs12024557	1	229676610	6.90833131537333e-07	5.56930969	intergenic	URB2;LINC01682	dist=16410;dist=198940		
3M	rs4562666	1	229689023	7.35045143592774e-07	5.51922599	intergenic	URB2;LINC01682	dist=28823;dist=186527		
3M	rs12036586	1	229690631	1.08649183321501e-06	5.22212176	intergenic	URB2;LINC01682	dist=30431;dist=184919		
3M	rs16850124	1	229695584	2.19845550423288e-06	5.18095662	intergenic	URB2;LINC01682	dist=35384;dist=179966		
3M	rs12045643	1	229698303	7.37332600021845e-07	5.48149152	intergenic	URB2;LINC01682	dist=38103;dist=177247		
3M	rs559559983	1	246813830	1.8701710647004e-06	13.811132	intergenic	LINC01341;AHCTF1	dist=22344;dist=25268	AHCTF1	
3M	rs550763536	2	7312216	3.04128022440287e-06	14.4871647	intergenic	LOC101929452;LOC100506274	dist=234336;dist=109045		RNF144A;ID2
12M	rs76220567	2	12919819	2.07255893928856e-06	20.6828868	intergenic	TRIB2;LOC100506474	dist=177087;dist=46963	TRIB2	
3M	rs558553658	2	15542600	2.00500271573415e-07	17.1099107	intronic	NBAS	.	NBAS	
3M	rs536781978	2	29510815	2.20535447064362e-06	20.5561882	intronic	ALK	.	ALK	
12M	rs536781978	2	29510815	2.20535447064362E-06	20.5561882	intronic	ALK			
3M	rs568321148	2	29647482	2.03643401922156e-06	20.7400203	intronic	ALK	.		
12M	rs568321148	2	29647482	2.03643401922156E-06	20.7400203	intronic	ALK			
12M	rs186792608	2	42869559	1.57557414570447e-07	22.4512499	intergenic	HAAO;LINC01819	dist=76976;dist=158293	HAAO	
3M	rs76777840	2	48085811	7.02623491851785e-07	14.3500784	intergenic	FBXO11;FOXN2	dist=179313;dist=228488	FBXO11;FOXN2	
3M	rs145080832	2	48246004	1.37120287877638e-07	17.1618477	intergenic	FBXO11;FOXN2	dist=339506;dist=68295		
3M	rs184220112	2	48404604	3.11727344916156e-07	16.4879043	intergenic	FOXN2;PPP1R21	dist=25309;dist=36162	FOXN2;PPP1R21	

3M	rs189890455	2	48428258	4.1971806049668e-07	15.1467391	intergenic	FOXN2;PPP1R21	dist=48963;dist=12508		
3M	rs181193202	2	52300216	1.62222587830176e-07	19.6312088	ncRNA_intronic	LOC730100	.		NRXN1;ASB3
3M	rs183378658	2	52336233	1.16662586966659e-07	19.7446169	ncRNA_intronic	LOC730100	.		
3M	rs190193113	2	52858640	1.13301212531008e-07	19.9533602	intergenic	MIR4431;ASB3	dist=156025;dist=811339		
3M	rs187520610	2	53132903	3.93234514941546e-09	18.8010396	intergenic	MIR4431;ASB3	dist=430288;dist=537076	ASB3	
3M	rs149421869	2	53256291	2.05046156638639e-06	11.7382186	intergenic	MIR4431;ASB3	dist=553676;dist=413688		
3M	rs146479102	2	65598625	2.18341304839895e-06	15.4846768	intergenic	SPRED2;MIR4778	dist=166026;dist=759622	SPRED2	
3M	rs528288879	2	65648796	5.421960882917e-07	14.7878085	intergenic	SPRED2;MIR4778	dist=216197;dist=709451		
3M	rs11690187	2	67338777	4.61292753502872e-06	13.6310532	intergenic	LINC01828;ETAA1	dist=49533;dist=58556	ETAA1	
3M	rs151272830	2	67455592	2.85197668301622e-06	13.3323479	intergenic	ETAA1;LINC01812	dist=43503;dist=340462		
3M	rs186142189	2	67475575	2.69645566407293e-06	12.7361927	intergenic	ETAA1;LINC01812	dist=63486;dist=320479		
12M	rs2902021	2	67598553	3.30567771883221e-06	4.55991411	intergenic	ETAA1;LINC01812	dist=186464;dist=197501		
12M	rs75082290	2	67604021	1.699715195267e-06	4.69395387	intergenic	ETAA1;LINC01812	dist=191932;dist=192033		
12M	rs191298981	2	67882914	7.69651662192921e-07	24.0078714	intergenic	LINC01812;C1D	dist=57352;dist=158216	C1D	
12M	rs113537164	2	68007798	1.017020860936e-06	20.3890413	intergenic	LINC01812;C1D	dist=182236;dist=33332		
12M	rs113154814	2	68062365	1.66384008676077e-07	19.8310188	intronic	C1D	.		
3M	rs184200893	2	69033781	2.24020739771759E-06	19.9580356	intronic	ANTXR1	.		
3M	rs111927235	2	74256827	8.4250114725101e-07	17.0468955	intronic	SLC4A5	.	SLC4A5	
3M	rs111838310	2	74446364	3.26261179053175e-07	17.7963938	intergenic	RTKN;INO80B-WBP1	dist=4427;dist=8659	RTKN	
3M	rs112983626	2	74470023	3.34719559082237e-07	17.7848079	intergenic	MOGS;MRPL53	dist=4641;dist=1959	MOGS	
3M	rs113006316	2	74575233	3.34707225210704e-06	22.8886792	intronic	M1AP	.	M1AP	
3M	rs17746486	2	95056864	1.47154878644706e-06	6.90851358	intergenic	MAL;MRPS5	dist=2872;dist=28507	MAL;MRPS5	
3M	rs76554191	2	95301880	3.87215481208204e-06	5.72148039	intronic	KCNIP3	.	KCNIP3	
3M	rs140352232	2	107421656	3.8788022972212e-06	21.0576511	intergenic	MIR548AU;LINC01886	dist=72131;dist=107763		ST6GAL2;RGPD4
12M	rs146919974	2	114495286	7.91246832646777e-07	19.7280466	intronic	DPP10	.	DPP10	
3M	rs116189766	2	125636287	1.17473937061964e-06	21.190764	intergenic	CNTNAP5;LINC01941	dist=715069;dist=473813		TSN;GYPC
3M	rs139877408	2	128848699	4.57552408670287e-06	20.3067129	intergenic	HS6ST1;LOC101927881	dist=529831;dist=15901	HS6ST1	
3M	rs142894171	2	150581757	1.40524666465064e-06	18.0710394	intergenic	LINC01920;LINC02612	dist=9536;dist=47140		RND3;
3M	rs541508507	2	169166755	2.32254126928461e-06	15.4630033	intronic	LRP2	.	LRP2	
3M	rs142549310	2	169173996	2.69758962759472e-06	15.2501713	exonic	LRP2	.		
3M	rs556293455	2	176085692	1.27371002978549e-06	14.3860463	intergenic	EVX2;HOXD13	dist=1730;dist=7029	EVX2;HOXD13	
3M	rs184098071	2	176251692	1.15315934031841e-06	13.947724	intergenic	HOXD1;MTX2	dist=60785;dist=17750	MTX2	
3M	rs79935606	2	176473907	2.55448017015286e-06	16.3396833	intergenic	MTX2;MIR1246	dist=135882;dist=127073		
12M	rs532416695	2	176622062	1.32803586650991e-07	19.8726656	intergenic	MIR1246;LINC01116	dist=21010;dist=7519		
3M	rs112557251	2	187510673	3.44653364518888e-06	20.6510064	intronic	TFPI	.	TFPI	
3M	rs185158855	2	222785307	3.71819301386344e-06	16.6678864	intergenic	MOGAT1;ACSL3	dist=75377;dist=75728	MOGAT1;ACSL3	
3M	rs185510569	2	222950143	5.51379458113093e-08	19.5886906	intergenic	ACSL3;KCNE4	dist=5505;dist=102047	ACSL3;KCNE4	
3M	rs147559909	2	236142879	1.75642429015382e-08	17.0840975	intergenic	AGAP1;GBX2	dist=11086;dist=22356	AGAP1	
3M	rs181217257	2	239067423	5.29269845950619e-10	21.5677368	intronic	HDAC4	.	HDAC4	
3M	rs188076929	2	239072023	1.0296847211524e-09	19.9775614	intronic	HDAC4	.		
3M	rs112475378	3	1584977	1.34664824685029e-06	9.55085929	intergenic	CNTN6;CNTN4	dist=180760;dist=513826	CNTN6;CNTN4	
3M	rs145676540	3	2002951	1.25427052978558e-06	13.980658	intergenic	CNTN6;CNTN4	dist=598734;dist=95852		
12M	rs144788248	3	19545253	3.87625815195203e-06	11.9086237	intergenic	KCNH8;EFHB	dist=9611;dist=334221	KCNH8;EFHB	
3M	rs146007933	3	28185932	1.34950762676418e-06	7.51149453	intergenic	LINC01980;CMC1	dist=325607;dist=55687	CMC1	
3M	rs73057656	3	33899079	3.43591498795888e-06	5.9238329	intergenic	PDCD6IP;LOC101928135	dist=29372;dist=976718	PDCD6IP	
3M	rs73085348	3	42669729	6.28155549798072e-07	10.139317	intergenic	ZBTB47;KLHL40	dist=2149;dist=15808	ZBTB47	
3M	rs142684595	3	55285273	7.16787983460343e-08	16.7729431	intergenic	LINC02017;WNT5A	dist=96059;dist=180442	WNT5A	
12M	rs149706477	3	66162553	2.87948941747911e-06	20.9828687	intronic	SLC25A26	.	SLC25A26	
3M	rs80203220	3	123003484	2.31429533714517e-06	13.426042	intronic	SEMA5B	.	SEMA5B	
12M	rs114280794	3	133123359	3.81823609520308e-06	8.76296877	intronic	TMEM108	.	TMEM108	
3M	rs192443987	3	135813503	1.3640761999881e-06	17.7540285	intergenic	EPHB1;PPP2R3A	dist=553038;dist=152225	EPHB1;PPP2R3A	
3M	rs148248743	3	136415753	7.68859185089755e-07	22.839379	intronic	STAG1	.	STAG1	
12M	rs148248743	3	136415753	6.68816287673531E-07	27.1548587	intronic	STAG1	.		
3M	rs576124203	3	142122354	4.46547319819514e-07	19.4039925	intronic	TFDP2	.	TFDP2	
3M	rs545552231	3	142145508	4.79811814113124e-07	19.8641332	intronic	TFDP2	.		
12M	rs138138661	3	147940027	5.4928185119564e-08	23.1347457	intergenic	LOC440982;LINC02032	dist=430117;dist=138132		
12M	rs189234695	3	147940371	3.47705303907978e-06	23.9697134	intergenic	LOC440982;LINC02032	dist=430461;dist=137788		
12M	rs148997617	3	148037789	1.05449927842458e-07	20.8774277	intergenic	LOC440982;LINC02032	dist=527879;dist=40370		
3M	rs193153124	3	148612923	2.77507554510464e-06	14.3657183	intergenic	LINC02046;AGTR1	dist=212967;dist=84948	AGTR1	
3M	rs188720948	3	150352093	8.59439366670513e-07	17.7032653	intergenic	LINC01214;TSC22D2	dist=28346;dist=56205	TSC22D2	
3M	rs16823323	3	153939413	3.35704338797875e-08	9.48464065	intergenic	LINC02006;ARHGEF26	dist=176887;dist=84988	ARHGEF26	
3M	rs139943877	3	155733500	2.25796218142194e-06	13.0535902	intronic	PLCH1	.	PLCH1	
12M	rs148483098	3	157664262	3.27691859100501e-06	15.7357844	intergenic	SLC66A1L;SHOX2	dist=63168;dist=431643	SHOX2	
12M	rs79252854	3	157805368	2.4780397246083e-06	17.5464336	intergenic	SLC66A1L;SHOX2	dist=204274;dist=290537		
12M	rs139055031	3	158869060	3.17888515002842e-06	16.2853761	intergenic	MFSD1;IQCJ	dist=39341;dist=200192	MFSD1	
12M	rs143669489	3	159675096	4.93900179409246e-06	24.2253199	intronic	IQCJ-SCHIP1;SCHIP1	.	IQCJ-SCHIP1;SCHIP1	
3M	rs187047882	3	164567833	3.41360195160902e-06	18.5641361	intergenic	MIR1263;LINC01324	dist=396277;dist=146262		
3M	rs141169929	3	165090674	2.90582805983588e-06	14.8454178	intergenic	SI;SLITRK3	dist=12178;dist=96046	SLITRK3	
3M	rs186649043	3	175214503	2.29531002487815e-06	19.2134209	intronic	NAALADL2	.	NAALADL2	
3M	rs189709453	3	177856520	1.55548473782376e-06	23.2009936	ncRNA_intronic	LINC02015	.		TBL1XR1;ZMAT3
12M	rs189709453	3	177856520	1.55548473782376E-06	23.2009936	ncRNA_intronic	LINC02015	.		
3M	rs186767531	3	177921989	1.62563417403378E-07	21.2013243	intergenic	LINC02015;LINC01014	dist=22765;dist=497212		
12M	rs186767531	3	177921989	1.66219440747609E-06	23.0749977	intergenic	LINC02015;LINC01014	dist=22765;dist=497212		
3M	rs182868205	3	177994660	1.36806623320862e-06	22.8544303	intergenic	LINC02015;LINC01014	dist=95436;dist=424541		
12M	rs182868205	3	177994660	1.36806623320862E-06	22.8544303	intergenic	LINC02015;LINC01014	dist=95436;dist=424541		
12M	rs76356799	3	179875980	2.9143183363234e-07	19.2867007	intronic	PEX5L	.	PEX5L	
12M	rs117591241	3	190866890	4.37648501942309e-06	19.9014028	intergenic	GMNC;SNAR-I	dist=4196;dist=11040	GMNC	
3M	rs191792521	3	195919734	2.52243498533689e-06	13.9515484	intergenic	TNK2;SDHAP1	dist=6470;dist=40187	TNK2	
3M	rs113651406	4	997179	2.52148284014823e-07	19.4054026	intronic	IDUA	.	IDUA	
3M	rs12502861	4	2424578	2.48774370313747e-06	11.1588472	intronic	CFAP99	.	CFAP99	
3M	rs189765693	4	4323066	2.55310143124462e-06	15.5191341	intergenic	ZBTB49;NSG1	dist=1283;dist=63466	ZBTB49;NSG1	
12M	rs189094663	4	11621826	2.89927274380224e-06	23.895416	intergenic	HS3ST1;LINC02360	dist=192932;dist=119125	HS3ST1	
12M	rs557276277	4	19012075	3.35996743882432e-06	19.0146344	intergenic	LCORL;SLIT2	dist=990200;dist=1239830	LCORL;SLIT2	

3M	rs183962155	4	21258020	3.64461882511902e-06	13.7646008	intronic	KCNIP4	.	KCNIP4	
3M	rs113751774	4	23427231	5.00531306271799e-07	14.9103274	intergenic	GBA3;PPARGC1A	dist=607659;dist=364790		
3M	rs113063005	4	23507444	1.79978248668523e-11	21.2073894	intergenic	GBA3;PPARGC1A	dist=687872;dist=284577	PPARGC1A	
12M	rs113063005	4	23507444	9.56810303773154E-07	16.61763	intergenic	GBA3;PPARGC1A			
3M	rs145875128	4	32071514	1.07037949708586e-07	24.479986	ncRNA_intronic	LINC02506	.	ARAP2	
3M	rs143287889	4	35570658	4.29676290263636e-06	19.96741	intergenic	LINC02484;ARAP2	dist=1300911;dist=495346		
3M	rs77141817	4	37052137	1.52230770368993e-08	20.6110292	intergenic	LINC02616;MIR4801	dist=31431;dist=189773		;C4orf19,RELL1
3M	rs190822761	4	37097734	1.6069016808543e-08	20.5846354	intergenic	LINC02616;MIR4801	dist=77028;dist=144176		
12M	rs147267707	4	43024541	2.25472034099615e-06	18.0094385	intronic	GRXCR1	.		ATP8A1;KCTD8
12M	rs77618729	4	43048951	2.25749765325168e-06	18.0130153	intergenic	GRXCR1;LINC02383	dist=18293;dist=408583		
12M	rs556435089	4	43549016	2.61570608345655e-06	17.939079	intergenic	LINC02383;LINC02475	dist=56473;dist=467845		
12M	rs532464521	4	43569022	2.45873915171385e-06	17.9895528	intergenic	LINC02383;LINC02475	dist=76479;dist=447839		
3M	rs999769259	4	61646247	3.61294355616724e-06	22.1246725	intronic	ADGRL3	.	ADGRL3	
3M	rs147630370	4	86529522	7.46814493303135e-07	16.0013633	intergenic	MAPK10;MIR4452	dist=76327;dist=12960	MAPK10	
3M	rs147171192	4	88214436	4.39967162056646e-06	13.3044047	intronic	ABCG2	.	ABCG2	
3M	rs142993106	4	90036221	3.08700493831608e-06	8.08463611	intergenic	MMRN1;CCSER1	dist=81611;dist=91173	MMRN1;CCSER1	
3M	rs146526206	4	90071867	1.81582969378392e-06	8.09176656	intergenic	MMRN1;CCSER1	dist=117257;dist=55527		
3M	rs17017794	4	90904734	3.46919898165651e-07	5.83240545	intronic	CCSER1	.		
3M	rs145116559	4	111756440	4.90673849934313e-06	18.2441224	intergenic	MIR297;FAM241A	dist=895793;dist=389014		PITX2;AP1AR
3M	rs181415102	4	111768110	4.81312717634891e-06	18.2631019	intergenic	MIR297;FAM241A	dist=907463;dist=377344		
3M	rs191423619	4	125595472	2.50684302750887e-07	21.694611	intergenic	MIR2054;INTU	dist=88165;dist=2037485	INTU	
3M	rs149298750	4	126237546	1.98725449883812e-07	22.184052	intergenic	MIR2054;INTU	dist=730239;dist=1395411		
12M	rs181132315	4	128726214	1.68603480568751e-06	22.1324679	intergenic	LINC02615;JADE1	dist=206818;dist=83486	JADE1	
3M	rs112679237	4	138219706	2.04053672228041e-07	8.71202752	intronic	SLC7A11	.	SLC7A11	
12M	rs147455971	4	140010558	3.87240564796723e-06	17.2160142	intronic	MAML3	.	MAML3	
3M	rs531769270	4	153489891	2.4145887031028e-06	16.5945338	intronic	TMEM131L	.		MND1;TLR2
3M	rs116651654	4	162317591	3.48829177722726e-07	15.6223694	intergenic	FSTL5;MIR4454	dist=153557;dist=775983	FSTL5	
12M	rs17615362	4	169012936	4.30304118178725e-06	5.41974472	intergenic	CBR4;SH3RF1	dist=2681;dist=81323	CBR4;SH3RF1	
12M	rs17543620	4	169013574	4.21133121953689e-06	5.42796755	intergenic	CBR4;SH3RF1	dist=3319;dist=80685		
3M	rs567982164	5	25631188	5.7310337989489e-07	21.8356286	intergenic	LINC02211;CDH9	dist=328908;dist=1249409	CDH9	
3M	rs191986449	5	25745578	4.59857674938651e-06	18.4978793	intergenic	LINC02211;CDH9	dist=443298;dist=1135019		
12M	rs142934021	5	53366842	1.40182538744286e-08	18.824715	intergenic	LOC257396;FST	dist=251716;dist=113787	FST	
3M	rs185771987	5	73989664	4.53395085864185e-06	18.2344824	intergenic	ARHGEF28;LINC01335	dist=47671;dist=316746	ARHGEF28	
3M	rs139360368	5	74076284	1.24776973080952e-06	18.3590487	intergenic	ARHGEF28;LINC01335	dist=134291;dist=230126		
3M	rs181933850	5	92169830	3.33393070013435e-06	11.0436586	intergenic	ARRDC3;NR2F1	dist=749115;dist=1279415	ARRDC3;NR2F1	
3M	rs190190051	5	92179668	3.47677941958718e-06	11.0589887	intergenic	ARRDC3;NR2F1	dist=758953;dist=1269577		
3M	rs182531466	5	92234256	4.23245053141191e-06	12.6804978	intergenic	ARRDC3;NR2F1	dist=813541;dist=1214989		
3M	rs187236873	5	92234630	1.97926852689169e-06	12.44907	intergenic	ARRDC3;NR2F1	dist=813915;dist=1214615		
3M	rs183816745	5	92336071	6.02067560021534e-08	17.3487909	intergenic	ARRDC3;NR2F1	dist=915356;dist=1113174		
3M	rs111407636	5	95744325	6.27809322802765e-07	18.642774	intronic	RHOBTB3	.	RHOBTB3	
3M	rs111676272	5	95758594	6.71649637293761e-07	18.6068625	intronic	RHOBTB3	.		
3M	rs75848314	5	95762636	1.11087241977704e-07	20.0998481	intronic	RHOBTB3	.		
3M	rs111846247	5	95775939	1.06810112370498e-07	20.4865772	intronic	RHOBTB3	.		
3M	rs137873790	5	97751337	4.51630701305597e-06	9.22240881	intergenic	LINC01340;LINC02234	dist=80286;dist=89421		RIOK2;RGMB
3M	rs189912648	5	135429749	2.55441427995549e-06	16.9732599	intergenic	MACROH2A1;DCANP1	dist=29862;dist=14465	MACROH2A1;DCANP1	
12M	rs189912648	5	135429749	3.32990944802665E-06	19.8604449	intergenic	MACROH2A1;DCANP1	dist=29862;dist=14465		
12M	rs111512950	5	154300867	4.11671051184833e-06	13.567443	intronic	GALNT10	.	GALNT10	
3M	rs191006910	5	154836449	3.35533057793161e-06	17.6173803	intronic	FAXDC2	.	FAXDC2	
3M	rs74343174	5	162066176	2.54836335504837e-06	14.7671824	intergenic	LINC01202;GABRG2	dist=64980;dist=1289	GABRG2	
3M	rs775626702	5	163203061	3.23411085027866e-06	18.9484137	intergenic	GABRG2;CCNG1	dist=1047522;dist=234510	GABRG2;CCNG1	
3M	rs371245624	5	163453950	1.65804063735814e-06	19.4795931	UTR3	NUDCD2	NM_001329991:c.*170>0;NM_	NUDCD2	
3M	rs290120	5	163841238	2.40444258455957e-06	13.3587764	intergenic	MAT2B;LINC02143	dist=321884;dist=607184	MAT2B	
3M	rs545428520	5	168394261	3.96137253437646e-09	21.566035	intronic	WWC1	.	WWC1	
3M	rs528404963	5	168425020	7.1738299793335e-09	21.1393977	intronic	WWC1	.		
3M	rs72832764	5	170577669	2.69939738977966e-06	18.2159757	intronic	KCNIP1	.	KCNIP1	
3M	rs72837643	5	170761951	4.147895992623e-06	16.9302874	intergenic	KCNIP1;GABRP	dist=25319;dist=21768	GABRP	
3M	rs142311947	5	177957468	2.99420386474277e-06	12.0339013	ncRNA_intronic	LOC100128340	.		FAM153A;FAM153C
3M	rs151015676	5	177963936	4.05781112465234e-06	20.1421367	intergenic	LOC100128340;PROP1	dist=4136;dist=28299		
3M	rs1877768	6	16534692	2.77630022916084e-06	8.28754685	intronic	ATXN1	.	ATXN1	
12M	rs73384619	6	20457948	3.65476878017977e-06	8.3916702	intronic	E2F3	.	E2F3	
3M	rs150586237	6	24491120	5.5145390836127e-09	22.0461443	intergenic	GPLD1;ALDH5A1	dist=1542;dist=3849	GPLD1;ALDH5A1	
3M	rs571986619	6	84969114	1.70115854486702e-06	20.7858067	intergenic	TBX18;LINC02535	dist=204516;dist=418105	TBX18	
3M	rs56224400	6	97644799	9.49951178557744e-07	8.4433064	ncRNA_intronic	LOC101927314	.		MMS22L;POU3F2
3M	rs147627638	6	98725240	2.84737109324869e-06	13.0785306	intergenic	MIR2113;PNKY	dist=700621;dist=104901		POU3F2
3M	rs141326851	6	134511989	2.157242978017e-06	8.57849688	intergenic	LINC01010;LOC101928304	dist=7969;dist=13329		SGK1;ALDH8A1
3M	rs146048121	6	141877818	3.23639603147815e-06	20.0214379	intergenic	MIR4465;NMBR	dist=1193935;dist=196666		
3M	rs142106992	6	141948748	5.23067431501407e-10	22.8345886	intergenic	MIR4465;NMBR	dist=1264865;dist=125736		
3M	rs72983831	6	141985963	7.82545989102806e-07	15.6733239	intergenic	MIR4465;NMBR	dist=1302080;dist=88521		
3M	rs72986533	6	142290121	1.53596722070896e-07	13.2812726	intergenic	VTA1;ADGRG6	dist=65437;dist=11798	VTA1;AGRG6	
3M	rs73586304	6	142518288	4.95566451287885e-06	18.2405116	intergenic	ADGRG6;LOC153910	dist=72022;dist=8167		
12M	rs4896997	6	148362788	3.04539162547005e-06	14.4320923	intronic	SASH1	.		
12M	rs4131286	6	148367827	3.05925201460773e-06	14.4284672	intronic	SASH1	.	SASH1	
12M	rs17078283	6	148375784	3.05230865558584e-06	14.4302821	intronic	SASH1	.		
12M	rs56821264	6	148382765	2.17257084724272e-06	14.7509213	intronic	SASH1	.		
3M	rs148532212	6	165086368	1.8149830669263e-07	19.3535457	intergenic	MEAT6;C6orf118	dist=264305;dist=193296	C6orf118	
3M	rs117498042	6	165100792	1.85878685547635e-07	19.335471	intergenic	MEAT6;C6orf118	dist=278729;dist=178872		
3M	rs148153037	6	167087898	3.20376100252789e-09	14.3359246	intergenic	CEP43;CCR6	dist=35180;dist=23909	CCR6	
3M	rs184487573	6	167099983	1.91568282565546e-07	13.5009635	intergenic	CEP43;CCR6	dist=47265;dist=11824		
3M	rs17776100	7	6386848	5.13308111425872e-07	6.63697397	intronic	RAC1	.	RAC1	
3M	rs187978759	7	11672218	2.76447739355703e-06	20.4555522	intronic	THSD7A	.	THSD7A	
12M	rs143686474	7	16252374	4.88904023817646e-06	10.8531498	ncRNA_intronic	CRPPA	.		MEOX2;SOSTDC1
3M	rs117166500	7	17013154	1.89815826168549e-06	12.5146865	intergenic	AGR3;AHR	dist=131171;dist=285498	AGR3;AHR	

3M	rs75689761	7	18366950	9.11790229535791e-07	11.8482917	intronic	HDAC9	.		
3M	rs77346868	7	18366976	1.13767941006556e-06	11.0210129	intronic	HDAC9	.		
3M	rs78225611	7	18367841	1.13375019479557e-06	11.0294387	intronic	HDAC9	.		
3M	rs77300464	7	18369138	2.09191655072049e-08	14.6659794	intronic	HDAC9	.	HDAC9	
3M	rs79602997	7	18370627	9.43076371109133e-07	11.8608211	intronic	HDAC9	.		
3M	rs75773869	7	18371222	9.36316662267224e-07	11.8642716	intronic	HDAC9	.		
3M	rs75606013	7	18374990	2.11098533218711e-08	14.6617119	intronic	HDAC9	.		
3M	rs61434999	7	18378728	9.22178787748122e-07	11.8411511	intronic	HDAC9	.		
3M	rs78907958	7	18385394	4.98811901973441e-07	12.0901322	intronic	HDAC9	.		
3M	rs76526501	7	18391487	8.57654056311165e-09	14.8397312	intronic	HDAC9	.		
3M	rs74455595	7	18392161	8.57654056311165e-09	14.8397312	intronic	HDAC9	.		
3M	rs79182806	7	18394204	4.13732512224196e-07	12.0972603	intronic	HDAC9	.		
3M	rs10279777	7	18401966	1.65309369490778e-08	12.8681335	intronic	HDAC9	.		
3M	rs77867199	7	18402652	3.93328414300688e-07	10.8221282	intronic	HDAC9	.		
3M	rs80156375	7	18403592	3.27106315853648e-07	11.5406255	intronic	HDAC9	.		
3M	rs17169602	7	18407118	1.78410513894506e-08	12.7273717	intronic	HDAC9	.		
3M	rs10486295	7	18407184	1.75006169477667e-08	12.76229	intronic	HDAC9	.		
3M	rs75090694	7	18407813	1.01013780102531e-08	13.8314005	intronic	HDAC9	.		
3M	rs55844051	7	23320744	1.93295366566624e-06	18.3875896	intronic	IGF2BP3	.	IGF2BP3	
3M	rs62447184	7	36534898	2.45196683172423e-06	5.12842209	intronic	AOAH	.		
3M	rs574076561	7	49505151	3.8458987487847e-06	19.6434766	intergenic	CDC14C;VWC2	dist=577697;dist=268487	VWC2	
3M	rs111391231	7	89867731	4.17997193738163e-06	8.31717755	intergenic	ZNF804B;STEAP2	dist=529203;dist=14622	ZNF804B;STEAP2	
3M	rs111900874	7	89887355	3.79796761664437e-06	8.42424013	ncRNA_intronic	STEAP2	.		
3M	rs181259864	7	97859511	3.26388920997597e-06	17.1360821	ncRNA_intronic	CZ1P-ASNS	.	CZ1P-ASNS	ASNS;TAC1
3M	rs192750513	7	97948518	3.9425756746568e-06	17.1892469	ncRNA_intronic	CZ1P-ASNS	.		
3M	rs539713344	7	100877165	2.02994489815685e-06	20.044756	intronic	SRRT	.	SRRT	
3M	rs188028357	7	101025144	5.38240258932335e-07	21.8425578	intronic	MUC17	.		MUC12;TRIM56
12M	rs73202425	7	109515659	3.05259237348811e-06	10.761844	intergenic	C7orf66;EIF3IP1	dist=631072;dist=443568	EIF3IP1	
12M	rs181182636	7	116014504	2.90894506747647e-06	21.9472639	intronic	TFEC	.	TFEC	
12M	rs182211730	7	116363561	1.45395858607717e-06	18.7746033	intergenic	LOC102724434;CAV2	dist=76834;dist=136177	CAV2	
12M	rs143281973	7	116367942	1.62372890091138e-06	18.2750778	intergenic	LOC102724434;CAV2	dist=81215;dist=131796		
12M	rs138873576	7	116385305	3.22380485845586e-06	17.6151711	intergenic	LOC102724434;CAV2	dist=98578;dist=114433		
12M	rs190354334	7	116435792	4.2862377340545e-06	17.3392581	intergenic	LOC102724434;CAV2	dist=149065;dist=63946		
12M	rs186893139	7	116456916	3.55104961576984e-06	18.602598	intergenic	LOC102724434;CAV2	dist=170189;dist=42822		
12M	rs558784715	7	117403240	1.5182227326648e-06	20.7591787	intronic	ASZ1	.		
12M	rs576047962	7	117468967	1.61732320840498e-06	20.7261549	intergenic	ASZ1;CFTR	dist=41474;dist=11058	CFTR	
12M	rs529110230	7	117535853	1.24629705452557e-06	20.8583633	intronic	CFTR	.		
12M	rs142215699	7	117559820	1.6210064953756e-06	20.7292287	ncRNA_intronic	CFTR	.		
12M	rs142721557	7	117578274	1.61401730397805e-06	20.7329975	intronic	CFTR	.		
12M	rs201355675	7	117585727	1.64203568676087e-06	20.718124	intronic	CFTR	.		
12M	rs188993522	7	117634677	1.9792198058251e-06	21.2984827	intronic	CFTR	.		
3M	rs536023430	7	147167980	2.63612685649085e-06	19.4038683	intronic	CNTNAP2	.	CNTNAP2	
3M	rs187384541	8	1684075	3.15110880283135e-06	6.88745117	intronic	DLGAP2	.	DLGAP2	
3M	rs575473987	8	5719972	1.07256928472273e-08	22.2248653	intergenic	CSMD1;LOC100287015	dist=725058;dist=683583	CSMD1	
3M	rs139062456	8	13394482	3.88565669511608e-06	7.83515809	intronic	DLC1	.	DLC1	
3M	rs185874707	8	18173165	2.74810255839378e-06	9.13934765	intronic	NAT1	.	NAT1	
3M	rs140797780	8	22230279	1.73425396214273e-07	19.2908396	intronic	PHYHIP	.	PHYHIP	
3M	rs147601511	8	22464806	7.14867637986866e-07	16.4471181	intronic	PPP3CC	.	PPP3CC	
3M	rs188415494	8	25755782	3.49911568838158e-06	18.5169478	intergenic	CDCA2;EBF2	dist=247865;dist=85943	CDCA2;EBF2	
3M	rs9643828	8	54616513	2.29737457097614e-07	-2.4236294	intronic	RP1	.		SOX17;XKR4
3M	rs423841	8	54643509	2.7508818251862e-06	-2.1495726	intronic	RP1	.		
3M	rs433324	8	54652049	9.22325760191311e-07	-2.2483462	intronic	RP1	.		
3M	rs369623	8	54659380	8.50610562336969e-07	-2.2359815	intronic	RP1	.		
3M	rs446222	8	54662400	7.36959451875675e-07	-2.247819	intronic	RP1	.		
3M	rs432393	8	54667738	1.22958170848882e-06	-2.1875382	intronic	RP1	.		
3M	rs3098298	8	54670278	1.22819650607779e-06	-2.1875635	intronic	RP1	.		
3M	rs367179	8	54675056	1.22819650607779e-06	-2.1875635	intronic	RP1	.		
3M	rs382476	8	54678415	7.36949131111935e-07	-2.2481938	intronic	RP1	.		
3M	rs384543	8	54679049	7.36949131111935e-07	-2.2481938	intronic	RP1	.		
3M	rs405226	8	54679776	1.2251324121268e-06	-2.1881305	intronic	RP1	.		
3M	rs384127	8	54684929	7.36964497256014e-07	-2.2481559	intronic	RP1	.		
3M	rs858397	8	54702130	7.161979275107e-07	2.2301348	intronic	RP1	.		
3M	rs2375537	8	54706948	6.2991993988762e-07	2.23876848	intronic	RP1	.		
3M	rs720372	8	54716077	2.93014534156507e-06	2.09872734	intronic	RP1	.		
3M	rs1437781	8	54717292	6.32222203401203e-07	2.23854673	intronic	RP1	.		
3M	rs1595406	8	54718055	2.80263765648492e-06	2.10018889	intronic	RP1	.		
3M	rs1437782	8	54720202	5.53724964407086e-07	2.2579585	exonic	RP1	.		
3M	rs10105693	8	54727912	3.41214459494352e-07	2.2928391	intronic	RP1	.		
3M	rs2375536	8	54728162	2.13408172799341e-06	2.12635806	intronic	RP1	.		
3M	rs4737674	8	54749094	2.86005059848221e-07	2.30559554	intronic	RP1	.		
3M	rs11987234	8	54757269	3.13190459972498e-07	2.29599259	intronic	RP1	.		
3M	rs13277510	8	54761589	2.8610568625199e-07	2.30494752	intronic	RP1	.		
3M	rs12548593	8	54762057	3.38344249342945e-07	2.28269961	intronic	RP1	.		
3M	rs1812506	8	54763541	2.00833101672028e-06	2.12258868	intronic	RP1	.		
3M	rs16920698	8	54765874	2.85868793626322e-07	2.30492695	intronic	RP1	.		
3M	rs1561297	8	54765978	3.34668102178151e-07	2.28315237	intronic	RP1	.		
3M	rs4737676	8	54766986	2.8589296796158e-07	2.30492057	intronic	RP1	.		
3M	rs2083123	8	54767758	3.4358513898515e-07	2.27910794	intronic	RP1	.		
3M	rs983248	8	54768232	2.87356270418993e-07	2.30260428	intronic	RP1	.		
3M	rs1391463	8	54769316	2.87382622216009e-07	2.30258865	intronic	RP1	.		
3M	rs10958428	8	54773081	5.0112447137886e-07	2.23996885	intronic	RP1	.		
3M	rs13278605	8	54775611	3.41581916245031e-07	2.27377348	intronic	RP1	.		
3M	rs13276543	8	54775614	3.13999361335922e-07	2.29263157	intronic	RP1	.		

3M	rs7822082	8	54777660	1.5040633792954e-07	2.3570878	intronic	RP1	.		
3M	rs4737201	8	54778898	2.89096388651053e-07	2.30197734	intronic	RP1	.		
3M	rs7843693	8	54779552	4.78812399757755e-06	1.99856756	intronic	RP1	.		
3M	rs1396896	8	54782750	4.77810054425197e-06	1.99853327	intronic	RP1	.		
3M	rs2375219	8	54785735	3.05253330469401e-06	2.05700167	intronic	RP1	.		
3M	rs1391462	8	54787221	4.79440218462524e-06	1.9984766	intronic	RP1	.		
3M	rs12678939	8	54792461	4.93547251213243e-06	2.01975618	intronic	RP1	.		
3M	rs1498183	8	54804345	4.76724404445351e-06	2.0245746	intronic	RP1	.		
12M	rs62515405	8	56143419	4.69438076015372e-06	8.07691204	intergenic	MOS;PLAG1	dist=29437;dist=17490	MOS;PLAG1	
12M	rs62515436	8	56228644	3.44618686083172e-06	9.27545902	intergenic	CHCHD7;SDR16C5	dist=9835;dist=71004	CHCHD7;SDR16C5	
12M	rs12114488	8	61760164	1.33492404675942e-06	2.62307914	intergenic	MIR4470;LINC02155	dist=45305;dist=129675		ASPH;NKAIN3
3M	rs117816016	8	102739034	3.21886378098173e-06	19.8807023	intergenic	LOC101927245;GASAL1	dist=52311;dist=67788		KLF10;AZIN1
3M	rs567383525	8	114017265	1.83606414606387e-06	17.7455227	intergenic	CSMD3;TRPS1	dist=580326;dist=1391230	CSMD3	
3M	rs545550279	8	114540200	4.75001774825929e-06	19.1812421	intergenic	CSMD3;TRPS1	dist=1103261;dist=868295	TRPS1	
3M	rs536803366	8	121989172	1.65900394985684e-06	19.2098639	intergenic	HAS2;SMILR	dist=343847;dist=425155		
3M	rs532513136	8	134801505	7.47175269151994e-07	19.9490594	upstream	MIR30B	dist=898		ZFAT
3M	rs532730683	9	1784492	1.02051348042702e-06	18.726838	intergenic	DMRT2;SMARCA2	dist=726938;dist=230855	DMRT2;SMARCA2	
3M	rs540065886	9	2770228	1.90434082268377e-06	19.9489151	intergenic	KCNV2;PUM3	dist=40191;dist=33927	PUM3	
3M	rs543844012	9	30107025	4.37741040782571e-06	18.1160485	intergenic	LINGO2;LINC01242	dist=893424;dist=281910	LINGO2	
3M	rs148556485	9	81408911	5.40048598404711e-07	21.3800152	intergenic	LINC01507;TLE1	dist=1374356;dist=174772	TLE1	
3M	rs140782222	9	81413979	4.53117855536307e-07	21.791526	intergenic	LINC01507;TLE1	dist=1379424;dist=169704		
3M	rs188034471	9	83322799	2.79197739516285e-06	14.7559999	intronic	FRMD3	.		
3M	rs190294315	9	83330550	4.29120280825722e-07	14.5664276	intronic	FRMD3	.	FRMD3	
3M	rs545690161	9	90268417	1.13978419923937e-08	17.1436874	intergenic	MIR4290HG;LINC01508	dist=226918;dist=32479		
3M	rs565682685	9	90460046	3.57832955336558e-07	18.938397	intergenic	LINC01508;LINC01501	dist=26557;dist=2386		
3M	rs183737367	9	90567765	3.8014567877404e-08	23.3452306	ncRNA_intronic	LINC01501	.		
3M	rs187213609	9	90653183	5.71707003010382e-08	23.1975304	intergenic	DIRAS2;SYK	dist=10359;dist=148417	DIRAS2;SYK	
12M	rs144138711	9	97160137	1.39149931661618e-06	6.08547131	ncRNA_intronic	ANKRD18CP	.		CTSV;CCDC180
3M	rs150027952	9	100623134	1.2828864296083e-06	21.8616648	intergenic	CAVIN4;PLPPR1	dist=34747;dist=405593	PLPPR1	
3M	rs146207930	9	126280057	2.39787202381566e-06	11.8624321	intergenic	LOC101929116;MVB12B	dist=4142;dist=46772	MVB12B	
3M	rs78296164	9	132391328	6.93811773227571e-07	11.6095338	intronic	TTF1	.	TTF1	
3M	rs77871739	9	135660463	3.19831184275799e-06	15.0724603	intergenic	GLT6D1;LCN9	dist=20923;dist=2859		MRPS2;CAMSAP1
3M	rs118040657	10	3430654	4.61906712585886e-06	12.1147712	ncRNA_intronic	LOC105376360	.		PITRM1;KLF6
3M	rs184425183	10	13415520	1.92440370103676e-09	22.6683174	intergenic	SEPHS1;BEND7	dist=67222;dist=22961	SEPHS1;BEND7	
3M	rs184458518	10	13429195	1.48323670154931e-09	22.8015789	intergenic	SEPHS1;BEND7	dist=80897;dist=9286		
3M	rs117998251	10	13455976	1.19486099712372e-09	22.9543311	intronic	BEND7	.		
3M	rs117025967	10	19918123	5.25165736443477e-07	9.66973563	intronic	PLXDC2	.		MALRD1;NEBL
3M	rs529011661	10	20083387	2.34383539239577e-06	15.6393469	intronic	PLXDC2	.		
12M	rs142956968	10	23201236	8.84691036334147e-07	10.4167992	downstream	C10orf67	dist=680		MSRB2;OTUD1
12M	rs145764464	10	23312918	1.10461979254428e-06	9.5967306	intronic	C10orf67	.		
3M	rs12266995	10	24563854	3.92980514429013e-06	5.96782535	intergenic	KIAA1217;ARHGAP21	dist=16006;dist=19760	ARHGAP21	
3M	rs138249376	10	61756071	4.86147789321271e-06	16.4300711	intronic	CABCOCO1	.		TMEM26;ARID5B
3M	rs140277951	10	80599344	1.98902982335697e-07	13.7463158	intronic	SH2D4B	.		TSPAN14;NRG3
3M	rs566018180	10	84995296	4.3799943792317e-06	18.563552	intergenic	CCSER2;LINC01519	dist=476775;dist=198125	CCSER2	
3M	rs140706881	10	94457778	1.40251021425162e-06	18.974805	intronic	TBC1D12	.	TBC1D12	
3M	rs117913371	10	101158729	2.30639701253664e-08	7.86609555	intergenic	TLX1NB;LINC01514	dist=17463;dist=17593		KAZALD1;BTRC
3M	rs75334617	10	101196395	7.30335368747825e-07	5.84603405	intergenic	LINC01514;LBX1	dist=2248;dist=30581		
3M	rs752259256	10	103165562	4.52164287436037e-06	20.4258639	intronic	NT5C2	.	NT5C2	
12M	rs147944608	10	108663951	9.83587566974791e-07	11.2205243	intergenic	LINC01435;XPNPEP1	dist=594658;dist=1200815	rs144138711	
3M	rs180828621	10	122773893	6.39935885478647e-08	15.1380574	ncRNA_intronic	DMBT1L1	.		CUZD1;FAM24B
3M	rs147393020	10	123019758	1.64586496415273e-06	14.1853505	intronic	ACADSB	.	ACADSB	
3M	rs193093906	10	125016920	3.17719154902332e-06	10.5219537	intronic	CTBP2	.	CTBP2	
12M	rs556274646	11	439012	1.93909342324625e-06	12.8914592	intronic	ANO9	.	ANO9	
12M	rs148815783	11	1013992	2.06734210421034e-06	13.5980313	exonic	MUC6	.		AP2A2;
3M	rs151115079	11	18634194	4.89469717121508e-08	17.8273587	intronic	SPTY2D1	.		
3M	rs138414342	11	18657851	3.15830767684997e-08	18.1582505	intergenic	SPTY2D1;TMEM86A	dist=23509;dist=40928	SPTY2D1;TMEM86A	
3M	rs541653703	11	18680239	2.1765671670172e-08	18.3169499	intergenic	SPTY2D1;TMEM86A	dist=45897;dist=18540		
3M	rs118093638	11	18696777	9.7151911784911e-07	14.2657259	intergenic	SPTY2D1;TMEM86A	dist=62435;dist=2002		
3M	rs181812512	11	66898258	4.24789238469822e-06	19.5093168	intronic	PC	.	PC	
3M	rs529345909	11	67343381	4.87261379674454e-06	18.70153	ncRNA_intronic	LOC100130987	.	LOC100130987	SSH3;POLD4
3M	rs544042801	11	68692775	3.07835940417967e-06	15.8869866	intergenic	GAL;TESMIN	dist=1600;dist=14665	GAL	
3M	rs149949098	11	95366702	2.52728186016237e-06	8.55927625	intergenic	LOC100129203;FAM76B	dist=132298;dist=402251	FAM76B	
3M	rs74521112	11	99218416	3.48297260681022e-07	6.25632387	intronic	CNTN5	.	CNTN5	
3M	rs79213709	11	99222724	5.77809319512938e-07	6.17081884	intronic	CNTN5	.		
3M	rs112007361	11	99317649	2.27561716225072e-07	6.35658468	intronic	CNTN5	.		
3M	rs148781275	11	103769875	2.2488967712367e-06	15.7111853	intergenic	DYNC2H1;MIR4693	dist=290012;dist=80031	DYNC2H1	
3M	rs141281289	11	123822316	3.07596924681855e-07	15.811518	intergenic	OR6M1;TMEM225	dist=15967;dist=60603		No gene nearby
3M	rs79539453	11	125396457	1.65356394618456e-06	20.8220616	intronic	PKNOX2	.	PKNOX2	
3M	rs528140343	11	125849332	9.33313906476653e-07	18.2835521	intergenic	PATE4;HYLS1	dist=9260;dist=34282		
3M	rs546409459	11	125885094	2.2842558352674e-06	18.9195474	intronic	HYLS1	.	HYLS1	
3M	rs528609331	11	125972300	7.07196590950882e-07	19.5812418	intronic	CDON	.	CDON	
3M	rs7104959	11	129976231	3.12645341825951e-06	17.9204611	intronic	PRDM10	.	PRDM10	
12M	rs117699122	12	451096	1.18257175082115e-06	21.4238256	intergenic	CCDC77;B4GALNT3	dist=8456;dist=8843	CCDC77;B4GALNT3	
3M	rs189360484	12	1761344	3.94900202113111e-07	16.8078794	intronic	ADIPOR2	.	ADIPOR2	
12M	rs61918041	12	6572702	4.39893214125342e-06	7.97264643	intronic	CHD4	.	CHD4	
12M	rs113244573	12	6575219	4.49284300484012e-06	7.97095746	intronic	CHD4	.		
3M	rs141754456	12	19998198	1.82427554715572e-07	12.1460333	intergenic	AEBP2;LINC02398	dist=475971;dist=16487	AEBP2	
3M	rs118184666	12	20271815	2.47036908420995e-06	10.9509803	intergenic	LINC02468;PDE3A	dist=143914;dist=96722		
3M	rs549931083	12	20363352	3.06794275866128e-07	14.3282694	intergenic	LINC02468;PDE3A	dist=235451;dist=5185	PDE3A	
3M	rs151323346	12	20859090	2.59127051015029e-06	15.8903888	intronic	SLCO1B3;SLCO1B3-SLCO1B7	.		
3M	rs371879555	12	22863028	1.69721932161597e-06	20.1033765	intergenic	ETNK1;LOC101928441	dist=172363;dist=312608	ETNK1	
3M	rs183466664	12	26668754	3.57331936763708e-06	16.0181882	intronic	ITPR2	.	ITPR2	
3M	rs77353774	12	28095919	1.00362822173041e-06	13.1054362	intergenic	PTHLH;LOC729291	dist=123186;dist=89706	PTHLH	

3M	rs113167689	12	28283029	2.31830826822625e-07	14.1364135	intronic	CCDC91	.		
3M	rs17510814	12	28316036	2.30925017950923e-07	14.0387924	intronic	CCDC91	.	CCDC91	
3M	rs141756120	12	28358163	2.4075549984719e-07	14.278403	intronic	CCDC91	.		
3M	rs117991215	12	28358540	2.50265389686764e-07	14.333666	intronic	CCDC91	.		
3M	rs191930622	12	47890872	4.88013920852721e-06	19.6783579	intronic	VDR	.	VDR	
3M	rs56302696	12	47899047	4.02736708601484e-06	19.8959041	intronic	VDR	.		
3M	rs185620578	12	48175616	2.41563563938621e-06	20.7232267	intergenic	ASB8;CCDC184	dist=18101;dist=8028	ASB8;CCDC184	
3M	rs190806532	12	48468364	1.54808652729946e-06	21.326955	intergenic	ZNF641;ANP32D	dist=117118;dist=4195	ZNF641;ANP32D	
3M	rs568658857	12	49460215	4.20762702895843e-06	14.2361193	intronic	SPATS2	.	SPATS2	
3M	rs137880949	12	62912517	1.74526564511423e-07	21.6084034	intronic	PPM1H	.		
3M	rs191053292	12	63051500	2.51400610111527e-08	22.9266488	intergenic	PPM1H;AVPR1A	dist=116350;dist=91259	PPM1H;AVPR1A	
3M	rs182437250	12	63214686	7.11750755969757e-07	18.8083451	intergenic	AVPR1A;DPY19L2	dist=63485;dist=344227		
3M	rs12315614	12	64527177	4.12213451917224e-06	3.77710662	intergenic	TBK1;RASSF3	dist=25064;dist=83318	TBK1,RASSF3	
12M	rs149616342	12	98785035	4.17235147721501e-06	11.2978214	intronic	ANKS1B	.	ANKS1B	
3M	rs76327548	12	100789188	2.03544908278967e-06	9.17842714	intergenic	GAS2L3;ANO4	dist=160900;dist=5588		
3M	rs76904423	12	100794966	3.57310045627432e-06	9.80576231	UTR5	ANO4	NM_001286615:c.-1068200>0;I	GAS2L3;ANO4	
3M	rs180764936	12	101104843	1.48879381343194e-06	19.3473053	intronic	ANO4	.		
3M	rs185855183	12	101111348	1.21523926305084e-06	18.7397727	intronic	ANO4	.		
12M	rs566724618	13	22889700	1.0727068950386e-06	22.3433705	ncRNA_intronic	LINC00621	.	SGCG	
12M	rs147221953	13	22911583	1.86149707525764e-06	22.5809676	ncRNA_intronic	LINC00621	.		
3M	rs139598422	13	23312875	1.84258662797026e-08	20.4886522	intronic	SGCG	.		
3M	rs143371352	13	46809699	1.20142384300403e-07	20.5341625	intergenic	ESD;HTR2A	dist=12538;dist=21847	ESD;HTR2A	
3M	rs75186966	13	46821623	1.30324459401267e-07	20.2281345	intergenic	ESD;HTR2A	dist=24462;dist=9923		
3M	rs150077525	13	57405415	1.65979661476121e-06	12.9984875	intergenic	PRR20E;PCDH17	dist=235197;dist=226329	PCDH17	
3M	rs534845494	13	57639730	5.5584801687088e-07	15.9431765	intronic	PCDH17	.		
3M	rs140062526	13	58711899	3.07703679852045e-06	14.0926088	intergenic	LINC00374;DIAPH3	dist=478782;dist=953688	DIAPH3	
12M	rs113791989	13	61344755	7.13417559871126e-07	25.2049582	intergenic	MIR3169;PCDH20	dist=144874;dist=64931	PCDH20	
12M	rs532269430	13	73480315	9.38644328296013e-07	18.6173752	intergenic	KLF5;LINC00392	dist=402772;dist=83929	KLF5	
12M	rs368187808	13	73783371	1.40533873966635e-06	21.2991251	intronic	KLF12	.	KLF12	
12M	rs372194899	13	73783373	1.40312572084772e-06	21.3012101	intronic	KLF12	.		
3M	rs546286713	13	90758825	2.98443520289082e-06	15.7027841	intergenic	LINC01049;LINC00410	dist=223484;dist=132129		SLITRK5;GPC5
3M	rs567080482	13	94423151	2.95390135279034e-06	15.4999561	intergenic	GPC6;DCT	dist=15132;dist=13660	GPC6;DCT	
3M	rs142928734	13	100948828	4.29382451218382e-06	17.009593	ncRNA_intronic	NALCN	.	NALCN	
3M	rs556680896	13	100950161	4.26743541179837e-06	17.0254659	ncRNA_intronic	NALCN	.		
3M	rs184265355	13	107359004	7.66559973381936e-07	14.4271719	intronic	FAM155A	.	FAM155A	
3M	rs572961122	13	107360637	3.07761414918354e-06	13.3354896	intronic	FAM155A	.		
3M	rs528809914	13	112386942	2.27269814316061e-07	19.5048552	intronic	SPACA7	.		TUBGCP3
3M	rs180765647	13	113724338	4.20477084480749e-06	20.1246521	intronic	GRK1	.		ATP4B;TMEM255B
3M	rs138215817	14	22173619	6.27722428502198e-07	16.311019	intergenic	OR4E1;LOC105370401	dist=502284;dist=206292		SALL2;
3M	rs74704551	14	29692681	1.97941397505543e-08	24.1990705	intronic	PRKD1	.	PRKD1	
3M	rs1686289	14	45791779	3.52212847732742e-06	-2.2551539	intergenic	LINC02303;LINC00871	dist=76177;dist=272380		MIS18BP1;RPL10L
3M	rs176783	14	45811710	3.87832460630423e-06	2.28010147	intergenic	LINC02303;LINC00871	dist=96108;dist=252449		
3M	rs176786	14	45813767	3.37672684888795e-06	2.28475641	intergenic	LINC02303;LINC00871	dist=98165;dist=250392		
3M	rs428110	14	45825457	4.15182083903445e-06	2.28482299	intergenic	LINC02303;LINC00871	dist=109855;dist=238702		
3M	rs116862847	14	63674959	2.1204003875388e-07	15.3160369	intergenic	WDR89;SGPP1	dist=33088;dist=9258	WDR89;SGPP1	
3M	rs569916471	14	75424639	3.10710931450174e-06	15.6404088	intergenic	LINC01220;JDP2	dist=128231;dist=3085	JDP2	
3M	rs113767990	14	81251219	4.97864018338844e-06	14.5811155	intergenic	LOC101928504;STON2	dist=27839;dist=9431	STON2	
3M	rs190251199	14	105124240	6.2650518901636e-07	20.0125292	intergenic	LINC02298;JAG2	dist=24744;dist=16758	JAG2	
12M	rs190251199	14	105124240	1.9790595982044E-06	22.6385061	intergenic	LINC02298;JAG2	dist=24744;dist=16758		
12M	rs187281112	15	32821186	3.3019635783423e-06	14.7343356	intronic	FMN1	.	FMN1	
12M	rs56324718	15	39987872	7.5163534768267e-07	12.5117374	intronic	EIF2AK4	.	EIF2AK4	
3M	rs185155853	15	40951902	3.01500290127566e-06	15.9164657	intergenic	DLL4;CHAC1	dist=12829;dist=1569		
3M	rs144026361	15	40956471	2.67002672054584e-06	16.1372994	UTR3	CHAC1	NM_001142776:c.*6970>0;NM	DLL4;CHAC1	
3M	rs558614420	15	41518672	3.91771199682616e-06	13.9750831	intronic	RPAP1	.		LTK;TYRO3
3M	rs138109686	15	41759244	4.62000628076245e-06	13.5956705	intronic	MGA	.	MGA	
3M	rs145896760	15	41827024	4.80897800219791e-06	13.3688825	UTR3	MAPKBP1	NM_014994:c.*15880>0;NM_0	MAPKBP1	
3M	rs140642138	15	41832967	4.54678591939158e-06	13.5832221	intronic	JMJD7;JMJD7-PLA2G4B	.	JMJD7;JMJD7-PLA2G4B	
3M	rs6080	15	58545734	4.85754782149583e-06	5.66476745	intronic	LIPC	.		
3M	rs145439370	15	58587566	9.02072868608363e-07	6.89328891	intergenic	LIPC;ADAM10	dist=17722;dist=1243	LIPC;ADAM10	
3M	rs149425014	15	58659461	1.84020904634255e-06	7.55224792	intronic	ADAM10	.		
3M	rs146442492	15	58689916	3.24578114232023e-06	7.05925858	intronic	ADAM10	.		
3M	rs193253461	15	58937154	3.76206055565965e-07	10.5374468	intergenic	SLTM;RNF111	dist=3475;dist=50509	SLTM;RNF111	
3M	rs184117160	15	59112107	1.25384693259541e-06	10.3417236	intronic	CCNB2	.	CCNB2	
3M	rs80292573	15	59142887	7.87214777000235e-07	6.55323491	intronic	MYO1E	.	MYO1E	
3M	rs182303755	15	59342593	4.69869428218951e-07	10.5479917	intronic	MYO1E	.		
3M	rs138217865	15	93849653	1.29412742467123e-06	16.709641	intergenic	LOC105370980;LINC02207	dist=641605;dist=6907		RGMA;MCTP2
12M	rs80212581	16	6362966	6.33070894509269e-07	22.2762003	intronic	RBFOX1	.		
12M	rs140276610	16	6383734	1.75581952039286e-06	22.1280577	intronic	RBFOX1	.		
12M	rs138164904	16	6808238	3.60740717035677e-08	23.892641	intronic	RBFOX1	.	RBFOX1	
3M	rs553840536	16	25686574	3.61488566678171e-06	19.6203442	intergenic	ZKSCAN2;HS3ST4	dist=428729;dist=5385	ZKSCAN2;HS3ST4	
3M	rs183817723	16	59268871	2.48366342700424e-06	17.5860507	intergenic	GOT2;APOOP5	dist=534555;dist=485270	GOT2	
3M	rs144954214	16	76145464	8.96489764315735e-07	21.8113263	intergenic	CPHXL;CNTNAP4	dist=418974;dist=131937	CNTNAP4	
3M	rs529523094	16	77681654	1.09798611725829e-06	18.1584346	intergenic	ADAMTS18;NUDT7	dist=246620;dist=40838	NUDT7	
12M	rs116897913	17	16935848	4.78180386603079e-06	20.1396765	intergenic	TBC1D27P;TNFRSF13B	dist=2672;dist=3233	TNFRSF13B	
3M	rs146728064	17	19362127	3.79796041699169e-06	12.6400411	intronic	B9D1	.	B9D1	
12M	rs188353596	17	48001236	6.85080774831596e-07	21.8057947	intergenic	CDK5RAP3;COPZ2	dist=19450;dist=24931	CDK5RAP3;COPZ2	
3M	rs184613584	17	50430860	5.40834238559884e-07	14.1377974	intronic	ACSF2	.	ACSF2	
3M	rs191271637	17	54045899	2.76242881040307e-06	18.379836	intergenic	KIF2B;TOM1L1	dist=220706;dist=854792	KIF2B;TOM1L1	
12M	rs112148840	17	68237633	3.82795090389498e-06	24.0241075	intronic	AMZ2	.	AMZ2	
12M	rs2606194	17	79214741	9.99647458252552e-07	-5.6956979	intronic	RBFOX3	.	RBFOX3	
12M	rs147669485	18	29023686	9.8819660331789e-08	22.1107639	intergenic	CDH2;MIR302F	dist=846556;dist=1275226	CDH2	
3M	rs185819304	18	29421615	4.31513948321026e-07	19.9401113	intergenic	CDH2;MIR302F	dist=1244486;dist=877296		
3M	rs187942235	18	29450465	2.77341075652478e-07	20.3968658	intergenic	CDH2;MIR302F	dist=1273336;dist=848446	CDH2	

3M	rs139493286	18	31236056	3.23770514196012e-06	17.4603093	intergenic	DSC1;DSG1	dist=73200;dist=82104	DSG1	
3M	rs143538552	18	31470299	1.40612679764676e-07	19.1457218	intronic	DSG3	.	DSG1;DSG3	
3M	rs373746073	18	31478421	1.55466285461099e-07	19.0847197	UTR3	DSG3	NM_001944:c.*21610>0		
3M	rs146333745	18	57830225	1.04499182739545e-06	17.4215784	intergenic	ATP8B1;NEDD4L	dist=26910;dist=214001	ATP8B1;NEDD4L	
12M	rs559152067	18	68362375	1.80839131277764e-06	24.7443692	intergenic	LOC643542;TMX3	dist=462756;dist=311313	TMX3	
3M	rs185464792	19	18686561	5.22729611714582e-07	22.5896015	intronic	CRTC1	.	CRTC1	
3M	rs186768950	19	18695314	4.67656376928778e-07	22.7152061	intronic	CRTC1	.		
3M	rs541288561	19	18758635	6.19920480169239e-07	15.6119012	intronic	CRTC1	.		
3M	rs559008174	19	18765249	6.10577196408926e-07	15.6025661	intronic	CRTC1	.		
3M	rs570407448	19	18769220	7.02455289936315e-07	15.4977984	intronic	CRTC1	.		
3M	rs546144116	19	19452530	2.29332366104571e-07	23.1354815	intronic	GATAD2A	.	GATAD2A	
3M	rs560206697	19	20546292	4.01812636832912e-08	24.9989943	intronic	ZNF737	.	ZNF737	
3M	rs111285015	19	22940396	3.3078789506342e-09	27.3347642	intergenic	ZNF723;ZNF728	dist=81729;dist=34487	ZNF728	
3M	rs1008091735	19	30599192	5.61396767442543e-07	18.4689243	intronic	ZNF536	.	ZNF536	
3M	rs148433854	19	30605571	4.40546670794092e-07	18.6267585	intronic	ZNF536	.		
12M	rs367732718	19	35427102	3.38991794276911e-06	23.8784923	intergenic	LINC01531;FFAR2	dist=10262;dist=21155	FFAR2	
12M	rs562831582	19	50731053	5.70199111188332e-07	21.0856548	intergenic	CLEC11A;GPR32	dist=5345;dist=39411	CLEC11A	
12M	rs62192733	20	1947511	2.76211842636971e-06	15.744618	ncRNA_exonic	PDYN	.		SIRPA;STK35
12M	rs547186621	20	6126094	2.39683963851735e-06	21.5510829	intergenic	FERMT1;CASC20	dist=3064;dist=300638	FERMT1	
3M	rs2327968	20	15832846	4.58567809597369e-06	6.15899856	intronic	MACROD2	.	MACROD2	
3M	rs2876414	20	15833059	4.41048907235012e-06	6.81910907	intronic	MACROD2	.		
3M	rs140788628	20	15877856	2.32626831948532e-07	11.8039284	intronic	MACROD2	.		
3M	rs559228693	20	15982684	3.15016069009906e-06	14.3643551	ncRNA_intronic	LOC613266	.		
12M	rs138733283	20	32087884	5.87309369634527e-07	18.2744744	intronic	HCK	.	HCK	
12M	rs149859280	20	32090367	5.71700141373349e-07	18.3057707	intronic	HCK	.		
12M	rs146249289	20	32094428	2.01850841331451e-07	19.6109527	intronic	HCK	.		
12M	rs145791959	20	32134626	8.67001886662025e-07	17.9091953	intronic	TM9SF4	.	TM9SF4	
12M	rs193041547	20	32184077	1.80079654856719e-07	19.6791358	intergenic	TM9SF4;TSPY26P	dist=16819;dist=5069		
12M	rs138055631	20	32192841	1.06243999550455e-06	17.6647762	UTR3	PLAGL2	NM_002657:c.*36110>0	PLAGL2	
12M	rs145421321	20	32274323	1.72232779371331e-06	17.0212611	intergenic	POFUT1;KIF3B	dist=35665;dist=3328	POFUT1;KIF3B	
12M	rs143432612	20	32304972	1.74726298083525e-06	16.9387232	intronic	KIF3B	.		
12M	rs139816293	20	32333540	1.16528363587405e-06	17.1384248	UTR3	KIF3B	NM_004798:c.*22210>0		
12M	rs200198574	20	32358851	2.44213593266147e-06	16.5282735	intronic	ASXL1	.	ASXL1	
12M	rs148157126	20	32361036	1.42669073977956e-07	19.7865001	intronic	ASXL1	.		
12M	rs192855100	20	32520938	4.17301607264236e-07	20.1986097	intronic	NOL4L	.	NOL4L	
3M	rs557092705	20	35601989	4.54067714932956e-06	19.4769546	ncRNA_exonic	FER1L4	.		ERGIC3;SPAG4
12M	rs2427460	20	62959430	5.30063377285508e-07	-2.574044	intronic	SLC17A9	.	SLC17A9	
3M	rs184785969	21	15799112	8.26040996676763e-08	19.8736486	intronic	USP25	.	USP25	
3M	rs117280553	21	15834844	5.79538719308743e-08	20.087417	intronic	USP25	.		
3M	rs79486609	21	15872687	4.53094466952756e-08	20.6624886	intronic	USP25	.		
3M	rs73227413	21	21764653	2.74560920737872e-06	5.96205514	ncRNA_intronic	LINC01425	.		
3M	rs75024143	21	21784226	2.0964109268482e-06	9.81254241	ncRNA_intronic	LINC01425	.		
3M	rs192134381	21	22078395	5.23445134161758e-09	21.788812	ncRNA_intronic	LINC01687	.		NCAM2;MRPL39
3M	rs118183140	21	34105187	1.16709964426479e-06	7.5893248	UTR3	SLC5A3	NM_006933:c.*78320>0	SLC5A3	
3M	rs183586634	21	37390730	1.72123504106205e-06	14.3132202	intronic	DYRK1A	.	DYRK1A	
3M	rs117185941	21	37394182	1.2357093777619e-06	15.3808786	intronic	DYRK1A	.		
3M	rs118084887	21	37491518	1.91359719468201e-06	14.9382616	intronic	DYRK1A	.		
12M	rs9636964	21	39932840	2.72523192147064e-07	-4.2543192	intergenic	PCP4;DSCAM	dist=3448;dist=78161	PCP4;DSCAM	
12M	rs9305683	21	39933795	8.57246540213203e-07	-4.1163679	intergenic	PCP4;DSCAM	dist=4403;dist=77206		
12M	rs9974985	21	39935648	1.59836223443899e-07	-4.3459395	intergenic	PCP4;DSCAM	dist=6256;dist=75353		
12M	rs7275595	21	39935998	3.24552446398997e-07	-4.2609754	intergenic	PCP4;DSCAM	dist=6606;dist=75003		
12M	rs1005412	21	39937023	1.75853470115381e-07	-4.4521096	intergenic	PCP4;DSCAM	dist=7631;dist=73978		
12M	rs9981433	21	39937640	1.14042775714184e-06	-4.1220124	intergenic	PCP4;DSCAM	dist=8248;dist=73361		
3M	rs150539922	21	41856807	4.79442196264294e-06	16.6696937	intronic	PRDM15	.	PRDM15	
3M	rs113625788	22	19981659	1.17014602462676e-06	11.8084829	exonic	ARVCF	.	ARVCF	
3M	rs78547898	22	32428291	4.85417555255089e-07	20.9098395	intronic	BPIFC	.		RTCB;FBXO7
12M	rs77297738	22	34077554	2.01836979224915e-07	10.2480572	intergenic	LARGE1;LINC02885	dist=154731;dist=679113	LARGE1	
12M	rs74572772	22	34096660	1.39505561568774e-07	10.2225183	intergenic	LARGE1;LINC02885	dist=173837;dist=660007		
12M	rs80019988	22	34097658	1.52143693387141e-07	10.0623306	intergenic	LARGE1;LINC02885	dist=174835;dist=659009		
3M	rs541680196	22	40132086	1.06912975962765e-06	14.3867808	intronic	TNRC6B	.	TNRC6B	
3M	rs185139807	22	40198777	1.02186831937043e-06	14.4330526	intronic	TNRC6B	.		
3M	rs141127122	22	40208435	3.1593298841889e-06	15.3824875	intronic	TNRC6B	.		
3M	rs148998974	22	40224526	8.65242968473446e-07	14.5129532	intronic	TNRC6B	.		
3M	rs555040883	22	40235472	3.05234476142884e-06	15.4273069	intronic	TNRC6B	.		
3M	rs182959028	22	45427152	2.37697466245549e-06	11.944461	intronic	RIBC2	.	RIBC2	
3M	rs150946694	22	46457283	3.3001729159907e-06	15.3082479	intronic	CELSR1	.	CELSR1	

Combined Results

7 Risk loci cluster these 11 SNPs

GENOME-WIDE RISK LOCI

SUGGESTIVE RISK LOCI (not genome-wide)

SUGGESTIVE RISK LOCI (including genome-wide)

TOTAL GENOME-WIDE & SUGGESTIVE

MERGED (Sum minus overlap)

Overlapping Risk Loci

ALK, DCANP1, JAG2, KCND3, PPARGC1A, STAG1, TBLXR1

GENOME-LEVEL SNPS

AGTR1, CDH2, ETAA1, MTX2, SGCG

SUGGESTIVE SNPS

TOTAL SNPS

SNPS REPEATED 12&3 MO

Supplementary Table S4. Target Gene Prioritization
Co-localization of associated SNPs with eQTLs or sQTLs and their target genes

NOTES
SNPs are grouped by risk locus and ordered by chromosomal position; risk loci are boxed; the one risk locus with SNPs of genome-wide signficance is highlighted in gray

HEADERS
QT: quantitiative trait; MAF ALPHA: minor allele frequency, dbSNP ALPHA project; rsid: reference SNP cluster ID, chr: chromosome;
pos_38, position of SNP on GRCh38 reference panel; REF and ALT, reference allele and alternative allele; Func.refGene: SNP location with respect to nearest gene;
Gene.refGene; nearest gene upstream and downstream; GeneDetail.refGene: distance to nearest gene upstream and downstream;
AOP: aqueous outflow pathway; QTL: quantitative trait locus; eQTL: expression quantitative trait locus; sQTL: slicing quantitative trait locus;
Gray Key for MAF ALPHA
light gray shaded: common SNP; mid-gray shaded: low frequency SNP; dark gray shaded: rare SNP
Counts
Loci and SNP counts are at bottom

QT	MAF ALPHA	rsid	chr	pos_38	REF	ALT	Func.refGene	Gene.refGene	GeneDetail.refGene	Closest gene(s) AOP-expressed	QTL driven gene, AOP-expressed	QTL type	Effect	QTL driven gene, AOP-expressed	QTL type	Effect	QTL driven AOP-expressed gene	QTL type	Effect
12 mo	0.093415	rs17127656	1	65477788	C	T	intronic	LEPR	.	LEPR									
12 mo	0.08334	rs7518849	1	65483108	T	C	intronic	LEPR	.		LEPROT	eQTL	Positive						
12 mo	0.069508	rs11579567	1	65491458	C	A	intronic	LEPR	.		LEPROT	eQTL	Positive						
12 mo	0.083589	rs7534177	1	65500037	A	G	intronic	LEPR	.		LEPROT	eQTL	Positive						
3 mo	0.002859	rs140420703	1	102337928	T	G	intergenic	OLFM3;COL11A1	dist=340694;dist=538539	COL11A1									
3 mo	0.001323	rs563167766	1	102400809	G	A	intergenic	OLFM3;COL11A1	dist=403575;dist=475658										
3 mo	0.009519	rs77180278	1	102496326	T	C	intergenic	OLFM3;COL11A1	dist=499092;dist=380141		COL11A1	sQTL	Negative						
3 mo	0.017196	rs112351653	1	102754804	T	C	intergenic	OLFM3;COL11A1	dist=757570;dist=121663		COL11A1	sQTL	Negative						
3 mo	0.000583	rs180926150	1	102760770	C	T	intergenic	OLFM3;COL11A1	dist=763536;dist=115697										
3 mo	0.017561	rs114413507	1	102953612	T	C	intronic	COL11A1	intron 41 of 66		COL11A1	sQTL	Negative						
3 mo	0.01704	rs116672066	1	103007360	G	A	intronic	COL11A1	intron 15 of 66		COL11A1	sQTL	Negative						
3 mo	0.016995	rs111928960	1	103168079	G	A	intergenic	COL11A1;LOC101928436	dist=59557;dist=325967		COL11A1	sQTL	Negative						
3 mo	0.016305	rs113221952	1	103288418	A	G	intergenic	COL11A1;LOC101928436	dist=179896;dist=205628		COL11A1	sQTL	Negative						
3&12 mo	0.017673	rs76098744	1	111806750	C	T	intronic	KCND3	.	KCND3									
3&12 mo	0.009033	rs74683551	1	111811796	G	A	intronic	KCND3	.		WNT2B	eQTL	Positive						
3 mo	0.059723	rs2274996	1	229668791	C	T	intergenic	URB2;LINC01682	dist=8591;dist=206759	URB2	TAF5L	sQTL	Negative						
3 mo	0.096148	rs2274997	1	229668899	A	G	intergenic	URB2;LINC01682	dist=8699;dist=206651		TAF5L	sQTL	Negative						
3 mo	0.05264	rs2891865	1	229670621	A	G	intergenic	URB2;LINC01682	dist=10421;dist=204929										
3 mo	0.069533	rs2385790	1	229671745	C	T	intergenic	URB2;LINC01682	dist=11545;dist=203805		TAF5L	sQTL	Negative						
3 mo	0.025802	rs12024557	1	229676610	A	C	intergenic	URB2;LINC01682	dist=16410;dist=198940										
3 mo	0.052214	rs4562666	1	229689023	T	C	intergenic	URB2;LINC01682	dist=28823;dist=186527										
3 mo	0.090471	rs12036586	1	229690631	G	A	intergenic	URB2;LINC01682	dist=30431;dist=184919		not AOP expressec	sQTL	Negative						
3 mo	0.084159	rs16850124	1	229695584	T	C	intergenic	URB2;LINC01682	dist=35384;dist=179966		TAF5L	sQTL	Negative						
3 mo	0.060487	rs12045643	1	229698303	C	T	intergenic	URB2;LINC01682	dist=38103;dist=177247										
3 mo	unknown	rs17746486	2	95056864	C	T	intergenic	MAL;MRPS5	dist=2872;dist=28507	MAL;MRPS5	GPAT2	eQTL	Positive	ZNF514	eQTL	Positive	ZNF514	sQTL	Positive
3 mo	0.015	rs73085348	3	42669729	A	G	intergenic	ZBTB47;KLHL40	dist=2149;dist=15808	ZBTB47	NKTR	eQTL	Negative	KRBOX1	eQTL	Negative			
3 mo	0.007	rs139943877	3	155733500	G	A	intronic	PLCH1	.	PLCH1	GMPS	eQTL	Positive	PLCH1	eQTL	Negative			
12 mo	0.014	rs76356799	3	179875980	G	A	intronic	PEX5L	.	PEX5L	USP13	eQTL	Positive						
12 mo	0.069	rs17615362	4	169012936	G	A	intergenic	CBR4;SH3RF1	dist=2681;dist=81323	SH3RF1	CBR4	eQTL	Positive	CBR4	sQTL	Pos/Neg	PALLD	eQTL	Negative
12 mo	0.023	rs17543620	4	169013574	T	C	intergenic	CBR4;SH3RF1	dist=3319;dist=80685		CBR4	eQTL	Positive	CBR4	sQTL	Pos/Neg	PALLD	eQTL	Negative
3 mo	0.002	rs111407636	5	95744325	C	T	intronic	RHOBTB3	.	RHOBTB3									
3 mo	unknown	rs111676272	5	95758594	C	A	intronic	RHOBTB3	.										
3 mo	0.008602	rs75848314	5	95762636	T	C	intronic	RHOBTB3	.		PCSK1	eQTL	Negative						
3 mo	0.012388	rs111846247	5	95775939	T	C	intronic	RHOBTB3	.		GLRX	eQTL	Positive						
3 mo	0.030	rs17776100	7	6386848	G	A	intronic	RAC1	.	RAC1	CCZ1	eQTL	Negative	CCZ1	sQTL	Pos/Neg	CCZ1B	eQTL	Positive
3 mo	0.28	rs9643828	8	54616513	C	T	intronic	RP1	.	SOX17;XKR4	not AOP expressec	eQTL	Positive						
3 mo	0.365421	rs423841	8	54643509	G	A	intronic	RP1	.		XKR4	eQTL	Positive						
3 mo	0.24537	rs433324	8	54652049	A	G	intronic	RP1	.		XKR4	eQTL	Positive						
3 mo	0.31998	rs369623	8	54659380	A	C	intronic	RP1	.		XKR4	eQTL	Positive						
3 mo	0.292979	rs446222	8	54662400	G	A	intronic	RP1	.		not AOP expressec	eQTL	Positive						
3 mo	0.499229	rs432393	8	54667738	C	T	intronic	RP1	.		XKR4	eQTL	Positive						
3 mo	0.380572	rs3098298	8	54670278	C	T	intronic	RP1	.		XKR4	eQTL	Positive						
3 mo	0.380413	rs367179	8	54675056	T	C	intronic	RP1	.		XKR4	eQTL	Positive						
3 mo	0.2961237	rs382476	8	54678415	G	A	intronic	RP1	.		XKR4	eQTL	Positive						
3 mo	0.320148	rs384543	8	54679049	G	A	intronic	RP1	.		XKR4	eQTL	Positive						
3 mo	0.392869	rs405226	8	54679776	A	G	intronic	RP1	.		XKR4	eQTL	Positive						
3 mo	0.313393	rs384127	8	54684929	G	A	intronic	RP1	.		XKR4	eQTL	Positive						
3 mo	0.296631	rs858397	8	54702130	A	G	intronic	RP1	.		XKR4	eQTL	Negative						
3 mo	0.345139	rs2375537	8	54706948	C	T	intronic	RP1	.		XKR4	eQTL	Negative						
3 mo	0.393862	rs720372	8	54716077	G	A	intronic	RP1	.		XKR4	eQTL	Negative						
3 mo	0.311297	rs1437781	8	54717292	T	C	intronic	RP1	.		XKR4	eQTL	Negative						
3 mo	0.339565	rs1595406	8	54718055	A	G	intronic	RP1	.		XKR4	eQTL	Negative						
3 mo	0.2992666	rs1437782	8	54720202	C	T	exonic	RP1	.		XKR4	eQTL	Negative						
3 mo	0.304288	rs10105693	8	54727912	C	T	intronic	RP1	.		XKR4	eQTL	Negative						
3 mo	0.34109	rs2375536	8	54728162	T	C	intronic	RP1	.		XKR4	eQTL	Negative						
3 mo	0.31424	rs4737674	8	54749094	C	A	intronic	RP1	.		XKR4	eQTL	Negative						
3 mo	0.306432	rs11987234	8	54757269	A	G	intronic	RP1	.		XKR4	eQTL	Negative						
3 mo	0.309582	rs13277510	8	54761589	G	A	intronic	RP1	.		XKR4	eQTL	Negative						
3 mo	0.372041	rs12548593	8	54762057	G	T	intronic	RP1	.		XKR4	eQTL	Negative						
3 mo	0.394494	rs1812506	8	54763541	A	G	intronic	RP1	.		XKR4	eQTL	Negative						
3 mo	0.313335	rs16920698	8	54765874	G	A	intronic	RP1	.		XKR4	eQTL	Negative						
3 mo	0.313298	rs1561297	8	54765978	A	C	intronic	RP1	.		XKR4	eQTL	Negative						
3 mo	0.309423	rs4737676	8	54766986	G	A	intronic	RP1	.		XKR4	eQTL	Negative						
3 mo	0.350238	rs2083123	8	54767758	C	T	intronic	RP1	.		XKR4	eQTL	Negative						
3 mo	0.302059	rs983248	8	54768232	C	T	intronic	RP1	.		XKR4	eQTL	Negative						
3 mo	0.312123	rs1391463	8	54769316	T	G	intronic	RP1	.		XKR4	eQTL	Negative						
3 mo	0.331682	rs10958428	8	54773081	A	G	intronic	RP1	.		XKR4	eQTL	Negative						
3 mo	0.290454	rs13278605	8	54775611	C	T	intronic	RP1	.		XKR4	eQTL	Negative						
3 mo	0.178507	rs13276543	8	54775614	G	T	intronic	RP1	.		XKR4	eQTL	Negative						
3 mo	0.141896	rs7822082	8	54777660	T	C	intronic	RP1	.		XKR4	eQTL	Negative						
3 mo	0.313945	rs4737201	8	54778898	C	T	intronic	RP1	.		XKR4	eQTL	Negative						
3 mo	0.420064	rs7843693	8	54779552	G	A	intronic	RP1	.		XKR4	eQTL	Negative						
3 mo	0.222222	rs1396896	8	54782750	A	G	intronic	RP1	.		XKR4	eQTL	Negative						
3 mo	0.224434	rs2375219	8	54785735	C	T	intronic	RP1	.										
3 mo	0.261909	rs1391462	8	54787221	C	A	intronic	RP1	.		XKR4	eQTL	Negative						
3 mo	0.354923	rs12678939	8	54792461	A	G	intronic	RP1	.										
3 mo	0.364267	rs1498183	8	54804345	C	T	intronic	RP1	.										
12 mo	unknown	rs12114488	8	61760164	G	A	intergenic	MIR4470;LINC02155	dist=45305;dist=129675	ASPH	ASPH	eQTL	Negative						
12 mo	0.023	rs144138711	9	97160137	T	C	ncRNA_intronic	ANKRD18CP	.	CTSV;CCDC180	CCDC180	sQTL	Pos/Neg						
3 mo	0.017	rs149949098	11	95366702	G	A	intergenic	LOC100129203;FAM76B	dist=132298;dist=402251	FAM76B	ENDOD1	eQTL	Positive						
12 mo	0.007191	rs145791959	20	32134626	G	A	intronic	TM9SF4	.	TM9SF4	LINC00028	eQTL	Negative						
12 mo	0.004852	rs193041547	20	32184077	T	C	intergenic	TM9SF4;TSPY26P	dist=16819;dist=5069										
12 mo	0.007191	rs138055631	20	32192841	G	A	UTR3	PLAGL2	NM_002657:c.*36110>0	PLAGL2	LINC00028	eQTL	Negative						
12 mo	0.007191	rs145421321	20	32274323	C	T	intergenic	POFUT1;KIF3B	dist=35665;dist=3328	POFUT1;KIF3B	LINC00028	eQTL	Negative						
12 mo	0.00667	rs143432612	20	32304972	C	T	intronic	KIF3B	.		LINC00028	eQTL	Negative						
12 mo	0.006569	rs139816293	20	32333540	C	T	UTR3	KIF3B	NM_004798:c.*22210>0		LINC00028	eQTL	Negative						
12 mo	0.481741	rs2427460	20	62959430	T	C	intronic	SLC17A9	.	SLC17A9	SLC17A9	eQTL	Negative	SLC17A9	sQTL	Positive	DIDO1	sQTL	Positive
12 mo	0.108413	rs9636964	21	39932840	A	G	intergenic	PCP4;DSCAM	dist=3448;dist=78161	PCP4;DSCAM	PCP4	eQTL	Negative						
12 mo	0.08756	rs9305683	21	39933795	G	A	intergenic	PCP4;DSCAM	dist=4403;dist=77206										
12 mo	0.116287	rs9974985	21	39935648	G	A	intergenic	PCP4;DSCAM	dist=6256;dist=75353										
12 mo	0.092658	rs7275595	21	39935998	G	A	intergenic	PCP4;DSCAM	dist=6606;dist=75003										
12 mo	0.096064	rs1005412	21	39937023	A	G	intergenic	PCP4;DSCAM	dist=7631;dist=73978										
12 mo	0.122456	rs9981433	21	39937640	G	T	intergenic	PCP4;DSCAM	dist=8248;dist=73361										

18 risk loci cluster SNPs that co-localize with QTLs: 6.87%
Total SNPs: 93
common SNPs: 67.74%
15 rare, 13 low frequency, 63 common, 3 unknown

Supplementary Table S4. Target Gene Prioritization
Prioritized target gene lists from the Indianapolis-1 discovery cohort analysis

NOTES
Alphabetical ordering of the full prioritized gene list highlights functionally similar paralogues or gene family members by grouping them together. We counted 41 such paralogous groups with shared function.
Information on gene product function is from the GeneCards Suite
HEADERS
AOP: aqueous outflow pathway; DEG: differentially expressed gene; eQTL: expression quantitative trait locus; TM: trabecular meshwork

Indianapolis-1 discovery cohort					
AOP-expressed	AOP-expressed up, downstream	AOP-expressed eQTLs	Combined	Paralogue groups	Gene product function
ABCG2	AGTR1	ASPH	ABCG2		Superfamily of ATP-binding cassette (ABC) transporters, white subfamily
ACADSB	ALDH8A1	CBR4	ACADSB		Acyl-CoA dehydrogenase family of enzymes that catalyze the dehydrogenation of acyl-CoA derivatives in the metabolism of fatty acids or branch chained amino acids
ACSF2	AP1AR	CCDC180	ACSF2	ACSF2	Acyl-CoA synthase family, catalyzes the initial reaction in fatty acid metabolism, by forming a thioester with CoA
ACSL3	AP2A2	CCZ1	ACSL3	ACSL3	"
ADAM10	ARAP2	CCZ1B	ADAM10		ADAM family transmembrane metalloprotease which mediates the ectodomain shedding of a myriad of transmembrane proteins
ADGRG6	ARID5B	COL11A1	ADGRG6		G-protein coupled receptor activated by type IV collagen
ADGRL3	ASB3	DIDO1	ADGRL3	ADGRL3	Latrophilin subfamily of G-protein coupled receptors
ADGRL4	ASPH	ENDOD1	ADGRL4	ADGRL4	"
ADIPOR2	ATP4B	GLRX	ADIPOR2		Adiponectin receptor for ADIPOQ, an essential hormone secreted by adipocytes that regulates glucose and lipid metabolism
AEBP2	ATP8A1	GMPS	AEBP2		Acts as an accessory subunit for the core Polycomb repressive complex 2 (PRC2), which mediates histone H3K27 (H3K27me3) trimethylation on chromatin leading to transcriptional repression of the affected target gene
AGAP1	AZIN1	GPAT2	AGAP1		GTPase-activating protein for ARF1 and ARF5
AGR3	BTRC	KRBOX1	AGR3		Disulfide isomerase (PDI) family of endoplasmic reticulum (ER) proteins that catalyze protein folding and thiol-disulfide interchange reactions
AGTR1	C4orf19	LEPROT	AGTR1		Receptor for angiotensin II, a potent vasopressor hormone and a primary regulator of aldosterone secretion
AHCTF1	CAMSAP1	LINC00028	AHCTF1		Involved in nuclear pore complex assembly and regulation of cytokinesis.
AHR	CCDC180	LINC00342	AHR		ligand-activated helix-loop-helix transcription factor involved in the regulation of biological responses to planar aromatic hydrocarbons.
ALDH5A1	CTSV	NKTR	ALDH5A1	ALDH5A1	Aldehyde dehydrogenase family
ALK	CUZD1	PALLD	ALDH8A1	ALDH8A1	"
AMY2A	DIRAS2	PCP4	ALK		receptor tyrosine kinase of the insulin receptor superfamily
AMY2B	EDEM3	PCSK1	AMY2A	AMY2A	Alpha-amylase family, catalyze starch catabolism
AMZ2	ERGIC3	PLCH1	AMY2B	AMY2B	"
ANKS1B	FAM24B	SLC17A9	AMZ2		Zinc metalloprotease that displays some activity against angiotensin-3
ANO4	FBX07	TAF5L	ANKS1B		Unknown
ANO9	GPC5	TEXT4	ANO4	ANO4	Anotamin family (Ca2+-activated Cl− channel)
ANP32D	GYPC	USP13	ANO9	ANO9	"
AOAH	ID2	WNT2B	ANP32D		Phosphoprotein 32 (PP32)
ARAP2	IQCM	XKR4	AOAH		Catalyzes the hydrolysis of acyloxyacyl-linked fatty acyl chains from bacterial lipopolysaccharides, effectively detoxifying these molecules
ARHGAP21	KAZALD1	ZNF514	AP1AR		Enables AP-1 adaptor complex binding activity and kinesin binding activity
ARHGEF26	KCTD8		AP2A2		Subunit of the AP-2 adaptor protein complex, which is involved in linking lipid and protein membrane components with the clathrin lattice
ARHGEF28	KLF10		ARAP2		Phosphatidylinositol 3,4,5-trisphosphate-dependent GTPase-activating protein that modulates actin cytoskeleton remodeling by regulating ARF and RHO family members.
ARRDC3	KLF6		ARHGAP21		Functions as a GTPase-activating protein (GAP) for RHOA and CDC42.
ARVCF	L1TD1		ARHGEF26	ARHGEF26	Rho-guanine nucleotide exchange factor (Rho-GEF) family
ASB3	LTK		ARHGEF28	ARHGEF28	"
ASB8	MALRD1		ARID5B		The encoded protein forms a histone H3K9Me2 demethylase complex with PHD finger protein 2 and regulates the transcription of target genes involved in adipogenesis and liver development
ASNS	MCTP2		ARRDC3		arrestin family of proteins, which regulate G protein-mediated signaling
ASXL1	MEOX2		ARVCF		Catenin family member that plays an important role in the formation of adherens junction complexes
ATP8B1	MIS18BP1		ASB3	ASB3	Family of substrate-recognition components of a SCF-like ECS (Elongin-Cullin-SOCS-box protein) E3 ubiquitin-protein ligase complex
ATXN1	MMS22L		ASB8	ASB8	"
AVPR1A	MND1		ASNS		Involved in the synthesis of asparagine
B4GALNT3	MRPL39		ASPH		Role in calcium homeostasis
B9D1	MRPS2		ASXL1		Probable Polycomb group (PcG) protein involved in transcriptional regulation mediated by ligand-bound nuclear hormone receptors, such as retinoic acid receptors (RARs) and peroxisome proliferator-activated receptor gamma (PPARG)
BEND7	MSRB2		ATP4B		P-type cation-transporting ATPase
C1D	NCAM2		ATP8A1	ATP8A1	Catalytic component of a P4-ATPase flippase complex
C1orf21	NEBL		ATP8B1	ATP8B1	"
C6orf118	NKAIN3		ATXN1		Chromatin-binding factor that repress Notch signaling in the absence of Notch intracellular domain by acting as a CBF1 corepressor.
CAV2	NRC32		AVPR1A		Receptor for arginine vasopressin
CBR4	NRG3		AZIN1		Antizyme inhibitor family
CCDC184	NRXN1		B4GALNT3		Catalyzes transfer of N-acetylgalactosamine (GalNAc) in N-linked glycans and probably O-linked glycans
CCDC77	OTUD1		B9D1		Component of the tectonic-like complex in primary cilia, required for ciliogenesis and sonic hedgehog/SHH signaling
CCDC91	PITRM1		BEND7		Transcription factor, unknown role
CCNB2	PITX2		BTRC		F-box protein, one of the four subunits of ubiquitin protein ligase complex SCF (SKP1-cullin-F-box), which functions in phosphorylation-dependent ubiquitination
CCNG1	POLD4		C1D		Nuclear Receptor Corepressor, binds RAC3, promotes apoptosis
CCR6	POU3F2		C1orf21		Unknown
CCSER1	RELL1		C4orf19		Unknown
CCSER2	RGMA		C6orf118		Unknown
CDC42BPA	RGMB		CAMSAP1		Key microtubule-organizing protein that specifically binds the minus-end of non-centrosomal microtubules and regulates their dynamics and organization
CDCA2	RGPD4		CAV2		Caveolin, a scaffolding protein within caveolar membranes
CDH2	RIOK2		CBR4		Carbonyl Reductase, forms part of the mitochondrial fatty acid synthase
CDH9	RND3		CCDC180		Coiled-Coil Domain-Containing Protein, unknown function
CDK5RAP3	RNF144A		CCDC184		Coiled-Coil Domain-Containing Protein, unknown function
CDON	RNF2		CCDC77		Coiled-Coil Domain-Containing Protein, unknown function
CELSR1	RPL10L		CCDC91		Coiled-Coil Domain-Containing Protein, involved in the regulation of membrane traffic through the trans-Golgi network
CFAP99	RTCB		CCNB2	CCNB2	Cyclin (cell cycle regulator)
CFTR	SALL2		CCNG1	CCNG1	"
CHAC1	SGK1		CCR6		Beta chemokine receptor family, ligand is CCL20
CHCHD7	SIRPA		CCSER1	CCSER1	Microtubule binding protein family, mediates bundle formation
CHD4	SLITRK5		CCSER2	CCSER2	"
CLEC11A	SOSTDC1		CCZ1	CCZ1	Enables guanyl-nucleotide exchange factor activity, predicted to be involved in vesicle-mediated transport.
CMC1	SOX17		CCZ1B	CCZ1B	"
CNTN4	SPAG4		CDC42BPA		Serine/threonine-protein kinase which is an important downstream effector of CDC42 and plays a role in the regulation of cytoskeleton reorganization and cell migratio
CNTN5	SSH3		CDCA2		Targeting subunit of the cell-cycle associated protein, protein phosphatase 1, with a role in targeting this protein to chromatin during anaphase.
CNTN6	ST6GAL2		CDH2	CDH2	Cadherin family (calcium-dependent cell adhesion protein)
CNTNAP2	STK35		CDH9	CDH9	"
CNTNAP4	TBL1XR1		CDK5RAP3		Substrate adapter of E3 ligase complexes mediating ufmylation, the covalent attachment of the ubiquitin-like modifier UFM1 to substrate proteins, and which is involved in various processes, such as ribosome recycling and reticulophagy (also called ER-phagy)
COL11A1	TLR2		CDON		Component of a cell-surface receptor complex that mediates cell-cell interactions between muscle precursor cells.
COPZ2	TM2D1		CELSR1		cadherin superfamily, flamingo subfamily
CRTC1	TMEM255B		CFAP99		Cilia And Flagella Associated Protein
CSMD1	TMEM26		CFTR		Superfamily of ATP-binding cassette (ABC) transporters, functions as a chloride channel; mutations in this gene cause cystic fibrosis
CSMD3	TSN		CHAC1		Gamma-glutamylcyclotransferase family; deglycinates the Notch receptor, which prevents receptor maturation and inhibits Notch signaling
CTBP2	TSPAN14		CHCHD7		Predicted to be involved in mitochondrial respiratory chain complex assembly
DCANP1	TUBGCP3		CHD4		CHD family (modifies chromatin structure)
DCT	TYRO3		CLEC11A		C-type lectin superfamily; functions as a growth factor for primitive hematopoietic progenitor cells
DIAPH3	XKR4		CMC1		Component of the MITRAC (mitochondrial translation regulation assembly intermediate of cytochrome c oxidase complex) complex, that regulates cytochrome c oxidase assembly
DIRAS2	ZFAT		CNTN4	CNTN4	Contactin (axon-associated cell adhesion molecule)
DLC1	ZMAT3		CNTN5	CNTN5	"
DLGAP2			CNTN6	CNTN6	"
DLL4			CNTNAP2	CNTNAP2	Contactin-associated protein family
DMRT2			CNTNAP4	CNTNAP4	"
DNASE2B			COL11A1		One of the three collagen chains that form type XI collagen
DPP10			COPZ2		subunit of the coatomer protein complex, a seven-subunit complex that functions in the formation of COPI-type, non-clathrin-coated vesicles. COPI vesicles function in the retrograde Golgi-to-ER transport of dilysine-tagged proteins
DSCAM			CRTC1		CREB-regulated transcription coactivator protein; involved in energy metabolism
DSG1			CSMD1	CSMD1	CUB And Sushi Multiple Domains 1 family, complement cascade inhibitor, epithelial-mesenchymal transition
DSG3			CSMD3	CSMD3	"
DYNC2H1			CTBP2		Corepressor targeting diverse transcription regulators. Functions in brown adipose tissue (BAT) differentiation
DYRK1A			CTSV		Cathepsin V, a lysosomal cysteine proteinase
E2F3			CUZD1		Localized to zymogen granules, where it functions in trypsinogen activation
EBF2			DCANP1		Binds with and transactivates the corticotropin-releasing hormone (CRH) promoter
EFHB			DCT		Dopachrome Tautomerase, role in melanin biosynthesis, also involved in energy metabolism, particularly related to adipose tissue function and lipid metabolism
EIF2AK4			DIAPH3		Diaphanous subfamily of the formin family; actin nucleation and elongation factor required for the assembly of F-actin structures, such as actin cables and stress fibers
EIF3IP1			DIDO1		Death inducer-obliterator-1 gene, upregulated by apoptotic signals
EPHB1			DIRAS2		DIRAS subfamily of small GTPases
ESD			DLC1		Rho GTPase Activating Protein
ETAA1			DLGAP2		May play a role in the molecular organization of synapses and neuronal cell signaling.
ETNK1			DLL4		Ligand in Notch signaling, Delta subfamily, characterized by a DSL domain, EGF repeats, and a transmembrane domain
EVX2			DMRT2		DMRT family transcriptional activator that directly regulates early activation of the myogenic determination
FAM155A			DNASE2B		Hydrolyzes DNA under acidic conditions
FAM76B			DPP10		S9B family in clan SC of the serine proteases, promotes cell surface expression of the potassium channel KCND2 and modulates its activity and gating characteristics
FAXDC2			DSCAM		immunoglobulin superfamily of cell adhesion molecules, role in neuronal self-avoidance
FBXO11			DSG1	DSG1	Desmoglein protein subfamily, components of desmosome
FERMT1			DSG3	DSG3	"
FFAR2			DYNC2H1		Dynein, may function as a motor for intraflagellar retrograde transport. Functions in cilia biogenesis.
FMN1			DYRK1A		Dual-specificity tyrosine phosphorylation-regulated kinase (DYRK) family
FOXN2			E2F3		Transcription factor, inhibits adipogenesis
FRMD3			EBF2		Transcription factor that acts in synergy with the Wnt-responsive LEF1/CTNNB1 pathway
FST			EDEM3		Involved in endoplasmic reticulum-associated degradation (ERAD), a quality control mechanism
FSTL5			EFHB		Microtubule inner protein (MIP) part of the dynein-decorated doublet microtubules (DMTs) in cilia axoneme; involved in regulation of calcineurin-NFAT signaling cascade and regulation of store-operated calcium entry
GABRG2			EIF2AK4		Member of a family of kinases that phosphorylate the alpha subunit of eukaryotic translation initiation factor-2 (EIF2), downregulating protein synthesis, involved in the integrated stress response
GABRP			EIF3IP1		Eukaryotic Translation Initiation Factor 3 Subunit I Pseudogene 1
GAL			ENDOD1		Plays a role in the modulation of innate immune signaling through the cGAS-STING pathway by interacting with RNF26
GALNT10			EPHB1		Receptor tyrosine kinase which binds promiscuously transmembrane ephrin-B family ligands residing on adjacent cells, leading to contact-dependent bidirectional signaling into neighboring cells.
GAS2L3			ERGIC3		Involved in endoplasmic reticulum to Golgi vesicle-mediated transport and positive regulation of intracellular protein transport.
GATAD2A			ESD		Serine hydrolase that belongs to the esterase D family, may be involved in the recycling of sialic acids

GMNC	ETAA1		Replication stress response protein that accumulates at DNA damage sites and promotes replication fork progression and integrity
GOT2	ETNK1		An ethanolamine kinase, may be a rate-controlling step in phosphatidylethanolamine biosynthesis
GPC6	EVX2		Homeobox transcription factor
GPLD1	FAM155A		Also known as NALF1; auxillary component of the NALCN sodium channel complex, a channel that regulates the resting membrane potential and controls neuronal excitability
GTF2B	FAM24B		Unknown
HAAO	FAM76B		Gene Ontology (GO) annotations related to this gene include deNEDDylase activity
HAS2	FAXDC2		Promotes megakaryocyte differentiation
HCK	FBXO11	FBXO11	Substrate recognition component of a SCF (SKP1-CUL1-F-box protein) E3 ubiquitin-protein ligase complex
HDAC4	FBXO7	FBXO7	"
HDAC9	FERMT1		Fermitin family member, involved in integrin signaling and linkage of the actin cytoskeleton to the extracellular matrix
HMCN1	FFAR2		GP40 family of G protein-coupled receptors, regulates whole-body energy homeostasis, adipogenesis
HOXD13	FMN1		Formin homology family, plays a role in the formation of adherens junction and the polymerization of linear actin cables
HS3ST1	FOXN2		Transcription factor
HS3ST4	FRMD3		Unknown
HS6ST1	FST	FST	TGFB superfamily member follistatin (activin antagonist)
HTR2A	FSTL5	FSTL5	"
HYLS1	GABRG2	GABRG2	Subunit of the GABA-A receptor, a major inhibitory neurotransmitter in the brain
IDUA	GABRP	GABRP	"
IGF2BP3	GAL		Neuroendocrine hormone of the central and peripheral nervous systems, role in contraction of smooth muscle
INTU	GALNT10		Catalyzes the first step in the synthesis of mucin-type oligosaccharides
IQCJ-SCHIP1	GAS2L3		Cytoskeletal linker protein, may promote and stabilize the formation of the actin and microtubule network
ITPR2	GATAD2A		Transcriptional repressor, acts as a component of the histone deacetylase NuRD complex which participates in the remodeling of chromatin
JADE1	GLRX		Glutaredoxin family, antioxidant defense system
JAG2	GMNC		Regulator of DNA replication
JDP2	GMPS		Catalyzes the conversion of xanthine monophosphate (XMP) to GMP
JMJD7	GOT2		Glutamic-oxaloacetic transaminase, role in the intracellular NAD(H) redox balance, facilitates cellular uptake of long-chain free fatty acids
JMJD7-PLA2G4B	GPAT2		Glycerol-3-phosphate O-acyltransferase
KCND3	GPC5	GPC5	Glypican family, act as co-receptors on the cell surface, playing crucial roles in regulating various signaling pathways by modifying the availability of growth factors through their heparan sulfate chain
KCND3	GPC6	GPC6	"
KCNE4	GPLD1		Glycosylphosphatidylinositol (GPI) anchor-degrading enzyme
KCNH8	GTF2B		General transcription factor IIB, one of the ubiquitous factors required for transcription initiation by RNA polymerase II
KCNIP1	GYPC		Integral membrane glycoprotein, regulates the mechanical stability of red cells
KCNIP3	HAAO		Catalyzes the synthesis of quinolinic acid (QUIN) from 3-hydroxyanthranilic acid, increased cerebral levels of QUIN may participate in the pathogenesis of neurologic and inflammatory disorders
KCNIP4	HAS2		Hyaluronic acid synthetase
KIAA1614	HCK		Src family of tyrosine kinases, regulates innate immune responses
KIF2B	HDAC4	HDAC4	Class IIa histone deacetylase
KIF3B	HDAC9	HDAC9	"
KLF12	HMCN1		Hemicentin, multifunctional
KLF5	HOXD13		Homeobox family transcription factors
KLHDC7A	HS3ST1	HS3ST1	Heparan sulfate biosynthetic enzyme family
KLHDC8A	HS3ST4	HS3ST4	"
LARGE1	HS6ST1	HS6ST1	"
LCORL	HTR2A		Seratonin receptor; stimulating these receptors increases IOP by increasing blood flow to the ciliary body, which increases aqueous humor production
LEMD1	HYLS1		Centriolar and ciliogenesis associated protein
LEPR	ID2		Transcriptional regulator, regulates the circadian clock, contributes to the regulation of a variety of liver clock-controlled genes involved in lipid metabolism
LINGO2	IDUA		Enzyme that catalyzes hydrolysis of the terminal alpha-L-iduronic acid residues of two glycosaminoglycans, dermatan sulfate and heparan sulfate, required for their degradation
LIPC	IGF2BP3		Binds to the 5' UTR of the insulin-like growth factor II leader 3 mRNA and may repress translation of insulin-like growth factor II
LOC100130987	INTU		Plays a key role in ciliogenesis
LPAR3	IQCJ-SCHIP1		Read-through protein; may play a role in action potential conduction in myelinated cells through the organization of molecular complexes at nodes of Ranvier and axon initial segments
LRP2	IQCM		Unknown
LUZP1	ITPR2		Inositol 1,4,5-triphosphate receptor family, whose members are second messenger intracellular calcium release channels
M1AP	JADE1		Enables transcription coactivator activity. Contributes to histone acetyltransferase activity. Involved in several processes, including negative regulation of canonical Wnt signaling pathway
MACROD2	JAG2		Notch ligand of the serrate family
MACROH2A1	JDP2		Component of the AP-1 transcription factor that represses transactivation mediated by the Jun family of protein
MAL	JMJD7		Endopeptidase that cleaves histones N-terminal tails at the carboxyl side of methylated arginine or lysine residues, to generate 'tailless nucleosomes', which may trigger transcription elongation
MAML3	JMJD7-PLA2G4B		Read-through protein; unknown function
MAPK10	KAZALD1		Secreted member of the insulin growth factor-binding protein (IGFBP) superfamily
MAPKBP1	KCND3	KCND3	Voltage-gated potassium (Kv) channel
MAT2B	KCNE4	KCNE4	"
MFSD1	KCNH8	KCNH8	"
MGA	KCNIP1	KCNIP1	Voltage-gated potassium (Kv) channel interacting protein
MMRN1	KCNIP3	KCNIP3	"
MOGAT1	KCNIP4	KCNIP4	"
MOGS	KCTD8		Auxiliary subunit of GABA-B receptors that determine the pharmacology and kinetics of the receptor response
MOS	KIAA1614		Predicted to be involved in centrosome cycle; establishment or maintenance of cell polarity; and regulation of cellular localization
MRPS5	KIF2B	KIF2B	Kinesin family (plus end microtubule-dependent motor)
MTX2	KIF3B	KIF3B	"
MTX2	KLF10	KLF10	Kruppel-like factor subfamily of zinc finger proteins (transcriptional regulators)
MUC12	KLF12	KLF12	"
MVB12B	KLF5	KLF5	"
MYO1E	KLF6	KLF6	"
NAALADL2	KLHDC7A	KLHDC7A	Kelch domain-containing family, unknown function
NALCN	KLHDC8A	KLHDC8A	"
NAT1	KRBOX1		Predicted to be involved in regulation of DNA-templated transcription
NBAS	L1TD1		Predicted to enable single-stranded RNA binding activity. Predicted to be involved in retrotransposition
NEDD4L	LARGE1		N-acetylglucosaminyltransferase gene family, participates in glycosylation of alpha-dystroglycan, and may carry out the synthesis of glycoprotein and glycosphingolipid sugar chains
NOL4L	LCORL		Ligand dependent nuclear receptor corepressor
NR2F1	LEMD1		Unknown
NSG1	LEPR		Receptor for leptin, an adipocyte-specific hormone that regulates body weight, and is involved in the regulation of fat metabolism
NT5C2	LEPROT		Negatively regulates leptin receptor (LEPR) cell surface expression, and thus decreases response to leptin.
NUDCD2	LINC00028		Unknown
NUDT7	LINC00342		Unknown
OLFM3	LINGO2		Predicted to act upstream of or within positive regulation of synapse assembly.
PC	LIPC		Catalyzes the hydrolysis of triglycerides and phospholipids
PCDH17	LOC100130987		Unknown
PCDH20	LPAR3		Receptor for lysophosphatidic acid (LPA), a mediator of diverse cellular activities
PCP4	LRP2		Megalin, a multi-ligand endocytic receptor
PDCD6IP	LTK		Ros/insulin receptor family of tyrosine kinases
PDE3A	LUZP1		Unknown
PEX5L	M1AP		Required for meiosis I progression during spermatogenesis
PHYHIP	MACROD2		Chromatin modifier; deacetylates O-acetyl-ADP ribose, a signaling molecule generated by the deacetylation of acetylated lysine residues in histones and other proteins
PKNOX2	MACROH2A1		Chromatin modifier; variant histone H2A which replaces conventional H2A in a subset of nucleosomes where it represses transcription
PLAG1	MAL		Integral membrane protein, could be an important component in vesicular trafficking cycling between the Golgi complex and the apical plasma membrane. Could be involved in myelin biogenesis and/or myelin function
PLAGL2	MALRD1		Enhances production and/or transport of FGF19 and thus has a role in regulation of bile acid synthesis
PLCH1	MAML3		Mastermind Like Transcriptional Coactivator, involved in Notch signaling pathway and positive regulation of transcription by RNA polymerase II
PLPPR1	MAPK10		MAP kinase family of signal transduction proteins
PLPPR5	MAPKBP1		MAP kinase signaling, scaffold protein
POFUT1	MAT2B		Regulatory subunit of S-adenosylmethionine synthetase
PPARGC1A	MCTP2		Predicted to be involved in regulation of neurotransmitter secretion
PPM1H	MEOX2		Homeobox transcription factor, acts as a negative regulator of angiogenesis
PPP1R21	MFSD1		Lysosomal dipeptide uniporter
PPP2R3A	MGA		Functions as a dual-specificity transcription factor, regulating the expression of both MAX-network and T-box family target genes. Functions as a repressor or an activator.
PPP3CC	MIS18BP1		Required for recruitment of CENPA to centromeres and normal chromosome segregation during mitosis
PRDM10	MMRN1		Multimerin, a massive, soluble protein found in platelets and in the endothelium of blood vessels; carrier protein for platelet (but not plasma) factor V/Va
PRDM15	MMS22L		Forms a complex with tonsoku-like, DNA repair protein (TONSL), and this complex recognizes and repairs DNA double-strand breaks at sites of stalled or collapsed replication forks
PRKD1	MND1		Required for proper homologous chromosome pairing and efficient cross-over and intragenic recombination during meiosis
PTHLH	MOGAT1		Acyl-CoA:monoacylglycerol acyltransferase; involved in glycerolipid synthesis and lipid metabolism
PTPN7	MOGS		This gene encodes the first enzyme in the N-linked oligosaccharide processing pathway
PUM3	MOS		Serine/threonine kinase that activates the MAP kinase cascade through direct phosphorylation of the MAP kinase activator MEK
RAC1	MRPL39	MRPL39	Mitochondrial ribosomal protein
RASSF3	MRPS2	MRPS2	"
RBFOX1	MRPS5	MRPS5	"
RBFOX3	MSRB2		Methionine-sulfoxide reductase; upon oxidative stress, may play a role in the preservation of mitochondrial integrity
RCBTB1	MTX2		Involved in transport of proteins into the mitochondrion
RHOBTB3	MUC12		Membrane-associated mucin
RIBC2	MVB12B		Component of the ESCRT-I complex, a regulator of vesicular trafficking process.
RNF111	MYO1E		Nonmuscle class I myosin of the unconventional myosin protein family, actin-based molecular motors; involved in clathrin-mediated endocytosis
RTKN	NAALADL2		N-acetylated alpha-linked acidic dipeptidase (NAALADase) gene family
SASH1	NALCN		Voltage-independent, nonselective cation channel which belongs to a family that regulates the resting membrane potential and excitability of neurons
SCHIP1	NAT1		One of two arylamine N-acetyltransferase (NAT) genes in the human genome, participates in the detoxification of a plethora of hydrazine and arylamine drugs
SDR16C5	NBAS		Involved in Golgi-to-endoplasmic reticulum (ER) retrograde transport
SEMA5B	NCAM2		Neural cell adhesion molecule
SEPHS1	NEBL		Binds to actin and plays an important role in the assembly of the Z-disk in cardiac muscle
SGCG	NEDD4L		Member of Nedd4 family of HECT domain E3 ubiquitin ligases
SGPP1	NIFK		May bind RNA and may play a role in mitosis and cell cycle progression
SH3RF1	NKAIN3		Interacts with the beta subunit of Na,K-ATPase
SHOX2	NKTR		PPIase that catalyzes the cis-trans isomerization of proline imidic peptide bonds in oligopeptides and may therefore assist protein folding
SLC17A9	NOL4L		Unknown
SLC25A26	NR2F1	NR2F1	Nuclear hormone receptor and transcriptional regulator (Coup transcription factor)
SLC4A5	NRC32	NRC32	" (mineralocorticoid receptor)
SLC5A3	NRG3		Neuregulin gene family, direct ligand for the ERBB4 tyrosine kinase receptor
SLC7A11	NRXN1		Neurexin-1, binds neuroligins
SLIT2	NSG1		Role in the recycling mechanism in neurons of multiple receptors, acts at the level of early endosomes to promote sorting of receptors toward a recycling pathway
SLITRK3	NT5C2		Hydrolase that serves as an important role in cellular purine metabolism

SLTM	NUDCD2		May regulate the LIS1/dynein pathway by stabilizing LIS1 with Hsp90 chaperone
SMARCA2	NUDT7		Nudix hydrolase involved in eliminating potentially toxic nucleotide metabolites from the cell
SPATS2	OLFM3		Olfactomedin Related ER Localized Protein
SPRED2	OTUD1		Deubiquitinating enzyme
SPTY2D1	PALLD		Cytoskeletal protein required for organizing the actin cytoskeleton
SRRT	PC		Pyruvate carboxylase
SSX2IP	PCDH17	PCDH17	Protocadherin gene family, a subfamily of the cadherin superfamily
STAG1	PCDH20	PCDH20	"
STEAP2	PCP4		Modulator of calcium-binding by calmodulin
STON2	PCSK1		Subtilisin-like proprotein convertase family, process protein and peptide precursors trafficking through regulated or constitutive branches of the secretory pathway
STX6	PDCD6IP		Multifunctional protein involved in endocytosis, multivesicular body biogenesis, membrane repair, cytokinesis, apoptosis and maintenance of tight junction integrity
SWT1	PDE3A		Member of the cGMP-inhibited cyclic nucleotide phosphodiesterase (cGI-PDE) family
SYK	PEX5L		Accessory subunit of hyperpolarization-activated cyclic nucleotide-gated (HCN) channels, regulating their cell-surface expression and cyclic nucleotide dependence
TAC1	PHYHIP		Interacts with PHYH, a peroxisomal protein involved in the alpha-oxidation of 3-methyl branched fatty acids
TBC1D12	PITRM1		ATP-dependent metalloprotease that degrades post-cleavage mitochondrial transit peptides
TBK1	PITX2		RIEG/PITX homeobox family of transcription factors
TBX18	PKNOX2		Homeodomain transcription factor
TFDP2	PLAG1	PLAG1	Transcription factor
TFEC	PLAGL2	PLAGL2	"
TFPI	PLCH1		PLC-eta family of the phosphoinositide-specific phospholipase C
THSD7A	PLPPR1	PLPPR1	Plasticity-related gene (PRG) family, mediates lipid phosphate phosphatase activity in neurons
TLE1	PLPPR5	PLPPR5	"
TM9SF4	POFUT1		Enzyme that O-fucosylates EGF-like repeats, including in the Notch protein; essential for Notch activity
TMEM108	POLD4		Component of the tetrameric DNA polymerase delta complex (Pol-delta4), plays a role in high fidelity genome replication and repair
TMEM201	POU3F2		POU-III class of neural transcription factors. The encoded protein is involved in neuronal differentiation and enhances the activation of corticotropin-releasing hormone regulated genes
TMEM86A	PPARGC1A		transcriptional coactivator that regulates the genes involved in energy metabolism
TMX3	PPM1H		Enables identical protein binding activity and phosphoprotein phosphatase activity
TNFRSF13B	PPP1R21		Component of the FERRY complex (Five-subunit Endosomal Rab5 and RNA/ribosome intermediary), functions as a RAB5A effector involved in the localization and the distribution of specific mRNAs most likely by mediating their endosomal transport.
TNK2	PPP2R3A		Regulatory subunits of the protein phosphatase 2
TNRC6B	PPP3CC		Calcineurin, a calcium-dependent, calmodulin-stimulated protein phosphatase involved in the downstream regulation of dopaminergic signal transduction
TOM1L1	PRDM10	PRDM10	Transcription factor that contains C2H2-type zinc-finger, unknown role
TRIB2	PRDM15	PRDM15	"
TRIM56	PRKD1		Serine/threonine-protein kinase that converts transient diacylglycerol (DAG) signals into prolonged physiological effects downstream of PKC
TRPS1	PTHLH		Parathyroid hormone-like hormone, a neuroendocrine peptide
TSC22D2	PTPN7		protein tyrosine phosphatase (PTP) family. PTPs are known to be signaling molecules that regulate a variety of cellular processes including cell growth, differentiation, mitotic cycle, and oncogenic transformation
TTF1	PUM3		Inhibits the poly(ADP-ribosyl)ation activity of PARP1 and the degradation of PARP1 by CASP3 following genotoxic stress
URB2	RAC1		Rac family GTPase
USP25	RASSF3		A member of a subfamily of the RAS superfamily, plasma membrane GTP-binding proteins that modulate intracellular signal transduction pathways.
VDR	RBFOX1	RBFOX1	Fox-1 family of RNA-binding proteins, regulate tissue-specific alternative splicing
VTA1	RBFOX3	RBFOX3	"
VWC2	RCBTB1		Encodes a protein that regulates angiogenesis; plays a role in maintaining healthy blood vessel development in the eye
WDR89	RELL1		Involved in positive regulation of p38MAPK cascade
WNT5A	RGMA	RGMA	Member of the repulsive guidance molecule (RGM) family
WWC1	RGMB	RGMB	"
XPNPEP1	RGPD4		Predicted to contribute to GTPase activator activity. Predicted to be involved in NLS-bearing protein import into nucleus. Predicted to be part of nuclear pore.
ZBTB47	RHOBTB3		Rab9-regulated ATPase required for endosome to Golgi transport.
ZBTB49	RIBC2		Predicted to be involved in flagellated sperm motility. Located in axonemal microtubule.
ZKSCAN2	RIOK2		Serine/threonine-protein kinase involved in the final steps of cytoplasmic maturation of the 40S ribosomal subunit.
ZNF536	RND3		Rho Family GTPase
ZNF641	RNF111	RNF111	RING finger domain-containing E3 ubiquitin ligase family, modifies transcription
ZNF728	RNF144A	RNF144A	"
ZNF737	RNF2	RNF2	"
ZNF804B	RPL10L		Ribosome protein L10
	RTCB		Catalytic subunit of the tRNA-splicing ligase complex
	RTKN		Scaffold protein that interacts with GTP-bound Rho proteins to inhibit their GTPase activity
	SALL2		Transcription factor that plays a role in eye development
	SASH1		Scaffold protein involved in the TLR4 signaling pathway
	SCHIP1		Predicted to be involved in positive regulation of hippo signaling.
	SDR16C5		Short-chain alcohol dehydrogenase/reductase superfamily of proteins and is involved in the oxidation of retinol to retinaldehyde
	SEMA5B		Semaphorin protein family which regulates axon growth during development of the nervous system
	SEPHS1		Enzyme that synthesizes selenophosphate from selenide and ATP
	SGCG		Component of the sarcoglycan complex, a subcomplex of the dystrophin-glycoprotein complex which forms a link between the F-actin cytoskeleton and the extracellular matrix of muscle
	SGK1		Serum/Glucocorticoid Regulated Kinase, plays an important role in cellular stress response
	SGPP1		Sphingosine-1-phosphate (S1P) is a bioactive sphingolipid metabolite that regulates diverse biologic processes; SGPP1 catalyzes the degradation of S1P via salvage and recycling of sphingosine into long-chain ceramides
	SH3RF1		Ubiquitin-protein ligase involved in protein sorting at the trans-Golgi network
	SHOX2		Homeodomain transcription factor
	SIRPA		Signal-regulatory-protein (SIRP) family, multifunctional
	SLC17A9		Participates in the vesicular uptake, storage, and secretion of adenoside triphosphate (ATP) and other nucleotides
	SLC25A26		Transport of S-adenosylmethionine (SAM) into the mitochondria
	SLC4A5		Mediates sodium- and bicarbonate-dependent electrogenic sodium bicarbonate cotransport
	SLC5A3		Electrogenic Na(+)-coupled sugar symporter that actively transports myo-inositol and its stereoisomer scyllo-inositol across the plasma membrane
	SLC7A11		Heterodimer with SLC3A2, that functions as an antiporter by mediating the exchange of extracellular anionic L-cystine and intracellular L-glutamate across the cellular plasma membrane
	SLIT2		Slit family of secreted glycoproteins, roles in axon guidance and neuronal migration, ligand of ROBO receptors
	SLITRK3	SLITRK3	SLITRK family of structurally related transmembrane proteins that are involved in controlling neurite outgrowth
	SLITRK5	SLITRK5	"
	SLTM		When overexpressed, acts as a general inhibitor of transcription that eventually leads to apoptosis
	SMARCA2		SWI/SNF family of proteins with helicase and ATPase activities and are thought to regulate transcription of certain genes by altering the chromatin structure around those genes
	SOSTDC1		Sclerostin family member, functions as a bone morphogenetic protein (BMP) antagonist
	SOX17		Transcription factor that Inhibits Wnt signaling, activates Notch signaling
	SPAG4		Involved in spermatogenesis
	SPATS2		Unknown
	SPRED2		Negatively regulates Ras signaling pathways and downstream activation of MAP kinases
	SPTY2D1		Histone chaperone
	SRRT		Acts as a mediator between the cap-binding complex (CBC) and the primary microRNAs (miRNAs) processing machinery during cell proliferation
	SSH3		Protein phosphatase which may play a role in the regulation of actin filament dynamics
	SSX2IP		Belongs to an adhesion system, which plays a role in the organization of homotypic, interneuronal and heterotypic cell-cell adherens junctions (AJs).
	ST6GAL2		Sialyltransferase, transfers sialic acid from the donor of substrate CMP-sialic acid to galactose containing acceptor substrate
	STAG1		Component of cohesin complex, a complex required for the cohesion of sister chromatids after DNA replication
	STEAP2		Integral membrane protein that functions as a NADPH-dependent ferric-chelate reductase
	STK35		Serine/threonine protein kinase
	STON2		Adapter protein involved in endocytic machinery
	STX6		Targets endosomes to the trans-Golgi network, and may therefore function in retrograde trafficking
	SWT1		Transcription factor
	SYK		Non-receptor type Tyr protein kinase
	TAC1		Encodes four products of the tachykinin peptide hormone family, substance P and neurokinin A, as well as the related peptides, neuropeptide K and neuropeptide gamma
	TAF5L		Functions as a component of the PCAF complex, capable of efficiently acetylating histones in a nucleosomal context
	TBC1D12		RAB11A-binding protein that plays a role in neurite outgrowth; enables GTPase activity
	TBK1		Serine/threonine kinase that plays an essential role in regulating inflammatory responses to foreign agents
	TBL1XR1		F-box-like protein involved in the recruitment of the ubiquitin/19S proteasome complex to nuclear receptor-regulated transcription units; plays an essential role in transcription activation mediated by nuclear receptors.
	TBX18		Tbx1 sub-family, acts as a transcriptional repressor by antagonizing transcriptional activators in the T-box family
	TEKT4		Microtubule inner protein (MIP) part of the dynein-decorated doublet microtubules (DMTs) in cilia and flagellar axoneme
	TFDP2		Binds DNA cooperatively with E2F family members to stimulate transcription
	TFEC		Microphthalmia (MiT) family of basic helix-loop-helix leucine zipper transcription factors
	TFPI		Kunitz-type serine protease inhibitor that regulates the tissue factor (TF)-dependent pathway of blood coagulation
	THSD7A		Plays a role in actin cytoskeleton rearrangement; soluble form promotes endothelial cell migration and filopodia formation during sprouting angiogenesis
	TLE1		Transcriptional corepressor involved in WNT and Notch signaling
	TLR2		Toll-like receptor (TLR) family, role in pathogen recognition and activation of innate immunity
	TM2D1		May participate in amyloid-beta-induced apoptosis via its interaction with beta-APP42
	TM9SF4		Multifunctional protein located in Golgi apparatus and early endosome
	TMEM108		Multifunctional protein located in endosomes
	TMEM201		Proposed to be involved in actin-dependent nuclear movement via association with transmembrane actin-associated nuclear (TAN) lines which are bound to F-actin cables and couple the nucleus to retrograde actin flow
	TMEM255B		Unknown
	TMEM26		Selective surface protein marker of beige adipocytes, which may coexist with classical brown adipocytes in brown adipose tissue
	TMEM86A		Enzyme involved in lysoplasmalogen metabolism in the adipocyte tissue and macrophages
	TMX3		Disulfide isomerase (PDI) family of endoplasmic reticulum (ER) proteins that catalyze protein folding and thiol-disulfide interchange reactions
	TNFRSF13B		Tumor necrosis factor (TNF) receptor superfamily; mediates calcineurin-dependent activation of NF-AT, as well as activation of NF-kappa-B and AP-1; involved in the stimulation of B- and T-cell function and the regulation of humoral immunity
	TNK2		Non-receptor tyrosine-protein and serine/threonine-protein kinase that is implicated in cell spreading and migration, cell survival, cell growth and proliferatio
	TNRC6B		Plays a role in RNA-mediated gene silencing by both micro-RNAs (miRNAs) and short interfering RNAs (siRNAs)
	TOM1L1		Probable adapter protein involved in signaling pathways. Interacts with the SH2 and SH3 domains of various signaling proteins when it is phosphorylated
	TRIB2		Tribbles family, interacts with MAPK kinases and regulates activation of MAP kinases
	TRIM56		Tripartite motif (TRIM) family, E3 ubiquitin-protein ligase
	TRPS1		Transcriptional repressor. Binds specifically to GATA sequences and represses expression of GATA-regulated genes
	TSC22D2		Involved in negative regulation of cell cycle
	TSN		DNA-binding protein that specifically recognizes consensus sequences at the breakpoint junctions in chromosomal translocation
	TSPAN14		Required for ADAM10 exit from the endoplasmic reticulum and for enzymatic maturation and trafficking to the cell surface as well as substrate specificity; negatively regulates ADAM10-mediated cleavage of GP6
	TTF1		Transcription termination factor that is localized to the nucleolus and plays a critical role in ribosomal gene transcription
	TUBGCP3		Gamma-tubulin complex necessary for microtubule nucleation at the centrosome
	TYRO3		Receptor tyrosine kinase that transduces signals from the extracellular matrix into the cytoplasm by binding to several ligands including TULP1 or GAS6
	URB2		Involved in regulation of signal transduction by p53 class mediator and ribosome biogenesis
	USP13	USP13	De-ubiquitinase
	USP25	USP25	"
	VDR		Vitamin D3 receptor, a member of the nuclear hormone receptor superfamily
	VTA1		Involved in trafficking of the multivesicular body
	VWC2		BMP antagonist

WDR89		Unknown
WNT2B	WNT2B	Wingless-type MMTV integration site (WNT) family of highly conserved, secreted signaling factors, function in the Wnt signaling pathway
WNT5A	WNT5A	"
WWC1		Acts as a scaffolding protein, facilitating protein-protein interactions within the Hippo pathway, regulated by Notch
XKR4		Phospholipid scramblase that promotes phosphatidylserine exposure on apoptotic cell surface
XPNPEP1		Metalloaminopeptidase that plays a role in degradation and maturation of tachykinins, neuropeptides, and peptide hormones
ZBTB47	ZBTB47	Zinc Finger And BTB Domain Containing transcription factor
ZBTB49	ZBTB49	"
ZFAT		Transcriptional regulator involved in apoptosis and cell survival
ZKSCAN2		Transcriptional regulator
ZMAT3		Target gene of p53/TP53
ZNF514		Zinc finger family of transcriptional regulators, unknown role
ZNF536		Zinc finger family of transcriptional regulators, transcriptional repressor that negatively regulates neuron differentiation by repressing retinoic acid-induced gene transcription
ZNF641		Zinc finger family of transcriptional regulators, unknown role
ZNF728		Zinc finger family of transcriptional regulators, unknown role
ZNF737		Zinc finger family of transcriptional regulators, unknown role
ZNF804B		Zinc finger family of transcriptional regulators, unknown role

408 total genes

41 paralogous groups
