# Supplementary material for: Pharmacogenomics of steroid-induced ocular hypertension: relationship to high-tension glaucomas and new pathophysiologic insight
Source: medRxiv. 2025 Aug 13:2025.08.11.25333245. Preprint. [Version 1] doi: 10.1101/2025.08.11.25333245 (PMC12363710; doi:10.1101/2025.08.11.25333245)
Supplement: Supplement 6 — Table S5. Prioritized Target Gene Validation [file media-6.pdf]

Supplementary Table S5. Prioritized Target Gene Validation
GC-regulation of prioritized target genes

NOTES

Alphabetical ordering of the full prioritized gene list highlights functionally similar paralogues or gene family members by grouping them together.

We counted 41 such paralogous groups with shared function.

HEADERS

AOP: aqueous outflow pathway; DEG: differentially expressed gene;
eQTL: expression quantitative trait locus; TM: trabecular meshwork
DEGs in TM cells: column adding up all glucocorticoid-regulated genes in TM cell dataset;
Count GC-DEG: column adding up all such genes, total at bottom;
GC-Regulated in silico: column adding up all GC-regulated genes in DAVID dataset, total at bottom; New DEGs in silico: column adding up all genes in DAVID dataset not already identified in TM cell dataset, total at bottom;
DEGs overlap: column adding up all GC-regulated genes in both TM cell and DAVID dataset, total at bottom

	GC-regulated count				
	DEGs in TM cells	New DEGs paired eye	Paired eye overlap	New DEGs in silico	In silico overlap
Prioritized genes					
ABCG2	1				1
ACADSB				1	
ACSF2					
ACSL3				1	
ADAM10	1				
ADGRG6				1	
ADGRL3				1	
ADGRL4					
ADIPOR2				1	
AEBP2				1	
AGAP1				1	
AGR3					
AGTR1					
AHCTF1	1				1
AHR					
ALDH5A1	1				1
ALDH8A1					
ALK				1	
AMY2A					
AMY2B					
AMZ2					
ANKS1B				1	
ANO4				1	
ANO9					
ANP32D	1				
AOAH					
AP1AR					
AP2A2				1	
ARAP2					
ARHGAP21		1			
ARHGEF26	1				1
ARHGEF28					
ARID5B				1	
ARRDC3	1				1
ARVCF	1				1
ASB3					
ASB8	1				1
ASNS	1				1
ASPH	1				1
ASXL1				1	
ATP4B				1	
ATP8A1					
ATP8B1	1				1
ATXN1	1				1
AVPR1A					
AZIN1				1	
B4GALNT3					
B9D1		1			
BEND7	1				1

BTRC	1				1
C1D					
C1orf21	1				1
C4orf19				1	
C6orf118					
CAMSAP1					
CAV2	1				1
CBR4					
CCDC180					
CCDC184					
CCDC77				1	
CCDC91	1				1
CCNB2	1				
CCNG1	1				
CCR6					
CCSER1				1	
CCSER2				1	
CCZ1					
CCZ1B					
CDC42BPA	1				1
CDCA2	1		1		1
CDH2	1				1
CDH9				1	
CDK5RAP3					
CDON				1	
CELSR1				1	
CFAP99					
CFTR		1			
CHAC1	1				
CHCHD7					
CHD4				1	
CLEC11A				1	
CMC1					
CNTN4				1	
CNTN5				1	
CNTN6	1				
CNTNAP2	1				1
CNTNAP4		1			
COL11A1	1		1		1
COPZ2	1				1
CRTC1				1	
CSMD1				1	
CSMD3				1	
CTBP2				1	
CTSV					
CUZD1					
DCANP1					
DCT			1	1	
DIAPH3	1				1
DIDO1	1				1
DIRAS2					
DLC1	1				1
DLGAP2					
DLL4					
DMRT2					
DNASE2B				1	
DPP10				1	
DSCAM				1	
DSG1					
DSG3					
DYNC2H1	1				1
DYRK1A				1	
E2F3					
EBF2				2	
EDEM3	1				
EFHB					
EIF2AK4				2	
EIF3IP1					
ENDOD1					
EPHB1				1	
ERGIC3	1				1
ESD					
ETAA1					

ETNK1	1				
EVX2				2	
FAM155A	1				
FAM24B					
FAM76B					
FAXDC2					
FBXO11				1	
FBXO7	1				1
FERMT1					
FFAR2					
FMN1					
FOXN2	1		1		1
FRMD3		1			
FST	1				
FSTL5	1				1
GABRG2				1	
GABRP		1			
GAL					
GALNT10	1				
GAS2L3	1				1
GATAD2A				1	
GLRX					
GMNC	1				
GMPS				1	
GOT2	1				
GPAT2				1	
GPC5	1				1
GPC6				1	
GPLD1				1	
GTF2B				1	
GYPC					
HAAO					
HAS2				1	
HCK	1				
HDAC4	1				
HDAC9			1	1	
HMCN1				1	
HOXD13				1	
HS3ST1	1				1
HS3ST4			1	1	
HS6ST1	1		1		
HTR2A				1	
HYLS1	1				
ID2	1				
IDUA	1				1
IGF2BP3				1	
INTU	1				
IQCJ-SCHIP1	1				1
IQCM				1	
ITPR2					
JADE1					
JAG2				2	
JDP2					
JMJD7	1				
JMJD7-PLA2G4B	1				1
KAZALD1				1	
KCND3			1	1	
KCNE4	1				1
KCNH8				1	
KCNIP1	1				1
KCNIP3				1	
KCNIP4				1	
KCTD8				1	
KIAA1614				1	
KIF2B					
KIF3B	1				1
KLF10				1	
KLF12	1				
KLF5	1				
KLF6			1	1	
KLHDC7A	1				1
KLHDC8A	1				1
KRBOX1	1				

L1TD1					
LARGE1				1	
LCORL					
LEMD1				1	
LEPR					
LEPROT					
LINC00028				1	
LINC00342	1				1
LINGO2					
LIPC	1				
LOC100130987				1	
LPAR3				1	
LRP2				1	
LTK					
LUZP1				1	
M1AP					
MACROD2				1	
MACROH2A1				1	
MAL				1	
MALRD1				1	
MAML3				1	
MAPK10					
MAPKBP1				1	
MAT2B	1				1
MCTP2	1				1
MEOX2	1		1		1
MFSD1				1	
MGA					
MIS18BP1	1				
MMRN1	1				1
MMS22L					
MND1					
MOGAT1				1	
MOGS	1				
MOS					
MRPL39					
MRPS2					
MRPS5					
MSRB2					
MTX2					
MUC12		1			
MVB12B				1	
MYO1E					
NAALADL2					
NALCN	1				1
NAT1	1				1
NBAS					
NCAM2					
NEBL					
NEDD4L				1	
NIFK				1	
NKAIN3				1	
NKTR	1				1
NOL4L					
NR2F1				1	
NRC32	1				1
NRG3				1	
NRXN1					
NSG1					
NT5C2				1	
NUDCD2				1	
NUDT7				1	
OLFM3				1	
OTUD1	1				
PALLD	1		1		
PC					
PCDH17				1	
PCDH20	1		1		1
PCP4				1	
PCSK1				1	
PDCD6IP	1				1
PDE3A					
PEX5L	1				

PHYHIP				1	
PITRM1				1	
PITX2	1				1
PKNOX2				1	
PLAG1				1	
PLAGL2	1				1
PLCH1	1				
PLPPR1					
PLPPR5					
POFUT1				1	
POLD4					
POU3F2				1	
PPARGC1A	1				1
PPM1H				1	
PPP1R21	1				1
PPP2R3A	1				1
PPP3CC				1	
PRDM10					
PRDM15				1	
PRKD1			1	1	
PTHLH				1	
PTPN7	1				1
PUM3	1				1
RAC1					
RASSF3		1			
RBFOX1	1		1		1
RBFOX3					
RCBTB1	1				1
RELL1	1				1
RGMA				1	
RGMB					
RGPD4	1				1
RHOBTB3	1				
RIBC2				1	
RIOK2	1				1
RND3				1	
RNF111					
RNF144A	1				1
RNF2					
RPL10L				1	
RTCB				1	
RTKN				1	
SALL2					
SASH1				1	
SCHIP1					
SDR16C5	1				1
SEMA5B	1				1
SEPHS1					
SGCG				1	
SGK1					
SGPP1	1				1
SH3RF1				1	
SHOX2					
SIRPA	1				1
SLC17A9					
SLC25A26	1				
SLC4A5					
SLC5A3				1	
SLC7A11				1	
SLIT2	1				1
SLITRK3					
SLITRK5	1				1
SLTM					
SMARCA2					
SOSTDC1					
SOX17	1				1
SPAG4				1	
SPATS2	1				
SPRED2				1	
SPTY2D1				1	
SRRT					
SSH3	1				
SSX2IP					

ST6GAL2					
STAG1		1			
STEAP2					
STK35	1		1		1
STON2	1				1
STX6				1	
SWT1	1				1
SYK	1				1
TAC1					
TAF5L					
TBC1D12					
TBK1					
TBL1XR1	1		1		
TBX18					
TEKT4	1				
TFDP2	1				
TFEC	1				
TFPI				1	
THSD7A					
TLE1	1				
TLR2				1	
TM2D1	1				1
TM9SF4	1				
TMEM108					
TMEM201	1				
TMEM255B					
TMEM26	1				1
TMEM86A					
TMX3					
TNFRSF13B	1		1		
TNK2				1	
TNRC6B				1	
TOM1L1				1	
TRIB2	1				1
TRIM56	1				1
TRPS1	1				
TSC22D2	1				1
TSN	1				
TSPAN14				1	
TTF1				1	
TUBGCP3					
TYRO3					
URB2					
USP13					
USP25	1				1
VDR					
VTA1	1				1
VWC2				1	
WDR89	1				1
WNT2B					
WNT5A					
WWC1					
XKR4	1				1
XPNPEP1	1				
ZBTB47	1				1
ZBTB49				1	
ZFAT	1				1
ZKSCAN2				1	
ZMAT3					
ZNF514					
ZNF536					
ZNF641	1				1
ZNF728	1				
ZNF737					
ZNF804B					
	134	9	17	133	88
GC-regulated count:	33%	2%	4%	33%	22%
Percent of total: red eye:		26			

Sum GC-regulated in silico:	221
Percent GC-regulated in silico	54%
Total GC-regulated:	276
Percent GC-regulated total:	68%
Total prioritized target genes:	406

Supplementary Table S5. Prioritized Target Gene Validation

GC-regulated differentially expressed genes (DEGs) from a human paired eye study

From Kathirvel et al. (96)

Responders all DEGs	Responders down-regulated DEGs	Responders up-regulated DEGs	Non-responders all DEGs	Non-responders down-regulated DEGs	Non-responders up-regulated DEGs	All DEGs that match GWAS prioritized genes	Responder DEGs that match GWAS prioritized genes
AATK	AATK	ABCA6	ABCA6	ADM2	ABCA6	ARHGEF26	BEND7
ABCA6	ABCB1	ABCA9	ACA59	ADRA2A	ACA59	BEND7	COL11A1
ABCA9	ABI3	ABRA	ADH1A	AQP1	ADH1A	CDH2	COPZ2
ABCB1	ACP5	ACA59	ADH1B	ARHGAP9	ADH1B	CHAC1	DIAPH3
ABI3	ACPP	ACTG1P3	ADH4	ARSI	ADH4	COL11A1	FRMD3
ABRA	ADAMTS9-AS1	ADH1A	ADM2	ASPN	ADRA1B	COPZ2	FST
ACA59	ADRA2A	ADH1B	ADRA1B	ATP1A3	AFAP1L1	DIAPH3	HMCN1
ACP5	ADRB1	ADH4	ADRA2A	BEX2	AIM1	FRMD3	KCNE4
ACPP	AGR2	ADRA1B	AFAP1L1	BST2	AKR1B15	FST	KLHDC7A
ACTG1P3	ANO7	AFF2	AIM1	C1orf87	ALDH1L1-AS2	GAL	MFSD1
ADAMTS9-AS1	AOC1	ANGPTL1	AKR1B15	C2orf40	ALOX15B	HMCN1	MVB12B
ADH1A	AOC3	ANGPTL5	ALDH1L1-AS2	CA3	ANGPTL1	HS6ST1	PC
ADH1B	AOC4P	ANGPTL7	ALOX15B	CADM1	ANGPTL5	HTR2A	PCP4
ADH4	AP1M2	ANO3	ANGPTL1	CCND2	ANGPTL7	KCNE4	RBFOX3
ADRA1B	AQP1	AOX1	ANGPTL5	CCND2-AS1	ANKRD2	KLHDC7A	STEAP2
ADRA2A	AQP3	APOD	ANGPTL7	CCND2-AS2	AOX1	MFSD1	STON2
ADRB1	AQP5	B3GALT2	ANKRD2	CERS1	APCDD1	MVB12B	TNK2
AFF2	ARSI	BCRP1	AOX1	CHAC1	APOD	PC	
AGR2	ASCL2	BHLHE22	APCDD1	CILP2	ARHGEF26	PCP4	
ANGPTL1	ATAD3C	C1QTNF7	APOD	CLDN1	B3GNT7	PTHLH	
ANGPTL5	ATP1A2	C5AR2	AQP1	CNGA1	C3	RBFOX1	
ANGPTL7	ATP8B4	CCDC54	ARHGAP9	CNTN6	CHI3L2	RBFOX3	
ANO3	AZGP1	CHRM2	ARHGEF26	COL19A1	CHRNA5	STEAP2	
ANO7	B3GAT1	CPM	ARSI	CPA4	CLSTN2	STON2	
AOC1	B4GALNT3	CYP51A1P2	ASPN	CTNNA3	CNTN1	TBX18	
AOC3	BCAS1	CYP7B1	ATP1A3	ELOVL2-AS1	CPM	TNK2	
AOC4P	BCL11B	DKK2	B3GNT7	EPHA3	CRISPLD2		
AOX1	BCL6B	FAM46B	BEX2	ERICH2	DGKG		
AP1M2	BMP7	FAM65C	BST2	FNDC1	DKK2		
APOD	BST2	FGD4	C1orf87	FST	DUSP5		
AQP1	BTC	FGF14	C2orf40	GRIA2	EDNRB		
AQP3	C10orf128	FGFR4	C3	GRID2	FAM107A		
AQP5	C1orf115	FHL5	CA3	GRM5	FAM150B		
ARSI	C1orf116	FKBP5	CADM1	IGFL2	FAM46B		
ASCL2	C1QC	FMO2	CCND2	IL32	FGFR4		
ATAD3C	C1QTNF2	FPR1	CCND2-AS1	INA	FHL5		
ATP1A2	C2orf40	FRG2C	CCND2-AS2	IPCEF1	FKBP5		
ATP8B4	C2orf54	FRMD3	CERS1	KAL1	FMO2		
AZGP1	C3orf80	GALNT15	CHAC1	KCNMB2	FPR1		
B3GALT2	C5orf38	GIP	CHI3L2	KCNS1	GALNT15		
B3GAT1	C7	GJA5	CHRNA5	KCTD16	GGT5		
B4GALNT3	CACNG4	GPX3	CILP2	KIAA1211	GPM6B		
BCAS1	CALML3	H19	CLDN1	KLHDC7B	GPRC5B		
BCL11B	CAMK2A	HIF3A	CLSTN2	KRT17	GRK5-IT1		
BCL6B	CAMK2B	HNRNPA3P11	CNGA1	KRT17P1	H19		
BCRP1	CAMSAP3	HSPD1P11	CNTN1	KRT23	HEYL		
BHLHE22	CAPN11	IGF2	CNTN6	LAMP3	HIF3A		
BMP7	CASKIN1	ITGA10	COL19A1	LFNG	HLX		
BST2	CBFA2T3	KCNE1	CPA4	LINC01133	HMGN2P15		
BTC	CBLC	KIAA1456	CPM	LPHN3	IGF2		
C10orf128	CCDC64B	KLF15	CRISPLD2	LRRC15	IGF2-AS		
C1orf115	CCDC88C	KRT18P62	CTNNA3	LRRN4CL	INHBB		
C1orf116	CCL5	LDHAL6B	DGKG	LTK	ISM1		

C1QC	CCM2L	LEP	DKK2	MXRA5Y	KIF5C
C1QTNF2	CCR1	LINC00547	DUSP5	MYHAS	LGI3
C1QTNF7	CD177	LINC00664	EDNRB	NGEF	LINC00525
C2orf40	CD34	LINC00702	ELOVL2-AS1	NGFR	LINC00702
C2orf54	CD38	LINC01088	EPHA3	NMNAT2	LINC00704
C3orf80	CD7	LOC100421166	ERICH2	NPY6R	LINC00968
C5AR2	CD74	LSP1	FAM107A	NTM	LINC01088
C5orf38	CD79B	MAOA	FAM150B	PADI2	LSP1
C7	CDH1	MAP1LC3C	FAM46B	PI16	MAOA
CACNG4	CDH22	MARCH10	FGFR4	PLXNC1	METTL7A
CALML3	CDH3	MIR5690	FHL5	PRSS35	MIR5685
CAMK2A	CDH5	MOB3B	FKBP5	RAB39B	MIRLET7D
CAMK2B	CEBPA	MRO	FMO2	RARRES2	MOB3B
CAMSAP3	CEMIP	MYOC	FNDC1	RBFOX1	MREG
CAPN11	CERS1	NEDD9	FPR1	RDH12	MRO
CASKIN1	CFD	NKAIN2	FST	RGS16	MTFP1
CBFA2T3	CHGB	NPSR1-AS1	GALNT15	RGS7BP	MTSS1
CBLC	CHI3L1	NTRK2	GGT5	RIMS2	MYBPHL
CCDC54	CHIT1	OCA2	GPM6B	RIMS3	MYOC
CCDC64B	CHODL	OLAH	GPRC5B	SEMA3D	NCAM1-AS1
CCDC88C	CHRDL2	P2RY14	GRIA2	SEMA6B	NEDD9
CCL5	CHRM1	PDK4	GRID2	SLC14A1	NR0B1
CCM2L	CHRNA2	PDLIM1P4	GRK5-IT1	SLC24A2	NRCAM
CCR1	CHRNA4	PER1	GRM5	SLC7A5	OCA2
CD177	CILP2	PLCE1-AS1	H19	TAC3	OLAH
CD34	CITED1	PPP1R14A	HEYL	TMEM63C	P2RY14
CD38	CLCA2	PRODH	HIF3A	TNFSF15	PDK4
CD7	CLDN3	PRR33	HLX	TNFSF18	PER1
CD74	CLDN4	PTK2B	HMGN2P15	TNNT2	PLIN5
CD79B	CLEC14A	RAPGEF5	IGF2	UNC5B	PMEL
CDH1	CNFN	RN7SKP69	IGF2-AS	VCAN	PNMT
CDH22	COL14A1	RN7SL608P	IGFL2	VCAN-AS1	POM121L9P
CDH3	COL15A1	RNA5SP111	IL32	VNN1	PPP1R14A
CDH5	COL17A1	RPL7P57	INA	WNT2	PRODH
CEBPA	COL9A1	SAA1	INHBB		PRR33
CEMIP	COL9A3	SAA2	IPCEF1		PTGDR2
CERS1	CPA3	SAA4	ISM1		PTHLH
CFD	CPA4	SAMHD1	KAL1		RAMP2
CHGB	CPAMD8	SCN3A	KCNMB2		RAMP2-AS1
CHI3L1	CPLX1	SEMG1	KCNS1		RGCC
CHIT1	CPNE4	SLC16A10	KCTD16		RN7SKP97
CHODL	CPXM1	SLC16A12	KIAA1211		RPA4
CHRDL2	CRABP2	SLC38A11	KIF5C		RPL23AP81
CHRM1	CTSH	ST7-AS2	KLHDC7B		SAA1
CHRM2	CUX2	SYNDIG1	KRT17		SAA2
CHRNA2	CWH43	TIMP4	KRT17P1		SAMHD1
CHRNA4	CX3CL1	TLDC2	KRT23		SCARA5
CILP2	CXADR	TNFAIP8L3	LAMP3		SCN3A
CITED1	CXCL13	TSC22D3	LFNG		SFTPC
CLCA2	CXCL14	TUSC5	LGI3		SIX2
CLDN3	CXorf36	UBE2CP1	LINC00525		SLC16A10
CLDN4	CYBB	USP2	LINC00702		SLC16A12
CLEC14A	CYP24A1	XRCC6P2	LINC00704		SOAT2
CNFN	CYP26A1	ZBTB16	LINC00968		SOX13
COL14A1	CYTH4		LINC01088		SPP1
COL15A1	DACT2		LINC01133		STAR
COL17A1	DENND1C		LPHN3		STEAP4
COL9A1	DES		LRRC15		STOX1
COL9A3	DIO3		LRRN4CL		SYN2
CPA3	DIO3OS		LSP1		SYTL4
CPA4	DIRAS3		LTK		TBXAS1
CPAMD8	DOC2B		MAOA		TIMP4
CPLX1	DRAXIN		METTL7A		TLDC2

CPM	DSC2	MIR5685	TLE2
CPNE4	DTX1	MIRLET7D	TLE6
CPXM1	ECSCR	MOB3B	TMOD1
CRABP2	EDN3	MREG	TNNT3
CTSH	EFCC1	MRO	TRAV39
CUX2	EFNA1	MTFP1	TRPC3
CWH43	EHF	MTSS1	TRPV6
CX3CL1	ELF3	MXRA5Y	TXNRD1
CXADR	ELFN1	MYBPHL	USP2
CXCL13	ELFN2	MYHAS	VAV3
CXCL14	ELOVL2	MYOC	WSCD1
CXorf36	ELOVL7	NCAM1-AS1	XRCC6P2
CYBB	EMID1	NEDD9	ZBTB16
CYP24A1	ENPP6	NGEF	
CYP26A1	EPCAM	NGFR	
CYP51A1P2	ESPN	NMNAT2	
CYP7B1	ESRP1	NPY6R	
CYTH4	EVPL	NR0B1	
DACT2	EVX2	NRCAM	
DENND1C	EXOC3L4	NTM	
DES	FAM110D	OCA2	
DIO3	FAM19A3	OLAH	
DIO3OS	FAM201A	P2RY14	
DIRAS3	FAM3B	PADI2	
DKK2	FAM3D	PDK4	
DOC2B	FAM46C	PER1	
DRAXIN	FAR2P1	PI16	
DSC2	FAR2P2	PLIN5	
DTX1	FBN3	PLXNC1	
ECSCR	FGR	PMEL	
EDN3	FLT4	PNMT	
EFCC1	FNDC1	POM121L9P	
EFNA1	FOLH1	PPP1R14A	
EHF	FOXA1	PRODH	
ELF3	FOXQ1	PRR33	
ELFN1	FST	PRSS35	
ELFN2	FXYD2	PTGDR2	
ELOVL2	FXYD3	PTHLH	
ELOVL7	FXYD6	RAB39B	
EMID1	FZD10	RAMP2	
ENPP6	FZD10-AS1	RAMP2-AS1	
EPCAM	G0S2	RARRES2	
ESPN	GALNT16	RBFOX1	
ESRP1	GAP43	RDH12	
EVPL	GATA5	RGCC	
EVX2	GGT6	RGS16	
EXOC3L4	GIMAP1	RGS7BP	
FAM110D	GIMAP4	RIMS2	
FAM19A3	GIMAP5	RIMS3	
FAM201A	GIMAP6	RN7SKP97	
FAM3B	GIMAP7	RPA4	
FAM3D	GIMAP8	RPL23AP81	
FAM46B	GJB1	SAA1	
FAM46C	GJB2	SAA2	
FAM65C	GLB1L2	SAMHD1	
FAR2P1	GNA15	SCARA5	
FAR2P2	GNG4	SCN3A	
FBN3	GOLT1A	SEMA3D	
FGD4	GP2	SEMA6B	
FGF14	GPR143	SFTPC	
FGFR4	GPR20	SIX2	
FGR	GPR56	SLC14A1	
FHL5	GRAMD4P2	SLC16A10	

FKBP5	GREB1	SLC16A12
FLT4	GREM2	SLC24A2
FMO2	GRHL2	SLC7A5
FNDC1	GRIK4	SOAT2
FOLH1	GRIN1	SOX13
FOXA1	GYLTL1B	SPP1
FOXQ1	HES2	STAR
FPR1	HGD	STEAP4
FRG2C	HID1	STOX1
FRMD3	HLA-DMB	SYN2
FST	HLA-DOA	SYTL4
FXYD2	HLA-DPA1	TAC3
FXYD3	HLA-DQA1	TBXAS1
FXYD6	HLA-DQA2	TIMP4
FZD10	HLA-DQB1	TLDC2
FZD10-AS1	HLA-DRA	TLE2
G0S2	HLA-DRB1	TLE6
GALNT15	HMCN2	TMEM63C
GALNT16	HMGCS2	TMOD1
GAP43	HMGN2P46	TNFSF15
GATA5	HOTTIP	TNFSF18
GGT6	HOXA10	TNNT2
GIMAP1	HOXA11	TNNT3
GIMAP4	HOXA11-AS	TRAV39
GIMAP5	HOXA13	TRPC3
GIMAP6	HOXA3	TRPV6
GIMAP7	HOXA5	TXNRD1
GIMAP8	HOXA7	UNC5B
GIP	HOXA9	USP2
GJA5	HOXB13	VAV3
GJB1	HOXD10	VCAN
GJB2	HOXD13	VCAN-AS1
GLB1L2	HOXD3	VNN1
GNA15	HOXD8	WNT2
GNG4	HOXD9	WSCD1
GOLT1A	HPD	XRCC6P2
GP2	HPN	ZBTB16
GPR143	HPSE2	
GPR20	HSH2D	
GPR56	HSPA12B	
GPX3	HSPA6	
GRAMD4P2	ICAM2	
GREB1	IGF1	
GREM2	IGHG3	
GRHL2	IGHM	
GRIK4	IGHV4-34	
GRIN1	IGHV4-39	
GYLTL1B	IGJ	
H19	IGKV1-5	
HES2	IGKV3-11	
HGD	IGLC3	
HID1	IGLV1-40	
HIF3A	IGSF9	
HLA-DMB	IRF8	
HLA-DOA	IRX4	
HLA-DPA1	ISL1	
HLA-DQA1	ITGAX	
HLA-DQA2	ITM2A	
HLA-DQB1	JPH3	
HLA-DRA	JPH4	
HLA-DRB1	KCNH2	
HMCN2	KCNH6	
HMGCS2	KCNK5	

HMGN2P46	KCNN3
HNRNPA3P11	KCNQ1
HOTTIP	KIAA1210
HOXA10	KIAA1211L
HOXA11	KIAA1324
HOXA11-AS	KIF12
HOXA13	KIF1A
HOXA3	KL
HOXA5	KLK11
HOXA7	KLK2
HOXA9	KLK3
HOXB13	KLK4
HOXD10	KLK7
HOXD13	KLKP1
HOXD3	KREMEN2
HOXD8	KRT13
HOXD9	KRT14
HPD	KRT15
HPN	KRT18
HPSE2	KRT23
HSH2D	KRT5
HSPA12B	KRT6A
HSPA6	KRT79
HSPD1P11	KRT8
ICAM2	LAD1
IGF1	LAMP3
IGF2	LAMP5
IGHG3	LCK
IGHM	LCN2
IGHV4-34	LCN6
IGHV4-39	LCP1
IGJ	LGALS7B
IGKV1-5	LGR6
IGKV3-11	LINC00086
IGLC3	LINC00261
IGLV1-40	LINC00668
IGSF9	LINC00890
IRF8	LINC00964
IRX4	LINC01018
ISL1	LINC01297
ITGA10	LINC01315
ITGAX	LMAN1L
ITM2A	LMO2
JPH3	LRG1
JPH4	LRRC15
KCNE1	LRRC26
KCNH2	LYPD3
KCNH6	LYZ
KCNK5	MAL2
KCNN3	MALL
KCNQ1	MAOB
KIAA1210	MARVELD3
KIAA1211L	MB
KIAA1324	MCF2L
KIAA1456	MCHR1
KIF12	MEOX1
KIF1A	MEST
KL	MIR200A
KLF15	MIR205HG
KLK11	MIR3189
KLK2	MIR429
KLK3	MLC1
KLK4	MMP7

KLK7	MMP9
KLKP1	MPZL2
KREMEN2	MS4A6A
KRT13	MSI1
KRT14	MSMB
KRT15	MT1G
KRT18	MT1H
KRT18P62	MXRA5
KRT23	MXRA5Y
KRT5	MYBPC1
KRT6A	MYCT1
KRT79	MYH14
KRT8	MYL4
LAD1	MYOZ3
LAMP3	MZB1
LAMP5	NAT8L
LCK	NDP
LCN2	NEFH
LCN6	NELL2
LCP1	NFAM1
LDHAL6B	NGEF
LEP	NGFR
LGALS7B	NKD2
LGR6	NKX3-1
LINC00086	NKX3-2
LINC00261	NOS3
LINC00547	NOSTRIN
LINC00664	NOVA2
LINC00668	NPR1
LINC00702	NPY
LINC00890	NTF4
LINC00964	NWD1
LINC01018	NYX
LINC01088	OGDHL
LINC01297	OR2I1P
LINC01315	OVOL2
LMAN1L	P2RX1
LMO2	P2RX2
LOC100421166	PAGE4
LRG1	PALD1
LRRC15	PARVG
LRRC26	PCAT18
LSP1	PCAT4
LYPD3	PCDH17
LYZ	PCGEM1
MAL2	PCP4
MALL	PDE2A
MAOA	PDE3B
MAOB	PDE9A
MAP1LC3C	PDZK1IP1
MARCH10	PECAM1
MARVELD3	PGF
MB	PGM5-AS1
MCF2L	PHF21B
MCHR1	PI15
MEOX1	PI16
MEST	PIGR
MIR200A	PKP1
MIR205HG	PKP3
MIR3189	PLA2G2A
MIR429	PLA2G4F
MIR5690	PLA2G7
MLC1	PLCH2

MMP7	PLVAP
MMP9	PMCH
MOB3B	POTEH
MPZL2	PRAC1
MRO	PROK1
MS4A6A	PRR15L
MSI1	PRSS16
MSMB	PRSS22
MT1G	PRSS35
MT1H	PRSS8
MXRA5	PTCH2
MXRA5Y	PTGER1
MYBPC1	PTPN6
MYCT1	RAB11FIP4
MYH14	RAB25
MYL4	RAI2
MYOC	RAMP3
MYOZ3	RAP1GAP
MZB1	RARRES2
NAT8L	RASAL3
NDP	RBBP8NL
NEDD9	RBFOX3
NEFH	RBM47
NELL2	RELN
NFAM1	REM1
NGEF	RGS16
NGFR	RGS7BP
NKAIN2	RIC3
NKD2	RLN1
NKX3-1	RNASE1
NKX3-2	RNF165
NOS3	RNF43
NOSTRIN	ROBO4
NOVA2	RORC
NPR1	RPLP0P2
NPSR1-AS1	RTN4RL1
NPY	RUFY4
NTF4	S100A14
NTRK2	S1PR1
NWD1	SALL3
NYX	SCGB3A1
OCA2	SDK2
OGDHL	SELE
OLAH	SELP
OR2I1P	SEMA6B
OVOL2	SERPINB11
P2RX1	SFN
P2RX2	SFRP2
P2RY14	SFRP4
PAGE4	SH2D3C
PALD1	SHH
PARVG	SHISA6
PCAT18	SLC14A1
PCAT4	SLC2A5
PCDH17	SLC44A4
PCGEM1	SLC45A3
PCP4	SLC52A3
PDE2A	SLC7A14
PDE3B	SLCO2A1
PDE9A	SMOC1
PDK4	SMR3B
PDLIM1P4	SORL1
PDZK1IP1	SOX18

PECAM1	SP5
PER1	SP8
PGF	SPDEF
PGM5-AS1	SPINK5
PHF21B	SPINT1
PI15	SPNS2
PI16	SPOCK3
PIGR	SPRR1B
PKP1	SRD5A2
PKP3	SSTR1
PLA2G2A	SSTR2
PLA2G4F	ST14
PLA2G7	STAB1
PLCE1-AS1	STAC2
PLCH2	STC1
PLVAP	SULT1C4
PMCH	SULT2B1
POTEH	SYNDIG1
PPP1R14A	SYT13
PRAC1	SYT17
PRODH	SYT7
PROK1	SYTL1
PRR15L	TAC3
PRR33	TAL1
PRSS16	TBX1
PRSS22	TBX4
PRSS35	TBX5-AS1
PRSS8	TCEAL2
PTCH2	TENM1
PTGER1	TFCP2L1
PTK2B	TFF1
PTPN6	TIE1
RAB11FIP4	TMC5
RAB25	TMC6
RAI2	TMC8
RAMP3	TMEFF2
RAP1GAP	TMEM125
RAPGEF5	TMEM150C
RARRES2	TMEM179
RASAL3	TMEM63C
RBBP8NL	TMPRSS2
RBFOX3	TNFSF15
RBM47	TNFSF18
RELN	TNNT2
REM1	TNRC6C-AS1
RGS16	TNS4
RGS7BP	TP63
RIC3	TPD52
RLN1	TPSAB1
RN7SKP69	TPSB2
RN7SL608P	TPSD1
RNA5SP111	TRGC1
RNASE1	TRIM29
RNF165	TRPM8
RNF43	TRPV6
ROBO4	TSPAN1
RORC	TSPAN7
RPL7P57	TTC22
RPLP0P2	TYROBP
RTN4RL1	UPK3A
RUFY4	VAMP8
S100A14	VENTX
S1PR1	VIPR1

SAA1	VSTM2A
SAA2	VWA1
SAA4	VWF
SALL3	WFDC2
SAMHD1	WNK2
SCGB3A1	WNT10A
SCN3A	WNT10B
SDK2	WNT11
SELE	WNT2
SELP	WNT4
SEMA6B	WNT6
SEMG1	WNT7B
SERPINB11	WSCD2
SFN	ZDHHC8P1
SFRP2	ZMYND15
SFRP4	ZNF385C
SH2D3C	
SHH	
SHISA6	
SLC14A1	
SLC16A10	
SLC16A12	
SLC2A5	
SLC38A11	
SLC44A4	
SLC45A3	
SLC52A3	
SLC7A14	
SLCO2A1	
SMOC1	
SMR3B	
SORL1	
SOX18	
SP5	
SP8	
SPDEF	
SPINK5	
SPINT1	
SPNS2	
SPOCK3	
SPRR1B	
SRD5A2	
SSTR1	
SSTR2	
ST14	
ST7-AS2	
STAB1	
STAC2	
STC1	
SULT1C4	
SULT2B1	
SYNDIG1	
SYT13	
SYT17	
SYT7	
SYTL1	
TAC3	
TAL1	
TBX1	
TBX4	
TBX5-AS1	
TCEAL2	
TENM1	

TFCP2L1
TFF1
TIE1
TIMP4
TLDC2
TMC5
TMC6
TMC8
TMEFF2
TMEM125
TMEM150C
TMEM179
TMEM63C
TMPRSS2
TNFAIP8L3
TNFSF15
TNFSF18
TNNT2
TNRC6C-AS1
TNS4
TP63
TPD52
TPSAB1
TPSB2
TPSD1
TRGC1
TRIM29
TRPM8
TRPV6
TSC22D3
TSPAN1
TSPAN7
TTC22
TUSC5
TYROBP
UBE2CP1
UPK3A
USP2
VAMP8
VENTX
VIPR1
VSTM2A
VWA1
VWF
WFDC2
WNK2
WNT10A
WNT10B
WNT11
WNT2
WNT4
WNT6
WNT7B
WSCD2
XRCC6P2
ZBTB16
ZDHHC8P1
ZMYND15
ZNF385C

Supplementary Table S5. Prioritized Target Gene Validation
Paired eye study matches with prioritized target genes

These studies used paired human donor eyes, or paired eyes from bovine donors as described in the text.
Gene functions are from GeneCards.
*Genes associated with a SNP identified in this study of genome-wide significance

Species	Gene	Log2 Fold change	Function
**Genes differentially regulated in TM cells of steroid responders**			
Bovine	*BEND7	-1.11	Transcription factor
Bovine	*COL11A1	-1.63	Collagen XI subunit involved in tendon fibrillogenesis
Bovine	COPZ2	-0.83	Adaptor for COPI-1 mediated Golgi-ER transport
Bovine	DIAPH3	1.34	Assembly of F-actin structures (Formin family)
Human	FRMD3	2.21	Unknown
Human	*FST	-2.08	Activin antagonist (TGFB superfamily member)
Bovine	HMCN1	-1.54	Multifunctional (Hemicentin)
Bovine	KCNE4	-1.83	Voltage-gated potassium channel, delayed rectifier (regulates KCNQ1)
Bovine	KLHDC7A	2.16	Unknown
Bovine	MFSD1	-0.72	Recycles lysosomal proteolysis products
Bovine	MVB12B	-0.82	Component of ESCRT-I complex that regulates vesicular trafficking
Bovine	PC	-0.77	Pyruvate carboxylase
Human	PCP4	-4.30	Calmodulin regulation
Bovine	PTHLH	-0.52	Parathyroid hormone-like hormone
Human	RBFOX3	-2.41	Regulates alternative RNA splicing
Bovine	STEAP2	0.77	Metalloreductase
Bovine	STON2	-1.55	Regulates vesicle-mediated transport
Bovine	TNK2	-0.83	Non-receptor protein kinase, downstream effector of CDC42
**Genes differentially regulated in TM cells of steroid non-responders**			
Human	*ARHGEF26	2.08	Rho-guanine nucleotide exchange factor (RhoG)
Bovine	CDH2	-0.95	Cell-cell adhesion (N-cadherin)
Human	CHAC1	-2.43	Enzymatic inhibition of Notch (g-glutamylcyclotransferase family)
Human	*FST	-2.00	TGFB superfamily inhibitor (follistatin)
Bovine	GAL	-2.26	Neuroendocrine peptide that controls smooth muscle contraction
Bovine	HS6ST1	-0.92	Enzyme that modifies heparan sulfate
Bovine	HTR2A	1.93	Serotonin receptor that reduces IOP when activated
Bovine	PTHLH	-2.50	Parathyroid hormone-like hormone
Human	PTHLH	2.42	Parathyroid hormone-like hormone
Human	*RBFOX1	-3.80	Regulates alternative RNA splicing
Bovine	TBX18	1.12	Transcriptional repressor

Supplementary Table S5. Prioritized Target Gene Validation
GC-regulation in silico

As analyzed using NIH Database for Annotation, Visualization and Integrated Discovery (DAVID) Bioinformatics functional annotation clustering tool

GWAS: 406 genes submitted **402 DAVID IDs** UCSF_TFBS

Classification stringency: Lowest
Annotation Cluster 1 (top score) Enrichment score: 9.76

Transcription Factor	Gene #	% of DAVID IDs	P value	Bonferroni-corrected	Notes
GR	222	0.58	4.7E-08	8.3E-06	Glucocorticoid receptor

Classification stringency: Medium
Single Annotation Cluster Enrichment score: 24.16

Transcription Factor	Gene #	% DAVID IDs	P value	Bonferroni-corrected	Notes
S8	252	0.63	4.00E-28	7.00E-26	Paired domain TF
LHX3	220	0.55	9.10E-28	1.60E-25	LIM domain TF
CHX10	211	0.52	1.30E-17	6.80E-17	Homeobox TF

Paired Eye: 618 genes submitted **590 DAVID IDs** **Responders** UCSF_TFBS

Classification stringency: Lowest
Annotation Cluster 1 (top score) Enrichment score: 3.42

TF	Gene #	% DAVID IDs	P value	Bonferroni-corrected	Notes
GR	264	0.45	8.01E-03	7.60E-01	Glucocorticoid receptor

Classification stringency: Medium
Single Annotation Cluster Enrichment score: 1.46

Transcription Factor	Gene #	% DAVID IDs	P value	Bonferroni-corrected	Notes
S8	244	0.41	2.00E-04	3.40E-02	
LHX3	182	0.31	4.72E-02	1.00E+00	
CHX10	196	0.33	1.80E-01	1.00E+00	
CART1	200	0.34	9.00E-01	1.00E+00	Encoded by *ALX4,* a paired-like homeodomain TF
