# Supplementary material for: Pharmacogenomics of steroid-induced ocular hypertension: relationship to high-tension glaucomas and new pathophysiologic insight
Source: medRxiv. 2025 Aug 13:2025.08.11.25333245. Preprint. [Version 1] doi: 10.1101/2025.08.11.25333245 (PMC12363710; doi:10.1101/2025.08.11.25333245)
Supplement: Supplement 7 — Table S6. Top Prioritized Target Gene Additional Validation [file media-7.pdf]

Supplementary Table S6. Top Prioritized Target Genes Additional Validation
Summary of chromatin analysis for SNPs that cluster in the top 26 risk loci

The analysis was performed on RegulomeDB

Prioritized Gene	Hit SNP	SNP Significance	Genome-level hit SNP in LD at R^2=0.4	SNP Significance	Co-localizes	AOP cell types where transcription factors listed are primarily expressed
**Features of active chromatin**						
HDAC9	rs74455595	Genome-level			caQTL	
NCAM2	rs75024143	Suggestive			SiPhy cons	
SPTY2D1	rs151115079	Genome-wide			SiPhy cons	
**Chip-identified binding proteins indicating active transcription**						
BEND7	rs184425183	Genome-wide			CCNT2	All cell types
GPLD1	rs150586237	Genome-wide			CTCF	All cell types
HDAC4	rs188076929	Genome-wide			RNA pol II	All cell types
SPTY2D1	rs151115079	Genome-wide			RNA pol II, TBP, TAF1	All cell types
**TF binding motif**						
AGAP1	rs188848340	Suggestive	rs147559909	Genome-wide	GR binding motif	All cell types
COL11A1	rs140420703	Suggestive			GR binding motif	All cell types
CCR6	rs184487573	Suggestive	rs148153037	Genome-wide	GR binding motif	All cell types
GPLD1	Multiple non-hits		rs150586237	Genome-wide	GR binding motif	All cell types
HDAC4	rs188076929				GR binding motif	All cell types
PPM1H	rs182437250	Suggestive			GR binding motif	All cell types
**Chip-identified binding proteins known to interact with the GR**						
HDAC4	rs181217257	Suggestive			JUND	All cell types
HDAC9	rs10279777	Genome-level			Pu-1, encoded by *SPI1*	Macrophages, ciliary muscle cells, neurons
SPTY2D1	rs138414342	Genome-level			STAT1, STAT3	All cell types
SPTY2D1	rs151115079	Genome-level			Oct-2, encoded by *POU2F2*	macrophages, ciliary muscle cells, neurons
SPTY2D1	rs151115079	Genome-level			HEY1	TM, vascular endothelium, Schwann cells

KEY

caQTL: "Chromatin accessibility" QTL as measured by the Assay for Transposase-Accessible Chromatin (ATAC-seq). Chromatin accessibility is a reliable indicator of local cis-regulatory activity (1).

CCNT2: A transcription factor that is a regulatory subunit of the positive transcription elongation factor B (P-TEFb) complex. P-TEFb is essential for RNA polymerase II's elongation of transcription and co-transcriptional processing.

CTCF: An eleven zinc finger (ZF), multivalent transcriptional regulator that organizes chromatin.

JUND: A subunit of AP1, which can act as a pioneer factor to prime GR binding.

HEY1: A TF target of the NOTCH signaling pathway. GCs inhibit HEY1 expression (2).

Oct-2: Encoded by POU2F2. The GR interacts physically with POU2F2 to modulate transcription (3).

Pu-1: Also known as Pu.1, a tissue-specific TF of the hematopoietic lineage (4) encoded by SPI1. GR binding elements in DNA are primed by Pu-1, which interacts with the GR (5).

RNA pol II: RNA polymerase II, an enzyme responsible for transcribing genes that encode proteins, as well as some non-coding RNA genes (6).

SiPhy Cons: A measure of evolutionary conservation based on alignment of 17 vertebrate species used by HaploregV.2. Conservation suggests function importance of these loci.

STAT1: A transcription factor. the GR does not physically bind STAT1, however an indirect mechanism of cross-modulation has been demonstrated that integrates STAT1 and the GR with PU.1 (7).

STAT3: A transcription factor. STAT3 interacts with the GR reciprocally by tethering, i.e., GR tethering to DNA-bound STAT3 results in transcriptional repression, whereas STAT3 tethering to GR results in synergism (8).

TBP: Also called TATA binding protein, a component of the transcription initiation machinery.

TAF1: A TBP-associated factor.

REFERENCES

1 J. D. Buenrostro, B. Wu, H. Y. Chang, W. J. Greenleaf, ATAC-seq: A Method for Assaying Chromatin Accessibility Genome-Wide. Curr Protoc Mol Biol 109, 21 29 21-21 29 29 (2015).

2 S. Zanotti, J. Yu, S. Adhikari, E. Canalis, Glucocorticoids inhibit notch target gene expression in osteoblasts. *Journal of cellular biochemistry* **119**, 6016-6023 (2018).

3 L. D. Ward, M. Kellis, HaploReg: a resource for exploring chromatin states, conservation, and regulatory motif alterations within sets of genetically linked variants. Nucleic acids research 40, D930-934 (2012).

4 A. Celada *et al.* , The transcription factor PU.1 is involved in macrophage proliferation. *The Journal of experimental medicine* **184**, 61-69 (1996).

5 D. Beck *et al.* , PU.1 eviction at lymphocyte-specific chromatin domains mediates glucocorticoid response in acute lymphoblastic leukemia. Nature communications 15, 9697 (2024).

6 K. M. Harlen, L. S. Churchman, The code and beyond: transcription regulation by the RNA polymerase II carboxy-terminal domain. Nature reviews. Molecular cell biology 18, 263-273 (2017).

7 S. Aittomaki *et al.* , Cooperation among Stat1, glucocorticoid receptor, and PU.1 in transcriptional activation of the high-affinity Fc gamma receptor I in monocytes. Journal of immunology 164, 5689-5697 (2000).

8 D. Langlais, C. Couture, A. Balsalobre, J. Drouin, The Stat3/GR interaction code: predictive value of direct/indirect DNA recruitment for transcription outcome. Molecular cell 47, 38-49 (2012).
