# Supplementary material for: Pharmacogenomics of steroid-induced ocular hypertension: relationship to high-tension glaucomas and new pathophysiologic insight
Source: medRxiv. 2025 Aug 13:2025.08.11.25333245. Preprint. [Version 1] doi: 10.1101/2025.08.11.25333245 (PMC12363710; doi:10.1101/2025.08.11.25333245)
Supplement: Supplement 8 — Table S7. GWAS Results Indianapolis-2 Replication Cohort [file media-8.pdf]

Supplementary Table S7. Independent Replication

Indianpolis-2 cohort, 12M quantitative trait (QT) data, comparing to discovery 12M QT data

Threshold for replicative significance:	5.00E-02
Total SNPs found:	92
Total risk loci found:	61

Gray shading = SNPs and target genes of genome-wide significance in the Indianapolis-1 cohort prior to adjustment for multiple comparisons
NA: not applicable

ID	chr	POS_37	freq	MAC	Score	Score.SE	Score.Stat	Score.pval	Func.refGene	Gene.refGene	GeneDetail.refGene	rsID	gnomAD_genome_ALL	gnomAD_genome_NFE	Rsq	hwe
1:23465122:A:G	1	23465122	0.004136	1	0.272297	0.126064	2.159983	3.08E-02	intronic	LUZP1	.	rs35145334	0.0093	0.016	0.847376	1
9:99922419:T:C	9	99922419	0.020922	4	0.611973	0.283548	2.158269	3.09E-02	ncRNA_intronic	ANKRD18CP	.	rs144138711	0.0219	0.0313	0.851063	1
3:179593768:G:A	3	1.8E+08	0.005053	1	-0.237	0.148742	-1.59336	1.11E-01	intronic	PEX5L	.	rs76356799	0.0159	0.0043	0.943713	1
8:62672723:G:A	8	62672723	0.270408	56	1.388749	0.884022	1.570944	1.16E-01	intergenic	MIR4470;NKAIN3	dist=45305;dist=488778	rs12114488	0.2842	0.3161	0.983174	0.315736
16:6412967:C:T	16	6412967	0.009704	2	-0.32659	0.210726	-1.54983	1.21E-01	intronic	RBFOX1	.	rs80212581	0.0053	0.0049	0.980445	1
16:6433735:C:T	16	6433735	0.009495	2	-0.32103	0.208076	-1.54287	1.23E-01	intronic	RBFOX1	.	rs140276610	0.0049	0.0053	0.976824	1
5:52662672:G:A	5	52662672	0.010612	2	0.318972	0.208004	1.53349	1.25E-01	intergenic	LOC257396;FST	dist=251716;dist=113592	rs142934021	0.0054	0.0073	0.873041	1
20:61590782:T:C	20	61590782	0.436893	90	-1.66492	1.092258	-1.52429	1.27E-01	intronic	SLC17A9	.	rs2427460	0.4464	0.502	1	0.843783
3:136134595:C:T	3	1.36E+08	0.003675	1	-0.1502	0.108093	-1.38952	1.65E-01	intronic	STAG1	.	rs148248743	0.0018	0.0025	0.67579	1
2:67825685:C:T	2	67825685	0.118481	24	0.992877	0.716431	1.385865	1.66E-01	intergenic	ETAA1;LOC101927701	dist=188152;dist=197501	rs2902021	0.1449	0.0918	0.896322	0.6091
21:41307923:G:A	21	41307923	0.891942	22	0.884584	0.662312	1.3356	1.82E-01	intergenic	PCP4;DSCAM	dist=6601;dist=76420	rs7275595	0.9016	0.9021	0.944606	1
21:41307573:G:A	21	41307573	0.894325	22	0.881903	0.660646	1.33491	1.82E-01	intergenic	PCP4;DSCAM	dist=6251;dist=76770	rs9974985	0.8689	0.9016	0.955895	1
7:109155716:T:C	7	1.09E+08	0.012738	3	0.281003	0.211468	1.328821	1.84E-01	intergenic	C7orf66;EIF3IP1	dist=631072;dist=443568	rs73202425	0.0101	0.0152	0.773926	1
21:41304765:A:G	21	41304765	0.894335	22	0.874736	0.658912	1.327545	1.84E-01	intergenic	PCP4;DSCAM	dist=3443;dist=79578	rs9636964	0.8763	0.9031	0.954712	1
21:41305720:G:A	21	41305720	0.894335	22	0.874386	0.658989	1.326859	1.85E-01	intergenic	PCP4;DSCAM	dist=4398;dist=78623	rs9305683	0.9114	0.9025	0.954916	1
16:6858239:C:T	16	6858239	0.001539	0	0.06332	0.049039	1.291222	1.97E-01	intronic	RBFOX1	.	rs138164904	0.0006	0.0009	0.313951	1
10:110423709:C:T	10	1.1E+08	0.023029	5	-0.46784	0.374512	-1.2492	2.12E-01	intergenic	LINC01435;XPNPEP1	dist=594658;dist=1200815	rs147944608	0.0105	0.0143	0.927213	1
11:439012:C:T	11	439012	0.00417	1	0.159245	0.128995	1.234507	2.17E-01	intronic	ANO9	.	rs556274646	0.0033	0.006	0.85841	1
7:16291999:A:C	7	16291999	0.00518	1	0.183439	0.150082	1.22226	2.22E-01	ncRNA_intronic	ISPD-AS1	.	rs143686474	0.0064	0.0088	0.935964	1
13:74357508:A:C	13	74357508	0.009981	2	-0.23228	0.205459	-1.13056	2.58E-01	intronic	KLF12	.	rs368187808	0.0015	0.0025	0.90129	1
13:74357510:A:G	13	74357510	0.009976	2	-0.23215	0.205448	-1.12998	2.58E-01	intronic	KLF12	.	rs372194899	0.0015	0.0025	0.90161	1
21:41309565:G:T	21	41309565	0.873786	26	0.828474	0.746251	1.110182	2.67E-01	intergenic	PCP4;DSCAM	dist=8243;dist=74778	rs9981433	0.8718	0.8747	1	0.657884
3:158586849:T:C	3	1.59E+08	0.005316	1	0.117913	0.111428	1.058203	2.90E-01	intergenic	MFSD1;IQCJ	dist=39341;dist=200192	rs139055031	0.0034	0.0053	0.484725	1
1:65965720:A:G	1	65965720	0.073262	15	0.565301	0.545915	1.035511	3.00E-01	intronic	LEPR	.	rs7534177	0.0979	0.0654	0.986327	1
4:169934087:G:A	4	1.7E+08	0.087379	18	0.629008	0.621674	1.011797	3.12E-01	intergenic	CBR4;SH3RF1	dist=2619;dist=81320	rs17615362	0.0556	0.0641	1	0.556401
4:169934725:T:C	4	1.7E+08	0.087379	18	0.626322	0.620036	1.010139	3.12E-01	intergenic	CBR4;SH3RF1	dist=3257;dist=80682	rs17543620	0.0621	0.0702	0.995146	1
1:65943471:C:T	1	65943471	0.072874	15	0.552048	0.546971	1.009281	3.13E-01	intronic	LEPR	.	rs17127656	0.1088	0.0656	0.992874	1
1:65957141:C:A	1	65957141	0.072816	15	0.550472	0.548981	1.002716	3.16E-01	intronic	LEPR	.	rs11579567	0.0982	0.0657	1	1
1:65948791:T:C	1	65948791	0.072762	15	0.547758	0.546973	1.001434	3.17E-01	intronic	LEPR	.	rs7518849	0.098	0.0656	0.993624	1
21:41308948:A:G	21	41308948	0.883495	24	0.709232	0.720103	0.984903	3.25E-01	intergenic	PCP4;DSCAM	dist=7626;dist=75395	rs1005412	0.872	0.8867	1	0.621408
17:77210823:A:G	17	77210823	0.935019	13	-0.44428	0.461435	-0.96281	3.36E-01	intronic	RBFOX3	.	rs2606194	0.8833	0.9459	0.8207	1
22:34473543:C:T	22	34473543	0.024262	5	0.317336	0.332644	0.953978	3.40E-01	intergenic	LARGE;ISX	dist=157127;dist=988586	rs77297738	0.0124	0.0161	0.99959	1
17:46078602:C:T	17	46078602	0.009699	2	-0.20219	0.216503	-0.93387	3.50E-01	intergenic	CDK5RAP3;COPZ2	dist=19450;dist=24931	rs188353596	0.0024	0.0041	0.99899	1
20:30948839:C:T	20	30948839	0.005403	1	0.133812	0.148013	0.904051	3.66E-01	intronic	ASXL1	.	rs148157126	0.0048	0.0067	0.890955	1
20:30946654:G:A	20	30946654	0.005393	1	0.133515	0.147933	0.902538	3.67E-01	intronic	ASXL1	.	rs200198574	0.0051	0.0072	0.891725	1
20:30921343:C:T	20	30921343	0.005325	1	0.132942	0.147636	0.900467	3.68E-01	UTR3	KIF3B	NM_004798:c.*2221C>T	rs139816293	0.0052	0.0072	0.899871	1
20:30892775:C:T	20	30892775	0.005316	1	0.13291	0.147611	0.900412	3.68E-01	intronic	KIF3B	.	rs143432612	0.005	0.0071	0.901	1
20:30862126:C:T	20	30862126	0.005733	1	0.129444	0.147579	0.877116	3.80E-01	intergenic	POFUT1;KIF3B	dist=35659;dist=3328	rs145421321	0.0052	0.0075	0.840335	1
20:30771880:T:C	20	30771880	0.006121	1	0.123868	0.151109	0.819724	4.12E-01	intergenic	TM9SF4;TSPY26P	dist=16819;dist=5069	rs193041547	0.0034	0.0046	0.830953	1
20:30780644:G:A	20	30780644	0.00665	1	0.124148	0.152056	0.816465	4.14E-01	UTR3	PLAGL2	NM_002657:c.*3611C>T	rs138055631	0.0052	0.0075	0.775332	1
20:30722429:G:A	20	30722429	0.006835	1	0.123006	0.153898	0.79927	4.24E-01	intronic	TM9SF4	.	rs145791959	0.0052	0.0074	0.773327	1
1:202122499:C:T	1	2.02E+08	0.009515	2	0.169365	0.213461	0.793423	4.28E-01	intronic	PTPN7	.	rs145804766	0.0059	0.008	0.98004	1
2:67831153:T:G	2	67831153	0.068092	14	0.452063	0.578871	0.780939	4.35E-01	intergenic	ETAA1;LOC101927701	dist=193620;dist=192033	rs75082290	0.0671	0.0909	0.993697	0.380032
20:30682231:C:T	20	30682231	0.006354	1	0.117001	0.151263	0.773494	4.39E-01	intronic	HCK	.	rs146249289	0.0032	0.0045	0.805469	1
20:6106741:A:G	20	6106741	0.006544	1	0.123424	0.159657	0.773058	4.39E-01	intergenic	FERMT1;CASC20	dist=2550;dist=300638	rs547186621	0.0023	0.0028	0.816709	1
20:30678170:C:T	20	30678170	0.007146	1	0.114355	0.153477	0.745095	4.56E-01	intronic	HCK	.	rs149859280	0.0049	0.0072	0.738256	1

20:30675687:C:T	20	30675687	0.007228	1	0.113145	0.153721	0.736045	4.62E-01	intronic	HCK	.	rs138733283	0.0052	0.0074	0.732554	1
3:19586745:T:C	3	19586745	0.006107	1	-0.09553	0.135182	-0.70668	4.80E-01	intergenic	KCNH8;EFHB	dist=9610;dist=334221	rs144788248	0.0068	0.0095	0.646941	1
13:61918888:G:A	13	61918888	0.003325	1	0.070028	0.1011	0.69266	4.89E-01	intergenic	MIR3169;PCDH20	dist=144874;dist=64931	rs113791989	0.0034	0.0052	0.633334	1
14:105590577:T:C	14	1.06E+08	0.004782	1	-0.06932	0.101416	-0.68353	4.94E-01	intergenic	LOC102723354;JAG2	dist=24744;dist=16741	rs190251199	0.0007	0.0012	0.479557	1
18:66029612:G:A	18	66029612	0.003282	1	-0.06389	0.095487	-0.66911	5.03E-01	intergenic	LOC643542;TMX3	dist=462756;dist=311313	rs559152067	0.0016	0.0026	0.635695	1
2:68110046:C:T	2	68110046	0.005029	1	0.101998	0.153105	0.6662	5.05E-01	intergenic	LOC101927701;C1D	dist=57352;dist=159286	rs191298981	0.0082	0.0125	0.96377	1
7:117175907:T:C	7	1.17E+08	0.004854	1	-0.10071	0.153134	-0.65764	5.11E-01	intronic	CFTR	.	rs529110230	0.0019	0.0031	1	1
7:117199874:T:C	7	1.17E+08	0.004854	1	-0.10071	0.153134	-0.65764	5.11E-01	intronic	CFTR	.	rs142215699	0.0019	0.0031	1	1
7:117218328:A:C	7	1.17E+08	0.004854	1	-0.10071	0.153134	-0.65764	5.11E-01	intronic	CFTR	.	rs142721557	0.0019	0.0031	1	1
7:117225781:G:A	7	1.17E+08	0.004854	1	-0.10071	0.153134	-0.65764	5.11E-01	intronic	CFTR	.	rs201355675	0.0019	0.0031	1	1
7:117109021:A:G	7	1.17E+08	0.004864	1	-0.10065	0.153135	-0.65728	5.11E-01	intergenic	ASZ1;CFTR	dist=41444;dist=10996	rs576047962	0.0017	0.0028	0.997998	1
13:74054452:T:C	13	74054452	0.00485	1	-0.09932	0.153747	-0.64602	5.18E-01	intergenic	KLF5;LINC00392	dist=402772;dist=83929	rs532269430	0.0015	0.0027	0.998995	1
7:117043294:A:G	7	1.17E+08	0.004893	1	-0.09876	0.152972	-0.64563	5.19E-01	intronic	ASZ1	.	rs558784715	0.0016	0.0026	0.990072	1
2:68289497:T:C	2	68289497	0.004859	1	0.097766	0.1532	0.638158	5.23E-01	intronic	C1D	.	rs113154814	0.0106	0.0165	0.996991	1
2:68234930:A:C	2	68234930	0.004864	1	0.097713	0.153361	0.637145	5.24E-01	intergenic	LOC101927701;C1D	dist=182236;dist=34402	rs113537164	0.0075	0.0113	0.997998	1
22:34492649:A:G	22	34492649	0.019971	4	0.173113	0.277568	0.623678	5.33E-01	intergenic	LARGE;ISX	dist=176233;dist=969480	rs74572772	0.013	0.0159	0.632536	1
12:6681868:C:T	12	6681868	0.03383	7	-0.21438	0.367327	-0.58361	5.59E-01	intronic	CHD4	.	rs61918041	0.0236	0.0328	0.98866	1
12:6684385:G:A	12	6684385	0.033092	7	-0.20101	0.360436	-0.5577	5.77E-01	intronic	CHD4	.	rs113244573	0.0235	0.0322	0.969714	1
1:84866196:G:A	1	84866196	0.013515	3	-0.12062	0.230011	-0.52442	6.00E-01	intronic	DNASE2B	.	rs139390630	0.0097	0.0138	0.823131	1
4:11623450:G:A	4	11623450	0.009558	2	0.110272	0.211241	0.522021	6.02E-01	intergenic	HS3ST1;LOC101929019	dist=192913;dist=601625	rs189094663	0.0041	0.0072	0.979654	1
4:19013698:T:C	4	19013698	0.003825	1	-0.0443	0.086384	-0.51281	6.08E-01	intergenic	LCORL;SLIT2	dist=990215;dist=1239830	rs557276277	0.0083	0.0145	0.408399	1
3:177574308:G:A	3	1.78E+08	0.004859	1	0.074678	0.153016	0.488038	6.26E-01	ncRNA_intronic	KCCAT211	.	rs189709453	0.002	0.0034	0.998997	1
3:177639777:T:C	3	1.78E+08	0.004854	1	0.074358	0.153014	0.485954	6.27E-01	intergenic	KCCAT211;LINC01014	dist=22765;dist=497212	rs186767531	0.0027	0.0044	1	1
3:177712448:C:T	3	1.78E+08	0.004854	1	0.074358	0.153014	0.485954	6.27E-01	intergenic	KCCAT211;LINC01014	dist=95436;dist=424541	rs182868205	0.0026	0.0045	1	1
15:40280073:G:A	15	40280073	0.009694	2	0.101464	0.208912	0.485679	6.27E-01	intronic	EIF2AK4	.	rs56324718	0.0071	0.0074	0.998486	1
6:20458179:C:T	6	20458179	0.014573	3	0.117291	0.247031	0.474803	6.35E-01	intronic	E2F3	.	rs73384619	0.0596	0.0208	0.999325	1
15:33113387:C:T	15	33113387	0.004772	1	-0.0691	0.149659	-0.4617	6.44E-01	intronic	FMN1	.	rs187281112	0.0022	0.0039	0.982918	1
2:13059945:T:C	2	13059945	0.003388	1	-0.04453	0.098359	-0.45274	6.51E-01	intergenic	TRIB2;LOC100506474	dist=177087;dist=46963	rs76220567	0.0046	0.0056	0.634926	1
7:117274731:T:C	7	1.17E+08	0.009432	2	-0.08504	0.20862	-0.40762	6.84E-01	intronic	CFTR	.	rs188993522	0.0017	0.003	0.971904	1
22:34493647:G:A	22	34493647	0.018714	4	0.107941	0.264895	0.407485	6.84E-01	intergenic	LARGE;ISX	dist=177231;dist=968482	rs80019988	0.013	0.016	0.588641	1
3:190584679:A:G	3	1.91E+08	0.006	1	-0.06018	0.150285	-0.40046	6.89E-01	intergenic	GMNC;SNAR-I	dist=4214;dist=11040	rs117591241	0.0085	0.0102	0.813408	1
4:23509067:T:C	4	23509067	0.00517	1	0.051794	0.145762	0.355335	7.22E-01	intergenic	MIR548AJ2;PPARGC1A	dist=44341;dist=284577	rs113063005	0.0035	0.0047	0.828627	1
1:85191044:G:A	1	85191044	0.009714	2	-0.0613	0.204456	-0.29982	7.64E-01	intergenic	SSX2IP;LPAR3	dist=34804;dist=88042	rs114067899	0.006	0.0099	0.999496	1
2:43096699:A:G	2	43096699	0.00484	1	-0.04328	0.153475	-0.28203	7.78E-01	intergenic	HAAO;LOC102723854	dist=76948;dist=158293	rs186792608	0.0013	0.0022	0.996985	1
20:1928157:A:G	20	1928157	0.020388	4	0.072971	0.368343	0.198106	8.43E-01	intergenic	SIRPA;PDYN	dist=7617;dist=31245	rs62192733	0.0101	0.0133	0.933047	1
3:132842203:G:A	3	1.33E+08	0.004782	1	-0.02836	0.150192	-0.18881	8.50E-01	intronic	TMEM108	.	rs114280794	0.0059	0.0089	0.984928	1
17:16839162:C:T	17	16839162	0.004714	1	0.025523	0.144513	0.176614	8.60E-01	intergenic	KRT16P2;TNFRSF13B	dist=103015;dist=3236	rs116897913	0.0122	0.0155	0.918062	1
1:112349372:C:T	1	1.12E+08	0.014718	3	-0.04239	0.252199	-0.16808	8.67E-01	intronic	KCND3	.	rs76098744	0.0239	0.0167	0.988834	1
1:112354418:G:A	1	1.12E+08	0.01468	3	-0.04122	0.252178	-0.16346	8.70E-01	intronic	KCND3	.	rs74683551	0.0237	0.0165	0.991424	1
8:57055978:G:A	8	57055978	0.015505	3	-0.03569	0.25758	-0.13855	8.90E-01	intergenic	MOS;PLAG1	dist=29437;dist=17490	rs62515405	0.0194	0.0231	0.875866	1
10:23490165:C:T	10	23490165	0.009024	2	0.022892	0.177334	0.129089	8.97E-01	intergenic	PTF1A;LINC01552	dist=6984;dist=2580	rs142956968	0.0083	0.0142	0.711695	1
18:26603650:G:T	18	26603650	0.014316	3	-0.02055	0.226207	-0.09086	9.28E-01	intergenic	CDH2;MIR302F	dist=846240;dist=1275226	rs147669485	0.0046	0.0075	0.684042	1
8:57141203:G:T	8	57141203	0.006277	1	-0.01312	0.146099	-0.0898	9.28E-01	intergenic	CHCHD7;SDR16C5	dist=10027;dist=71367	rs62515436	0.0114	0.0136	0.695945	1
10:23601847:G:A	10	23601847	0.009451	2	0.015327	0.177616	0.086294	9.31E-01	intergenic	LINC01552;C10orf67	dist=73126;dist=3673	rs145764464	0.0088	0.0145	0.685335	1
4:129647369:C:T	4	1.3E+08	0.008466	2	0.013773	0.182927	0.075292	9.40E-01	intergenic	LOC100507487;JADE1	dist=206818;dist=83409	rs181132315	0.0051	0.0056	0.803697	1
3:159392885:G:A	3	1.59E+08	0.00367	1	0.006371	0.093544	0.068105	9.46E-01	intronic	IQCJ-SCHIP1;SCHIP1	.	rs143669489	0.0016	0.0029	0.705905	1

Supplementary Table S7. Independent Replication

Indianpolis-2 cohort, 3M quantitative trait (QT) data, comparing to discovery 3M QT data

Threshold for replicative significance:	5.00E-02
Total SNPs found:	290
Total risk loci found:	164

Gray shading = SNPs and target genes of genome-wide significance in the Indianapolis-1 cohort prior to adjustment for multiple comparisons
NA: not applicable

ID	chr	POS_37	freq	MAC	Score	Score.SE	Score.Stat	Score.pval	Func.refGene	Gene.refGene	GeneDetail.refGene	rsID	gnomAD_genome_ALL	gnomAD_genome_NFE	Rsq	hwe
15:59634792:	15	59634792	0.00785437	2	0.72549628	0.23800588	3.04822843	2.30E-03	intronic	MYO1E	.	rs182303755	0.0048	0.008	0.815149	1
1:9655903:G:	1	9655903	0.0048835	1	0.44239391	0.2092153	2.11453898	3.45E-02	intronic	TMEM201	.	rs115348382	0.003	0.0038	0.988049	1
8:123001411:	8	123001411	0.01012621	2	-0.5208585	0.25910027	-2.01025844	4.44E-02	intergenic	HAS2;SMILR	dist=343847;dist=425155	rs536803366	0.0019	0.0033	0.829081	1
2:188375400:	2	188375400	0.00799029	2	0.44447255	0.22253499	1.99731537	4.58E-02	intronic	TFPI	.	rs112557251	0.0071	0.0095	0.735086	1
4:91825885:T	4	91825885	0.03020388	6	-0.93114505	0.48910792	-1.90376194	5.69E-02	intronic	CCSER1	.	rs17017794	0.0466	0.0424	0.96782	1
4:163238743:	4	163238743	0.01746117	4	-0.60422933	0.32428843	-1.86324663	6.24E-02	intergenic	FSTL5;MIR4454	dist=153557;dist=775983	rs116651654	0.0122	0.0151	0.707424	1
8:18030674:C	8	18030674	0.00499515	1	-0.33601812	0.19641112	-1.71078969	8.71E-02	intronic	NAT1	.	rs185874707	0.005	0.0077	0.968653	1
13:58213864:	13	58213864	0.00693689	1	0.3593686	0.21068655	1.70570263	8.81E-02	intronic	PCDH17	.	rs534845494	0.0028	0.0046	0.795123	1
8:13251991:C	8	13251991	0.02897087	6	0.81880035	0.48330222	1.69417875	9.02E-02	intronic	DLC1	.	rs139062456	0.0108	0.012	0.994566	1
1:180930424:	1	180930424	0.00915534	2	-0.45705544	0.27028043	-1.69104159	9.08E-02	intergenic	KIAA1614;STX6	dist=15185;dist=11426	rs76617932	0.007	0.0085	0.944101	1
13:57979549:	13	57979549	0.0086068	2	0.39361307	0.24115519	1.63219821	1.03E-01	intergenic	PRR20E;PCDH17	dist=235197;dist=226240	rs150077525	0.0043	0.0069	0.811421	1
6:16534923:C	6	16534923	0.02279612	5	0.66818429	0.42178265	1.58419103	1.13E-01	intronic	ATXN1	.	rs1877768	0.0351	0.0251	0.946874	1
10:20372316:	10	20372316	0.00485922	1	-0.31534037	0.2019935	-1.56114122	1.18E-01	intronic	PLXDC2	.	rs529011661	0.0024	0.004	0.998997	1
7:18414613:G	7	18414613	0.00750971	2	0.35381562	0.22837308	1.54928774	1.21E-01	intronic	HDAC9	.	rs75606013	0.0176	0.0088	0.837192	1
7:18410845:G	7	18410845	0.0075	2	0.35371015	0.22837492	1.54881349	1.21E-01	intronic	HDAC9	.	rs75773869	0.0169	0.0089	0.838286	1
7:18408761:A	7	18408761	0.00750485	2	0.35353624	0.22837328	1.54806305	1.22E-01	intronic	HDAC9	.	rs77300464	0.0194	0.0089	0.837738	1
7:18410250:G	7	18410250	0.00750485	2	0.35353624	0.22837328	1.54806305	1.22E-01	intronic	HDAC9	.	rs79602997	0.0172	0.0091	0.837738	1
7:18406573:C	7	18406573	0.00750971	2	0.35365957	0.22847274	1.54792894	1.22E-01	intronic	HDAC9	.	rs75689761	0.0199	0.009	0.837902	1
8:55692112:G	8	55692112	0.46905825	97	-2.28157298	1.48522301	-1.53618209	1.24E-01	intergenic	RP1;XKR4	dist=148718;dist=322905	rs7843693	0.4432	0.388	0.990415	0.690082
8:55695310:A	8	55695310	0.46908253	97	-2.28105744	1.48521945	-1.53583866	1.25E-01	intergenic	RP1;XKR4	dist=151916;dist=319707	rs1396896	0.4436	0.3881	0.990319	0.690082
16:59302775:	16	59302775	0.00819903	2	0.3450539	0.22555602	1.52979244	1.26E-01	intergenic	GOT2;APOOP5	dist=534514;dist=485270	rs183817723	0.0044	0.0032	0.723856	1
10:24852783:	10	24852783	0.02349515	5	-0.67863561	0.44451742	-1.52667945	1.27E-01	intergenic	KIAA1217;ARHGAP21	dist=16006;dist=19755	rs12266995	0.1372	0.0298	0.965512	1
12:64920957:	12	64920957	0.06796117	14	-1.28781283	0.84420877	-1.52546725	1.27E-01	intergenic	TBK1;RASSF3	dist=25058;dist=83336	rs12315614	0.0961	0.0987	1	0.0619479
8:55699781:C	8	55699781	0.46989321	97	-2.25231369	1.48620762	-1.51547715	1.30E-01	intergenic	RP1;XKR4	dist=156387;dist=315236	rs1391462	0.4423	0.3872	0.985264	0.55697
10:12670548	10	126705489	0.00558252	1	-0.30609897	0.20590453	-1.48660628	1.37E-01	intronic	CTBP2	.	rs193093906	0.0079	0.0128	0.884528	1
10:13471195:	10	13471195	0.00971359	2	0.42523176	0.2870612	1.48132789	1.39E-01	intergenic	SEPHS1;BEND7	dist=80897;dist=9289	rs184458518	0.0051	0.0091	0.953876	1
10:13497976:	10	13497976	0.00968447	2	0.4241555	0.28706533	1.47755736	1.40E-01	intronic	BEND7	.	rs117998251	0.0045	0.0079	0.956758	1
21:38863820:	21	38863820	0.00485437	1	-0.29925684	0.20380794	-1.46832769	1.42E-01	intronic	DYRK1A	.	rs118084887	0.0034	0.0057	0.993983	1
8:55705021:A	8	55705021	0.46419418	96	-2.19012891	1.49158643	-1.46832183	1.42E-01	intergenic	RP1;XKR4	dist=161627;dist=309996	rs12678939	0.3477	0.3842	0.987656	0.549725
21:38766484:	21	38766484	0.00491748	1	-0.30034437	0.20455787	-1.46826114	1.42E-01	intronic	DYRK1A	.	rs117185941	0.0044	0.0069	0.987225	1
21:38763032:	21	38763032	0.00491748	1	-0.30034437	0.20455787	-1.46826114	1.42E-01	intronic	DYRK1A	.	rs183586634	0.0035	0.0057	0.987225	1
8:55716905:C	8	55716905	0.46564078	96	-2.17879595	1.48634229	-1.46587765	1.43E-01	intergenic	RP1;XKR4	dist=173511;dist=298112	rs1498183	0.3488	0.3861	0.983748	0.690082
13:59286033:	13	59286033	0.00468932	1	0.27744904	0.19035668	1.45752193	1.45E-01	intergenic	LINC00374;DIAPH3	dist=478782;dist=953688	rs140062526	0.0029	0.005	0.934134	1
8:55698295:C	8	55698295	0.46430583	96	-2.17232303	1.49332116	-1.45469246	1.46E-01	intergenic	RP1;XKR4	dist=154901;dist=316722	rs2375219	0.3341	0.3847	0.990078	0.549725
10:13457520:	10	13457520	0.00958252	2	0.41311337	0.28412944	1.45396183	1.46E-01	intergenic	SEPHS1;BEND7	dist=67222;dist=22964	rs184425183	0.0037	0.0066	0.946776	1
8:55529073:C	8	55529073	0.66018932	70	1.95385328	1.3487623	1.44862685	1.47E-01	intronic	RP1	.	rs9643828	0.698	0.6852	0.999978	0.826165
7:18406599:A	7	18406599	0.0096068	2	0.40693621	0.28244996	1.44073739	1.50E-01	intronic	HDAC9	.	rs77346868	0.0255	0.009	0.987516	1
7:18407464:A	7	18407464	0.00961165	2	0.40687836	0.2824402	1.44058233	1.50E-01	intronic	HDAC9	.	rs78225611	0.0266	0.0093	0.987013	1
7:18441589:G	7	18441589	0.00975728	2	0.41068288	0.2856315	1.4378067	1.50E-01	intronic	HDAC9	.	rs10279777	0.0875	0.0105	0.993989	1
7:18425017:T	7	18425017	0.00968447	2	0.40865008	0.28449932	1.43638331	1.51E-01	intronic	HDAC9	.	rs78907958	0.0305	0.0097	0.993463	1
7:18418351:A	7	18418351	0.00967961	2	0.40862411	0.28450667	1.43625493	1.51E-01	intronic	HDAC9	.	rs61434999	0.0296	0.0096	0.993966	1
7:18447436:A	7	18447436	0.00998544	2	0.41019612	0.28563996	1.43606001	1.51E-01	intronic	HDAC9	.	rs75090694	0.0289	0.0089	0.971757	1
7:18431110:G	7	18431110	0.00970874	2	0.40971334	0.28568154	1.43416107	1.52E-01	intronic	HDAC9	.	rs76526501	0.0299	0.009	0.998991	1
7:18431784:A	7	18431784	0.00970874	2	0.40971334	0.28568154	1.43416107	1.52E-01	intronic	HDAC9	.	rs74455595	0.0207	0.0091	0.998991	1
7:18433827:T	7	18433827	0.00970874	2	0.40971334	0.28568154	1.43416107	1.52E-01	intronic	HDAC9	.	rs79182806	0.0192	0.0093	0.998991	1
7:18446741:G	7	18446741	0.00971359	2	0.40983667	0.28582926	1.43385134	1.52E-01	intronic	HDAC9	.	rs17169602	0.0606	0.0097	0.999496	1
7:18443215:A	7	18443215	0.00970874	2	0.40982622	0.28583174	1.43380237	1.52E-01	intronic	HDAC9	.	rs80156375	0.0293	0.0095	1	1
7:18446807:G	7	18446807	0.00970874	2	0.40939242	0.28553735	1.43376139	1.52E-01	intronic	HDAC9	.	rs10486295	0.0603	0.0093	0.997983	1
7:18442275:G	7	18442275	0.00971845	2	0.40947561	0.28568053	1.43333399	1.52E-01	intronic	HDAC9	.	rs77867199	0.1233	0.0109	0.997984	1
11:12526635:	11	125266353	0.00484952	1	0.2862077	0.20021915	1.42947215	1.53E-01	intronic	PKNOX2	.	rs79539453	0.0389	0.0025	0.998995	1

9:93415465:C	9	93415465	0.00476214	1	0.2780232	0.19538179	1.42297394	1.55E-01	intergenic	DIRAS2;SYK	dist=10078;dist=148497	rs187213609	0.0016	0.0021	0.968915	1
8:1632241:A:	8	1632241	0.03656311	8	0.7855068	0.55347195	1.41923506	1.56E-01	intronic	DLGAP2	.	rs187384541	0.0001	7.43E-05	0.733414	1
7:36574504:G	7	36574504	0.0497233	10	-0.80335714	0.56735516	-1.41596869	1.57E-01	intronic	AOAH	.	rs62447184	0.0457	0.0637	0.770789	1
3:155451289:	3	155451289	0.0125	3	-0.42971876	0.30457365	-1.4108862	1.58E-01	intergenic	PLCH1;C3orf33	dist=29292;dist=29112	rs139943877	0.0066	0.0089	0.872873	1
16:77715551:	16	77715551	0.00730583	2	0.30227684	0.22038314	1.37159694	1.70E-01	intergenic	ADAMTS18;NUDT7	dist=246540;dist=40838	rs529523094	0.002	0.003	0.754817	1
8:55556069:G	8	55556069	0.63747088	75	1.84802295	1.36717244	1.35171168	1.76E-01	intergenic	RP1;XKR4	dist=12675;dist=458948	rs423841	0.5779	0.6682	0.980476	0.669247
3:122722331:	3	122722331	0.01008738	2	0.36512822	0.2705252	1.34970132	1.77E-01	intronic	SEMA5B	.	rs80203220	0.0061	0.0094	0.95657	1
14:22641516:	14	22641516	0.00970874	2	0.3901669	0.29078665	1.34176346	1.80E-01	intergenic	OR4E1;DAD1	dist=502284;dist=392291	rs138215817	0.0013	0.002	0.981013	1
8:55676101:A	8	55676101	0.37233981	77	-1.84721403	1.39730572	-1.32198273	1.86E-01	intergenic	RP1;XKR4	dist=132707;dist=338916	rs1812506	0.4385	0.3406	0.99112	1
8:55630615:A	8	55630615	0.37378641	77	-1.85238189	1.40315023	-1.32015935	1.87E-01	intergenic	RP1;XKR4	dist=87221;dist=384402	rs1595406	0.4595	0.3453	1	1
8:55628637:G	8	55628637	0.37276214	77	-1.84311115	1.39647	-1.31983584	1.87E-01	intergenic	RP1;XKR4	dist=85243;dist=386380	rs720372	0.4592	0.3452	0.99107	1
8:55564609:A	8	55564609	0.64240777	74	1.79482003	1.37005262	1.3100373	1.90E-01	intergenic	RP1;XKR4	dist=21215;dist=450408	rs433324	0.5758	0.6715	0.98104	0.829223
8:55640722:T	8	55640722	0.36893689	76	-1.79459071	1.41295153	-1.27010068	2.04E-01	intergenic	RP1;XKR4	dist=97328;dist=374295	rs2375536	0.4555	0.3417	0.999979	1
9:93330047:T	9	93330047	0.00485437	1	0.21871848	0.17295374	1.26460683	2.06E-01	ncRNA_intronic	LINC01501	.	rs183737367	0.0016	0.002	0.751636	1
22:19969182:	22	19969182	0.00311651	1	-0.1179241	0.09397486	-1.25484732	2.10E-01	exonic	ARVCF	.	rs113625788	0.0017	0.0025	0.324346	1
2:48655397:C	2	48655397	0.01459709	3	0.44891532	0.35836978	1.25265953	2.10E-01	intergenic	FOXN2;PPP1R21	dist=48963;dist=12511	rs189890455	0.0021	0.0032	0.996316	1
3:153657202:	3	153657202	0.03714078	8	0.78752596	0.62917763	1.25167508	2.11E-01	intergenic	C3orf79;ARHGEF26-AS1	dist=436716;dist=84988	rs16823323	0.0473	0.0194	0.961634	0.100888
9:93030699:G	9	93030699	0.0043932	1	-0.17792576	0.14232613	-1.25012711	2.11E-01	intergenic	MIR4290HG;LINC01508	dist=226918;dist=32479	rs545690161	0.0048	0.0067	0.588805	1
15:58879765:	15	58879765	0.0559466	12	0.71250471	0.58898954	1.20970689	2.26E-01	intergenic	LIPC;ADAM10	dist=18692;dist=7638	rs145439370	0.0125	0.0206	0.857914	1
21:23136973:	21	23136973	0.04752427	10	-0.69443215	0.57532419	-1.20702755	2.27E-01	ncRNA_intronic	LINC01425	.	rs73227413	0.0173	0.0277	0.954032	1
6:167501386:	6	167501386	0.00606796	1	-0.2461956	0.20397669	-1.20697911	2.27E-01	intergenic	FGFR1OP;CCR6	dist=45480;dist=23909	rs148153037	0.0107	0.0154	0.844667	1
8:55592336:A	8	55592336	0.63262136	76	1.58213184	1.33625472	1.18400468	2.36E-01	intergenic	RP1;XKR4	dist=48942;dist=422681	rs405226	0.5785	0.6707	0.981155	0.526796
8:55582838:C	8	55582838	0.63262136	76	1.58213184	1.33625472	1.18400468	2.36E-01	intergenic	RP1;XKR4	dist=39444;dist=432179	rs3098298	0.579	0.6705	0.981155	0.526796
8:55587616:T	8	55587616	0.63262136	76	1.58213184	1.33625472	1.18400468	2.36E-01	intergenic	RP1;XKR4	dist=44222;dist=427401	rs367179	0.5779	0.6699	0.981155	0.526796
8:55580298:C	8	55580298	0.63255825	76	1.57973282	1.33643121	1.18205323	2.37E-01	intergenic	RP1;XKR4	dist=36904;dist=434719	rs432393	0.5785	0.6703	0.980895	0.526796
8:55680318:C	8	55680318	0.36259224	75	-1.58182122	1.34109432	-1.17950035	2.38E-01	intergenic	RP1;XKR4	dist=136924;dist=334699	rs2083123	0.3732	0.326	0.991313	0.518201
8:55674617:G	8	55674617	0.3626602	75	-1.58167598	1.34115013	-1.17934297	2.38E-01	intergenic	RP1;XKR4	dist=131223;dist=340400	rs12548593	0.401	0.3263	0.990983	0.518201
8:55685641:A	8	55685641	0.36258738	75	-1.58094881	1.34102424	-1.17891143	2.38E-01	intergenic	RP1;XKR4	dist=142247;dist=329376	rs10958428	0.4012	0.3258	0.991337	0.518201
8:55619508:C	8	55619508	0.36306311	75	-1.57981267	1.34073667	-1.17831689	2.39E-01	intergenic	RP1;XKR4	dist=76114;dist=395509	rs2375537	0.4222	0.3305	0.991097	0.518201
8:55678538:A	8	55678538	0.36407767	75	-1.58650703	1.34702313	-1.17778752	2.39E-01	intergenic	RP1;XKR4	dist=135144;dist=336479	rs1561297	0.4011	0.3261	1	0.53138
8:55629852:T	8	55629852	0.36407767	75	-1.58650703	1.34702313	-1.17778752	2.39E-01	intergenic	RP1;XKR4	dist=86458;dist=385165	rs1437781	0.4243	0.3302	1	0.53138
7:97577830:A	7	97577830	0.00496602	1	-0.22977371	0.19710528	-1.16574102	2.44E-01	intergenic	ASNS;MIR5692A1	dist=75976;dist=15140	rs192750513	0.0017	0.0021	0.977693	1
14:10559057	14	105590577	0.00478155	1	-0.15912936	0.13727357	-1.15921342	2.46E-01	intergenic	LOC102723354;JAG2	dist=24744;dist=16741	rs190251199	0.0007	0.0012	0.479557	1
6:167513471:	6	167513471	0.00788835	2	-0.2748431	0.23718786	-1.15875705	2.47E-01	intergenic	FGFR1OP;CCR6	dist=57565;dist=11824	rs184487573	0.0111	0.0159	0.851684	1
7:97488823:C	7	97488823	0.00485437	1	-0.2274835	0.19712797	-1.15398893	2.49E-01	intronic	ASNS	.	rs181259864	0.0017	0.0021	1	1
17:48508221:	17	48508221	0.02424757	5	0.51565366	0.45058422	1.14441129	2.52E-01	intronic	ACSF2	.	rs184613584	0.0079	0.012	0.992901	1
3:28227423:T	3	28227423	0.02367961	5	-0.43930097	0.39074283	-1.12427134	2.61E-01	intergenic	LOC100996624;CMC1	dist=351796;dist=55701	rs146007933	0.0187	0.0233	0.830561	1
15:58951660:	15	58951660	0.04635437	10	0.56667404	0.50817586	1.11511404	2.65E-01	intronic	ADAM10	.	rs149425014	0.0102	0.0173	0.759352	1
8:55691458:C	8	55691458	0.35292233	73	-1.50681641	1.35767381	-1.10985157	2.67E-01	intergenic	RP1;XKR4	dist=148064;dist=323559	rs4737201	0.3056	0.323	0.990899	0.825756
8:55690220:T	8	55690220	0.3529466	73	-1.50258745	1.35814603	-1.10635191	2.69E-01	intergenic	RP1;XKR4	dist=146826;dist=324797	rs7822082	0.292	0.3259	0.991442	0.825756
8:55590975:G	8	55590975	0.63752427	75	1.47054953	1.33573729	1.10092721	2.71E-01	intergenic	RP1;XKR4	dist=47581;dist=424042	rs382476	0.6871	0.6731	0.981044	0.669247
8:55591609:G	8	55591609	0.63752427	75	1.47054953	1.33573729	1.10092721	2.71E-01	intergenic	RP1;XKR4	dist=48215;dist=423408	rs384543	0.6874	0.6732	0.981044	0.669247
8:55571940:A	8	55571940	0.63752913	75	1.46958173	1.33575453	1.10018847	2.71E-01	intergenic	RP1;XKR4	dist=28546;dist=443077	rs369623	0.6874	0.6729	0.981058	0.669247
8:55681876:T	8	55681876	0.35778156	74	-1.47304892	1.3405121	-1.09887029	2.72E-01	intergenic	RP1;XKR4	dist=138482;dist=333141	rs1391463	0.3108	0.3238	0.991203	0.662691
8:55597489:G	8	55597489	0.63731068	75	1.46895537	1.33738957	1.09837508	2.72E-01	intergenic	RP1;XKR4	dist=54095;dist=417528	rs384127	0.6871	0.6739	0.980706	0.669247
8:55679546:G	8	55679546	0.35778156	74	-1.47207174	1.34069826	-1.09798884	2.72E-01	intergenic	RP1;XKR4	dist=136152;dist=335471	rs4737676	0.3059	0.3233	0.991451	0.662691
8:55680792:C	8	55680792	0.35778156	74	-1.47207174	1.34069826	-1.09798884	2.72E-01	intergenic	RP1;XKR4	dist=137398;dist=334225	rs983248	0.3066	0.3238	0.991451	0.662691
8:55669829:A	8	55669829	0.35778641	74	-1.4718121	1.34072426	-1.0977739	2.72E-01	intergenic	RP1;XKR4	dist=126435;dist=345188	rs11987234	0.2969	0.3231	0.991224	0.662691
8:55688174:G	8	55688174	0.35774757	74	-1.47128038	1.3406321	-1.09745274	2.72E-01	intergenic	RP1;XKR4	dist=144780;dist=326843	rs13276543	0.2881	0.3202	0.991558	0.662691
8:55674149:G	8	55674149	0.3578301	74	-1.47115141	1.34056307	-1.09741305	2.72E-01	intergenic	RP1;XKR4	dist=130755;dist=340868	rs13277510	0.306	0.323	0.991119	0.662691
8:55678434:G	8	55678434	0.35777185	74	-1.47134764	1.34078154	-1.09738059	2.72E-01	intergenic	RP1;XKR4	dist=135040;dist=336583	rs16920698	0.3061	0.3233	0.991493	0.662691
8:55614690:A	8	55614690	0.35821845	74	-1.47023155	1.3401521	-1.0970632	2.73E-01	intergenic	RP1;XKR4	dist=71296;dist=400327	rs858397	0.2987	0.3262	0.991085	0.662691
8:55688171:C	8	55688171	0.3592233	74	-1.47572798	1.34660514	-1.09588768	2.73E-01	intergenic	RP1;XKR4	dist=144777;dist=326846	rs13278605	0.2879	0.32	1	0.671646
8:55632762:C	8	55632762	0.3592233	74	-1.47572798	1.34660514	-1.09588768	2.73E-01	intergenic	RP1;XKR4	dist=89368;dist=382255	rs1437782	0.3139	0.3281	1	0.671646
8:55574960:G	8	55574960	0.6407767	74	1.47572798	1.34660514	1.09588768	2.73E-01	intergenic	RP1;XKR4	dist=31566;dist=440057	rs446222	0.6836	0.6727	1	0.671646
8:55661654:C	8	55661654	0.35807282	74	-1.46115439	1.33994925	-1.09045502	2.76E-01	intergenic	RP1;XKR4	dist=118260;dist=353363	rs4737674	0.3142	0.3243	0.990315	0.662691
3:148330710:	3	148330710	0.00986893	2	0.31483129	0.29092366	1.08217836	2.79E-01	intergenic	LOC440982;AGTR1	dist=1103013;dist=84948	rs193153124	0.0046	0.0071	0.978098	1
10:3472846:C	10	3472846	0.00759709	2	0.23535612	0.21966033	1.07145481	2.84E-01	ncRNA_intronic	LOC105376360	.	rs118040657	0.0038	0.0061	0.75025	1
4:90957372:G	4	90957372	0.01477185	3	0.3764391	0.35152562	1.07087246	2.84E-01	intergenic	MMRN1;CCSER1	dist=81592;dist=91312	rs142993106	0.0141	0.0158	0.985319	1
4:35572280:C	4	35572280	0.00960194	2	0.30233113	0.28236665	1.0707041	2.84E-01	intergenic	LOC101928622;ARAP2	dist=1530765;dist=495340	rs143287889	0.003	0.0051	0.988019	1
15:58837933:	15	58837933	0.06800486	14	0.7150937	0.66861064	1.06952186	2.85E-01	intronic	LIPC	.	rs6080	0.0208	0.0325	0.926378	1

5:177390937:	5	177390937	0.00842233	2	-0.26338408	0.24665417	-1.06782741	2.86E-01	intergenic	LOC728554;PROP1	dist=79668;dist=28299	rs151015676	0.0028	0.0041	0.793454	1
4:87450675:T	4	87450675	0.01436893	3	-0.35002974	0.32934748	-1.06279767	2.88E-01	intergenic	MAPK10;MIR4452	dist=76392;dist=12960	rs147630370	0.0042	0.0069	0.846233	1
16:76179362:	16	76179362	0.00514078	1	0.21213859	0.19985807	1.06144616	2.88E-01	intergenic	TERF2IP;CNTNAP4	dist=488021;dist=131814	rs144954214	0.012	0.0091	0.86118	1
8:55640472:C	8	55640472	0.35365049	73	-1.4121439	1.3487482	-1.04700336	2.95E-01	intergenic	RP1;XKR4	dist=97078;dist=374545	rs10105693	0.2971	0.3231	0.991111	0.825756
2:48312950:G	2	48312950	0.01345146	3	0.34321802	0.33187281	1.03418543	3.01E-01	intergenic	FBXO11;FOXN2	dist=180018;dist=228845	rs76777840	0.0027	0.0042	0.92873	1
15:58982115:	15	58982115	0.04498058	9	0.5128583	0.49958433	1.02657003	3.05E-01	intronic	ADAM10	.	rs146442492	0.0093	0.0159	0.750996	1
10:96217535:	10	96217535	0.00973786	2	-0.29443974	0.29332166	-1.00381179	3.15E-01	intronic	TBC1D12	.	rs140706881	0.0099	0.0125	0.989983	1
1:103753974:	1	103753974	0.01480097	3	0.24859719	0.25571623	0.97216041	3.31E-01	intergenic	COL11A1;LOC101928436	dist=179922;dist=206727	rs113221952	0.0148	0.0207	0.596434	1
4:154411043:	4	154411043	0.00691748	1	-0.1809627	0.18830796	-0.96099339	3.37E-01	intronic	KIAA0922	.	rs531769270	0.0009	0.0013	0.587973	1
2:177116420:	2	177116420	0.0049466	1	0.1849834	0.19319589	0.95749136	3.38E-01	intergenic	HOXD1;MTX2	dist=60785;dist=17703	rs184098071	0.0024	0.0035	0.909749	1
2:126393864:	2	126393864	0.0048835	1	-0.1835457	0.19343847	-0.94885831	3.43E-01	intergenic	CNTNAP5;GYPC	dist=720910;dist=1019647	rs116189766	0.004	0.006	0.992027	1
4:89135588:A	4	89135588	0.01553884	3	-0.30708339	0.32653729	-0.94042364	3.47E-01	intronic	ABCG2	.	rs147171192	0.0053	0.0061	0.870597	1
12:49853998:	12	49853998	0.00226214	0	-0.08861555	0.09615902	-0.92155213	3.57E-01	intronic	SPATS2	.	rs568658857	0.0019	0.0036	0.464789	1
13:10801298!	13	108012985	0.00766019	2	-0.20567337	0.23037643	-0.89277127	3.72E-01	intronic	FAM155A	.	rs572961122	0.0029	0.0042	0.822166	1
4:90993018:T	4	90993018	0.01207282	2	0.2641875	0.29872262	0.8843907	3.76E-01	intergenic	MMRN1;CCSER1	dist=117238;dist=55666	rs146526206	0.0145	0.0161	0.885605	1
1:205331874:	1	205331874	0.00485437	1	0.18816409	0.21525414	0.87414854	3.82E-01	intergenic	KLHDC8A;LEMD1-AS1	dist=5656;dist=10506	rs10494861	0.0305	0.0033	1	1
3:177574308:	3	177574308	0.00485922	1	0.18000965	0.20700024	0.86961084	3.85E-01	ncRNA_intronic	KCCAT211	.	rs189709453	0.002	0.0034	0.998997	1
12:28248852:	12	28248852	0.0097233	2	-0.25519409	0.29372871	-0.86880879	3.85E-01	intergenic	PTHLH;CCDC91	dist=123936;dist=161281	rs77353774	0.0047	0.0069	0.99849	1
3:177712448:	3	177712448	0.00485437	1	0.17982452	0.20699733	0.86872868	3.85E-01	intergenic	KCCAT211;LINC01014	dist=95436;dist=424541	rs182868205	0.0026	0.0045	1	1
3:177639777:	3	177639777	0.00485437	1	0.17982452	0.20699733	0.86872868	3.85E-01	intergenic	KCCAT211;LINC01014	dist=22765;dist=497212	rs186767531	0.0027	0.0044	1	1
12:26821687:	12	26821687	0.00485437	1	0.17982452	0.20699733	0.86872868	3.85E-01	intronic	ITPR2	.	rs183466664	0.0039	0.0046	1	1
13:10801135	13	108011352	0.00770388	2	-0.19963877	0.23042389	-0.86639788	3.86E-01	intronic	FAM155A	.	rs184265355	0.0023	0.0033	0.817644	1
1:185129502:	1	185129502	0.00303398	1	-0.08542469	0.0996576	-0.85718185	3.91E-01	intronic	SWT1	.	rs145766563	0.0007	0.0009	0.364085	1
1:184955657:	1	184955657	0.0019466	0	-0.06342383	0.07405374	-0.85645693	3.92E-01	intergenic	FAM129A;RNF2	dist=11939;dist=58894	rs138480898	0.0008	0.001	0.31212	1
12:20424749:	12	20424749	0.00976214	2	-0.24642395	0.28890319	-0.85296376	3.94E-01	intergenic	LOC100506393;PDE3A	dist=172947;dist=97430	rs118184666	0.005	0.0075	0.982689	1
12:28511473:	12	28511473	0.00980583	2	-0.25000717	0.29372451	-0.8511621	3.95E-01	intronic	CCDC91	.	rs117991215	0.0044	0.0065	0.990095	1
12:28435962:	12	28435962	0.00981553	2	-0.24986877	0.29370617	-0.85074401	3.95E-01	intronic	CCDC91	.	rs113167689	0.0047	0.0069	0.989132	1
7:89497045:T	7	89497045	0.02540777	5	-0.37731499	0.4449349	-0.8480229	3.96E-01	intergenic	ZNF804B;STEAP2-AS1	dist=530674;dist=14622	rs111391231	0.0077	0.0123	0.929489	1
12:28468969:	12	28468969	0.0098301	2	-0.24869443	0.29371565	-0.84671574	3.97E-01	intronic	CCDC91	.	rs17510814	0.005	0.007	0.98766	1
12:28511096:	12	28511096	0.0098301	2	-0.24817368	0.29372682	-0.84491322	3.98E-01	intronic	CCDC91	.	rs141756120	0.005	0.0071	0.987665	1
7:89516669:G	7	89516669	0.02492719	5	-0.36944585	0.43781414	-0.84384174	3.99E-01	ncRNA_intronic	STEAP2-AS1	.	rs111900874	0.0077	0.0123	0.916811	1
13:23887014:	13	23887014	0.00415534	1	-0.14213336	0.16933337	-0.83937004	4.01E-01	intronic	SGCG	.	rs139598422	0.003	0.0047	0.764294	1
4:990967:C:T	4	990967	0.00427185	1	-0.14854758	0.17859567	-0.83175355	4.06E-01	intronic	IDUA	.	rs113651406	0.0025	0.0041	0.847816	1
11:99188380:	11	99188380	0.02467476	5	0.36739406	0.44665973	0.82253678	4.11E-01	intronic	CNTN5	.	rs112007361	0.0222	0.0289	0.976846	1
18:55497457:	18	55497457	0.00826699	2	-0.19784548	0.24068482	-0.82201064	4.11E-01	intergenic	ATP8B1;NEDD4L	dist=27130;dist=214153	rs146333745	0.0026	0.0042	0.804458	1
20:15858501:	20	15858501	0.0188932	4	-0.31907799	0.38923887	-0.81974853	4.12E-01	intronic	MACROD2	.	rs140788628	0.0068	0.0107	0.930617	1
1:79880089:G	1	79880089	0.00569418	1	-0.11994275	0.14942111	-0.80271621	4.22E-01	intergenic	ADGRL4;LOC101927412	dist=407594;dist=1121351	rs149493615	0.0081	0.012	0.311201	1
12:20151132:	12	20151132	0.01435922	3	-0.28274382	0.35330677	-0.80027854	4.24E-01	intergenic	AEBP2;LOC100506393	dist=475959;dist=16487	rs141754456	0.0061	0.0088	0.984648	1
11:99089147:	11	99089147	0.02435437	5	0.35240798	0.44618974	0.78981641	4.30E-01	intronic	CNTN5	.	rs74521112	0.0221	0.0288	0.986931	1
11:99093455:	11	99093455	0.02435437	5	0.35229432	0.44618837	0.78956412	4.30E-01	intronic	CNTN5	.	rs79213709	0.0223	0.0291	0.986921	1
1:18948328:C	1	18948328	0.00481553	1	0.1618529	0.20583153	0.7863368	4.32E-01	intergenic	KLHDC7A;PAX7	dist=135848;dist=9172	rs186532456	0.0043	0.0063	0.991961	1
1:18959333:A	1	18959333	0.00481553	1	0.1618529	0.20583153	0.7863368	4.32E-01	intronic	PAX7	.	rs562032622	0.0049	0.007	0.991961	1
3:136134595:	3	136134595	0.00367476	1	-0.11370888	0.14607079	-0.77845055	4.36E-01	intronic	STAG1	.	rs148248743	0.0018	0.0025	0.67579	1
2:223814861:	2	223814861	0.00459709	1	0.14317546	0.18417463	0.77738969	4.37E-01	intergenic	ACSL3;KCNE4	dist=6742;dist=101787	rs185510569	0.0033	0.0052	0.882334	1
11:95099866:	11	95099866	0.01479126	3	-0.2745327	0.35316263	-0.7773549	4.37E-01	intergenic	LOC100129203;FAM76B	dist=132298;dist=402240	rs149949098	0.0154	0.0228	0.947369	1
14:46294660:	14	46294660	0.27903398	57	1.01329024	1.31694435	0.76942525	4.42E-01	intergenic	MIS18BP1;LINC00871	dist=572055;dist=238702	rs428110	0.2639	0.3041	0.993976	0.624668
3:135532345:	3	135532345	0.00469418	1	-0.14923903	0.19399589	-0.76928965	4.42E-01	intergenic	EPHB1;PPP2R3A	dist=553038;dist=152170	rs192443987	0.0019	0.0025	0.933075	1
1:89370702:G	1	89370702	0.02508738	5	-0.29457778	0.38461758	-0.76589785	4.44E-01	intergenic	GTF2B;CCBL2	dist=13401;dist=30754	rs34270375	0.0157	0.0211	0.688983	1
9:129042336:	9	129042336	0.00452427	1	0.14328556	0.18753726	0.7640378	4.45E-01	intergenic	LOC101929116;MVB12B	dist=4142;dist=46787	rs146207930	0.0174	0.024	0.931691	1
18:27001580:	18	27001580	0.00237864	0	0.07277788	0.09580724	0.75962812	4.47E-01	intergenic	CDH2;MIR302F	dist=1244170;dist=877296	rs185819304	0.0025	0.0021	0.46127	1
18:27030430:	18	27030430	0.0019466	0	0.05910881	0.07809365	0.75689647	4.49E-01	intergenic	CDH2;MIR302F	dist=1273020;dist=848446	rs187942235	0.0023	0.002	0.374316	1
8:25613298:C	8	25613298	0.00600971	1	-0.1326355	0.17748976	-0.74728537	4.55E-01	intergenic	CDCA2;EBF2	dist=247873;dist=85948	rs188415494	0.0019	0.0033	0.644528	1
4:23509067:T	4	23509067	0.0051699	1	0.14590427	0.19775652	0.73779751	4.61E-01	intergenic	MIR548AJ2;PPARGC1A	dist=44341;dist=284577	rs113063005	0.0035	0.0047	0.828627	1
7:17052778:G	7	17052778	0.00495146	1	-0.15159745	0.20607567	-0.73563974	4.62E-01	intergenic	AGR3;AHR	dist=131165;dist=285498	rs117166500	0.0057	0.0054	0.980476	1
14:46280913:	14	46280913	0.27882524	57	0.94398138	1.30478252	0.72347795	4.69E-01	intergenic	MIS18BP1;LINC00871	dist=558308;dist=252449	rs176783	0.2781	0.3092	0.982613	0.803044
12:1870510:A	12	1870510	0.00532524	1	0.14689221	0.20312563	0.72315942	4.70E-01	intronic	ADIPOR2	.	rs189360484	0.0003	0.0005	0.907353	1
14:46282970:	14	46282970	0.2815534	58	0.94410239	1.31947222	0.71551517	4.74E-01	intergenic	MIS18BP1;LINC00871	dist=560365;dist=250392	rs176786	0.2792	0.3091	1	0.634039
2:95967628:G	2	95967628	0.0575534	12	0.55813521	0.78126016	0.71440377	4.75E-01	intronic	KCNIP3	.	rs76554191	0.0321	0.044	0.983166	0.169814
1:181247121:	1	181247121	0.01397087	3	-0.2247907	0.31663714	-0.70993157	4.78E-01	intergenic	GM140;CACNA1E	dist=39381;dist=205565	rs183180157	0.0045	0.0053	0.835566	1
20:15813704:	20	15813704	0.0290534	6	0.34861589	0.49308591	0.70700841	4.80E-01	intronic	MACROD2	.	rs2876414	0.0327	0.0266	0.996403	1
14:46260982:	14	46260982	0.71485437	59	-0.92281342	1.30945611	-0.70473032	4.81E-01	intergenic	MIS18BP1;LINC00871	dist=538377;dist=272380	rs1686289	0.6998	0.6934	0.977943	0.807255

20:15813491:	20	15813491	0.02942718	6	0.34556664	0.49252048	0.70162899	4.83E-01	intronic	MACROD2	.	rs2327968	0.0388	0.0336	0.983503	1
1:104114545:	1	104114545	0.00949515	2	0.1690099	0.24351883	0.69403217	4.88E-01	intronic	AMY2B	.	rs1856085	0.0047	0.0013	0.930312	1
1:104157143:	1	104157143	0.00958738	2	0.17118256	0.2471166	0.69271977	4.88E-01	intergenic	AMY2B;AMY2A	dist=34987;dist=2811	rs143597860	0.0048	0.0014	0.948665	1
1:104310729:	1	104310729	0.00925728	2	0.16424125	0.23779794	0.69067566	4.90E-01	intergenic	AMY1A;LOC100129138	dist=9418;dist=304916	rs144541665	0.0048	0.0013	0.909477	1
1:79968131:T	1	79968131	0.00691748	1	-0.1243414	0.18038128	-0.68932542	4.91E-01	intergenic	ADGRL4;LOC101927412	dist=495636;dist=1033309	rs143811231	0.0073	0.0122	0.488277	1
7:100668425:	7	100668425	0.00264078	1	-0.07336753	0.107454	-0.68278085	4.95E-01	intronic	MUC17	.	rs188028357	0.0012	0.0005	0.492675	1
22:45823032:	22	45823032	0.00218932	0	0.06223473	0.09281172	0.67054821	5.03E-01	intronic	RIBC2	.	rs182959028	0.0055	0.0093	0.447795	1
6:134833127:	6	134833127	0.00968932	2	0.19262125	0.28806153	0.66868094	5.04E-01	intergenic	LINC01010;LOC101928304	dist=7969;dist=13329	rs141326851	0.0152	0.0166	0.993965	1
4:21259643:A	4	21259643	0.00308738	1	-0.07272364	0.10889196	-0.66785139	5.04E-01	intronic	KCNIP4	.	rs183962155	0.0027	0.004	0.43349	1
3:1626661:T:(	3	1626661	0.00864078	2	0.14649408	0.21943009	0.66761162	5.04E-01	intergenic	CNTN6;CNTN4	dist=181369;dist=513889	rs112475378	0.0133	0.0218	0.650295	1
10:82359100:	10	82359100	0.00753398	2	-0.15711058	0.23632408	-0.66480988	5.06E-01	intronic	SH2D4B	.	rs140277951	0.0046	0.0063	0.833841	1
2:48473143:C	2	48473143	0.00489806	1	-0.13344564	0.20718978	-0.64407443	5.20E-01	intergenic	FBXO11;FOXN2	dist=340211;dist=68652	rs145080832	0.0023	0.0035	0.991117	1
2:48631743:C	2	48631743	0.00489806	1	-0.13344564	0.20718978	-0.64407443	5.20E-01	intergenic	FOXN2;PPP1R21	dist=25309;dist=36165	rs184220112	0.0021	0.0032	0.991117	1
12:21012024:	12	21012024	0.00468447	1	-0.12737119	0.19849718	-0.64167754	5.21E-01	intronic	SLCO1B3	.	rs151323346	0.0013	0.0021	0.950859	1
10:10295615;	10	102956152	0.04854369	10	-0.38863869	0.61565434	-0.63126119	5.28E-01	intergenic	LINC01514;LBX1	dist=2248;dist=30581	rs75334617	0.0323	0.0466	0.99979	1
2:223650026:	2	223650026	0.00842718	2	-0.15458772	0.2482031	-0.62282751	5.33E-01	intergenic	MOGAT1;ACSL3	dist=75377;dist=75706	rs185158855	0.0044	0.0069	0.854385	1
2:15682724:C	2	15682724	0.00928155	2	-0.16218645	0.2646395	-0.61285806	5.40E-01	intronic	NBAS	.	rs558553658	0.003	0.0053	0.953147	1
3:164808462:	3	164808462	0.00857282	2	-0.14648565	0.23949598	-0.61164138	5.41E-01	intergenic	SI;SLITRK3	dist=12179;dist=96046	rs141169929	0.0036	0.0053	0.774857	1
5:73285489:T	5	73285489	0.00504854	1	-0.11957494	0.1957184	-0.61095399	5.41E-01	intergenic	ARHGEF28;LINC01335	dist=47671;dist=316746	rs185771987	0.0027	0.0041	0.85912	1
22:46853180:	22	46853180	0.00681553	1	-0.11713689	0.19236142	-0.60894169	5.43E-01	intronic	CELSR1	.	rs150946694	0.0051	0.0062	0.629526	1
13:91411079:	13	91411079	0.0158932	3	0.21782246	0.36676046	0.59390933	5.53E-01	intergenic	LINC01049;LINC00410	dist=223484;dist=132129	rs546286713	0.0038	0.0055	0.935869	1
2:53483429:G	2	53483429	0.01595631	3	-0.19860931	0.34469792	-0.57618367	5.64E-01	intergenic	MIR4431;ASB3	dist=553676;dist=413688	rs149421869	0.0032	0.0047	0.902614	1
15:94392882:	15	94392882	0.00485437	1	0.12054613	0.21073279	0.57203309	5.67E-01	intergenic	RGMA;LOC101927153	dist=760439;dist=6907	rs138217865	0.0013	0.0021	1	1
5:73372109:A	5	73372109	0.00487864	1	-0.11618831	0.20721267	-0.56072008	5.75E-01	intergenic	ARHGEF28;LINC01335	dist=134291;dist=230126	rs139360368	0.0031	0.0041	0.995013	1
11:66665729:	11	66665729	0.00576214	1	-0.09669872	0.17269163	-0.55995021	5.76E-01	intronic	PC	.	rs181812512	0.0026	0.0045	0.831451	1
8:22322319:A	8	22322319	0.00464563	1	0.10981454	0.19724	0.55675591	5.78E-01	intronic	PPP3CC	.	rs147601511	0.002	0.0036	0.915529	1
2:108038112:	2	108038112	0.00713592	1	0.11343718	0.20593234	0.55084683	5.82E-01	intergenic	MIR548AU;GACAT1	dist=72131;dist=332456	rs140352232	0.0021	0.0032	0.724891	1
1:99455745:T	1	99455745	0.01333981	3	-0.17828798	0.32573131	-0.54734677	5.84E-01	intronic	PLPPR5	.	rs187518659	0.0043	0.0068	0.924329	1
1:193044178:	1	193044178	0.00491262	1	-0.103734	0.1980264	-0.52383928	6.00E-01	intronic	TROVE2	.	rs180989936	0.0017	0.0029	0.880828	1
12:10118874	12	101188744	0.01456311	3	-0.17403446	0.33240476	-0.52356189	6.01E-01	UTR5	ANO4	NM_178826:c.-106820G>A;NM_	rs76904423	0.0146	0.0193	1	1
12:10118296(	12	101182966	0.01448544	3	-0.17284956	0.3309544	-0.52227606	6.01E-01	intergenic	GAS2L3;ANO4	dist=160900;dist=5408	rs76327548	0.0293	0.0223	0.994618	1
15:59435086:	15	59435086	0.03308738	7	0.24283277	0.48819428	0.49741011	6.19E-01	intronic	MYO1E	.	rs80292573	0.0289	0.025	0.830242	1
4:2426305:T:(	4	2426305	0.00950971	2	-0.13017179	0.26223206	-0.49639921	6.20E-01	intronic	CFAP99	.	rs12502861	0.0205	0.0183	0.855507	1
12:63306297:	12	63306297	0.00448544	1	-0.08561271	0.18168687	-0.47121024	6.37E-01	intronic	PPM1H	.	rs137880949	0.0011	0.0019	0.834481	1
1:112354418:	1	112354418	0.01467961	3	0.16005174	0.34143383	0.46876357	6.39E-01	intronic	KCND3	.	rs74683551	0.0237	0.0165	0.991424	1
1:112349372:	1	112349372	0.01471845	3	0.15899613	0.34146237	0.46563294	6.41E-01	intronic	KCND3	.	rs76098744	0.0239	0.0167	0.988834	1
10:12453340(	10	124533409	0.00937379	2	-0.12917376	0.27778761	-0.46500908	6.42E-01	ncRNA_intronic	DMBT1P1	.	rs180828621	0.0022	0.0035	0.959645	1
9:85945465:C	9	85945465	0.00970874	2	-0.13617591	0.29512332	-0.46142038	6.44E-01	intronic	FRMD3	.	rs190294315	0.0073	0.0119	1	1
9:85937714:G	9	85937714	0.00960194	2	-0.13458944	0.2917304	-0.46134869	6.45E-01	intronic	FRMD3	.	rs188034471	0.0061	0.0101	0.987885	1
2:95722609:C	2	95722609	0.05342233	11	0.3575444	0.77672804	0.46032122	6.45E-01	intergenic	MAL;MRPS5	dist=2872;dist=30343	rs17746486	0.0239	0.0341	0.999522	0.0225883
2:176950420:	2	176950420	0.00808252	2	0.10253581	0.23091352	0.44404422	6.57E-01	intergenic	EVX2;HOXD13	dist=1730;dist=7112	rs556293455	0.0025	0.0034	0.832379	1
5:163268244:	5	163268244	0.0051068	1	-0.08900504	0.20103246	-0.44273967	6.58E-01	intergenic	MAT2B;LOC101927835	dist=321885;dist=607184	rs290120	0.0026	0.0041	0.951037	1
18:29050262:	18	29050262	0.00488835	1	0.09200424	0.20908917	0.44002393	6.60E-01	intronic	DSG3	.	rs143538552	0.0034	0.0051	0.993025	1
18:29058384:	18	29058384	0.00489806	1	0.09185895	0.20908607	0.43933555	6.60E-01	UTR3	DSG3	NM_001944:c.*2161C>A	rs373746073	0.0034	0.0051	0.991053	1
7:100474786:	7	100474786	0.00286893	1	-0.04219979	0.09659454	-0.43687546	6.62E-01	intronic	SRRT	.	rs539713344	0.0017	0.0016	0.366221	1
11:67110852:	11	67110852	0.00853398	2	-0.09278968	0.21503911	-0.43150141	6.66E-01	ncRNA_intronic	LOC100130987	.	rs529345909	0.0027	0.0046	0.784094	1
7:49544747:A	7	49544747	0.00566019	1	-0.08265789	0.20216681	-0.40885986	6.83E-01	intergenic	CDC14C;VWC2	dist=577698;dist=268510	rs574076561	0.0012	0.0023	0.74363	1
18:28816019:	18	28816019	0.00424757	1	0.07214139	0.1775047	0.40641962	6.84E-01	intergenic	DSC1;DSG1	dist=73200;dist=82033	rs139493286	0.0022	0.0029	0.823631	1
3:33940571:A	3	33940571	0.06219417	13	0.25709647	0.65361904	0.393343	6.94E-01	intergenic	PDCD6IP;LOC101928135	dist=29372;dist=976718	rs73057656	0.0413	0.0633	0.850362	1
22:32824278:	22	32824278	0.00672816	1	-0.0796672	0.21324038	-0.37360276	7.09E-01	intronic	BPIFC	.	rs78547898	0.0862	0.0418	0.735175	1
14:81717563:	14	81717563	0.00485922	1	-0.07380478	0.19779175	-0.37314389	7.09E-01	intergenic	GTF2A1;STON2	dist=29988;dist=9431	rs113767990	0.0061	0.0091	0.994994	1
10:12477927	10	124779274	0.0145534	3	0.12543817	0.34800648	0.36044779	7.19E-01	intronic	ACADSB	.	rs147393020	0.0038	0.0058	0.999324	1
1:62484462:G	1	62484462	0.01630097	3	0.11681659	0.32574933	0.35860884	7.20E-01	intronic	INADL	.	rs2365739	0.0268	0.0259	0.907808	1
5:161493182:	5	161493182	0.00951456	2	0.08538779	0.23837656	0.35820547	7.20E-01	intergenic	LINC01202;GABRG2	dist=64980;dist=1466	rs74343174	0.0055	0.008	0.676915	1
2:67702707:G	2	67702707	0.00943204	2	0.09036869	0.26393369	0.34239166	7.32E-01	intergenic	ETAA1;LOC101927701	dist=65174;dist=320479	rs186142189	0.0034	0.0047	0.971958	1
2:67682724:G	2	67682724	0.00944175	2	0.08898472	0.26330544	0.33795246	7.35E-01	intergenic	ETAA1;LOC101927701	dist=45191;dist=340462	rs151272830	0.0031	0.0044	0.967052	1
2:151438271:	2	151438271	0.01	2	-0.09249531	0.294759	-0.31379979	7.54E-01	intergenic	LOC101929260;LOC101929	dist=9536;dist=47140	rs142894171	0.0041	0.0067	0.969366	1
1:229826378:	1	229826378	0.0314466	6	-0.1482821	0.49541331	-0.29930988	7.65E-01	intergenic	URB2;GALNT2	dist=30431;dist=367158	rs12036586	0.1365	0.0478	0.954664	1
10:20207052:	10	20207052	0.01941748	4	-0.12169638	0.41089326	-0.29617517	7.67E-01	intronic	PLXDC2	.	rs117025967	0.0094	0.0101	1	1
1:102803484:	1	102803484	0.00400971	1	-0.04914759	0.1667589	-0.29472244	7.68E-01	intergenic	OLFM3;COL11A1	dist=340694;dist=538539	rs140420703	0.0042	0.0066	0.785826	1
7:11711845:G	7	11711845	0.00747087	2	-0.0681348	0.23499403	-0.28994267	7.72E-01	intronic	THSD7A	.	rs187978759	0.0012	0.0022	0.828633	1

7:6426479:G:	7	6426479	0.02857282	6	-0.12434427	0.44149559	-0.28164329	7.78E-01	intronic	RAC1	.	rs17776100	0.028	0.0338	0.781514	1
6:99173116:A	6	99173116	0.00428155	1	-0.04769256	0.17560257	-0.27159375	7.86E-01	intergenic	MIR2113;POU3F2	dist=700621;dist=109464	rs147627638	0.0055	0.0081	0.81131	1
4:112677596:	4	112677596	0.0119466	2	-0.06454508	0.25063805	-0.25752307	7.97E-01	intergenic	PITX2;C4orf32	dist=1114317;dist=388957	rs145116559	0.0023	0.0041	0.636852	1
19:23123198:	19	23123198	0.01480097	3	0.08187372	0.31929389	0.25642121	7.98E-01	intergenic	ZNF99;ZNF728	dist=156225;dist=34487	rs111285015	0.0062	0.0102	0.724747	1
3:195646605:	3	195646605	0.01941748	4	0.0978037	0.38203395	0.25600787	7.98E-01	intergenic	TNK2;SDHAP1	dist=10725;dist=40187	rs191792521	0.0062	0.0105	0.863354	1
4:112689266:	4	112689266	0.0119466	2	-0.0609985	0.25169008	-0.24235559	8.09E-01	intergenic	PITX2;C4orf32	dist=1125987;dist=377287	rs181415102	0.0019	0.0033	0.642191	1
11:12369302	11	123693024	0.01457282	3	0.08589684	0.36200104	0.2372834	8.12E-01	intergenic	OR6M1;TMEM225	dist=15967;dist=60609	rs141281289	0.0053	0.0089	0.994625	1
6:142307100:	6	142307100	0.01361165	3	0.05884836	0.25543344	0.23038627	8.18E-01	intergenic	MIR4465;NMBR	dist=1302080;dist=89645	rs72983831	0.0225	0.0173	0.660998	1
4:139140860:	4	139140860	0.03605825	7	-0.11040444	0.49326313	-0.22382462	8.23E-01	intronic	SLC7A11	.	rs112679237	0.0142	0.0215	0.854571	1
6:165514281:	6	165514281	0.00637379	1	-0.04360284	0.19925364	-0.21883085	8.27E-01	intergenic	MEAT6;C6orf118	dist=278729;dist=178872	rs117498042	0.0042	0.0043	0.737781	1
15:59229353:	15	59229353	0.01822816	4	0.07070707	0.326751	0.21639435	8.29E-01	intergenic	SLTM;RNF111	dist=3501;dist=50512	rs193253461	0.0039	0.0067	0.779464	1
6:98092675:T	6	98092675	0.00759223	2	-0.04473159	0.21551523	-0.20755653	8.36E-01	ncRNA_intronic	LOC101927314	.	rs56224400	0.0082	0.0137	0.681669	1
12:63608466:	12	63608466	0.00901456	2	-0.04938488	0.24082995	-0.20506121	8.38E-01	intergenic	AVPR1A;DPY19L2	dist=61876;dist=344227	rs182437250	0.002	0.0033	0.739218	1
15:59404306:	15	59404306	0.01714078	4	0.06477319	0.32705492	0.19804989	8.43E-01	intronic	CCNB2	.	rs184117160	0.0036	0.0062	0.821999	1
12:63445280:	12	63445280	0.01001456	2	-0.05165032	0.26703588	-0.1934209	8.47E-01	intergenic	PPM1H;AVPR1A	dist=116615;dist=91259	rs191053292	0.002	0.0031	0.818462	1
21:35477486:	21	35477486	0.03398058	7	0.09515843	0.53639433	0.17740386	8.59E-01	UTR3	SLC5A3	NM_006933:c.*7832C>T	rs118183140	0.0133	0.0202	1	1
6:165499857:	6	165499857	0.00646602	1	-0.03576208	0.20752178	-0.17232927	8.63E-01	intergenic	MEAT6;C6orf118	dist=264305;dist=193296	rs148532212	0.0029	0.0035	0.785224	1
6:142611258:	6	142611258	0.01621359	3	-0.05521243	0.32412283	-0.17034416	8.65E-01	intergenic	VTA1;ADGRG6	dist=69173;dist=11798	rs72986533	0.0083	0.014	0.839327	1
3:141841196:	3	141841196	0.00939806	2	-0.04205967	0.26798504	-0.15694783	8.75E-01	intronic	TFDP2	.	rs576124203	0.0017	0.0028	0.88872	1
5:91631888:A	5	91631888	0.00402427	1	0.02563369	0.16856192	0.15207283	8.79E-01	intergenic	ARRDC3-AS1;NR2F1-AS1	dist=915356;dist=1113174	rs183816745	0.0085	0.0153	0.828309	1
5:91530447:G	5	91530447	0.00445631	1	0.02838568	0.18665843	0.15207283	8.79E-01	intergenic	ARRDC3-AS1;NR2F1-AS1	dist=813915;dist=1214615	rs187236873	0.0085	0.0153	0.917633	1
5:91465647:A	5	91465647	0.00482524	1	0.03073569	0.20211164	0.15207283	8.79E-01	intergenic	ARRDC3-AS1;NR2F1-AS1	dist=749115;dist=1279415	rs181933850	0.0085	0.0151	0.993971	1
5:91475485:G	5	91475485	0.00477185	1	0.03039555	0.19987499	0.15207283	8.79E-01	intergenic	ARRDC3-AS1;NR2F1-AS1	dist=758953;dist=1269577	rs190190051	0.0084	0.0151	0.982918	1
5:91530073:C	5	91530073	0.00445146	1	0.02835476	0.1864551	0.15207283	8.79E-01	intergenic	ARRDC3-AS1;NR2F1-AS1	dist=813541;dist=1214989	rs182531466	0.0079	0.0143	0.916629	1
3:141864350:	3	141864350	0.00941262	2	-0.04067729	0.27007401	-0.15061534	8.80E-01	intronic	TFDP2	.	rs545552231	0.0017	0.0029	0.904348	1
6:142269885:	6	142269885	0.01472816	3	-0.0499046	0.331489	-0.15054679	8.80E-01	intergenic	MIR4465;NMBR	dist=1264865;dist=126860	rs142106992	0.0068	0.0116	0.974922	1
1:229804538:	1	229804538	0.01941748	4	0.05619014	0.39697883	0.14154444	8.87E-01	intergenic	URB2;GALNT2	dist=8591;dist=388998	rs2274996	0.119	0.0431	1	1
1:229806368:	1	229806368	0.01941748	4	0.05619014	0.39697883	0.14154444	8.87E-01	intergenic	URB2;GALNT2	dist=10421;dist=387168	rs2891865	0.0843	0.0424	1	1
1:229824770:	1	229824770	0.01941748	4	0.05619014	0.39697883	0.14154444	8.87E-01	intergenic	URB2;GALNT2	dist=28823;dist=368766	rs4562666	0.1094	0.043	1	1
1:229831331:	1	229831331	0.01941748	4	0.05619014	0.39697883	0.14154444	8.87E-01	intergenic	URB2;GALNT2	dist=35384;dist=362205	rs16850124	0.1613	0.0447	1	1
21:23156546:	21	23156546	0.01758738	4	0.0449786	0.32176516	0.13978704	8.89E-01	ncRNA_intronic	LINC01425	.	rs75024143	0.007	0.0122	0.702913	1
2:52563371:C	2	52563371	0.00220874	0	-0.01305165	0.09516368	-0.1371495	8.91E-01	intergenic	NRXN1;MIR4431	dist=1303697;dist=366289	rs183378658	0.0017	0.0025	0.422723	1
1:229807492:	1	229807492	0.01933981	4	0.05291944	0.39489101	0.13401025	8.93E-01	intergenic	URB2;GALNT2	dist=11545;dist=386044	rs2385790	0.0661	0.0417	0.993425	1
2:52527354:T	2	52527354	0.00207767	0	-0.01200578	0.08995596	-0.13346282	8.94E-01	intergenic	NRXN1;MIR4431	dist=1267680;dist=402306	rs181193202	0.0018	0.0027	0.401439	1
1:229812357:	1	229812357	0.01934466	4	0.05261465	0.39487567	0.13324359	8.94E-01	intergenic	URB2;GALNT2	dist=16410;dist=381179	rs12024557	0.1093	0.0426	0.993171	1
1:229804646:	1	229804646	0.01936408	4	0.05234459	0.39487456	0.13256005	8.95E-01	intergenic	URB2;GALNT2	dist=8699;dist=388890	rs2274997	0.1186	0.043	0.992157	1
2:67565909:A	2	67565909	0.00786408	2	0.02790624	0.21524991	0.12964579	8.97E-01	intergenic	LOC102800447;ETAA1	dist=49533;dist=58533	rs11690187	0.0032	0.0047	0.837388	1
21:43276916:	21	43276916	0.00510194	1	-0.02342163	0.20678399	-0.11326615	9.10E-01	intronic	PRDM15	.	rs150539922	0.0026	0.0035	0.918546	1
2:53085778:G	2	53085778	0.00436408	1	-0.02145063	0.19447952	-0.11029764	9.12E-01	intergenic	MIR4431;ASB3	dist=156025;dist=811339	rs190193113	0.0016	0.0024	0.894549	1
1:103633635:	1	103633635	0.01638835	3	0.03086612	0.28612599	0.10787596	9.14E-01	intergenic	COL11A1;LOC101928436	dist=59583;dist=327066	rs111928960	0.015	0.0213	0.591355	1
22:40528090:	22	40528090	0.00556311	1	-0.01974371	0.19432826	-0.1015998	9.19E-01	intronic	TNRC6B	.	rs541680196	0.0007	0.0013	0.747052	1
1:103419168:	1	103419168	0.01709709	4	-0.02811392	0.29796789	-0.09435217	9.25E-01	intronic	COL11A1	.	rs114413507	0.0156	0.0224	0.530043	1
3:164285621:	3	164285621	0.00413592	1	0.01403123	0.17131386	0.08190364	9.35E-01	intergenic	MIR1263;LINC01324	dist=396277;dist=146262	rs187047882	0.0026	0.004	0.827625	1
3:42711221:A	3	42711221	0.01973786	4	-0.03198562	0.40445891	-0.0790825	9.37E-01	intergenic	ZBTB47;KLHL40	dist=2149;dist=15790	rs73085348	0.0132	0.0192	0.962032	1
22:40620530:	22	40620530	0.00540777	1	0.01375367	0.20531176	0.0669892	9.47E-01	intronic	TNRC6B	.	rs148998974	0.0007	0.0013	0.851831	1
7:146865072:	7	146865072	0.00787379	2	0.01517629	0.24014534	0.06319627	9.50E-01	intronic	CNTNAP2	.	rs536023430	0.0025	0.0031	0.812462	1
22:40604439:	22	40604439	0.00533495	1	0.01161262	0.20487662	0.05668105	9.55E-01	intronic	TNRC6B	.	rs141127122	0.0004	0.0006	0.859782	1
1:102961882:	1	102961882	0.01406311	3	0.01428562	0.25538907	0.05593671	9.55E-01	intergenic	OLFM3;COL11A1	dist=499092;dist=380141	rs77180278	0.0151	0.0218	0.587391	1
2:65875930:C	2	65875930	0.00468447	1	0.01016422	0.18533674	0.05484192	9.56E-01	intergenic	SPRED2;MIR4778	dist=216274;dist=709451	rs528288879	0.0043	0.0063	0.81586	1
22:40631476:	22	40631476	0.00598544	1	-0.00987839	0.20679901	-0.04776807	9.62E-01	intronic	TNRC6B	.	rs555040883	0.0003	0.0005	0.785825	1
1:103220360:	1	103220360	0.01623787	3	-0.013522	0.29931658	-0.04517624	9.64E-01	intergenic	OLFM3;COL11A1	dist=757570;dist=121663	rs112351653	0.0156	0.0224	0.659632	1
22:40594781:	22	40594781	0.00522816	1	0.00894748	0.19987571	0.04476521	9.64E-01	intronic	TNRC6B	.	rs185139807	0.0007	0.0013	0.834478	1
1:103472916:	1	103472916	0.01778641	4	0.01007671	0.31422553	0.03206841	9.74E-01	intronic	COL11A1	.	rs116672066	0.0151	0.0219	0.573939	1
6:85678832:A	6	85678832	0.00214078	0	0.00222023	0.08048978	0.02758402	9.78E-01	intergenic	TBX18;NT5E	dist=204878;dist=480470	rs571986619	0.0004	0.0005	0.356816	1
10:10291848(	10	102918486	0.02915534	6	0.01274117	0.49073343	0.02596352	9.79E-01	intergenic	TLX1NB;LINC01514	dist=17463;dist=17593	rs117913371	0.0141	0.0239	0.987133	1
9:135266715:	9	135266715	0.00491262	1	0.00344492	0.20028125	0.01720042	9.86E-01	intronic	TTF1	.	rs78296164	0.0047	0.0078	0.98038	1
1:229834050:	1	229834050	0.01748544	4	-0.00570229	0.36438542	-0.01564905	9.88E-01	intergenic	URB2;GALNT2	dist=38103;dist=359486	rs12045643	0.1113	0.0429	0.928351	1

Supplementary Table S7. Independent Replication
Florida-1 cohort, 12M quantitative trait (QT) data, comparing to discovery 12M QT

Threshold for replicative significance:	5.00E-02
Total SNPs found:	1
Total risk loci found:	1

NA: not applicable

rsid	chr	pos_37	REF	ALT	caf	MAC	Est	Est.SE	Score.pval	caf.sch	Est.sch	Est.SE.sch	pval.sch	adj pval SNPs	adj pval loci	Closest gene	Prioritized gene
rs9636964	21	41304765	A	G	0.884965831	115	-4.254319226	0.827443714	2.73E-07	0.06	2.37989208	2.5889182	3.58E-01	3.58E-01	3.58E-01	PCP4	PCP4
rs142934021	5	52662672	G	A	0.007972665	7	18.82471499	3.318202936	1.40E-08	NA	NA	NA	NA				
rs138164904	16	6858239	C	T	0.003416856	3	23.89264096	4.336966899	3.61E-08	NA	NA	NA	NA				
rs138138661	3	147657814	C	T	0.004555809	4	23.13474571	4.256958355	5.49E-08	NA	NA	NA	NA				
rs147669485	18	26603650	G	T	0.003416856	4	22.11076388	4.149231981	9.88E-08	NA	NA	NA	NA				
rs148997617	3	147755576	C	T	0.004555809	4	20.87742767	3.926489017	1.05E-07	NA	NA	NA	NA				
rs74572772	22	34492649	A	G	0.018223235	16	10.22251833	1.941267739	1.40E-07	NA	NA	NA	NA				
rs148157126	20	30948839	C	T	0.004555809	5	19.78650011	3.760421027	1.43E-07	NA	NA	NA	NA				
rs80019988	22	34493647	G	A	0.018223235	16	10.06233062	1.916654069	1.52E-07	NA	NA	NA	NA				
rs186792608	2	43096699	A	G	0.003416856	3	22.45124992	4.281728413	1.58E-07	NA	NA	NA	NA				
rs9974985	21	41307573	G	A	0.886104784	114	-4.345939521	0.829242821	1.60E-07	NA	NA	NA	NA				
rs113154814	2	68289497	T	C	0.005694761	5	19.83101879	3.789289395	1.66E-07	NA	NA	NA	NA				
rs1005412	21	41308948	A	G	0.887243736	112	-4.452109624	0.852371384	1.76E-07	NA	NA	NA	NA				
rs386818616	21	41308948	A	G	0.887243736	112	-4.452109624	0.852371384	1.76E-07	NA	NA	NA	NA				
rs193041547	20	30771880	T	C	0.004555809	4	19.67913585	3.770811462	1.80E-07	NA	NA	NA	NA				
rs77297738	22	34473543	C	T	0.018223235	16	10.24805718	1.971675922	2.02E-07	NA	NA	NA	NA				
rs146249289	20	30682231	C	T	0.004555809	4	19.6109527	3.773060354	2.02E-07	NA	NA	NA	NA				
rs76356799	3	179593768	G	A	0.004555809	4	19.28670071	3.760393191	2.91E-07	NA	NA	NA	NA				
rs7275595	21	41307923	G	A	0.888382688	113	-4.260975379	0.834078676	3.25E-07	NA	NA	NA	NA				
rs192855100	20	31108741	G	A	0.004555809	4	20.19860967	3.99111726	4.17E-07	NA	NA	NA	NA				
rs2427460	20	61590782	T	C	0.486332574	420	-2.574043965	0.513258918	5.30E-07	NA	NA	NA	NA				
rs562831582	19	51234310	A	C	0.003416856	4	21.08565484	4.216248377	5.70E-07	NA	NA	NA	NA				
rs149859280	20	30678170	C	T	0.004555809	5	18.30577068	3.660758895	5.72E-07	NA	NA	NA	NA				
rs138733283	20	30675687	C	T	0.004555809	5	18.27447439	3.658301378	5.87E-07	NA	NA	NA	NA				
rs80212581	16	6412967	C	T	0.003416856	3	22.27620028	4.472373329	6.33E-07	NA	NA	NA	NA				
rs148248743	3	136134595	C	T	0.002277904	3	27.15485873	5.463527255	6.69E-07	NA	NA	NA	NA				
rs188353596	17	46078602	C	T	0.003416856	3	21.80579474	4.391419406	6.85E-07	NA	NA	NA	NA				
rs113791989	13	61918888	G	A	0.002277904	3	25.20495822	5.084027893	7.13E-07	NA	NA	NA	NA				
rs56324718	15	40280073	G	A	0.010250569	9	12.5117374	2.5288888	7.52E-07	NA	NA	NA	NA				
rs191298981	2	68110046	C	T	0.004555809	3	24.00787141	4.857032236	7.70E-07	NA	NA	NA	NA				
rs146919974	2	115252863	T	C	0.004555809	5	19.72804656	3.995542316	7.91E-07	NA	NA	NA	NA				
rs9305683	21	41305720	G	A	0.88952164	112	-4.116367918	0.836343627	8.57E-07	NA	NA	NA	NA				
rs145791959	20	30722429	G	A	0.005694761	5	17.90919529	3.640341121	8.67E-07	NA	NA	NA	NA				
rs142956968	10	23490165	C	T	0.01594533	14	10.41679921	2.119090829	8.85E-07	NA	NA	NA	NA				
rs532269430	13	74054452	T	C	0.004555809	5	18.61737516	3.796300403	9.39E-07	NA	NA	NA	NA				
rs113063005	4	23509067	T	C	0.006833713	7	16.61762999	3.391132089	9.57E-07	NA	NA	NA	NA				
rs147944608	10	110423709	C	T	0.012528474	11	11.22052434	2.292291613	9.84E-07	NA	NA	NA	NA				
rs2606194	17	77210823	A	G	0.948747153	44	-5.695697885	1.164357735	1.00E-06	NA	NA	NA	NA				
rs113537164	2	68234930	A	C	0.004555809	4	20.38904135	4.17097432	1.02E-06	NA	NA	NA	NA				
rs138055631	20	30780644	G	A	0.005694761	5	17.66477622	3.620049149	1.06E-06	NA	NA	NA	NA				
rs566724618	13	23463839	C	T	0.003416856	3	22.34337048	4.580616053	1.07E-06	NA	NA	NA	NA				
rs145764464	10	23601847	G	A	0.017084282	15	9.596730604	1.969763479	1.10E-06	NA	NA	NA	NA				
rs9981433	21	41309565	G	T	0.881548975	116	-4.122012439	0.84715422	1.14E-06	NA	NA	NA	NA				
rs139816293	20	30921343	C	T	0.005694761	6	17.13842475	3.525372234	1.17E-06	NA	NA	NA	NA				
rs117699122	12	560262	T	C	0.003416856	4	21.42382563	4.40952328	1.18E-06	NA	NA	NA	NA				
rs529110230	7	117175907	T	C	0.003416856	3	20.85836332	4.302350877	1.25E-06	NA	NA	NA	NA				
rs12114488	8	62672723	G	A	0.30523918	266	2.623079144	0.54257702	1.33E-06	NA	NA	NA	NA				
rs182868205	3	177712448	C	T	0.003416856	3	22.85443031	4.732156621	1.37E-06	NA	NA	NA	NA				

rs144138711	9	99922419	T	C	0.045558087	40	6.085471305	1.260919179	1.39E-06	NA	NA	NA	NA
rs372194899	13	74357510	A	G	0.003416856	3	21.30121009	4.41516106	1.40E-06	NA	NA	NA	NA
rs368187808	13	74357508	A	C	0.003416856	3	21.2991251	4.415016369	1.41E-06	NA	NA	NA	NA
rs145804766	1	202122499	C	T	0.010250569	9	12.58644653	2.611189618	1.43E-06	NA	NA	NA	NA
rs182211730	7	116003615	G	A	0.004555809	4	18.7746033	3.897198104	1.45E-06	NA	NA	NA	NA
rs558784715	7	117043294	A	G	0.003416856	3	20.75917871	4.316894751	1.52E-06	NA	NA	NA	NA
rs189709453	3	177574308	G	A	0.003416856	3	23.20099361	4.829543736	1.56E-06	NA	NA	NA	NA
rs142721557	7	117218328	A	C	0.003416856	3	20.73299749	4.322458941	1.61E-06	NA	NA	NA	NA
rs576047962	7	117109021	A	G	0.003416856	3	20.72615487	4.321401824	1.62E-06	NA	NA	NA	NA
rs142215699	7	117199874	T	C	0.003416856	3	20.72922868	4.322453614	1.62E-06	NA	NA	NA	NA
rs143281973	7	116007996	C	T	0.004555809	4	18.27507784	3.810981978	1.62E-06	NA	NA	NA	NA
rs17127656	1	65943471	C	T	0.055808656	49	5.070465376	1.057806414	1.64E-06	NA	NA	NA	NA
rs201355675	7	117225781	G	A	0.003416856	3	20.71812396	4.322467219	1.64E-06	NA	NA	NA	NA
rs186767531	3	177639777	T	C	0.003416856	3	23.07499768	4.81664642	1.66E-06	NA	NA	NA	NA
rs7518849	1	65948791	T	C	0.055808656	49	5.074569555	1.059644692	1.68E-06	NA	NA	NA	NA
rs181132315	4	129647369	C	T	0.003416856	3	22.13246791	4.62266164	1.69E-06	NA	NA	NA	NA
rs75082290	2	67831153	T	G	0.078587699	69	4.693953865	0.98072733	1.70E-06	NA	NA	NA	NA
rs145421321	20	30862126	C	T	0.005694761	6	17.02126111	3.558296484	1.72E-06	NA	NA	NA	NA
rs143432612	20	30892775	C	T	0.005694761	6	16.93872319	3.543181539	1.75E-06	NA	NA	NA	NA
rs140276610	16	6433735	C	T	0.003416856	4	22.18065773	4.640624008	1.76E-06	NA	NA	NA	NA
rs559152067	18	66029612	G	A	0.002277904	3	24.74436915	5.183438622	1.81E-06	NA	NA	NA	NA
rs147221953	13	23485722	G	A	0.003416856	3	22.58096755	4.736033596	1.86E-06	NA	NA	NA	NA
rs556274646	11	439012	C	T	0.010250569	9	12.89145917	2.708477277	1.94E-06	NA	NA	NA	NA
rs190251199	14	105590577	T	C	0.003416856	3	22.63850613	4.760438165	1.98E-06	NA	NA	NA	NA
rs188993522	7	117274731	T	C	0.003416856	3	21.29848267	4.478672657	1.98E-06	NA	NA	NA	NA
rs568321148	2	29870348	T	G	0.003416856	3	20.74002026	4.366527169	2.04E-06	NA	NA	NA	NA
rs148815783	11	1013992	C	T	0.007972665	8	13.59803132	2.864717097	2.07E-06	NA	NA	NA	NA
rs76220567	2	13059945	T	C	0.003416856	4	20.68288684	4.357762163	2.07E-06	NA	NA	NA	NA
rs35145334	1	23465122	A	G	0.017084282	14	10.65526559	2.246207918	2.10E-06	NA	NA	NA	NA
rs11579567	1	65957141	C	A	0.055808656	49	5.029737931	1.060973792	2.13E-06	NA	NA	NA	NA
rs114067899	1	85191044	G	A	0.007972665	7	13.77606734	2.908363649	2.17E-06	NA	NA	NA	NA
rs56821264	6	148703901	C	T	0.006833713	6	14.75092126	3.114195656	2.17E-06	NA	NA	NA	NA
rs917335559	6	148703901	C	T	0.006833713	6	14.75092126	3.114195656	2.17E-06	NA	NA	NA	NA
rs536781978	2	29733681	A	G	0.003416856	3	20.55618824	4.342580992	2.21E-06	NA	NA	NA	NA
rs147267707	4	43026558	G	A	0.004555809	4	18.00943854	3.808184082	2.25E-06	NA	NA	NA	NA
rs77618729	4	43050968	T	C	0.004555809	4	18.01301525	3.809141731	2.26E-06	NA	NA	NA	NA
rs547186621	20	6106741	A	G	0.003416856	4	21.55108289	4.569088099	2.40E-06	NA	NA	NA	NA
rs200198574	20	30946654	G	A	0.005694761	6	16.52827354	3.50702674	2.44E-06	NA	NA	NA	NA
rs532464521	4	43571039	T	C	0.004555809	4	17.98955275	3.818204376	2.46E-06	NA	NA	NA	NA
rs79252854	3	157523157	C	T	0.004555809	5	17.54643357	3.72541417	2.48E-06	NA	NA	NA	NA
rs79935606	2	177338635	T	C	0.005694761	5	16.33968329	3.473769742	2.55E-06	NA	NA	NA	NA
rs556435089	4	43551033	G	A	0.004555809	4	17.93907904	3.817721456	2.62E-06	NA	NA	NA	NA
rs62192733	20	1928157	A	G	0.006833713	7	15.74461797	3.358665632	2.76E-06	NA	NA	NA	NA
rs7534177	1	65965720	A	G	0.056947608	49	4.961233778	1.059861601	2.85E-06	NA	NA	NA	NA
rs139390630	1	84866196	G	A	0.011389522	13	11.69759713	2.499486296	2.87E-06	NA	NA	NA	NA
rs149706477	3	66148228	A	G	0.003416856	3	20.98286869	4.484253102	2.88E-06	NA	NA	NA	NA
rs189094663	4	11623450	G	A	0.002277904	3	23.895416	5.108227082	2.90E-06	NA	NA	NA	NA
rs181182636	7	115654558	A	G	0.003416856	3	21.94726388	4.692447434	2.91E-06	NA	NA	NA	NA
rs76098744	1	112349372	C	T	0.01594533	14	9.568565696	2.046351926	2.93E-06	NA	NA	NA	NA
rs4896997	6	148683924	C	T	0.006833713	6	14.43209229	3.091883755	3.05E-06	NA	NA	NA	NA
rs17078283	6	148696920	C	T	0.006833713	6	14.43028205	3.091804795	3.05E-06	NA	NA	NA	NA
rs73202425	7	109155716	T	C	0.012528474	12	10.76184396	2.305821662	3.05E-06	NA	NA	NA	NA
rs4131286	6	148688963	G	T	0.006833713	6	14.42846716	3.091725323	3.06E-06	NA	NA	NA	NA
rs74683551	1	112354418	G	A	0.01594533	14	9.547228317	2.046406793	3.08E-06	NA	NA	NA	NA
rs139055031	3	158586849	T	C	0.005694761	6	16.28537606	3.4955337	3.18E-06	NA	NA	NA	NA
rs138873576	7	116025359	G	T	0.004555809	4	17.61517106	3.78331079	3.22E-06	NA	NA	NA	NA
rs148483098	3	157382051	C	A	0.005694761	5	15.73578443	3.382110237	3.28E-06	NA	NA	NA	NA
rs187281112	15	33113387	C	T	0.005694761	5	14.73433563	3.167936491	3.30E-06	NA	NA	NA	NA

rs1162141034	2	67825685	C	T	0.079726651	71	4.559914114	0.980447215	3.31E-06	NA	NA	NA	NA
rs146465493	2	67825685	C	T	0.079726651	71	4.559914114	0.980447215	3.31E-06	NA	NA	NA	NA
rs2902021	2	67825685	C	T	0.079726651	71	4.559914114	0.980447215	3.31E-06	NA	NA	NA	NA
rs189912648	5	134765439	C	T	0.003416856	4	19.8604449	4.271665199	3.33E-06	NA	NA	NA	NA
rs557276277	4	19013698	T	C	0.005694761	5	19.01463443	4.091376275	3.36E-06	NA	NA	NA	NA
rs147869916	1	227307652	A	G	0.005694761	5	17.59645507	3.78625881	3.36E-06	NA	NA	NA	NA
rs367732718	19	35918004	G	A	0.002277904	3	23.87849231	5.139957519	3.39E-06	NA	NA	NA	NA
rs62515436	8	57141203	G	T	0.017084282	15	9.275459023	1.998048118	3.45E-06	NA	NA	NA	NA
rs189234695	3	147658158	T	C	0.003416856	3	23.96971341	5.165421292	3.48E-06	NA	NA	NA	NA
rs186893139	7	116096970	C	T	0.004555809	4	18.60259803	4.012583835	3.55E-06	NA	NA	NA	NA
rs73384619	6	20458179	C	T	0.022779043	21	8.391670204	1.812413894	3.65E-06	NA	NA	NA	NA
rs114280794	3	132842203	G	A	0.01594533	14	8.762968775	1.896320161	3.82E-06	NA	NA	NA	NA
rs112148840	17	66233774	C	T	0.002277904	3	24.02410753	5.199447601	3.83E-06	NA	NA	NA	NA
rs147455971	4	140931712	T	C	0.007972665	5	17.21601424	3.727930571	3.87E-06	NA	NA	NA	NA
rs144788248	3	19586745	T	C	0.011389522	10	11.90862374	2.578791237	3.88E-06	NA	NA	NA	NA
rs111512950	5	153680427	C	T	0.009111617	8	13.56744305	2.945984928	4.12E-06	NA	NA	NA	NA
rs149616342	12	99178813	C	T	0.011389522	10	11.2978214	2.454656997	4.17E-06	NA	NA	NA	NA
rs17543620	4	169934725	T	C	0.050113895	44	5.427967555	1.179820972	4.21E-06	NA	NA	NA	NA
rs190354334	7	116075846	G	A	0.004555809	5	17.33925809	3.7718663	4.29E-06	NA	NA	NA	NA
rs17615362	4	169934087	G	A	0.050113895	44	5.419744721	1.179184251	4.30E-06	NA	NA	NA	NA
rs117591241	3	190584679	A	G	0.003416856	4	19.90140277	4.333313893	4.38E-06	NA	NA	NA	NA
rs1472243465	3	190584679	A	G	0.003416856	4	19.90140277	4.333313893	4.38E-06	NA	NA	NA	NA
rs61918041	12	6681868	C	T	0.023917995	21	7.972646426	1.736360556	4.40E-06	NA	NA	NA	NA
rs113244573	12	6684385	G	A	0.023917995	21	7.970957457	1.737661556	4.49E-06	NA	NA	NA	NA
rs62515405	8	57055978	G	A	0.023917995	21	8.076912036	1.76428755	4.69E-06	NA	NA	NA	NA
rs116897913	17	16839162	C	T	0.003416856	3	20.13967647	4.402942883	4.78E-06	NA	NA	NA	NA
rs143686474	7	16291999	A	C	0.012528474	11	10.85314984	2.375131845	4.89E-06	NA	NA	NA	NA
rs143669489	3	159392885	G	A	0.003416856	3	24.22531986	5.304006744	4.94E-06	NA	NA	NA	NA

Supplementary Table S7. Independent Replication

Florida-1 cohort, 12M quantitative trait (QT) data, comparing to discovery 3M QT

Threshold for replicative significance = 5.00E-02<br>Total SNPs found: 7<br>Total risk loci found: 4	NA: not applicable

MAF ALPHA	rsid	chr	pos_37	REF	ALT	caf	MAC	Est	Est.SE	Score.pval	caf.sch	Est.sch	Est.SE.sch	pval.sch	adj pval SNPs	adj pval loci	Closest gene	Prioritized gene
0.345139	rs2375537	8	55619508	C	T	0.332541568	280	2.23876848	0.44938849	6.30E-07	0.37	1.57403151	1.34444327	2.42E-01	1.00E+00	9.67E-01	RP1	SOX17
0.042426	rs2327968	20	15813491	C	T	0.024940618	21	6.15899856	1.34390809	4.59E-06	0.05	3.76518574	2.97243008	2.05E-01	1.00E+00	8.21E-01	MACROD2	MACROD2
0.036625	rs2876414	20	15813704	G	T	0.022565321	19	6.81910907	1.48530907	4.41E-06	0.05	3.76518574	2.97243008	2.05E-01	1.00E+00	8.21E-01	MACROD2	MACROD2
0.365421	rs423841	8	55556069	G	A	0.662707838	291	-2.1495726	0.45846846	2.75E-06	0.62	-1.324842	1.28913487	3.04E-01	1.00E+00	1.00E+00	RP1	SOX17
0.096148	rs2274997	1	229804646	A	G	0.041567696	35	5.58731228	1.1238603	6.64E-07	0.05	2.30579163	3.86926843	5.51E-01	1.00E+00	1.00E+00	URB2	URB2
0.283534	rs9643828	8	55529073	C	T	0.67695962	279	-2.4236294	0.46846911	2.30E-07	0.69	-0.6795104	1.3833319	6.23E-01	1.00E+00	1.00E+00	RP1	SOX17
0.097988	rs12315614	12	64920957	C	A	0.076009501	64	3.77710662	0.8201959	4.12E-06	0.08	-0.1411232	2.16890982	9.48E-01	1.00E+00	1.00E+00	TBK1	TBK1
	rs113063005	4	23509067	T	C	0.005938242	6	21.2073894	3.15520096	1.80E-11	NA	NA	NA	NA				
	rs142106992	6	142269885	C	A	0.003562945	4	22.8345886	3.6758699	5.23E-10	NA	NA	NA	NA				
	rs181217257	2	239989119	C	T	0.004750594	4	21.5677368	3.47296989	5.29E-10	NA	NA	NA	NA				
	rs188076929	2	239993719	T	C	0.004750594	4	19.9775614	3.27246746	1.03E-09	NA	NA	NA	NA				
	rs117998251	10	13497976	C	T	0.003562945	3	22.9543311	3.77480488	1.19E-09	NA	NA	NA	NA				
	rs184458518	10	13471195	T	G	0.003562945	3	22.8015789	3.77123942	1.48E-09	NA	NA	NA	NA				
	rs184425183	10	13457520	A	G	0.003562945	3	22.6683174	3.77549648	1.92E-09	NA	NA	NA	NA				
	rs148153037	6	167501386	G	A	0.008313539	8	14.3359246	2.42128102	3.20E-09	NA	NA	NA	NA				
	rs111285015	19	23123198	G	A	0.003562945	3	27.3347642	4.62083983	3.31E-09	NA	NA	NA	NA				
	rs111928960	1	103633635	G	A	0.028503563	22	8.60257208	1.45827516	3.65E-09	NA	NA	NA	NA				
	rs187520610	2	53360041	G	A	0.004750594	4	18.8010396	3.19364595	3.93E-09	NA	NA	NA	NA				
	rs545428520	5	167821266	T	C	0.003562945	4	21.566035	3.66407988	3.96E-09	NA	NA	NA	NA				
	rs116672066	1	103472916	G	A	0.027315914	23	8.04769476	1.37187844	4.46E-09	NA	NA	NA	NA				
	rs192134381	21	23450714	T	C	0.003562945	3	21.788812	3.73125357	5.23E-09	NA	NA	NA	NA				
	rs397836601	21	23450714	T	C	0.003562945	3	21.788812	3.73125357	5.23E-09	NA	NA	NA	NA				
	rs150586237	6	24491348	C	T	0.003562945	3	22.0461443	3.78094793	5.51E-09	NA	NA	NA	NA				
	rs528404963	5	167852025	T	C	0.003562945	4	21.1393977	3.65303517	7.17E-09	NA	NA	NA	NA				
	rs76526501	7	18431110	G	A	0.007125891	6	14.8397312	2.5778114	8.58E-09	NA	NA	NA	NA				
	rs74455595	7	18431784	A	G	0.007125891	6	14.8397312	2.5778114	8.58E-09	NA	NA	NA	NA				
	rs75090694	7	18447436	A	G	0.008313539	7	13.8314005	2.41427081	1.01E-08	NA	NA	NA	NA				
	rs575473987	8	5577494	C	T	0.003562945	3	22.2248653	3.88625754	1.07E-08	NA	NA	NA	NA				
	rs545690161	9	93030699	G	A	0.005938242	6	17.1436874	3.00318779	1.14E-08	NA	NA	NA	NA				
	rs77141817	4	37053759	T	C	0.003562945	3	20.6110292	3.64214866	1.52E-08	NA	NA	NA	NA				
	rs190822761	4	37099356	G	T	0.003562945	3	20.5846354	3.64346559	1.61E-08	NA	NA	NA	NA				
	rs10279777	7	18441589	G	A	0.009501188	8	12.8681335	2.27961694	1.65E-08	NA	NA	NA	NA				
	rs10486295	7	18446807	G	A	0.009501188	8	12.76229	2.26480456	1.75E-08	NA	NA	NA	NA				
	rs147559909	2	237051523	T	C	0.005938242	5	17.0840975	3.03209195	1.76E-08	NA	NA	NA	NA				
	rs17169602	7	18446741	G	A	0.009501188	8	12.7273717	2.25993984	1.78E-08	NA	NA	NA	NA				
	rs139598422	13	23887014	A	G	0.003562945	4	20.4886522	3.64167194	1.84E-08	NA	NA	NA	NA				
	rs74704551	14	30161887	C	T	0.003562945	3	24.1990705	4.31064693	1.98E-08	NA	NA	NA	NA				
	rs77300464	7	18408761	A	G	0.007125891	6	14.6659794	2.6169516	2.09E-08	NA	NA	NA	NA				
	rs75606013	7	18414613	G	A	0.007125891	6	14.6617119	2.61692421	2.11E-08	NA	NA	NA	NA				
	rs541653703	11	18701786	G	A	0.004750594	4	18.3169499	3.27243418	2.18E-08	NA	NA	NA	NA				
	rs117913371	10	102918486	G	A	0.024940618	21	7.86609555	1.40785512	2.31E-08	NA	NA	NA	NA				
	rs191053292	12	63445280	T	C	0.003562945	3	22.9266488	4.11439732	2.51E-08	NA	NA	NA	NA				
	rs112351653	1	103220360	T	C	0.028503563	24	7.42386947	1.33286772	2.55E-08	NA	NA	NA	NA				
	rs114413507	1	103419168	T	C	0.028503563	24	7.42216029	1.33294954	2.57E-08	NA	NA	NA	NA				
	rs138414342	11	18679398	G	A	0.004750594	5	18.1582505	3.28214943	3.16E-08	NA	NA	NA	NA				
	rs16823323	3	153657202	G	A	0.016627078	14	9.48464065	1.71769795	3.36E-08	NA	NA	NA	NA				
	rs183737367	9	93330047	T	C	0.003562945	3	23.3452306	4.24471431	3.80E-08	NA	NA	NA	NA				
	rs560206697	19	20729098	C	T	0.002375297	3	24.9989943	4.55350673	4.02E-08	NA	NA	NA	NA				
	rs79486609	21	17245006	G	A	0.003562945	3	20.6624886	3.77824957	4.53E-08	NA	NA	NA	NA				

rs151115079	11	18655741	T	C	0.004750594	5	17.8273587	3.2680205	4.89E-08	NA	NA	NA	NA
rs185510569	2	223814861	G	A	0.003562945	3	19.5886906	3.60490977	5.51E-08	NA	NA	NA	NA
rs187213609	9	93415465	C	T	0.003562945	3	23.1975304	4.27412656	5.72E-08	NA	NA	NA	NA
rs117280553	21	17207163	T	C	0.003562945	3	20.087417	3.70274826	5.80E-08	NA	NA	NA	NA
rs183816745	5	91631888	A	G	0.005938242	5	17.3487909	3.20195556	6.02E-08	NA	NA	NA	NA
rs180828621	10	124533409	G	A	0.007125891	6	15.1380574	2.79957627	6.40E-08	NA	NA	NA	NA
rs142684595	3	55319301	T	C	0.005938242	5	16.7729431	3.11364755	7.17E-08	NA	NA	NA	NA
rs184785969	21	17171431	C	A	0.003562945	3	19.8736486	3.70683937	8.26E-08	NA	NA	NA	NA
rs77180278	1	102961882	T	C	0.028503563	24	7.33619996	1.37404112	9.34E-08	NA	NA	NA	NA
rs189709453	3	177574308	G	A	0.003562945	3	21.6581488	4.05916142	9.52E-08	NA	NA	NA	NA
rs111846247	5	95111643	T	C	0.003562945	3	20.4865772	3.85467205	1.07E-07	NA	NA	NA	NA
rs145875128	4	32073136	G	A	0.003562945	3	24.479986	4.60639213	1.07E-07	NA	NA	NA	NA
rs75848314	5	95098340	T	C	0.003562945	3	20.0998481	3.78700451	1.11E-07	NA	NA	NA	NA
rs190193113	2	53085778	G	A	0.003562945	3	19.9533602	3.7619559	1.13E-07	NA	NA	NA	NA
rs183378658	2	52563371	C	T	0.003562945	3	19.7446169	3.72634921	1.17E-07	NA	NA	NA	NA
rs143371352	13	47383834	C	T	0.003562945	3	20.5341625	3.87928964	1.20E-07	NA	NA	NA	NA
rs75186966	13	47395758	A	C	0.003562945	3	20.2281345	3.83225523	1.30E-07	NA	NA	NA	NA
rs532416695	2	177486790	G	A	0.003562945	3	19.8726656	3.76737683	1.33E-07	NA	NA	NA	NA
rs145080832	2	48473143	G	A	0.004750594	4	17.1618477	3.25709537	1.37E-07	NA	NA	NA	NA
rs182868205	3	177712448	C	T	0.003562945	3	20.9548768	3.97841839	1.39E-07	NA	NA	NA	NA
rs143538552	18	29050262	A	G	0.003562945	3	19.1457218	3.63679703	1.41E-07	NA	NA	NA	NA
rs1221830047	8	55690220	T	C	0.330166271	277	2.3570878	0.44879289	1.50E-07	NA	NA	NA	NA
rs7822082	8	55690220	T	C	0.330166271	277	2.3570878	0.44879289	1.50E-07	NA	NA	NA	NA
rs72986533	6	142611258	T	C	0.008313539	8	13.2812726	2.53063629	1.54E-07	NA	NA	NA	NA
rs373746073	18	29058384	C	A	0.003562945	3	19.0847197	3.63798064	1.55E-07	NA	NA	NA	NA
rs181193202	2	52527354	T	C	0.003562945	3	19.6312088	3.74776015	1.62E-07	NA	NA	NA	NA
rs186767531	3	177639777	T	C	0.003562945	3	21.2013243	4.04780758	1.63E-07	NA	NA	NA	NA
rs140797780	8	22087792	C	T	0.003562945	3	19.2908396	3.69147785	1.73E-07	NA	NA	NA	NA
rs137880949	12	63306297	T	C	0.004750594	3	21.6084034	4.13589168	1.75E-07	NA	NA	NA	NA
rs148532212	6	165499857	T	C	0.003562945	3	19.3535457	3.70945693	1.81E-07	NA	NA	NA	NA
rs141754456	12	20151132	T	C	0.007125891	7	12.1460333	2.32842921	1.82E-07	NA	NA	NA	NA
rs117498042	6	165514281	C	T	0.003562945	3	19.335471	3.70913521	1.86E-07	NA	NA	NA	NA
rs184487573	6	167513471	A	G	0.007125891	7	13.5009635	2.59268014	1.92E-07	NA	NA	NA	NA
rs149298750	4	127158701	A	C	0.003562945	3	22.184052	4.26573246	1.99E-07	NA	NA	NA	NA
rs140277951	10	82359100	G	A	0.008313539	6	13.7463158	2.64333932	1.99E-07	NA	NA	NA	NA
rs558553658	2	15682724	C	T	0.004750594	3	17.1099107	3.29108061	2.01E-07	NA	NA	NA	NA
rs112679237	4	139140860	T	C	0.017814727	18	8.71202752	1.67680671	2.04E-07	NA	NA	NA	NA
rs116862847	14	64141677	C	T	0.007125891	7	15.3160369	2.95194262	2.12E-07	NA	NA	NA	NA
rs528809914	13	113041256	G	A	0.003562945	3	19.5048552	3.76867111	2.27E-07	NA	NA	NA	NA
rs112007361	11	99188380	A	C	0.03087886	26	6.35658468	1.22825758	2.28E-07	NA	NA	NA	NA
rs546144116	19	19563339	C	T	0.002375297	3	23.1354815	4.47162758	2.29E-07	NA	NA	NA	NA
rs17510814	12	28468969	A	C	0.007125891	6	14.0387924	2.71409689	2.31E-07	NA	NA	NA	NA
rs113167689	12	28435962	C	T	0.007125891	6	14.1364135	2.73335629	2.32E-07	NA	NA	NA	NA
rs140788628	20	15858501	C	A	0.010688836	8	11.8039284	2.2826396	2.33E-07	NA	NA	NA	NA
rs141756120	12	28511096	A	C	0.008313539	6	14.278403	2.76458538	2.41E-07	NA	NA	NA	NA
rs117991215	12	28511473	T	C	0.008313539	6	14.333666	2.77918736	2.50E-07	NA	NA	NA	NA
rs191423619	4	126516627	G	T	0.002375297	3	21.694611	4.20667337	2.51E-07	NA	NA	NA	NA
rs113651406	4	990967	C	T	0.003562945	3	19.4054026	3.7635826	2.52E-07	NA	NA	NA	NA
rs187942235	18	27030430	C	T	0.003562945	3	20.3968658	3.96963025	2.77E-07	NA	NA	NA	NA
rs16920698	8	55678434	G	A	0.330166271	277	2.30492695	0.44908177	2.86E-07	NA	NA	NA	NA
rs4737676	8	55679546	G	A	0.330166271	277	2.30492057	0.44908192	2.86E-07	NA	NA	NA	NA
rs4737674	8	55661654	C	A	0.330166271	277	2.30559554	0.44921988	2.86E-07	NA	NA	NA	NA
rs13277510	8	55674149	G	A	0.330166271	277	2.30494752	0.44909942	2.86E-07	NA	NA	NA	NA
rs983248	8	55680792	C	T	0.330166271	277	2.30260428	0.44871461	2.87E-07	NA	NA	NA	NA
rs1391463	8	55681876	T	G	0.330166271	277	2.30258865	0.44871307	2.87E-07	NA	NA	NA	NA
rs4737201	8	55691458	C	T	0.330166271	277	2.30197734	0.44869179	2.89E-07	NA	NA	NA	NA
rs549931083	12	20516286	A	C	0.003562945	3	14.3282694	2.79891395	3.07E-07	NA	NA	NA	NA
rs141281289	11	123693024	A	G	0.005938242	6	15.811518	3.0889522	3.08E-07	NA	NA	NA	NA

rs184220112	2	48631743	C	A	0.004750594	4	16.4879043	3.22267606	3.12E-07	NA	NA	NA	NA
rs11987234	8	55669829	A	G	0.328978622	276	2.29599259	0.44884532	3.13E-07	NA	NA	NA	NA
rs13276543	8	55688174	G	T	0.328978622	276	2.29263157	0.44823093	3.14E-07	NA	NA	NA	NA
rs111838310	2	74673491	C	A	0.004750594	5	17.7963938	3.48429066	3.26E-07	NA	NA	NA	NA
rs80156375	7	18443215	A	C	0.009501188	8	11.5406255	2.25971308	3.27E-07	NA	NA	NA	NA
rs1561297	8	55678538	A	C	0.332541568	279	2.28315237	0.44743146	3.35E-07	NA	NA	NA	NA
rs112983626	2	74697150	G	A	0.004750594	4	17.7848079	3.48532494	3.35E-07	NA	NA	NA	NA
rs12548593	8	55674617	G	T	0.332541568	279	2.28269961	0.44752402	3.38E-07	NA	NA	NA	NA
rs10105693	8	55640472	C	T	0.328978622	276	2.2928391	0.44965282	3.41E-07	NA	NA	NA	NA
rs13278605	8	55688171	C	T	0.328978622	277	2.27377348	0.44593165	3.42E-07	NA	NA	NA	NA
rs2083123	8	55680318	C	T	0.332541568	279	2.27910794	0.44707491	3.44E-07	NA	NA	NA	NA
rs17017794	4	91825885	T	C	0.039192399	33	5.83240545	1.14450838	3.47E-07	NA	NA	NA	NA
rs74521112	11	99089147	G	T	0.032066508	27	6.25632387	1.22787586	3.48E-07	NA	NA	NA	NA
rs116651654	4	163238743	C	T	0.007125891	6	15.6223694	3.06624451	3.49E-07	NA	NA	NA	NA
rs565682685	9	93222328	T	C	0.004750594	4	18.938397	3.7206174	3.58E-07	NA	NA	NA	NA
rs144541665	1	104310729	G	A	0.002375297	3	21.8734611	4.30339839	3.72E-07	NA	NA	NA	NA
rs193253461	15	59229353	A	G	0.013064133	12	10.5374468	2.07404763	3.76E-07	NA	NA	NA	NA
rs77867199	7	18442275	G	T	0.010688836	9	10.8221282	2.13363365	3.93E-07	NA	NA	NA	NA
rs189360484	12	1870510	A	G	0.004750594	4	16.8078794	3.31424855	3.95E-07	NA	NA	NA	NA
rs79182806	7	18433827	T	C	0.008313539	7	12.0972603	2.38956889	4.14E-07	NA	NA	NA	NA
rs189890455	2	48655397	C	T	0.005938242	5	15.1467391	2.99355083	4.20E-07	NA	NA	NA	NA
rs190294315	9	85945465	C	T	0.005938242	5	14.5664276	2.88126649	4.29E-07	NA	NA	NA	NA
rs185819304	18	27001580	G	A	0.003562945	3	19.9401113	3.94501953	4.32E-07	NA	NA	NA	NA
rs148433854	19	31096478	G	A	0.003562945	3	18.6267585	3.68806754	4.41E-07	NA	NA	NA	NA
rs576124203	3	141841196	T	G	0.003562945	3	19.4039925	3.84392582	4.47E-07	NA	NA	NA	NA
rs143597860	1	104157143	A	G	0.002375297	3	21.5748154	4.2760379	4.52E-07	NA	NA	NA	NA
rs140782222	9	84028894	T	C	0.002375297	3	21.791526	4.31928461	4.53E-07	NA	NA	NA	NA
rs1856085	1	104114545	G	A	0.002375297	3	21.51144	4.26747898	4.64E-07	NA	NA	NA	NA
rs186768950	19	18806124	C	A	0.002375297	3	22.7152061	4.50776479	4.68E-07	NA	NA	NA	NA
rs182303755	15	59634792	A	C	0.013064133	12	10.5479917	2.09359291	4.70E-07	NA	NA	NA	NA
rs545552231	3	141864350	C	T	0.003562945	3	19.8641332	3.94582563	4.80E-07	NA	NA	NA	NA
rs78547898	22	32824278	G	A	0.003562945	3	20.9098395	4.15538274	4.85E-07	NA	NA	NA	NA
rs796777817	22	32824278	G	A	0.003562945	3	20.9098395	4.15538274	4.85E-07	NA	NA	NA	NA
rs78907958	7	18425017	T	G	0.008313539	7	12.0901322	2.40514961	4.99E-07	NA	NA	NA	NA
rs113751774	4	23428854	C	T	0.007125891	7	14.9103274	2.96657459	5.01E-07	NA	NA	NA	NA
rs10958428	8	55685641	A	G	0.333729216	280	2.23996885	0.44568673	5.01E-07	NA	NA	NA	NA
rs17776100	7	6426479	G	A	0.029691211	25	6.63697397	1.32177185	5.13E-07	NA	NA	NA	NA
rs185464792	19	18797371	C	T	0.002375297	3	22.5896015	4.50191473	5.23E-07	NA	NA	NA	NA
rs117025967	10	20207052	C	A	0.013064133	11	9.66973563	1.927439	5.25E-07	NA	NA	NA	NA
rs188028357	7	100668425	C	T	0.002375297	3	21.8425578	4.35791809	5.38E-07	NA	NA	NA	NA
rs148556485	9	84023826	A	C	0.002375297	3	21.3800152	4.26618316	5.40E-07	NA	NA	NA	NA
rs184613584	17	48508221	A	C	0.007125891	6	14.1377974	2.8212234	5.41E-07	NA	NA	NA	NA
rs528288879	2	65875930	C	T	0.005938242	6	14.7878085	2.95121929	5.42E-07	NA	NA	NA	NA
rs1437782	8	55632762	C	T	0.330166271	278	2.2579585	0.45098781	5.54E-07	NA	NA	NA	NA
rs534845494	13	58213864	A	G	0.004750594	5	15.9431765	3.18484007	5.56E-07	NA	NA	NA	NA
rs1008091735	19	31090099	T	C	0.003562945	3	18.4689243	3.69079909	5.61E-07	NA	NA	NA	NA
rs567982164	5	25631297	G	A	0.002375297	3	21.8356286	4.36706703	5.73E-07	NA	NA	NA	NA
rs79213709	11	99093455	G	A	0.032066508	26	6.17081884	1.23453654	5.78E-07	NA	NA	NA	NA
rs559008174	19	18876059	C	T	0.004750594	5	15.6025661	3.12812006	6.11E-07	NA	NA	NA	NA
rs541288561	19	18869445	T	G	0.004750594	5	15.6119012	3.13183483	6.20E-07	NA	NA	NA	NA
rs190251199	14	105590577	T	C	0.003562945	3	20.0125292	4.01627173	6.27E-07	NA	NA	NA	NA
rs138215817	14	22641516	A	G	0.004750594	4	16.311019	3.27367022	6.28E-07	NA	NA	NA	NA
rs111407636	5	95080029	C	T	0.003562945	3	18.642774	3.74168052	6.28E-07	NA	NA	NA	NA
rs73085348	3	42711221	A	G	0.011876485	11	10.139317	2.03504571	6.28E-07	NA	NA	NA	NA
rs1437781	8	55629852	T	C	0.332541568	280	2.23854673	0.44940766	6.32E-07	NA	NA	NA	NA
rs147032554	1	186148864	T	G	0.003562945	3	18.162609	3.65402821	6.68E-07	NA	NA	NA	NA
rs111676272	5	95094298	C	A	0.003562945	3	18.6068625	3.74429748	6.72E-07	NA	NA	NA	NA
rs2274996	1	229804538	C	T	0.041567696	35	5.58527433	1.12406363	6.74E-07	NA	NA	NA	NA

rs2891865	1	229806368	A	G	0.041567696	35	5.58181376	1.12378916	6.80E-07	NA	NA	NA	NA
rs2385790	1	229807492	C	T	0.041567696	35	5.58224822	1.12394723	6.81E-07	NA	NA	NA	NA
rs12024557	1	229812357	A	C	0.042755344	35	5.56930969	1.12195729	6.91E-07	NA	NA	NA	NA
rs78296164	9	135266715	C	T	0.008313539	7	11.6095338	2.33917557	6.94E-07	NA	NA	NA	NA
rs570407448	19	18880030	G	A	0.004750594	5	15.4977984	3.12412555	7.02E-07	NA	NA	NA	NA
rs76777840	2	48312950	G	A	0.005938242	5	14.3500784	2.8927894	7.03E-07	NA	NA	NA	NA
rs528609331	11	125842195	C	T	0.003562945	3	19.5812418	3.94832705	7.07E-07	NA	NA	NA	NA
rs182437250	12	63608466	T	C	0.004750594	4	18.8083451	3.79343554	7.12E-07	NA	NA	NA	NA
rs147601511	8	22322319	A	G	0.004750594	4	16.4471181	3.31777038	7.15E-07	NA	NA	NA	NA
rs858397	8	55614690	A	G	0.331353919	278	2.2301348	0.44990341	7.16E-07	NA	NA	NA	NA
rs75334617	10	102956152	G	A	0.038004751	32	5.84603405	1.18027345	7.30E-07	NA	NA	NA	NA
rs4562666	1	229824770	T	C	0.042755344	36	5.51922599	1.11457457	7.35E-07	NA	NA	NA	NA
rs382476	8	55590975	G	A	0.666270784	288	-2.2481938	0.4540554	7.37E-07	NA	NA	NA	NA
rs384543	8	55591609	G	A	0.666270784	288	-2.2481938	0.4540554	7.37E-07	NA	NA	NA	NA
rs446222	8	55574960	G	A	0.666270784	288	-2.247819	0.45397995	7.37E-07	NA	NA	NA	NA
rs384127	8	55597489	G	A	0.666270784	288	-2.2481559	0.45404811	7.37E-07	NA	NA	NA	NA
rs12045643	1	229834050	C	T	0.042755344	36	5.48149152	1.10708948	7.37E-07	NA	NA	NA	NA
rs147630370	4	87450675	T	C	0.004750594	4	16.0013633	3.23339786	7.47E-07	NA	NA	NA	NA
rs532513136	8	135813748	C	A	0.003562945	3	19.9490594	4.03118599	7.47E-07	NA	NA	NA	NA
rs184265355	13	108011352	A	C	0.005938242	6	14.4271719	2.91829792	7.67E-07	NA	NA	NA	NA
rs148248743	3	136134595	C	T	0.002375297	3	22.839379	4.62044689	7.69E-07	NA	NA	NA	NA
rs72983831	6	142307100	T	G	0.005938242	6	15.6733239	3.17294911	7.83E-07	NA	NA	NA	NA
rs80292573	15	59435086	T	G	0.034441805	30	6.55323491	1.32696583	7.87E-07	NA	NA	NA	NA
rs111927235	2	74483954	A	G	0.004750594	4	17.0468955	3.46111889	8.43E-07	NA	NA	NA	NA
rs369623	8	55571940	A	C	0.666270784	287	-2.2359815	0.45415557	8.51E-07	NA	NA	NA	NA
rs188720948	3	150069880	T	C	0.003562945	3	17.7032653	3.59722874	8.59E-07	NA	NA	NA	NA
rs148998974	22	40620530	A	G	0.005938242	5	14.5129532	2.94976024	8.65E-07	NA	NA	NA	NA
rs144954214	16	76179362	A	G	0.002375297	3	21.8113263	4.43942529	8.96E-07	NA	NA	NA	NA
rs145439370	15	58879765	T	C	0.03087886	26	6.89328891	1.40339117	9.02E-07	NA	NA	NA	NA
rs75689761	7	18406573	C	T	0.008313539	7	11.8482917	2.41320258	9.12E-07	NA	NA	NA	NA
rs113221952	1	103753974	A	G	0.021377672	19	7.92350704	1.61421682	9.17E-07	NA	NA	NA	NA
rs61434999	7	18418351	A	G	0.008313539	7	11.8411511	2.41284027	9.22E-07	NA	NA	NA	NA
rs433324	8	55564609	A	G	0.666270784	287	-2.2483462	0.45814252	9.22E-07	NA	NA	NA	NA
rs528140343	11	125719227	A	C	0.003562945	3	18.2835521	3.72738029	9.33E-07	NA	NA	NA	NA
rs34270375	1	89370702	G	A	0.026128266	21	7.71147017	1.57216792	9.34E-07	NA	NA	NA	NA
rs75773869	7	18410845	G	T	0.008313539	7	11.8642716	2.41902322	9.36E-07	NA	NA	NA	NA
rs79602997	7	18410250	G	A	0.008313539	7	11.8608211	2.41901626	9.43E-07	NA	NA	NA	NA
rs56224400	6	98092675	T	C	0.016627078	14	8.4433064	1.72251471	9.50E-07	NA	NA	NA	NA
rs186532456	1	18948328	C	T	0.003562945	3	20.1730343	4.11737745	9.61E-07	NA	NA	NA	NA
rs118093638	11	18718324	C	T	0.005938242	5	14.2657259	2.91296506	9.72E-07	NA	NA	NA	NA
rs77353774	12	28248852	G	A	0.007125891	6	13.1054362	2.67954103	1.00E-06	NA	NA	NA	NA
rs532730683	9	1784492	G	T	0.003562945	3	18.726838	3.8314675	1.02E-06	NA	NA	NA	NA
rs185139807	22	40594781	G	A	0.005938242	5	14.4330526	2.95312686	1.02E-06	NA	NA	NA	NA
rs146333745	18	55497457	C	T	0.003562945	4	17.4215784	3.56782335	1.04E-06	NA	NA	NA	NA
rs541680196	22	40528090	G	A	0.005938242	5	14.3867808	2.94903659	1.07E-06	NA	NA	NA	NA
rs12036586	1	229826378	G	A	0.047505938	40	5.22212176	1.07114093	1.09E-06	NA	NA	NA	NA
rs529523094	16	77715551	A	G	0.003562945	3	18.1584346	3.72617419	1.10E-06	NA	NA	NA	NA
rs562032622	1	18959333	A	C	0.003562945	3	21.0267115	4.3197503	1.13E-06	NA	NA	NA	NA
rs78225611	7	18407464	A	C	0.009501188	8	11.0294387	2.26622466	1.13E-06	NA	NA	NA	NA
rs77346868	7	18406599	A	G	0.009501188	8	11.0210129	2.26481172	1.14E-06	NA	NA	NA	NA
rs184098071	2	177116420	G	A	0.003562945	3	13.947724	2.86782452	1.15E-06	NA	NA	NA	NA
rs118183140	21	35477486	C	T	0.020190024	17	7.5893248	1.56122232	1.17E-06	NA	NA	NA	NA
rs113625788	22	19969182	C	T	0.008313539	7	11.8084829	2.42941561	1.17E-06	NA	NA	NA	NA
rs116189766	2	126393864	T	C	0.002375297	3	21.190764	4.36037289	1.17E-06	NA	NA	NA	NA
rs76098744	1	112349372	C	T	0.016627078	14	8.38612452	1.72704116	1.20E-06	NA	NA	NA	NA
rs74683551	1	112354418	G	A	0.016627078	14	8.38252043	1.72705812	1.21E-06	NA	NA	NA	NA
rs185855183	12	101505126	C	T	0.003562945	3	18.7397727	3.86137343	1.22E-06	NA	NA	NA	NA
rs405226	8	55592336	A	G	0.662707838	291	-2.1881305	0.45101874	1.23E-06	NA	NA	NA	NA

rs3098298	8	55582838	C	T	0.662707838	291	-2.1875635	0.45094791	1.23E-06	NA	NA	NA	NA
rs367179	8	55587616	T	C	0.662707838	291	-2.1875635	0.45094791	1.23E-06	NA	NA	NA	NA
rs432393	8	55580298	C	T	0.662707838	291	-2.1875382	0.45096346	1.23E-06	NA	NA	NA	NA
rs117185941	21	38766484	G	A	0.005938242	5	15.3808786	3.17143013	1.24E-06	NA	NA	NA	NA
rs1329159859	21	38766484	G	A	0.005938242	5	15.3808786	3.17143013	1.24E-06	NA	NA	NA	NA
rs139360368	5	73372109	A	C	0.003562945	4	18.3590487	3.78701272	1.25E-06	NA	NA	NA	NA
rs184117160	15	59404306	C	T	0.014251781	11	10.3417236	2.13366338	1.25E-06	NA	NA	NA	NA
rs145676540	3	2044635	C	T	0.005938242	5	13.980658	2.88447377	1.25E-06	NA	NA	NA	NA
rs556293455	2	176950420	G	A	0.003562945	3	14.3860463	2.96998402	1.27E-06	NA	NA	NA	NA
rs150027952	9	103385416	A	G	0.002375297	3	21.8616648	4.51464643	1.28E-06	NA	NA	NA	NA
rs138217865	15	94392882	C	T	0.004750594	4	16.709641	3.45193896	1.29E-06	NA	NA	NA	NA
rs112475378	3	1626661	T	C	0.016627078	14	9.55085929	1.97628138	1.35E-06	NA	NA	NA	NA
rs146007933	3	28227423	T	C	0.021377672	18	7.51149453	1.55442805	1.35E-06	NA	NA	NA	NA
rs192443987	3	135532345	G	A	0.004750594	4	17.7540285	3.67564302	1.36E-06	NA	NA	NA	NA
rs149493615	1	79880089	G	A	0.008313539	8	11.750632	2.43451244	1.39E-06	NA	NA	NA	NA
rs140706881	10	96217535	G	A	0.004750594	4	18.974805	3.93288931	1.40E-06	NA	NA	NA	NA
rs142894171	2	151438271	G	T	0.003562945	3	18.0710394	3.74586836	1.41E-06	NA	NA	NA	NA
rs17746486	2	95722609	C	T	0.032066508	29	6.90851358	1.43477175	1.47E-06	NA	NA	NA	NA
rs180764936	12	101498621	T	C	0.003562945	3	19.3473053	4.0200238	1.49E-06	NA	NA	NA	NA
rs190806532	12	48862147	G	T	0.002375297	3	21.326955	4.43856043	1.55E-06	NA	NA	NA	NA
rs147393020	10	124779274	A	G	0.005938242	5	14.1853505	2.95980896	1.65E-06	NA	NA	NA	NA
rs79539453	11	125266353	C	T	0.002375297	3	20.8220616	4.34542406	1.65E-06	NA	NA	NA	NA
rs371245624	5	162880956	T	C	0.003562945	3	19.4795931	4.06572011	1.66E-06	NA	NA	NA	NA
rs536803366	8	123001411	T	C	0.002375297	3	19.2098639	4.00952057	1.66E-06	NA	NA	NA	NA
rs555249476	8	123001411	T	C	0.002375297	3	19.2098639	4.00952057	1.66E-06	NA	NA	NA	NA
rs150077525	13	57979549	A	G	0.007125891	7	12.9984875	2.71312414	1.66E-06	NA	NA	NA	NA
rs371879555	12	23015962	T	C	0.003562945	3	20.1033765	4.20002323	1.70E-06	NA	NA	NA	NA
rs571986619	6	85678832	A	G	0.002375297	3	20.7858067	4.34301983	1.70E-06	NA	NA	NA	NA
rs183586634	21	38763032	G	A	0.005938242	5	14.3132202	2.99210048	1.72E-06	NA	NA	NA	NA
rs143811231	1	79968131	T	C	0.008313539	8	11.5747409	2.42066027	1.74E-06	NA	NA	NA	NA
rs2365739	1	62484462	G	A	0.021377672	18	6.86815917	1.43887996	1.81E-06	NA	NA	NA	NA
rs146526206	4	90993018	T	C	0.017814727	15	8.09176656	1.69535286	1.82E-06	NA	NA	NA	NA
rs567383525	8	115029494	C	T	0.003562945	3	17.7455227	3.71970627	1.84E-06	NA	NA	NA	NA
rs149425014	15	58951660	T	C	0.026128266	22	7.55224792	1.58320604	1.84E-06	NA	NA	NA	NA
rs559559983	1	246977132	C	A	0.007125891	5	13.811132	2.89725588	1.87E-06	NA	NA	NA	NA
rs117166500	7	17052778	G	T	0.007125891	7	12.5146865	2.62694198	1.90E-06	NA	NA	NA	NA
rs145766563	1	185129502	G	A	0.003562945	4	16.8682102	3.54127008	1.90E-06	NA	NA	NA	NA
rs540065886	9	2770228	T	C	0.003562945	3	19.9489151	4.18802805	1.90E-06	NA	NA	NA	NA
rs118084887	21	38863820	T	C	0.005938242	5	14.9382616	3.13674741	1.91E-06	NA	NA	NA	NA
rs140420703	1	102803484	T	G	0.004750594	5	16.487654	3.46213891	1.91E-06	NA	NA	NA	NA
rs568321148	2	29870348	T	G	0.003562945	3	17.7125046	3.7201251	1.92E-06	NA	NA	NA	NA
rs55844051	7	23360363	T	C	0.003562945	3	18.3875896	3.86268704	1.93E-06	NA	NA	NA	NA
rs187236873	5	91530447	G	A	0.007125891	6	12.44907	2.61780937	1.98E-06	NA	NA	NA	NA
rs1812506	8	55676101	A	G	0.345605701	291	2.12258868	0.4466178	2.01E-06	NA	NA	NA	NA
rs539713344	7	100474786	G	A	0.002375297	3	20.044756	4.21957545	2.03E-06	NA	NA	NA	NA
rs536781978	2	29733681	A	G	0.003562945	3	17.5870824	3.70259451	2.03E-06	NA	NA	NA	NA
rs76327548	12	101182966	G	A	0.013064133	11	9.17842714	1.93235231	2.04E-06	NA	NA	NA	NA
rs149421869	2	53483429	G	T	0.008313539	8	11.7382186	2.47204376	2.05E-06	NA	NA	NA	NA
rs75024143	21	23156546	G	T	0.014251781	12	9.81254241	2.0684542	2.10E-06	NA	NA	NA	NA
rs2375536	8	55640722	T	C	0.347980998	292	2.12635806	0.44857091	2.13E-06	NA	NA	NA	NA
rs141326851	6	134833127	A	C	0.016627078	14	8.57849688	1.81053268	2.16E-06	NA	NA	NA	NA
rs187518659	1	99455745	T	G	0.009501188	8	11.6819623	2.46564537	2.16E-06	NA	NA	NA	NA
rs146479102	2	65825759	G	A	0.005938242	5	15.4846768	3.26980199	2.18E-06	NA	NA	NA	NA
rs16850124	1	229831331	T	C	0.043942993	37	5.18095662	1.09435188	2.20E-06	NA	NA	NA	NA
rs115348382	1	9655903	G	A	0.003562945	3	18.2107495	3.84721698	2.21E-06	NA	NA	NA	NA
rs184200893	2	69260913	C	T	0.003562945	3	19.9580356	4.21905487	2.24E-06	NA	NA	NA	NA
rs148781275	11	103640603	A	G	0.004750594	5	15.7111853	3.32183832	2.25E-06	NA	NA	NA	NA
rs139943877	3	155451289	G	A	0.007125891	7	13.0535902	2.76041594	2.26E-06	NA	NA	NA	NA

rs546409459	11	125754989	A	G	0.003562945	3	18.9195474	4.00286836	2.28E-06	NA	NA	NA	NA
rs186649043	3	174932293	C	T	0.003562945	3	19.2134209	4.06588786	2.30E-06	NA	NA	NA	NA
rs80203220	3	122722331	C	T	0.005938242	6	13.426042	2.84218656	2.31E-06	NA	NA	NA	NA
rs541508507	2	170023265	G	A	0.004750594	4	15.4630033	3.27389618	2.32E-06	NA	NA	NA	NA
rs529011661	10	20372316	G	A	0.004750594	4	15.6393469	3.3125339	2.34E-06	NA	NA	NA	NA
rs182959028	22	45823032	T	C	0.008313539	7	11.944461	2.53146	2.38E-06	NA	NA	NA	NA
rs146207930	9	129042336	A	G	0.007125891	6	11.8624321	2.51502465	2.40E-06	NA	NA	NA	NA
rs290120	5	163268244	T	G	0.007125891	6	13.3587764	2.83260801	2.40E-06	NA	NA	NA	NA
rs531769270	4	154411043	T	C	0.003562945	3	16.5945338	3.51936092	2.41E-06	NA	NA	NA	NA
rs185620578	12	48569399	C	T	0.002375297	3	20.7232267	4.39505442	2.42E-06	NA	NA	NA	NA
rs1301444047	7	36574504	G	A	0.042755344	43	5.12842209	1.08835549	2.45E-06	NA	NA	NA	NA
rs62447184	7	36574504	G	A	0.042755344	43	5.12842209	1.08835549	2.45E-06	NA	NA	NA	NA
rs118184666	12	20424749	G	A	0.007125891	6	10.9509803	2.32477242	2.47E-06	NA	NA	NA	NA
rs10494861	1	205331874	G	A	0.003562945	3	19.1820498	4.0729134	2.48E-06	NA	NA	NA	NA
rs183817723	16	59302775	C	T	0.003562945	4	17.5860507	3.7341919	2.48E-06	NA	NA	NA	NA
rs12502861	4	2426305	T	C	0.010688836	9	11.1588472	2.36961887	2.49E-06	NA	NA	NA	NA
rs191792521	3	195646605	G	A	0.008313539	6	13.9515484	2.96443599	2.52E-06	NA	NA	NA	NA
rs149949098	11	95099866	G	A	0.016627078	13	8.55927625	1.81883308	2.53E-06	NA	NA	NA	NA
rs74343174	5	161493182	C	A	0.005938242	5	14.7671824	3.13913408	2.55E-06	NA	NA	NA	NA
rs189765693	4	4324793	T	C	0.004750594	4	15.5191341	3.29924607	2.55E-06	NA	NA	NA	NA
rs189912648	5	134765439	C	T	0.003562945	4	16.9732599	3.60846227	2.55E-06	NA	NA	NA	NA
rs151323346	12	21012024	T	C	0.005938242	4	15.8903888	3.38034868	2.59E-06	NA	NA	NA	NA
rs536023430	7	146865072	T	C	0.003562945	3	19.4038683	4.13084856	2.64E-06	NA	NA	NA	NA
rs144026361	15	41248669	C	T	0.004750594	5	16.1372994	3.43734659	2.67E-06	NA	NA	NA	NA
rs186142189	2	67702707	G	A	0.007125891	6	12.7361927	2.71405368	2.70E-06	NA	NA	NA	NA
rs142549310	2	170030506	C	T	0.004750594	4	15.2501713	3.24983634	2.70E-06	NA	NA	NA	NA
rs72832764	5	170004673	G	A	0.003562945	3	18.2159757	3.88196743	2.70E-06	NA	NA	NA	NA
rs183180157	1	181247121	A	C	0.009501188	7	12.61386	2.68921121	2.72E-06	NA	NA	NA	NA
rs73227413	21	23136973	G	A	0.034441805	28	5.96205514	1.27150176	2.75E-06	NA	NA	NA	NA
rs185874707	8	18030674	C	T	0.011876485	10	9.13934765	1.94918644	2.75E-06	NA	NA	NA	NA
rs191271637	17	52123260	A	G	0.003562945	3	18.379836	3.92083355	2.76E-06	NA	NA	NA	NA
rs187978759	7	11711845	G	A	0.003562945	3	20.4555522	4.3637719	2.76E-06	NA	NA	NA	NA
rs193153124	3	148330710	A	G	0.005938242	5	14.3657313	3.06514594	2.78E-06	NA	NA	NA	NA
rs1877768	6	16534923	C	T	0.017814727	15	8.28754685	1.76830748	2.78E-06	NA	NA	NA	NA
rs76617932	1	180930424	T	C	0.011876485	9	10.5449608	2.25040726	2.79E-06	NA	NA	NA	NA
rs188034471	9	85937714	G	A	0.004750594	4	14.7559999	3.14925113	2.79E-06	NA	NA	NA	NA
rs1595406	8	55630615	A	G	0.345605701	291	2.10018889	0.44830064	2.80E-06	NA	NA	NA	NA
rs147627638	6	99173116	A	G	0.007125891	6	13.0785306	2.79364254	2.85E-06	NA	NA	NA	NA
rs151272830	2	67682724	G	T	0.007125891	6	13.3323479	2.84806076	2.85E-06	NA	NA	NA	NA
rs141169929	3	164808462	A	G	0.004750594	5	14.8454178	3.1738839	2.91E-06	NA	NA	NA	NA
rs720372	8	55628637	G	A	0.346793349	292	2.09872734	0.44886259	2.93E-06	NA	NA	NA	NA
rs567080482	13	95075405	T	C	0.004750594	4	15.4999561	3.31620837	2.95E-06	NA	NA	NA	NA
rs546286713	13	91411079	G	A	0.004750594	4	15.7027841	3.3611216	2.98E-06	NA	NA	NA	NA
rs142311947	5	177384469	G	A	0.007125891	8	12.0339013	2.57618116	2.99E-06	NA	NA	NA	NA
rs185155853	15	41244100	C	T	0.004750594	5	15.9164657	3.40838631	3.02E-06	NA	NA	NA	NA
rs550763536	2	7452347	T	G	0.005938242	5	14.4871647	3.10349768	3.04E-06	NA	NA	NA	NA
rs555040883	22	40631476	G	A	0.004750594	4	15.4273069	3.30542717	3.05E-06	NA	NA	NA	NA
rs2375219	8	55698295	C	T	0.393111639	330	2.05700167	0.44073069	3.05E-06	NA	NA	NA	NA
rs140062526	13	59286033	G	A	0.005938242	5	14.0926088	3.02052903	3.08E-06	NA	NA	NA	NA
rs572961122	13	108012985	C	T	0.005938242	6	13.3354896	2.85827607	3.08E-06	NA	NA	NA	NA
rs544042801	11	68460243	G	A	0.004750594	4	15.8869866	3.40519019	3.08E-06	NA	NA	NA	NA
rs142993106	4	90957372	G	A	0.017814727	15	8.08463611	1.73306162	3.09E-06	NA	NA	NA	NA
rs569916471	14	75891342	G	A	0.004750594	5	15.6404088	3.3537133	3.11E-06	NA	NA	NA	NA
rs7104959	11	129846126	C	T	0.003562945	3	17.9204611	3.84366851	3.13E-06	NA	NA	NA	NA
rs559228693	20	15963329	G	A	0.005938242	5	14.3643551	3.08196497	3.15E-06	NA	NA	NA	NA
rs187384541	8	1632241	A	G	0.035629454	26	6.88745117	1.47776668	3.15E-06	NA	NA	NA	NA
rs141127122	22	40604439	G	A	0.004750594	4	15.3824875	3.30083549	3.16E-06	NA	NA	NA	NA
rs193093906	10	126705489	G	A	0.009501188	9	10.5219537	2.25840517	3.18E-06	NA	NA	NA	NA

rs77871739	9	138552309	G	A	0.004750594	4	15.0724603	3.23606181	3.20E-06	NA	NA	NA	NA
rs117816016	8	103751262	C	T	0.003562945	3	19.8807023	4.26960212	3.22E-06	NA	NA	NA	NA
rs775626702	5	162630067	A	C	0.003562945	3	18.9484137	4.07023372	3.23E-06	NA	NA	NA	NA
rs146048121	6	142198955	G	A	0.002375297	3	20.0214379	4.30086025	3.24E-06	NA	NA	NA	NA
rs139493286	18	28816019	G	A	0.003562945	3	17.4603093	3.75076428	3.24E-06	NA	NA	NA	NA
rs146442492	15	58982115	C	T	0.027315914	24	7.05925858	1.51661316	3.25E-06	NA	NA	NA	NA
rs181259864	7	97488823	C	A	0.003562945	3	17.1360821	3.68242789	3.26E-06	NA	NA	NA	NA
rs150946694	22	46853180	T	C	0.004750594	4	15.3082479	3.29125059	3.30E-06	NA	NA	NA	NA
rs181933850	5	91465647	A	G	0.008313539	7	11.0436586	2.37544216	3.33E-06	NA	NA	NA	NA
rs113006316	2	74802360	A	G	0.002375297	3	22.8886792	4.92411335	3.35E-06	NA	NA	NA	NA
rs191006910	5	154216009	G	A	0.003562945	3	17.6173803	3.79049689	3.36E-06	NA	NA	NA	NA
rs176786	14	46282970	T	C	0.32304038	268	2.28475641	0.49171936	3.38E-06	NA	NA	NA	NA
rs187047882	3	164285621	G	A	0.003562945	3	18.5641361	3.99725417	3.41E-06	NA	NA	NA	NA
rs73057656	3	33940571	A	G	0.036817102	33	5.9238329	1.27589711	3.44E-06	NA	NA	NA	NA
rs112557251	2	188375400	T	C	0.002375297	3	20.6510064	4.44850106	3.45E-06	NA	NA	NA	NA
rs190190051	5	91475485	G	A	0.008313539	7	11.0589887	2.38317975	3.48E-06	NA	NA	NA	NA
rs180989936	1	193044178	A	G	0.004750594	4	17.1793579	3.70215494	3.48E-06	NA	NA	NA	NA
rs188415494	8	25613298	C	T	0.003562945	3	18.5169478	3.9914865	3.50E-06	NA	NA	NA	NA
rs1686289	14	46260982	G	A	0.678147268	277	-2.2551539	0.48625976	3.52E-06	NA	NA	NA	NA
rs76904423	12	101188744	G	A	0.010688836	10	9.80576231	2.11568908	3.57E-06	NA	NA	NA	NA
rs183466664	12	26821687	A	G	0.004750594	4	16.0181882	3.45609016	3.57E-06	NA	NA	NA	NA
rs999769259	4	62511965	G	A	0.003562945	3	22.1246725	4.77597881	3.61E-06	NA	NA	NA	NA
rs553840536	16	25697895	A	G	0.003562945	3	19.6203442	4.23547954	3.61E-06	NA	NA	NA	NA
rs183962155	4	21259643	A	C	0.005938242	6	13.7646008	2.97247759	3.64E-06	NA	NA	NA	NA
rs138480898	1	184955657	C	T	0.003562945	3	16.9873974	3.66972373	3.67E-06	NA	NA	NA	NA
rs185158855	2	223650026	C	A	0.004750594	4	16.6678864	3.60266524	3.72E-06	NA	NA	NA	NA
rs180926150	1	103226326	C	T	0.002375297	3	19.641918	4.24793649	3.77E-06	NA	NA	NA	NA
rs146728064	17	19265440	G	A	0.007125891	6	12.6400411	2.73467115	3.80E-06	NA	NA	NA	NA
rs111900874	7	89516669	G	A	0.014251781	12	8.42424013	1.82258334	3.80E-06	NA	NA	NA	NA
rs574076561	7	49544747	A	G	0.003562945	3	19.6434766	4.25225723	3.85E-06	NA	NA	NA	NA
rs76554191	2	95967628	G	A	0.040380048	34	5.72148039	1.2389174	3.87E-06	NA	NA	NA	NA
rs176783	14	46280913	A	G	0.321852732	267	2.28010147	0.49376374	3.88E-06	NA	NA	NA	NA
rs140352232	2	108038112	G	A	0.002375297	3	21.0576511	4.56013129	3.88E-06	NA	NA	NA	NA
rs139062456	8	13251991	C	T	0.017814727	15	7.83515809	1.69687423	3.89E-06	NA	NA	NA	NA
rs558614420	15	41810870	C	T	0.005938242	6	13.9750831	3.02772732	3.92E-06	NA	NA	NA	NA
rs12266995	10	24852783	T	C	0.03087886	26	5.96782535	1.29311959	3.93E-06	NA	NA	NA	NA
rs192750513	7	97577830	A	G	0.003562945	3	17.1892469	3.72514218	3.94E-06	NA	NA	NA	NA
rs56302696	12	48292830	G	A	0.002375297	3	19.8959041	4.31584707	4.03E-06	NA	NA	NA	NA
rs151015676	5	177390937	T	G	0.002375297	3	20.1421367	4.37074482	4.06E-06	NA	NA	NA	NA
rs72837643	5	170188955	T	C	0.003562945	4	16.9302874	3.67743479	4.15E-06	NA	NA	NA	NA
rs143048774	14	46294660	A	C	0.317102138	265	2.28482299	0.49630858	4.15E-06	NA	NA	NA	NA
rs428110	14	46294660	A	C	0.317102138	265	2.28482299	0.49630858	4.15E-06	NA	NA	NA	NA
rs111391231	7	89497045	T	C	0.014251781	12	8.31717755	1.80720714	4.18E-06	NA	NA	NA	NA
rs180765647	13	114427311	G	T	0.002375297	3	20.1246521	4.37397806	4.20E-06	NA	NA	NA	NA
rs568658857	12	49853998	G	A	0.005938242	5	14.2361193	3.09423422	4.21E-06	NA	NA	NA	NA
rs182531466	5	91530073	C	A	0.007125891	6	12.6804978	2.75685261	4.23E-06	NA	NA	NA	NA
rs181812512	11	66665729	C	T	0.003562945	3	19.5093168	4.24219816	4.25E-06	NA	NA	NA	NA
rs556680896	13	101602415	C	T	0.003562945	3	17.0254659	3.70286799	4.27E-06	NA	NA	NA	NA
rs142928734	13	101601082	G	A	0.003562945	3	17.009593	3.70044989	4.29E-06	NA	NA	NA	NA
rs143287889	4	35572280	C	T	0.002375297	3	19.96741	4.34406	4.30E-06	NA	NA	NA	NA
rs543844012	9	30107023	C	T	0.003562945	3	18.1160485	3.9446103	4.38E-06	NA	NA	NA	NA
rs566018180	10	86755052	C	T	0.003562945	3	18.563552	4.04215862	4.38E-06	NA	NA	NA	NA
rs147171192	4	89135588	A	G	0.007125891	5	13.3044047	2.89758492	4.40E-06	NA	NA	NA	NA
rs137873790	5	97087041	A	G	0.013064133	11	9.22240881	2.01095379	4.52E-06	NA	NA	NA	NA
rs752259256	10	104925319	T	C	0.003562945	3	20.4258639	4.45411591	4.52E-06	NA	NA	NA	NA
rs185771987	5	73285489	T	C	0.003562945	3	18.2344824	3.97675014	4.53E-06	NA	NA	NA	NA
rs557092705	20	34189911	C	T	0.003562945	3	19.4769546	4.2480073	4.54E-06	NA	NA	NA	NA
rs140642138	15	42125165	G	A	0.005938242	6	13.5832221	2.96274049	4.55E-06	NA	NA	NA	NA

rs139877408	2	129606273	A	G	0.002375297	3	20.3067129	4.43052497	4.58E-06	NA	NA	NA	NA
rs191986449	5	25745687	C	T	0.004750594	3	18.4978793	4.03679831	4.60E-06	NA	NA	NA	NA
rs11690187	2	67565909	A	C	0.005938242	5	13.6310532	2.9751322	4.61E-06	NA	NA	NA	NA
rs118040657	10	3472846	C	T	0.008313539	8	12.1147712	2.64434694	4.62E-06	NA	NA	NA	NA
rs138109686	15	42051442	A	G	0.005938242	6	13.5956705	2.96761718	4.62E-06	NA	NA	NA	NA
rs563167766	1	102866365	G	A	0.002375297	3	19.415739	4.24240395	4.73E-06	NA	NA	NA	NA
rs545550279	8	115552429	G	T	0.003562945	3	19.1812421	4.19212988	4.75E-06	NA	NA	NA	NA
rs1498183	8	55716905	C	T	0.394299287	332	2.0245746	0.44255141	4.77E-06	NA	NA	NA	NA
rs375790303	1	184530482	G	A	0.008313539	7	11.4240393	2.49719192	4.77E-06	NA	NA	NA	NA
rs1396896	8	55695310	A	G	0.397862233	334	1.99853327	0.43690452	4.78E-06	NA	NA	NA	NA
rs7843693	8	55692112	G	A	0.397862233	334	1.99856756	0.43695394	4.79E-06	NA	NA	NA	NA
rs1391462	8	55699781	C	A	0.397862233	334	1.9984766	0.43696027	4.79E-06	NA	NA	NA	NA
rs150539922	21	43276916	T	C	0.003562945	3	16.6696937	3.64477381	4.79E-06	NA	NA	NA	NA
rs145896760	15	42119222	G	A	0.005938242	6	13.3688825	2.92346809	4.81E-06	NA	NA	NA	NA
rs181415102	4	112689266	T	C	0.003562945	4	18.2631019	3.99387944	4.81E-06	NA	NA	NA	NA
rs6080	15	58837933	C	A	0.043942993	36	5.66476745	1.2393253	4.86E-06	NA	NA	NA	NA
rs138249376	10	63515829	T	G	0.004750594	4	16.4300711	3.59466791	4.86E-06	NA	NA	NA	NA
rs529345909	11	67110852	A	G	0.003562945	3	18.70153	4.09206035	4.87E-06	NA	NA	NA	NA
rs191930622	12	48284655	G	A	0.002375297	3	19.6783579	4.30610368	4.88E-06	NA	NA	NA	NA
rs145116559	4	112677596	T	C	0.003562945	4	18.2441224	3.9932536	4.91E-06	NA	NA	NA	NA
rs12678939	8	55705021	A	G	0.394299287	331	2.01975618	0.44220051	4.94E-06	NA	NA	NA	NA
rs73586304	6	142839425	C	T	0.003562945	3	18.2405116	3.99428202	4.96E-06	NA	NA	NA	NA
rs113767990	14	81717563	G	A	0.004750594	4	14.5811155	3.19363095	4.98E-06	NA	NA	NA	NA

Supplementary Table S7. Independent Replication

Florida-1 cohort, gene-based replication of top 26 prioritized genes

Gene	Chr	Start	End	No.SNPs	Pvalue	Pvalue_adjusted
RBFOX1	16	6069095	7763340	54	0.00026108	0.003394054
CSMD1	8	2792875	4852494	50	0.00333806	0.021697393
HDAC9	7	18126572	19042039	13	0.02338453	0.101332968
PRKD1	14	30045687	30661104	5	0.03541744	0.115106687
NCAM2	21	22370633	22915650	5	0.05728018	0.139142201
PPM1H	12	63037762	63328817	4	0.06421948	0.139142201
SPTY2D1	11	18627948	18656338	1	0.07862637	0.146020404
AGAP1	2	236402733	237040444	2	0.09194578	0.149411895
SGCG	13	23755091	23899304	1	0.12415358	0.179332944
HDAC4	2	239969864	240323348	1	0.27330078	0.35529101
GPLD1	6	24424793	24495433	2	0.5232773	0.618418633
VTA1	6	142468367	142545826	1	0.65065227	0.704873297
WWC1	5	167718656	167899308	1	0.90507783	0.90507783
ARHGEF26	3	153838792	153975616	0		
ASB3	2	53897117	54014090	0		
BEND7	10	13480484	13570974	0		
C4orf19	4	37455563	37625117	0		
CCR6	6	167525295	167553184	0		
COL11A1	1	103342023	103574052	0		
DIRAS2	9	93372114	93405386	0		
FST	5	52776239	52782964	0		
KAZALD1	10	102821598	102827888	0		
PPARGC1A	4	23756664	23905712	0		
USP25	21	17102344	17252377	0		
ZNF728	19	23158270	23185978	0		
ZNF737	19	20718631	20748615	0		

*signed MB score test that collapse SNPs with MAF < 0.01

Supplementary Table S7. Independent Replication

Chennai-1 cohort, case-control data, comparing to discovery 12M quantitative trait (QT) data

Threshold for replicative significance:	5.00E-02
Total SNPs found:	37
Total risk loci found:	21

NA: not applicable

rsid	chr	pos_37	REF	ALT	n.obs	caf	MAC	Est	Est.SE	Score.pval	pval.chennai	adj pval SNPs	adj pval loci	Closest gene	Prioritized gene
rs76098744	1	112349372	C	T	439	0.01594533	14	9.5685657	2.04635193	2.93E-06	6.78E-02	1.00E+00	1.00E+00	KCND3	KCND3
rs74683551	1	112354418	G	A	439	0.01594533	14	9.54722832	2.04640679	3.08E-06	6.78E-02	1.00E+00	1.00E+00	KCND3	KCND3
rs2606194	17	77210823	A	G	439	0.948747153	44	-5.6956979	1.16435773	1.00E-06	9.08E-02	1.00E+00	1.00E+00	RBFOX3	RBFOX3
rs7534177	1	65965720	A	G	439	0.056947608	49	4.96123378	1.0598616	2.85E-06	1.44E-01	1.00E+00	1.00E+00	LEPR	LEPR
rs2427460	20	61590782	T	C	439	0.486332574	420	-2.574044	0.51325892	5.30E-07	1.72E-01	1.00E+00	1.00E+00	SLC17A9	SLC17A9
rs77297738	22	34473543	C	T	439	0.018223235	16	10.2480572	1.97167592	2.02E-07	1.98E-01	1.00E+00	1.00E+00	LARGE1	LARGE1
rs74572772	22	34492649	A	G	439	0.018223235	16	10.2225183	1.94126774	1.40E-07	1.98E-01	1.00E+00	1.00E+00	LARGE1	LARGE1
rs80019988	22	34493647	G	A	439	0.018223235	16	10.0623306	1.91665407	1.52E-07	1.98E-01	1.00E+00	1.00E+00	LARGE1	LARGE1
rs17127656	1	65943471	C	T	439	0.055808656	49	5.07046538	1.05780641	1.64E-06	2.75E-01	1.00E+00	1.00E+00	LEPR	LEPR
rs7518849	1	65948791	T	C	439	0.055808656	49	5.07456956	1.05964469	1.68E-06	2.75E-01	1.00E+00	1.00E+00	LEPR	LEPR
rs11579567	1	65957141	C	A	439	0.055808656	49	5.02973793	1.06097379	2.13E-06	2.75E-01	1.00E+00	1.00E+00	LEPR	LEPR
rs35145334	1	23465122	A	G	439	0.017084282	14	10.6552656	2.24620792	2.10E-06	2.96E-01	1.00E+00	1.00E+00	LUZP1	LUZP1
rs142956968	10	23490165	C	T	439	0.01594533	14	10.4167992	2.11909083	8.85E-07	2.96E-01	1.00E+00	1.00E+00	C10orf67	OTUD1
rs145764464	10	23601847	G	A	439	0.017084282	15	9.5967306	1.96976348	1.10E-06	2.96E-01	1.00E+00	1.00E+00	C10orf67	OTUD1
rs117699122	12	560262	T	C	439	0.003416856	4	21.4238256	4.40952328	1.18E-06	2.96E-01	1.00E+00	1.00E+00	CCDC77	CCDC77
rs149616342	12	99178813	C	T	439	0.011389522	10	11.2978214	2.454657	4.17E-06	2.96E-01	1.00E+00	1.00E+00	ANKS1B	ANKS1B
rs62192733	20	1928157	A	G	439	0.006833713	7	15.744618	3.35866563	2.76E-06	2.96E-01	1.00E+00	1.00E+00	PDYN	SIRPA
rs117591241	3	190584679	A	G	439	0.003416856	4	19.9014028	4.33331389	4.38E-06	3.34E-01	1.00E+00	1.00E+00	GMNC	GMNC
rs17615362	4	169934087	G	A	439	0.050113895	44	5.41974472	1.17918425	4.30E-06	3.34E-01	1.00E+00	1.00E+00	CBR4	CBR4
rs17543620	4	169934725	T	C	439	0.050113895	44	5.42796755	1.17982097	4.21E-06	3.34E-01	1.00E+00	1.00E+00	CBR4	CBR4
rs62515405	8	57055978	G	A	439	0.023917995	21	8.07691204	1.76428755	4.69E-06	3.34E-01	1.00E+00	1.00E+00	PLAG1	PLAG1
rs113244573	12	6684385	G	A	439	0.023917995	21	7.97095746	1.73766156	4.49E-06	3.34E-01	1.00E+00	1.00E+00	CHD4	CHD4
rs139816293	20	30921343	C	T	439	0.005694761	6	17.1384248	3.52537223	1.17E-06	3.34E-01	1.00E+00	1.00E+00	KIF3B	KIF3B
rs76356799	3	179593768	G	A	439	0.004555809	4	19.2867007	3.76039319	2.91E-07	5.13E-01	1.00E+00	1.00E+00	PEX5L	PEX5L
rs147944608	10	110423709	C	T	439	0.012528474	11	11.2205243	2.29229161	9.84E-07	5.84E-01	1.00E+00	1.00E+00	XPNPEP1	XPNPEP1
rs4896997	6	148683924	C	T	439	0.006833713	6	14.4320923	3.09188376	3.05E-06	6.04E-01	1.00E+00	1.00E+00	SASH1	SASH1
rs17078283	6	148696920	C	T	439	0.006833713	6	14.4302821	3.0918048	3.05E-06	6.04E-01	1.00E+00	1.00E+00	SASH1	SASH1
rs56821264	6	148703901	C	T	439	0.006833713	6	14.7509213	3.11419566	2.17E-06	6.04E-01	1.00E+00	1.00E+00	SASH1	SASH1
rs9981433	21	41309565	G	T	439	0.881548975	116	-4.1220124	0.84715422	1.14E-06	7.75E-01	1.00E+00	1.00E+00	PCP4	PCP4
rs12114488	8	62672723	G	A	439	0.30523918	266	2.62307914	0.54257702	1.33E-06	8.99E-01	1.00E+00	1.00E+00	MIR4470	ASPH
rs9636964	21	41304765	A	G	439	0.884965831	115	-4.2543192	0.82744371	2.73E-07	9.23E-01	1.00E+00	1.00E+00	PCP4	PCP4
rs9305683	21	41305720	G	A	439	0.88952164	112	-4.1163679	0.83634363	8.57E-07	9.23E-01	1.00E+00	1.00E+00	PCP4	PCP4
rs9974985	21	41307573	G	A	439	0.886104784	114	-4.3459395	0.82924282	1.60E-07	9.23E-01	1.00E+00	1.00E+00	PCP4	PCP4
rs7275595	21	41307923	G	A	439	0.888382688	113	-4.2609754	0.83407868	3.25E-07	9.23E-01	1.00E+00	1.00E+00	PCP4	PCP4
rs1005412	21	41308948	A	G	439	0.887243736	112	-4.4521096	0.85237138	1.76E-07	9.23E-01	1.00E+00	1.00E+00	PCP4	PCP4
rs75082290	2	67831153	T	G	439	0.078587699	69	4.69395387	0.98072733	1.70E-06	9.56E-01	1.00E+00	1.00E+00	ETAA1	ETAA1
rs61918041	12	6681868	C	T	439	0.023917995	21	7.97264643	1.73636056	4.40E-06	9.56E-01	1.00E+00	1.00E+00	CHD4	CHD4
rs139390630	1	84866196	G	A	439	0.011389522	13	11.6975971	2.4994863	2.87E-06	NA				
rs114067899	1	85191044	G	A	439	0.007972665	7	13.7760673	2.90836365	2.17E-06	NA				
rs145804766	1	202122499	C	T	439	0.010250569	9	12.5864465	2.61118962	1.43E-06	NA				
rs147869916	1	227307652	A	G	439	0.005694761	5	17.5964551	3.78625881	3.36E-06	NA				
rs76220567	2	13059945	T	C	439	0.003416856	4	20.6828868	4.35776216	2.07E-06	NA				
rs536781978	2	29733681	A	G	439	0.003416856	3	20.5561882	4.34258099	2.21E-06	NA				

rs568321148	2	29870348	T	G	439	0.003416856	3	20.7400203	4.36652717	2.04E-06	NA
rs186792608	2	43096699	A	G	439	0.003416856	3	22.4512499	4.28172841	1.58E-07	NA
rs1162141034	2	67825685	C	T	439	0.079726651	71	4.55991411	0.98044721	3.31E-06	NA
rs146465493	2	67825685	C	T	439	0.079726651	71	4.55991411	0.98044721	3.31E-06	NA
rs2902021	2	67825685	C	T	439	0.079726651	71	4.55991411	0.98044721	3.31E-06	NA
rs191298981	2	68110046	C	T	439	0.004555809	3	24.0078714	4.85703224	7.70E-07	NA
rs113537164	2	68234930	A	C	439	0.004555809	4	20.3890413	4.17097432	1.02E-06	NA
rs113154814	2	68289497	T	C	439	0.005694761	5	19.8310188	3.7892894	1.66E-07	NA
rs146919974	2	115252863	T	C	439	0.004555809	5	19.7280466	3.99554232	7.91E-07	NA
rs79935606	2	177338635	T	C	439	0.005694761	5	16.3396833	3.47376974	2.55E-06	NA
rs144788248	3	19586745	T	C	439	0.011389522	10	11.9086237	2.57879124	3.88E-06	NA
rs149706477	3	66148228	A	G	439	0.003416856	3	20.9828687	4.4842531	2.88E-06	NA
rs114280794	3	132842203	G	A	439	0.01594533	14	8.76296877	1.89632016	3.82E-06	NA
rs148248743	3	136134595	C	T	439	0.002277904	3	27.1548587	5.46352726	6.69E-07	NA
rs138138661	3	147657814	C	T	439	0.004555809	4	23.1347457	4.25695836	5.49E-08	NA
rs189234695	3	147658158	T	C	439	0.003416856	3	23.9697134	5.16542129	3.48E-06	NA
rs148997617	3	147755576	C	T	439	0.004555809	4	20.8774277	3.92648902	1.05E-07	NA
rs148483098	3	157382051	C	A	439	0.005694761	5	15.7357844	3.38211024	3.28E-06	NA
rs79252854	3	157523157	C	T	439	0.004555809	5	17.5464336	3.72541417	2.48E-06	NA
rs139055031	3	158586849	T	C	439	0.005694761	6	16.2853761	3.4955337	3.18E-06	NA
rs143669489	3	159392885	G	A	439	0.003416856	3	24.2253199	5.30400674	4.94E-06	NA
rs189709453	3	177574308	G	A	439	0.003416856	3	23.2009936	4.82954374	1.56E-06	NA
rs186767531	3	177639777	T	C	439	0.003416856	3	23.0749977	4.81664642	1.66E-06	NA
rs182868205	3	177712448	C	T	439	0.003416856	3	22.8544303	4.73215662	1.37E-06	NA
rs1472243465	3	190584679	A	G	439	0.003416856	4	19.9014028	4.33331389	4.38E-06	NA
rs189094663	4	11623450	G	A	439	0.002277904	3	23.895416	5.10822708	2.90E-06	NA
rs557276277	4	19013698	T	C	439	0.005694761	5	19.0146344	4.09137627	3.36E-06	NA
rs113063005	4	23509067	T	C	439	0.006833713	7	16.61763	3.39113209	9.57E-07	NA
rs147267707	4	43026558	G	A	439	0.004555809	4	18.0094385	3.80818408	2.25E-06	NA
rs77618729	4	43050968	T	C	439	0.004555809	4	18.0130153	3.80914173	2.26E-06	NA
rs556435089	4	43551033	G	A	439	0.004555809	4	17.939079	3.81772146	2.62E-06	NA
rs532464521	4	43571039	T	C	439	0.004555809	4	17.9895528	3.81820438	2.46E-06	NA
rs181132315	4	129647369	C	T	439	0.003416856	3	22.1324679	4.62266164	1.69E-06	NA
rs147455971	4	140931712	T	C	439	0.007972665	5	17.2160142	3.72793057	3.87E-06	NA
rs142934021	5	52662672	G	A	439	0.007972665	7	18.824715	3.31820294	1.40E-08	NA
rs189912648	5	134765439	C	T	439	0.003416856	4	19.8604449	4.2716652	3.33E-06	NA
rs111512950	5	153680427	C	T	439	0.009111617	8	13.567443	2.94598493	4.12E-06	NA
rs73384619	6	20458179	C	T	439	0.022779043	21	8.3916702	1.81241389	3.65E-06	NA
rs4131286	6	148688963	G	T	439	0.006833713	6	14.4284672	3.09172532	3.06E-06	NA
rs917335559	6	148703901	C	T	439	0.006833713	6	14.7509213	3.11419566	2.17E-06	NA
rs143686474	7	16291999	A	C	439	0.012528474	11	10.8531498	2.37513184	4.89E-06	NA
rs73202425	7	109155716	T	C	439	0.012528474	12	10.761844	2.30582166	3.05E-06	NA
rs181182636	7	115654558	A	G	439	0.003416856	3	21.9472639	4.69244743	2.91E-06	NA
rs182211730	7	116003615	G	A	439	0.004555809	4	18.7746033	3.8971981	1.45E-06	NA
rs143281973	7	116007996	C	T	439	0.004555809	4	18.2750778	3.81098198	1.62E-06	NA
rs138873576	7	116025359	G	T	439	0.004555809	4	17.6151711	3.78331079	3.22E-06	NA
rs190354334	7	116075846	G	A	439	0.004555809	5	17.3392581	3.7718663	4.29E-06	NA
rs186893139	7	116096970	C	T	439	0.004555809	4	18.602598	4.01258383	3.55E-06	NA
rs558784715	7	117043294	A	G	439	0.003416856	3	20.7591787	4.31689475	1.52E-06	NA
rs576047962	7	117109021	A	G	439	0.003416856	3	20.7261549	4.32140182	1.62E-06	NA
rs529110230	7	117175907	T	C	439	0.003416856	3	20.8583633	4.30235088	1.25E-06	NA
rs142215699	7	117199874	T	C	439	0.003416856	3	20.7292287	4.32245361	1.62E-06	NA

rs142721557	7	117218328	A	C	439	0.003416856	3	20.7329975	4.32245894	1.61E-06	NA
rs201355675	7	117225781	G	A	439	0.003416856	3	20.718124	4.32246722	1.64E-06	NA
rs188993522	7	117274731	T	C	439	0.003416856	3	21.2984827	4.47867266	1.98E-06	NA
rs62515436	8	57141203	G	T	439	0.017084282	15	9.27545902	1.99804812	3.45E-06	NA
rs144138711	9	99922419	T	C	439	0.045558087	40	6.08547131	1.26091918	1.39E-06	NA
rs556274646	11	439012	C	T	439	0.010250569	9	12.8914592	2.70847728	1.94E-06	NA
rs148815783	11	1013992	C	T	439	0.007972665	8	13.5980313	2.8647171	2.07E-06	NA
rs566724618	13	23463839	C	T	439	0.003416856	3	22.3433705	4.58061605	1.07E-06	NA
rs147221953	13	23485722	G	A	439	0.003416856	3	22.5809676	4.7360336	1.86E-06	NA
rs113791989	13	61918888	G	A	439	0.002277904	3	25.2049582	5.08402789	7.13E-07	NA
rs532269430	13	74054452	T	C	439	0.004555809	5	18.6173752	3.7963004	9.39E-07	NA
rs368187808	13	74357508	A	C	439	0.003416856	3	21.2991251	4.41501637	1.41E-06	NA
rs372194899	13	74357510	A	G	439	0.003416856	3	21.3012101	4.41516106	1.40E-06	NA
rs190251199	14	105590577	T	C	439	0.003416856	3	22.6385061	4.76043817	1.98E-06	NA
rs187281112	15	33113387	C	T	439	0.005694761	5	14.7343356	3.16793649	3.30E-06	NA
rs56324718	15	40280073	G	A	439	0.010250569	9	12.5117374	2.5288888	7.52E-07	NA
rs80212581	16	6412967	C	T	439	0.003416856	3	22.2762003	4.47237333	6.33E-07	NA
rs140276610	16	6433735	C	T	439	0.003416856	4	22.1806577	4.64062401	1.76E-06	NA
rs138164904	16	6858239	C	T	439	0.003416856	3	23.892641	4.3369669	3.61E-08	NA
rs116897913	17	16839162	C	T	439	0.003416856	3	20.1396765	4.40294288	4.78E-06	NA
rs188353596	17	46078602	C	T	439	0.003416856	3	21.8057947	4.39141941	6.85E-07	NA
rs112148840	17	66233774	C	T	439	0.002277904	3	24.0241075	5.1994476	3.83E-06	NA
rs147669485	18	26603650	G	T	439	0.003416856	4	22.1107639	4.14923198	9.88E-08	NA
rs559152067	18	66029612	G	A	439	0.002277904	3	24.7443692	5.18343862	1.81E-06	NA
rs367732718	19	35918004	G	A	439	0.002277904	3	23.8784923	5.13995752	3.39E-06	NA
rs562831582	19	51234310	A	C	439	0.003416856	4	21.0856548	4.21624838	5.70E-07	NA
rs547186621	20	6106741	A	G	439	0.003416856	4	21.5510829	4.5690881	2.40E-06	NA
rs138733283	20	30675687	C	T	439	0.004555809	5	18.2744744	3.65830138	5.87E-07	NA
rs149859280	20	30678170	C	T	439	0.004555809	5	18.3057707	3.66075889	5.72E-07	NA
rs146249289	20	30682231	C	T	439	0.004555809	4	19.6109527	3.77306035	2.02E-07	NA
rs145791959	20	30722429	G	A	439	0.005694761	5	17.9091953	3.64034112	8.67E-07	NA
rs193041547	20	30771880	T	C	439	0.004555809	4	19.6791358	3.77081146	1.80E-07	NA
rs138055631	20	30780644	G	A	439	0.005694761	5	17.6647762	3.62004915	1.06E-06	NA
rs145421321	20	30862126	C	T	439	0.005694761	6	17.0212611	3.55829648	1.72E-06	NA
rs143432612	20	30892775	C	T	439	0.005694761	6	16.9387232	3.54318154	1.75E-06	NA
rs200198574	20	30946654	G	A	439	0.005694761	6	16.5282735	3.50702674	2.44E-06	NA
rs148157126	20	30948839	C	T	439	0.004555809	5	19.7865001	3.76042103	1.43E-07	NA
rs192855100	20	31108741	G	A	439	0.004555809	4	20.1986097	3.99111726	4.17E-07	NA
rs386818616	21	41308948	A	G	439	0.887243736	112	-4.4521096	0.85237138	1.76E-07	NA

Supplementary Table S7. Independent Replication

Chennai-1 cohort, case-control data, comparing to discovery 3M quantitative trait (QT) data

Threshold for replicative significance:	5.00E-02
Total SNPs found:	122
Total risk loci found:	38

Gray shading: SNPs and target genes of genome-wide significance in the discovery cohort
NA: not applicable

rsid	chr	pos_37	REF	ALT	n.obs	caf	MAC	Est	Est.SE	Score.pval	pval.chennai	adj pval SNPs	adj pval loci	Closest gene	Prioritized gene
rs17017794	4	91825885	T	C	421	0.0391924	33	5.83240545	1.14450838	3.47E-07	6.39E-02	1.00E+00	1.00E+00	CCSER1	CCSER1
rs75334617	10	102956152	G	A	421	0.03800475	32	5.84603405	1.18027345	7.30E-07	6.39E-02	1.00E+00	1.00E+00	LINC01514	KAZALD1
rs76098744	1	112349372	C	T	421	0.01662708	14	8.38612452	1.72704116	1.20E-06	6.78E-02	1.00E+00	1.00E+00	KCND3	KCND3
rs74683551	1	112354418	G	A	421	0.01662708	14	8.38252043	1.72705812	1.21E-06	6.78E-02	1.00E+00	1.00E+00	KCND3	KCND3
rs17510814	12	28468969	A	C	421	0.00712589	6	14.0387924	2.71409689	2.31E-07	6.78E-02	1.00E+00	1.00E+00	CCDC91	CCDC91
rs141756120	12	28511096	A	C	421	0.00831354	6	14.278403	2.76458538	2.41E-07	6.78E-02	1.00E+00	1.00E+00	CCDC91	CCDC91
rs143371352	13	47383834	C	T	421	0.00356295	3	20.5341625	3.87928964	1.20E-07	6.78E-02	1.00E+00	1.00E+00	HTR2A	HTR2A
rs9643828	8	55529073	C	T	421	0.67695962	279	-2.4236294	0.46846911	2.30E-07	8.50E-02	1.00E+00	1.00E+00	RP1	SOX17
rs147601511	8	22322319	A	G	421	0.00475059	4	16.4471181	3.31777038	7.15E-07	9.08E-02	1.00E+00	1.00E+00	PPP3CC	PPP3CC
rs112007361	11	99188380	A	C	421	0.03087886	26	6.35658468	1.22825758	2.28E-07	9.08E-02	1.00E+00	1.00E+00	CNTN5	CNTN5
rs76327548	12	101182966	G	A	421	0.01306413	11	9.17842714	1.93235231	2.04E-06	1.13E-01	1.00E+00	1.00E+00	ANO4	ANO4
rs10105693	8	55640472	C	T	421	0.32897862	276	2.2928391	0.44965282	3.41E-07	1.28E-01	1.00E+00	1.00E+00	RP1	SOX17
rs11987234	8	55669829	A	G	421	0.32897862	276	2.29599259	0.44884532	3.13E-07	1.28E-01	1.00E+00	1.00E+00	RP1	SOX17
rs7822082	8	55690220	T	C	421	0.33016627	277	2.3570878	0.44879289	1.50E-07	1.28E-01	1.00E+00	1.00E+00	RP1	SOX17
rs149421869	2	53483429	G	T	421	0.00831354	8	11.7382186	2.47204376	2.05E-06	1.38E-01	1.00E+00	1.00E+00	ASB3	ASB3
rs17776100	7	6426479	G	A	421	0.02969121	25	6.63697397	1.32177185	5.13E-07	1.38E-01	1.00E+00	1.00E+00	RAC1	RAC1
rs2274997	1	229804646	A	G	421	0.0415677	35	5.58731228	1.1238603	6.64E-07	1.44E-01	1.00E+00	1.00E+00	URB2	URB2
rs74521112	11	99089147	G	T	421	0.03206651	27	6.25632387	1.22787586	3.48E-07	1.69E-01	1.00E+00	1.00E+00	CNTN5	CNTN5
rs79213709	11	99093455	G	A	421	0.03206651	26	6.17081884	1.23453654	5.78E-07	1.69E-01	1.00E+00	1.00E+00	CNTN5	CNTN5
rs1498183	8	55716905	C	T	421	0.39429929	332	2.0245746	0.44255141	4.77E-06	1.83E-01	1.00E+00	1.00E+00	RP1	SOX17
rs858397	8	55614690	A	G	421	0.33135392	278	2.2301348	0.44990341	7.16E-07	1.85E-01	1.00E+00	1.00E+00	RP1	SOX17
rs13278605	8	55688171	C	T	421	0.32897862	277	2.27377348	0.44593165	3.42E-07	1.85E-01	1.00E+00	1.00E+00	RP1	SOX17
rs13276543	8	55688174	G	T	421	0.32897862	276	2.29263157	0.44823093	3.14E-07	1.85E-01	1.00E+00	1.00E+00	RP1	SOX17
rs2375219	8	55698295	C	T	421	0.39311164	330	2.05700167	0.44073069	3.05E-06	1.85E-01	1.00E+00	1.00E+00	RP1	SOX17
rs12502861	4	2426305	T	C	421	0.01068884	9	11.1588472	2.36961887	2.49E-06	1.98E-01	1.00E+00	1.00E+00	CFAP99	CFAP99
rs2274996	1	229804538	C	T	421	0.0415677	35	5.58527433	1.12406363	6.74E-07	2.43E-01	1.00E+00	1.00E+00	URB2	URB2
rs2891865	1	229806368	A	G	421	0.0415677	35	5.58181376	1.12378916	6.80E-07	2.43E-01	1.00E+00	1.00E+00	URB2	URB2
rs2385790	1	229807492	C	T	421	0.0415677	35	5.58224822	1.12394723	6.81E-07	2.43E-01	1.00E+00	1.00E+00	URB2	URB2
rs12024557	1	229812357	A	C	421	0.04275534	35	5.56930969	1.12195729	6.91E-07	2.43E-01	1.00E+00	1.00E+00	URB2	URB2
rs4562666	1	229824770	T	C	421	0.04275534	36	5.51922599	1.11457457	7.35E-07	2.43E-01	1.00E+00	1.00E+00	URB2	URB2
rs12036586	1	229826378	G	A	421	0.04750594	40	5.22212176	1.07114093	1.09E-06	2.43E-01	1.00E+00	1.00E+00	URB2	URB2
rs16850124	1	229831331	T	C	421	0.04394299	37	5.18095662	1.09435188	2.20E-06	2.43E-01	1.00E+00	1.00E+00	URB2	URB2
rs12045643	1	229834050	C	T	421	0.04275534	36	5.48149152	1.10708948	7.37E-07	2.43E-01	1.00E+00	1.00E+00	URB2	URB2
rs2375536	8	55640722	T	C	421	0.347981	292	2.12635806	0.44857091	2.13E-06	2.58E-01	1.00E+00	1.00E+00	RP1	SOX17
rs4737674	8	55661654	C	A	421	0.33016627	277	2.30559554	0.44921988	2.86E-07	2.58E-01	1.00E+00	1.00E+00	RP1	SOX17
rs13277510	8	55674149	G	A	421	0.33016627	277	2.30494752	0.44909942	2.86E-07	2.58E-01	1.00E+00	1.00E+00	RP1	SOX17
rs12548593	8	55674617	G	T	421	0.33254157	279	2.28269961	0.44752402	3.38E-07	2.58E-01	1.00E+00	1.00E+00	RP1	SOX17
rs1812506	8	55676101	A	G	421	0.3456057	291	2.12258868	0.4466178	2.01E-06	2.58E-01	1.00E+00	1.00E+00	RP1	SOX17
rs16920698	8	55678434	G	A	421	0.33016627	277	2.30492695	0.44908177	2.86E-07	2.58E-01	1.00E+00	1.00E+00	RP1	SOX17
rs1561297	8	55678538	A	C	421	0.33254157	279	2.28315237	0.44743146	3.35E-07	2.58E-01	1.00E+00	1.00E+00	RP1	SOX17
rs4737676	8	55679546	G	A	421	0.33016627	277	2.30492057	0.44908192	2.86E-07	2.58E-01	1.00E+00	1.00E+00	RP1	SOX17
rs2083123	8	55680318	C	T	421	0.33254157	279	2.27910794	0.44707491	3.44E-07	2.58E-01	1.00E+00	1.00E+00	RP1	SOX17
rs983248	8	55680792	C	T	421	0.33016627	277	2.30260428	0.44871461	2.87E-07	2.58E-01	1.00E+00	1.00E+00	RP1	SOX17

rs1391463	8	55681876	T	G	421	0.33016627	277	2.30258865	0.44871307	2.87E-07	2.58E-01	1.00E+00	1.00E+00	RP1	SOX17
rs4737201	8	55691458	C	T	421	0.33016627	277	2.30197734	0.44869179	2.89E-07	2.58E-01	1.00E+00	1.00E+00	RP1	SOX17
rs7843693	8	55692112	G	A	421	0.39786223	334	1.99856756	0.43695394	4.79E-06	2.58E-01	1.00E+00	1.00E+00	RP1	SOX17
rs1396896	8	55695310	A	G	421	0.39786223	334	1.99853327	0.43690452	4.78E-06	2.58E-01	1.00E+00	1.00E+00	RP1	SOX17
rs1391462	8	55699781	C	A	421	0.39786223	334	1.9984766	0.43696027	4.79E-06	2.58E-01	1.00E+00	1.00E+00	RP1	SOX17
rs12678939	8	55705021	A	G	421	0.39429929	331	2.01975618	0.44220051	4.94E-06	2.58E-01	1.00E+00	1.00E+00	RP1	SOX17
rs12266995	10	24852783	T	C	421	0.03087886	26	5.96782535	1.29311959	3.93E-06	2.85E-01	1.00E+00	1.00E+00	ARHGAP21	ARHGAP21
rs149421869	2	52563371	C	T	421	0.00356295	3	19.7446169	3.72634921	1.17E-07	2.96E-01	1.00E+00	1.00E+00	ASB3	ASB3
rs187520610	2	53360041	G	A	421	0.00475059	4	18.8010396	3.19364595	3.93E-09	2.96E-01	1.00E+00	1.00E+00	ASB3	ASB3
rs116651654	4	163238743	C	T	421	0.00712589	6	15.6223694	3.06624451	3.49E-07	2.96E-01	1.00E+00	1.00E+00	FSTL5	FSTL5
rs290120	5	163268244	T	G	421	0.00712589	6	13.3587764	2.83260801	2.40E-06	2.96E-01	1.00E+00	1.00E+00	MAT2B	MAT2B
rs73586304	6	142839425	C	T	421	0.00356295	3	18.2405116	3.99428202	4.96E-06	2.96E-01	1.00E+00	1.00E+00	VTA1	VTA1
rs181259864	7	97488823	C	A	421	0.00356295	3	17.1360821	3.68242789	3.26E-06	2.96E-01	1.00E+00	1.00E+00	CZ1P-ASNS	ASNS
rs192750513	7	97577830	A	G	421	0.00356295	3	17.1892469	3.72514218	3.94E-06	2.96E-01	1.00E+00	1.00E+00	CZ1P-ASNS	ASNS
rs151115079	11	18655741	T	C	421	0.00475059	5	17.8273587	3.2680205	4.89E-08	2.96E-01	1.00E+00	1.00E+00	SPTY2D1	SPTY2D1
rs138414342	11	18679398	G	A	421	0.00475059	5	18.1582505	3.28214943	3.16E-08	2.96E-01	1.00E+00	1.00E+00	SPTY2D1	SPTY2D1
rs151323346	12	21012024	T	C	421	0.00593824	4	15.8903888	3.38034868	2.59E-06	2.96E-01	1.00E+00	1.00E+00	PDE3A	PDE3A
rs75186966	13	47395758	A	C	421	0.00356295	3	20.2281345	3.83225523	1.30E-07	2.96E-01	1.00E+00	1.00E+00	HTR2A	HTR2A
rs146442492	15	58982115	C	T	421	0.02731591	24	7.05925858	1.51661316	3.25E-06	2.96E-01	1.00E+00	1.00E+00	ADAM10	ADAM10
rs2327968	20	15813491	C	T	421	0.02494062	21	6.15899856	1.34390809	4.59E-06	2.96E-01	1.00E+00	1.00E+00	MACROD2	MACROD2
rs2876414	20	15813704	G	T	421	0.02256532	19	6.81910907	1.48530907	4.41E-06	2.96E-01	1.00E+00	1.00E+00	MACROD2	MACROD2
rs140788628	20	15858501	C	A	421	0.01068884	8	11.8039284	2.2826396	2.33E-07	2.96E-01	1.00E+00	1.00E+00	MACROD2	MACROD2
rs2365739	1	62484462	G	A	421	0.02137767	18	6.86815917	1.43887996	1.81E-06	3.34E-01	1.00E+00	1.00E+00	PATJ	PATJ
rs1856085	1	104114545	G	A	421	0.0023753	3	21.51144	4.26747898	4.64E-07	3.34E-01	1.00E+00	1.00E+00	AMY2B	AMY2B
rs143597860	1	104157143	A	G	421	0.0023753	3	21.5748154	4.2760379	4.52E-07	3.34E-01	1.00E+00	1.00E+00	AMY2B	AMY2B
rs144541665	1	104310729	G	A	421	0.0023753	3	21.8734611	4.30339839	3.72E-07	3.34E-01	1.00E+00	1.00E+00	AMY2B	AMY2B
rs113651406	4	990967	C	T	421	0.00356295	3	19.4054026	3.7635826	2.52E-07	3.34E-01	1.00E+00	1.00E+00	IDUA	IDUA
rs140797780	8	22087792	C	T	421	0.00356295	3	19.2908396	3.69147785	1.73E-07	3.34E-01	1.00E+00	1.00E+00	PHYHIP	PHYHIP
rs76904423	12	101188744	G	A	421	0.01068884	10	9.80576231	2.11568908	3.57E-06	3.34E-01	1.00E+00	1.00E+00	ANO4	ANO4
rs150586237	6	24491348	C	T	421	0.00356295	3	22.0461443	3.78094793	5.51E-09	3.46E-01	1.00E+00	1.00E+00	GPLD1	GPLD1
rs423841	8	55556069	G	A	421	0.66270784	291	-2.1495726	0.45846846	2.75E-06	3.49E-01	1.00E+00	1.00E+00	RP1	SOX17
rs433324	8	55564609	A	G	421	0.66627078	287	-2.2483462	0.45814252	9.22E-07	3.49E-01	1.00E+00	1.00E+00	RP1	SOX17
rs369623	8	55571940	A	C	421	0.66627078	287	-2.2359815	0.45415557	8.51E-07	3.49E-01	1.00E+00	1.00E+00	RP1	SOX17
rs446222	8	55574960	G	A	421	0.66627078	288	-2.247819	0.45397995	7.37E-07	3.49E-01	1.00E+00	1.00E+00	RP1	SOX17
rs432393	8	55580298	C	T	421	0.66270784	291	-2.1875382	0.45096346	1.23E-06	3.49E-01	1.00E+00	1.00E+00	RP1	SOX17
rs3098298	8	55582838	C	T	421	0.66270784	291	-2.1875635	0.45094791	1.23E-06	3.49E-01	1.00E+00	1.00E+00	RP1	SOX17
rs367179	8	55587616	T	C	421	0.66270784	291	-2.1875635	0.45094791	1.23E-06	3.49E-01	1.00E+00	1.00E+00	RP1	SOX17
rs382476	8	55590975	G	A	421	0.66627078	288	-2.2481938	0.4540554	7.37E-07	3.49E-01	1.00E+00	1.00E+00	RP1	SOX17
rs384543	8	55591609	G	A	421	0.66627078	288	-2.2481938	0.4540554	7.37E-07	3.49E-01	1.00E+00	1.00E+00	RP1	SOX17
rs405226	8	55592336	A	G	421	0.66270784	291	-2.1881305	0.45101874	1.23E-06	3.49E-01	1.00E+00	1.00E+00	RP1	SOX17
rs384127	8	55597489	G	A	421	0.66627078	288	-2.2481559	0.45404811	7.37E-07	3.49E-01	1.00E+00	1.00E+00	RP1	SOX17
rs2375537	8	55619508	C	T	421	0.33254157	280	2.23876848	0.44938849	6.30E-07	3.49E-01	1.00E+00	1.00E+00	RP1	SOX17
rs720372	8	55628637	G	A	421	0.34679335	292	2.09872734	0.44886259	2.93E-06	3.49E-01	1.00E+00	1.00E+00	RP1	SOX17
rs1437781	8	55629852	T	C	421	0.33254157	280	2.23854673	0.44940766	6.32E-07	3.49E-01	1.00E+00	1.00E+00	RP1	SOX17
rs1595406	8	55630615	A	G	421	0.3456057	291	2.10018889	0.44830064	2.80E-06	3.49E-01	1.00E+00	1.00E+00	RP1	SOX17
rs1437782	8	55632762	C	T	421	0.33016627	278	2.2579585	0.45098781	5.54E-07	3.49E-01	1.00E+00	1.00E+00	RP1	SOX17
rs10958428	8	55685641	A	G	421	0.33372922	280	2.23996885	0.44568673	5.01E-07	3.49E-01	1.00E+00	1.00E+00	RP1	SOX17
rs75689761	7	18406573	C	T	421	0.00831354	7	11.8482917	2.41320258	9.12E-07	4.27E-01	1.00E+00	1.00E+00	HDAC9	HDAC9
rs77346868	7	18406599	A	G	421	0.00950119	8	11.0210129	2.26481172	1.14E-06	4.27E-01	1.00E+00	1.00E+00	HDAC9	HDAC9
rs78225611	7	18407464	A	C	421	0.00950119	8	11.0294387	2.26622466	1.13E-06	4.27E-01	1.00E+00	1.00E+00	HDAC9	HDAC9
rs77300464	7	18408761	A	G	421	0.00712589	6	14.6659794	2.6169516	2.09E-08	4.27E-01	1.00E+00	1.00E+00	HDAC9	HDAC9
rs79602997	7	18410250	G	A	421	0.00831354	7	11.8608211	2.41901626	9.43E-07	4.27E-01	1.00E+00	1.00E+00	HDAC9	HDAC9

rs75773869	7	18410845	G	T	421	0.00831354	7	11.8642716	2.41902322	9.36E-07	4.27E-01	1.00E+00	1.00E+00	HDAC9	HDAC9
rs75606013	7	18414613	G	A	421	0.00712589	6	14.6617119	2.61692421	2.11E-08	4.27E-01	1.00E+00	1.00E+00	HDAC9	HDAC9
rs61434999	7	18418351	A	G	421	0.00831354	7	11.8411511	2.41284027	9.22E-07	4.27E-01	1.00E+00	1.00E+00	HDAC9	HDAC9
rs78907958	7	18425017	T	G	421	0.00831354	7	12.0901322	2.40514961	4.99E-07	4.27E-01	1.00E+00	1.00E+00	HDAC9	HDAC9
rs76526501	7	18431110	G	A	421	0.00712589	6	14.8397312	2.5778114	8.58E-09	4.27E-01	1.00E+00	1.00E+00	HDAC9	HDAC9
rs74455595	7	18431784	A	G	421	0.00712589	6	14.8397312	2.5778114	8.58E-09	4.27E-01	1.00E+00	1.00E+00	HDAC9	HDAC9
rs79182806	7	18433827	T	C	421	0.00831354	7	12.0972603	2.38956889	4.14E-07	4.27E-01	1.00E+00	1.00E+00	HDAC9	HDAC9
rs10279777	7	18441589	G	A	421	0.00950119	8	12.8681335	2.27961694	1.65E-08	4.27E-01	1.00E+00	1.00E+00	HDAC9	HDAC9
rs77867199	7	18442275	G	T	421	0.01068884	9	10.8221282	2.13363365	3.93E-07	4.27E-01	1.00E+00	1.00E+00	HDAC9	HDAC9
rs80156375	7	18443215	A	C	421	0.00950119	8	11.5406255	2.25971308	3.27E-07	4.27E-01	1.00E+00	1.00E+00	HDAC9	HDAC9
rs17169602	7	18446741	G	A	421	0.00950119	8	12.7273717	2.25993984	1.78E-08	4.27E-01	1.00E+00	1.00E+00	HDAC9	HDAC9
rs10486295	7	18446807	G	A	421	0.00950119	8	12.76229	2.26480456	1.75E-08	4.27E-01	1.00E+00	1.00E+00	HDAC9	HDAC9
rs75090694	7	18447436	A	G	421	0.00831354	7	13.8314005	2.41427081	1.01E-08	4.27E-01	1.00E+00	1.00E+00	HDAC9	HDAC9
rs12315614	12	64920957	C	A	421	0.0760095	64	3.77710662	0.8201959	4.12E-06	5.64E-01	1.00E+00	1.00E+00	TBK1	TBK1
rs147630370	4	87450675	T	C	421	0.00475059	4	16.0013633	3.23339786	7.47E-07	6.04E-01	1.00E+00	1.00E+00	MAPK10	MAPK10
rs142993106	4	90957372	G	A	421	0.01781473	15	8.08463611	1.73306162	3.09E-06	6.04E-01	1.00E+00	1.00E+00	CCSER1	CCSER1
rs146526206	4	90993018	T	C	421	0.01781473	15	8.09176656	1.69535286	1.82E-06	6.04E-01	1.00E+00	1.00E+00	CCSER1	CCSER1
rs139493286	18	28816019	G	A	421	0.00356295	3	17.4603093	3.75076428	3.24E-06	7.11E-01	1.00E+00	1.00E+00	DSG1	DSG1
rs1686289	14	46260982	G	A	421	0.67814727	277	-2.2551539	0.48625976	3.52E-06	8.30E-01	1.00E+00	1.00E+00	LINC02303	MIS18BP1
rs176786	14	46282970	T	C	421	0.32304038	268	2.28475641	0.49171936	3.38E-06	8.30E-01	1.00E+00	1.00E+00	LINC02303	MIS18BP1
rs79539453	11	125266353	C	T	421	0.0023753	3	20.8220616	4.34542406	1.65E-06	9.23E-01	1.00E+00	1.00E+00	PKNOX2	PKNOX2
rs78547898	22	32824278	G	A	421	0.00356295	3	20.9098395	4.15538274	4.85E-07	9.27E-01	1.00E+00	1.00E+00	BPIFC	FOX07
rs7104959	11	129846126	C	T	421	0.00356295	3	17.9204611	3.84366851	3.13E-06	9.37E-01	1.00E+00	1.00E+00	PRDM10	PRDM10
rs10494861	1	205331874	G	A	421	0.00356295	3	19.1820498	4.0729134	2.48E-06	9.56E-01	1.00E+00	1.00E+00	KLHDC8A	KLHDC8A
rs1877768	6	16534923	C	T	421	0.01781473	15	8.28754685	1.76830748	2.78E-06	9.56E-01	1.00E+00	1.00E+00	ATXN1	ATXN1
rs111391231	7	89497045	T	C	421	0.01425178	12	8.31717755	1.80720714	4.18E-06	9.56E-01	1.00E+00	1.00E+00	STEAP2	STEAP2
rs111900874	7	89516669	G	A	421	0.01425178	12	8.42424013	1.82258334	3.80E-06	9.56E-01	1.00E+00	1.00E+00	STEAP2	STEAP2
rs115348382	1	9655903	G	A	421	0.00356295	3	18.2107495	3.84721698	2.21E-06	NA				
rs186532456	1	18948328	C	T	421	0.00356295	3	20.1730343	4.11737745	9.61E-07	NA				
rs562032622	1	18959333	A	C	421	0.00356295	3	21.0267115	4.3197503	1.13E-06	NA				
rs149493615	1	79880089	G	A	421	0.00831354	8	11.750632	2.43451244	1.39E-06	NA				
rs143811231	1	79968131	T	C	421	0.00831354	8	11.5747409	2.42066027	1.74E-06	NA				
rs34270375	1	89370702	G	A	421	0.02612827	21	7.71147017	1.57216792	9.34E-07	NA				
rs187518659	1	99455745	T	G	421	0.00950119	8	11.6819623	2.46564537	2.16E-06	NA				
rs140420703	1	102803484	T	G	421	0.00475059	5	16.487654	3.46213891	1.91E-06	NA				
rs563167766	1	102866365	G	A	421	0.0023753	3	19.415739	4.24240395	4.73E-06	NA				
rs77180278	1	102961882	T	C	421	0.02850356	24	7.33619996	1.37404112	9.34E-08	NA				
rs112351653	1	103220360	T	C	421	0.02850356	24	7.42386947	1.33286772	2.55E-08	NA				
rs180926150	1	103226326	C	T	421	0.0023753	3	19.641918	4.24793649	3.77E-06	NA				
rs114413507	1	103419168	T	C	421	0.02850356	24	7.42216029	1.33294954	2.57E-08	NA				
rs116672066	1	103472916	G	A	421	0.02731591	23	8.04769476	1.37187844	4.46E-09	NA				
rs111928960	1	103633635	G	A	421	0.02850356	22	8.60257208	1.45827516	3.65E-09	NA				
rs113221952	1	103753974	A	G	421	0.02137767	19	7.92350704	1.61421682	9.17E-07	NA				
rs76617932	1	180930424	T	C	421	0.01187648	9	10.5449608	2.25040726	2.79E-06	NA				
rs183180157	1	181247121	A	C	421	0.00950119	7	12.61386	2.68921121	2.72E-06	NA				
rs375790303	1	184530482	G	A	421	0.00831354	7	11.4240393	2.49719192	4.77E-06	NA				
rs138480898	1	184955657	C	T	421	0.00356295	3	16.9873974	3.66972373	3.67E-06	NA				
rs145766563	1	185129502	G	A	421	0.00356295	4	16.8682102	3.54127008	1.90E-06	NA				
rs147032554	1	186148864	T	G	421	0.00356295	3	18.162609	3.65402821	6.68E-07	NA				
rs180989936	1	193044178	A	G	421	0.00475059	4	17.1793579	3.70215494	3.48E-06	NA				
rs559559983	1	246977132	C	A	421	0.00712589	5	13.811132	2.89725588	1.87E-06	NA				
rs550763536	2	7452347	T	G	421	0.00593824	5	14.4871647	3.10349768	3.04E-06	NA				

rs558553658	2	15682724	C	T	421	0.00475059	3	17.1099107	3.29108061	2.01E-07	NA
rs536781978	2	29733681	A	G	421	0.00356295	3	17.5870824	3.70259451	2.03E-06	NA
rs568321148	2	29870348	T	G	421	0.00356295	3	17.7125046	3.7201251	1.92E-06	NA
rs76777840	2	48312950	G	A	421	0.00593824	5	14.3500784	2.8927894	7.03E-07	NA
rs145080832	2	48473143	G	A	421	0.00475059	4	17.1618477	3.25709537	1.37E-07	NA
rs184220112	2	48631743	C	A	421	0.00475059	4	16.4879043	3.22267606	3.12E-07	NA
rs189890455	2	48655397	C	T	421	0.00593824	5	15.1467391	2.99355083	4.20E-07	NA
rs181193202	2	52527354	T	C	421	0.00356295	3	19.6312088	3.74776015	1.62E-07	NA
rs190193113	2	53085778	G	A	421	0.00356295	3	19.9533602	3.7619559	1.13E-07	NA
rs146479102	2	65825759	G	A	421	0.00593824	5	15.4846768	3.26980199	2.18E-06	NA
rs528288879	2	65875930	C	T	421	0.00593824	6	14.7878085	2.95121929	5.42E-07	NA
rs11690187	2	67565909	A	C	421	0.00593824	5	13.6310532	2.9751322	4.61E-06	NA
rs151272830	2	67682724	G	T	421	0.00712589	6	13.3323479	2.84806076	2.85E-06	NA
rs186142189	2	67702707	G	A	421	0.00712589	6	12.7361927	2.71405368	2.70E-06	NA
rs184200893	2	69260913	C	T	421	0.00356295	3	19.9580356	4.21905487	2.24E-06	NA
rs111927235	2	74483954	A	G	421	0.00475059	4	17.0468955	3.46111889	8.43E-07	NA
rs111838310	2	74673491	C	A	421	0.00475059	5	17.7963938	3.48429066	3.26E-07	NA
rs112983626	2	74697150	G	A	421	0.00475059	4	17.7848079	3.48532494	3.35E-07	NA
rs113006316	2	74802360	A	G	421	0.0023753	3	22.8886792	4.92411335	3.35E-06	NA
rs17746486	2	95722609	C	T	421	0.03206651	29	6.90851358	1.43477175	1.47E-06	NA
rs76554191	2	95967628	G	A	421	0.04038005	34	5.72148039	1.2389174	3.87E-06	NA
rs140352232	2	108038112	G	A	421	0.0023753	3	21.0576511	4.56013129	3.88E-06	NA
rs116189766	2	126393864	T	C	421	0.0023753	3	21.190764	4.36037289	1.17E-06	NA
rs139877408	2	129606273	A	G	421	0.0023753	3	20.3067129	4.43052497	4.58E-06	NA
rs142894171	2	151438271	G	T	421	0.00356295	3	18.0710394	3.74586836	1.41E-06	NA
rs541508507	2	170023265	G	A	421	0.00475059	4	15.4630033	3.27389618	2.32E-06	NA
rs142549310	2	170030506	C	T	421	0.00475059	4	15.2501713	3.24983634	2.70E-06	NA
rs556293455	2	176950420	G	A	421	0.00356295	3	14.3860463	2.96998402	1.27E-06	NA
rs184098071	2	177116420	G	A	421	0.00356295	3	13.947724	2.86782452	1.15E-06	NA
rs532416695	2	177486790	G	A	421	0.00356295	3	19.8726656	3.76737683	1.33E-07	NA
rs112557251	2	188375400	T	C	421	0.0023753	3	20.6510064	4.44850106	3.45E-06	NA
rs185158855	2	223650026	C	A	421	0.00475059	4	16.6678864	3.60266524	3.72E-06	NA
rs185510569	2	223814861	G	A	421	0.00356295	3	19.5886906	3.60490977	5.51E-08	NA
rs147559909	2	237051523	T	C	421	0.00593824	5	17.0840975	3.03209195	1.76E-08	NA
rs181217257	2	239989119	C	T	421	0.00475059	4	21.5677368	3.47296989	5.29E-10	NA
rs188076929	2	239993719	T	C	421	0.00475059	4	19.9775614	3.27246746	1.03E-09	NA
rs112475378	3	1626661	T	C	421	0.01662708	14	9.55085929	1.97628138	1.35E-06	NA
rs145676540	3	2044635	C	T	421	0.00593824	5	13.980658	2.88447377	1.25E-06	NA
rs146007933	3	28227423	T	C	421	0.02137767	18	7.51149453	1.55442805	1.35E-06	NA
rs73057656	3	33940571	A	G	421	0.0368171	33	5.9238329	1.27589711	3.44E-06	NA
rs73085348	3	42711221	A	G	421	0.01187648	11	10.139317	2.03504571	6.28E-07	NA
rs142684595	3	55319301	T	C	421	0.00593824	5	16.7729431	3.11364755	7.17E-08	NA
rs80203220	3	122722331	C	T	421	0.00593824	6	13.426042	2.84218656	2.31E-06	NA
rs192443987	3	135532345	G	A	421	0.00475059	4	17.7540285	3.67564302	1.36E-06	NA
rs148248743	3	136134595	C	T	421	0.0023753	3	22.839379	4.62044689	7.69E-07	NA
rs576124203	3	141841196	T	G	421	0.00356295	3	19.4039925	3.84392582	4.47E-07	NA
rs545552231	3	141864350	C	T	421	0.00356295	3	19.8641332	3.94582563	4.80E-07	NA
rs193153124	3	148330710	A	G	421	0.00593824	5	14.3657313	3.06514594	2.78E-06	NA
rs188720948	3	150069880	T	C	421	0.00356295	3	17.7032653	3.59722874	8.59E-07	NA
rs16823323	3	153657202	G	A	421	0.01662708	14	9.48464065	1.71769795	3.36E-08	NA
rs139943877	3	155451289	G	A	421	0.00712589	7	13.0535902	2.76041594	2.26E-06	NA
rs187047882	3	164285621	G	A	421	0.00356295	3	18.5641361	3.99725417	3.41E-06	NA

rs141169929	3	164808462	A	G	421	0.00475059	5	14.8454178	3.1738839	2.91E-06	NA
rs186649043	3	174932293	C	T	421	0.00356295	3	19.2134209	4.06588786	2.30E-06	NA
rs189709453	3	177574308	G	A	421	0.00356295	3	21.6581488	4.05916142	9.52E-08	NA
rs186767531	3	177639777	T	C	421	0.00356295	3	21.2013243	4.04780758	1.63E-07	NA
rs182868205	3	177712448	C	T	421	0.00356295	3	20.9548768	3.97841839	1.39E-07	NA
rs191792521	3	195646605	G	A	421	0.00831354	6	13.9515484	2.96443599	2.52E-06	NA
rs189765693	4	4324793	T	C	421	0.00475059	4	15.5191341	3.29924607	2.55E-06	NA
rs183962155	4	21259643	A	C	421	0.00593824	6	13.7646008	2.97247759	3.64E-06	NA
rs113751774	4	23428854	C	T	421	0.00712589	7	14.9103274	2.96657459	5.01E-07	NA
rs113063005	4	23509067	T	C	421	0.00593824	6	21.2073894	3.15520096	1.80E-11	NA
rs145875128	4	32073136	G	A	421	0.00356295	3	24.479986	4.60639213	1.07E-07	NA
rs143287889	4	35572280	C	T	421	0.0023753	3	19.96741	4.34406	4.30E-06	NA
rs77141817	4	37053759	T	C	421	0.00356295	3	20.6110292	3.64214866	1.52E-08	NA
rs190822761	4	37099356	G	T	421	0.00356295	3	20.5846354	3.64346559	1.61E-08	NA
rs999769259	4	62511965	G	A	421	0.00356295	3	22.1246725	4.77597881	3.61E-06	NA
rs147171192	4	89135588	A	G	421	0.00712589	5	13.3044047	2.89758492	4.40E-06	NA
rs145116559	4	112677596	T	C	421	0.00356295	4	18.2441224	3.9932536	4.91E-06	NA
rs181415102	4	112689266	T	C	421	0.00356295	4	18.2631019	3.99387944	4.81E-06	NA
rs191423619	4	126516627	G	T	421	0.0023753	3	21.694611	4.20667337	2.51E-07	NA
rs149298750	4	127158701	A	C	421	0.00356295	3	22.184052	4.26573246	1.99E-07	NA
rs112679237	4	139140860	T	C	421	0.01781473	18	8.71202752	1.67680671	2.04E-07	NA
rs531769270	4	154411043	T	C	421	0.00356295	3	16.5945338	3.51936092	2.41E-06	NA
rs567982164	5	25631297	G	A	421	0.0023753	3	21.8356286	4.36706703	5.73E-07	NA
rs191986449	5	25745687	C	T	421	0.00475059	3	18.4978793	4.03679831	4.60E-06	NA
rs185771987	5	73285489	T	C	421	0.00356295	3	18.2344824	3.97675014	4.53E-06	NA
rs139360368	5	73372109	A	C	421	0.00356295	4	18.3590487	3.78701272	1.25E-06	NA
rs181933850	5	91465647	A	G	421	0.00831354	7	11.0436586	2.37544216	3.33E-06	NA
rs190190051	5	91475485	G	A	421	0.00831354	7	11.0589887	2.38317975	3.48E-06	NA
rs182531466	5	91530073	C	A	421	0.00712589	6	12.6804978	2.75685261	4.23E-06	NA
rs187236873	5	91530447	G	A	421	0.00712589	6	12.44907	2.61780937	1.98E-06	NA
rs183816745	5	91631888	A	G	421	0.00593824	5	17.3487909	3.20195556	6.02E-08	NA
rs111407636	5	95080029	C	T	421	0.00356295	3	18.642774	3.74168052	6.28E-07	NA
rs111676272	5	95094298	C	A	421	0.00356295	3	18.6068625	3.74429748	6.72E-07	NA
rs75848314	5	95098340	T	C	421	0.00356295	3	20.0998481	3.78700451	1.11E-07	NA
rs111846247	5	95111643	T	C	421	0.00356295	3	20.4865772	3.85467205	1.07E-07	NA
rs137873790	5	97087041	A	G	421	0.01306413	11	9.22240881	2.01095379	4.52E-06	NA
rs189912648	5	134765439	C	T	421	0.00356295	4	16.9732599	3.60846227	2.55E-06	NA
rs191006910	5	154216009	G	A	421	0.00356295	3	17.6173803	3.79049689	3.36E-06	NA
rs74343174	5	161493182	C	A	421	0.00593824	5	14.7671824	3.13913408	2.55E-06	NA
rs775626702	5	162630067	A	C	421	0.00356295	3	18.9484137	4.07023372	3.23E-06	NA
rs371245624	5	162880956	T	C	421	0.00356295	3	19.4795931	4.06572011	1.66E-06	NA
rs545428520	5	167821266	T	C	421	0.00356295	4	21.566035	3.66407988	3.96E-09	NA
rs528404963	5	167852025	T	C	421	0.00356295	4	21.1393977	3.65303517	7.17E-09	NA
rs72832764	5	170004673	G	A	421	0.00356295	3	18.2159757	3.88196743	2.70E-06	NA
rs72837643	5	170188955	T	C	421	0.00356295	4	16.9302874	3.67743479	4.15E-06	NA
rs142311947	5	177384469	G	A	421	0.00712589	8	12.0339013	2.57618116	2.99E-06	NA
rs151015676	5	177390937	T	G	421	0.0023753	3	20.1421367	4.37074482	4.06E-06	NA
rs571986619	6	85678832	A	G	421	0.0023753	3	20.7858067	4.34301983	1.70E-06	NA
rs56224400	6	98092675	T	C	421	0.01662708	14	8.4433064	1.72251471	9.50E-07	NA
rs147627638	6	99173116	A	G	421	0.00712589	6	13.0785306	2.79364254	2.85E-06	NA
rs141326851	6	134833127	A	C	421	0.01662708	14	8.57849688	1.81053268	2.16E-06	NA
rs146048121	6	142198955	G	A	421	0.0023753	3	20.0214379	4.30086025	3.24E-06	NA

rs142106992	6	142269885	C	A	421	0.00356295	4	22.8345886	3.6758699	5.23E-10	NA
rs72983831	6	142307100	T	G	421	0.00593824	6	15.6733239	3.17294911	7.83E-07	NA
rs72986533	6	142611258	T	C	421	0.00831354	8	13.2812726	2.53063629	1.54E-07	NA
rs148532212	6	165499857	T	C	421	0.00356295	3	19.3535457	3.70945693	1.81E-07	NA
rs117498042	6	165514281	C	T	421	0.00356295	3	19.335471	3.70913521	1.86E-07	NA
rs148153037	6	167501386	G	A	421	0.00831354	8	14.3359246	2.42128102	3.20E-09	NA
rs184487573	6	167513471	A	G	421	0.00712589	7	13.5009635	2.59268014	1.92E-07	NA
rs187978759	7	11711845	G	A	421	0.00356295	3	20.4555522	4.3637719	2.76E-06	NA
rs117166500	7	17052778	G	T	421	0.00712589	7	12.5146865	2.62694198	1.90E-06	NA
rs55844051	7	23360363	T	C	421	0.00356295	3	18.3875896	3.86268704	1.93E-06	NA
rs1301444047	7	36574504	G	A	421	0.04275534	43	5.12842209	1.08835549	2.45E-06	NA
rs62447184	7	36574504	G	A	421	0.04275534	43	5.12842209	1.08835549	2.45E-06	NA
rs574076561	7	49544747	A	G	421	0.00356295	3	19.6434766	4.25225723	3.85E-06	NA
rs539713344	7	100474786	G	A	421	0.0023753	3	20.044756	4.21957545	2.03E-06	NA
rs188028357	7	100668425	C	T	421	0.0023753	3	21.8425578	4.35791809	5.38E-07	NA
rs536023430	7	146865072	T	C	421	0.00356295	3	19.4038683	4.13084856	2.64E-06	NA
rs187384541	8	1632241	A	G	421	0.03562945	26	6.88745117	1.47776668	3.15E-06	NA
rs575473987	8	5577494	C	T	421	0.00356295	3	22.2248653	3.88625754	1.07E-08	NA
rs139062456	8	13251991	C	T	421	0.01781473	15	7.83515809	1.69687423	3.89E-06	NA
rs185874707	8	18030674	C	T	421	0.01187648	10	9.13934765	1.94918644	2.75E-06	NA
rs188415494	8	25613298	C	T	421	0.00356295	3	18.5169478	3.9914865	3.50E-06	NA
rs1221830047	8	55690220	T	C	421	0.33016627	277	2.3570878	0.44879289	1.50E-07	NA
rs117816016	8	103751262	C	T	421	0.00356295	3	19.8807023	4.26960212	3.22E-06	NA
rs567383525	8	115029494	C	T	421	0.00356295	3	17.7455227	3.71970627	1.84E-06	NA
rs545550279	8	115552429	G	T	421	0.00356295	3	19.1812421	4.19212988	4.75E-06	NA
rs536803366	8	123001411	T	C	421	0.0023753	3	19.2098639	4.00952057	1.66E-06	NA
rs555249476	8	123001411	T	C	421	0.0023753	3	19.2098639	4.00952057	1.66E-06	NA
rs532513136	8	135813748	C	A	421	0.00356295	3	19.9490594	4.03118599	7.47E-07	NA
rs532730683	9	1784492	G	T	421	0.00356295	3	18.726838	3.8314675	1.02E-06	NA
rs540065886	9	2770228	T	C	421	0.00356295	3	19.9489151	4.18802805	1.90E-06	NA
rs543844012	9	30107023	C	T	421	0.00356295	3	18.1160485	3.9446103	4.38E-06	NA
rs148556485	9	84023826	A	C	421	0.0023753	3	21.3800152	4.26618316	5.40E-07	NA
rs140782222	9	84028894	T	C	421	0.0023753	3	21.791526	4.31928461	4.53E-07	NA
rs188034471	9	85937714	G	A	421	0.00475059	4	14.7559999	3.14925113	2.79E-06	NA
rs190294315	9	85945465	C	T	421	0.00593824	5	14.5664276	2.88126649	4.29E-07	NA
rs545690161	9	93030699	G	A	421	0.00593824	6	17.1436874	3.00318779	1.14E-08	NA
rs565682685	9	93222328	T	C	421	0.00475059	4	18.938397	3.7206174	3.58E-07	NA
rs183737367	9	93330047	T	C	421	0.00356295	3	23.3452306	4.24471431	3.80E-08	NA
rs187213609	9	93415465	C	T	421	0.00356295	3	23.1975304	4.27412656	5.72E-08	NA
rs150027952	9	103385416	A	G	421	0.0023753	3	21.8616648	4.51464643	1.28E-06	NA
rs146207930	9	129042336	A	G	421	0.00712589	6	11.8624321	2.51502465	2.40E-06	NA
rs78296164	9	135266715	C	T	421	0.00831354	7	11.6095338	2.33917557	6.94E-07	NA
rs77871739	9	138552309	G	A	421	0.00475059	4	15.0724603	3.23606181	3.20E-06	NA
rs118040657	10	3472846	C	T	421	0.00831354	8	12.1147712	2.64434694	4.62E-06	NA
rs184425183	10	13457520	A	G	421	0.00356295	3	22.6683174	3.77549648	1.92E-09	NA
rs184458518	10	13471195	T	G	421	0.00356295	3	22.8015789	3.77123942	1.48E-09	NA
rs117998251	10	13497976	C	T	421	0.00356295	3	22.9543311	3.77480488	1.19E-09	NA
rs117025967	10	20207052	C	A	421	0.01306413	11	9.66973563	1.927439	5.25E-07	NA
rs529011661	10	20372316	G	A	421	0.00475059	4	15.6393469	3.3125339	2.34E-06	NA
rs138249376	10	63515829	T	G	421	0.00475059	4	16.4300711	3.59466791	4.86E-06	NA
rs140277951	10	82359100	G	A	421	0.00831354	6	13.7463158	2.64333932	1.99E-07	NA
rs566018180	10	86755052	C	T	421	0.00356295	3	18.563552	4.04215862	4.38E-06	NA

rs140706881	10	96217535	G	A	421	0.00475059	4	18.974805	3.93288931	1.40E-06	NA
rs117913371	10	102918486	G	A	421	0.02494062	21	7.86609555	1.40785512	2.31E-08	NA
rs752259256	10	104925319	T	C	421	0.00356295	3	20.4258639	4.45411591	4.52E-06	NA
rs180828621	10	124533409	G	A	421	0.00712589	6	15.1380574	2.79957627	6.40E-08	NA
rs147393020	10	124779274	A	G	421	0.00593824	5	14.1853505	2.95980896	1.65E-06	NA
rs193093906	10	126705489	G	A	421	0.00950119	9	10.5219537	2.25840517	3.18E-06	NA
rs541653703	11	18701786	G	A	421	0.00475059	4	18.3169499	3.27243418	2.18E-08	NA
rs118093638	11	18718324	C	T	421	0.00593824	5	14.2657259	2.91296506	9.72E-07	NA
rs181812512	11	66665729	C	T	421	0.00356295	3	19.5093168	4.24219816	4.25E-06	NA
rs529345909	11	67110852	A	G	421	0.00356295	3	18.70153	4.09206035	4.87E-06	NA
rs544042801	11	68460243	G	A	421	0.00475059	4	15.8869866	3.40519019	3.08E-06	NA
rs149949098	11	95099866	G	A	421	0.01662708	13	8.55927625	1.81883308	2.53E-06	NA
rs148781275	11	103640603	A	G	421	0.00475059	5	15.7111853	3.32183832	2.25E-06	NA
rs141281289	11	123693024	A	G	421	0.00593824	6	15.811518	3.0889522	3.08E-07	NA
rs528140343	11	125719227	A	C	421	0.00356295	3	18.2835521	3.72738029	9.33E-07	NA
rs546409459	11	125754989	A	G	421	0.00356295	3	18.9195474	4.00286836	2.28E-06	NA
rs528609331	11	125842195	C	T	421	0.00356295	3	19.5812418	3.94832705	7.07E-07	NA
rs189360484	12	1870510	A	G	421	0.00475059	4	16.8078794	3.31424855	3.95E-07	NA
rs141754456	12	20151132	T	C	421	0.00712589	7	12.1460333	2.32842921	1.82E-07	NA
rs118184666	12	20424749	G	A	421	0.00712589	6	10.9509803	2.32477242	2.47E-06	NA
rs549931083	12	20516286	A	C	421	0.00356295	3	14.3282694	2.79891395	3.07E-07	NA
rs371879555	12	23015962	T	C	421	0.00356295	3	20.1033765	4.20002323	1.70E-06	NA
rs183466664	12	26821687	A	G	421	0.00475059	4	16.0181882	3.45609016	3.57E-06	NA
rs77353774	12	28248852	G	A	421	0.00712589	6	13.1054362	2.67954103	1.00E-06	NA
rs113167689	12	28435962	C	T	421	0.00712589	6	14.1364135	2.73335629	2.32E-07	NA
rs117991215	12	28511473	T	C	421	0.00831354	6	14.333666	2.77918736	2.50E-07	NA
rs191930622	12	48284655	G	A	421	0.0023753	3	19.6783579	4.30610368	4.88E-06	NA
rs56302696	12	48292830	G	A	421	0.0023753	3	19.8959041	4.31584707	4.03E-06	NA
rs185620578	12	48569399	C	T	421	0.0023753	3	20.7232267	4.39505442	2.42E-06	NA
rs190806532	12	48862147	G	T	421	0.0023753	3	21.326955	4.43856043	1.55E-06	NA
rs568658857	12	49853998	G	A	421	0.00593824	5	14.2361193	3.09423422	4.21E-06	NA
rs137880949	12	63306297	T	C	421	0.00475059	3	21.6084034	4.13589168	1.75E-07	NA
rs191053292	12	63445280	T	C	421	0.00356295	3	22.9266488	4.11439732	2.51E-08	NA
rs182437250	12	63608466	T	C	421	0.00475059	4	18.8083451	3.79343554	7.12E-07	NA
rs180764936	12	101498621	T	C	421	0.00356295	3	19.3473053	4.0200238	1.49E-06	NA
rs185855183	12	101505126	C	T	421	0.00356295	3	18.7397727	3.86137343	1.22E-06	NA
rs139598422	13	23887014	A	G	421	0.00356295	4	20.4886522	3.64167194	1.84E-08	NA
rs150077525	13	57979549	A	G	421	0.00712589	7	12.9984875	2.71312414	1.66E-06	NA
rs534845494	13	58213864	A	G	421	0.00475059	5	15.9431765	3.18484007	5.56E-07	NA
rs140062526	13	59286033	G	A	421	0.00593824	5	14.0926088	3.02052903	3.08E-06	NA
rs546286713	13	91411079	G	A	421	0.00475059	4	15.7027841	3.3611216	2.98E-06	NA
rs567080482	13	95075405	T	C	421	0.00475059	4	15.4999561	3.31620837	2.95E-06	NA
rs142928734	13	101601082	G	A	421	0.00356295	3	17.009593	3.70044989	4.29E-06	NA
rs556680896	13	101602415	C	T	421	0.00356295	3	17.0254659	3.70286799	4.27E-06	NA
rs184265355	13	108011352	A	C	421	0.00593824	6	14.4271719	2.91829792	7.67E-07	NA
rs572961122	13	108012985	C	T	421	0.00593824	6	13.3354896	2.85827607	3.08E-06	NA
rs528809914	13	113041256	G	A	421	0.00356295	3	19.5048552	3.76867111	2.27E-07	NA
rs180765647	13	114427311	G	T	421	0.0023753	3	20.1246521	4.37397806	4.20E-06	NA
rs138215817	14	22641516	A	G	421	0.00475059	4	16.311019	3.27367022	6.28E-07	NA
rs74704551	14	30161887	C	T	421	0.00356295	3	24.1990705	4.31064693	1.98E-08	NA
rs176783	14	46280913	A	G	421	0.32185273	267	2.28010147	0.49376374	3.88E-06	NA
rs143048774	14	46294660	A	C	421	0.31710214	265	2.28482299	0.49630858	4.15E-06	NA

rs428110	14	46294660	A	C	421	0.31710214	265	2.28482299	0.49630858	4.15E-06	NA
rs116862847	14	64141677	C	T	421	0.00712589	7	15.3160369	2.95194262	2.12E-07	NA
rs569916471	14	75891342	G	A	421	0.00475059	5	15.6404088	3.3537133	3.11E-06	NA
rs113767990	14	81717563	G	A	421	0.00475059	4	14.5811155	3.19363095	4.98E-06	NA
rs190251199	14	105590577	T	C	421	0.00356295	3	20.0125292	4.01627173	6.27E-07	NA
rs185155853	15	41244100	C	T	421	0.00475059	5	15.9164657	3.40838631	3.02E-06	NA
rs144026361	15	41248669	C	T	421	0.00475059	5	16.1372994	3.43734659	2.67E-06	NA
rs558614420	15	41810870	C	T	421	0.00593824	6	13.9750831	3.02772732	3.92E-06	NA
rs138109686	15	42051442	A	G	421	0.00593824	6	13.5956705	2.96761718	4.62E-06	NA
rs145896760	15	42119222	G	A	421	0.00593824	6	13.3688825	2.92346809	4.81E-06	NA
rs140642138	15	42125165	G	A	421	0.00593824	6	13.5832221	2.96274049	4.55E-06	NA
rs6080	15	58837933	C	A	421	0.04394299	36	5.66476745	1.2393253	4.86E-06	NA
rs145439370	15	58879765	T	C	421	0.03087886	26	6.89328891	1.40339117	9.02E-07	NA
rs149425014	15	58951660	T	C	421	0.02612827	22	7.55224792	1.58320604	1.84E-06	NA
rs193253461	15	59229353	A	G	421	0.01306413	12	10.5374468	2.07404763	3.76E-07	NA
rs184117160	15	59404306	C	T	421	0.01425178	11	10.3417236	2.13366338	1.25E-06	NA
rs80292573	15	59435086	T	G	421	0.03444181	30	6.55323491	1.32696583	7.87E-07	NA
rs182303755	15	59634792	A	C	421	0.01306413	12	10.5479917	2.09359291	4.70E-07	NA
rs138217865	15	94392882	C	T	421	0.00475059	4	16.709641	3.45193896	1.29E-06	NA
rs553840536	16	25697895	A	G	421	0.00356295	3	19.6203442	4.23547954	3.61E-06	NA
rs183817723	16	59302775	C	T	421	0.00356295	4	17.5860507	3.7341919	2.48E-06	NA
rs144954214	16	76179362	A	G	421	0.0023753	3	21.8113263	4.43942529	8.96E-07	NA
rs529523094	16	77715551	A	G	421	0.00356295	3	18.1584346	3.72617419	1.10E-06	NA
rs146728064	17	19265440	G	A	421	0.00712589	6	12.6400411	2.73467115	3.80E-06	NA
rs184613584	17	48508221	A	C	421	0.00712589	6	14.1377974	2.8212234	5.41E-07	NA
rs191271637	17	52123260	A	G	421	0.00356295	3	18.379836	3.92083355	2.76E-06	NA
rs185819304	18	27001580	G	A	421	0.00356295	3	19.9401113	3.94501953	4.32E-07	NA
rs187942235	18	27030430	C	T	421	0.00356295	3	20.3968658	3.96963025	2.77E-07	NA
rs143538552	18	29050262	A	G	421	0.00356295	3	19.1457218	3.63679703	1.41E-07	NA
rs373746073	18	29058384	C	A	421	0.00356295	3	19.0847197	3.63798064	1.55E-07	NA
rs146333745	18	55497457	C	T	421	0.00356295	4	17.4215784	3.56782335	1.04E-06	NA
rs185464792	19	18797371	C	T	421	0.0023753	3	22.5896015	4.50191473	5.23E-07	NA
rs186768950	19	18806124	C	A	421	0.0023753	3	22.7152061	4.50776479	4.68E-07	NA
rs541288561	19	18869445	T	G	421	0.00475059	5	15.6119012	3.13183483	6.20E-07	NA
rs559008174	19	18876059	C	T	421	0.00475059	5	15.6025661	3.12812006	6.11E-07	NA
rs570407448	19	18880030	G	A	421	0.00475059	5	15.4977984	3.12412555	7.02E-07	NA
rs546144116	19	19563339	C	T	421	0.0023753	3	23.1354815	4.47162758	2.29E-07	NA
rs560206697	19	20729098	C	T	421	0.0023753	3	24.9989943	4.55350673	4.02E-08	NA
rs111285015	19	23123198	G	A	421	0.00356295	3	27.3347642	4.62083983	3.31E-09	NA
rs1008091735	19	31090099	T	C	421	0.00356295	3	18.4689243	3.69079909	5.61E-07	NA
rs148433854	19	31096478	G	A	421	0.00356295	3	18.6267585	3.68806754	4.41E-07	NA
rs559228693	20	15963329	G	A	421	0.00593824	5	14.3643551	3.08196497	3.15E-06	NA
rs557092705	20	34189911	C	T	421	0.00356295	3	19.4769546	4.2480073	4.54E-06	NA
rs184785969	21	17171431	C	A	421	0.00356295	3	19.8736486	3.70683937	8.26E-08	NA
rs117280553	21	17207163	T	C	421	0.00356295	3	20.087417	3.70274826	5.80E-08	NA
rs79486609	21	17245006	G	A	421	0.00356295	3	20.6624886	3.77824957	4.53E-08	NA
rs73227413	21	23136973	G	A	421	0.03444181	28	5.96205514	1.27150176	2.75E-06	NA
rs75024143	21	23156546	G	T	421	0.01425178	12	9.81254241	2.0684542	2.10E-06	NA
rs192134381	21	23450714	T	C	421	0.00356295	3	21.788812	3.73125357	5.23E-09	NA
rs397836601	21	23450714	T	C	421	0.00356295	3	21.788812	3.73125357	5.23E-09	NA
rs118183140	21	35477486	C	T	421	0.02019002	17	7.5893248	1.56122232	1.17E-06	NA
rs183586634	21	38763032	G	A	421	0.00593824	5	14.3132202	2.99210048	1.72E-06	NA

rs117185941	21	38766484	G	A	421	0.00593824	5	15.3808786	3.17143013	1.24E-06	NA
rs1329159859	21	38766484	G	A	421	0.00593824	5	15.3808786	3.17143013	1.24E-06	NA
rs118084887	21	38863820	T	C	421	0.00593824	5	14.9382616	3.13674741	1.91E-06	NA
rs150539922	21	43276916	T	C	421	0.00356295	3	16.6696937	3.64477381	4.79E-06	NA
rs113625788	22	19969182	C	T	421	0.00831354	7	11.8084829	2.42941561	1.17E-06	NA
rs796777817	22	32824278	G	A	421	0.00356295	3	20.9098395	4.15538274	4.85E-07	NA
rs541680196	22	40528090	G	A	421	0.00593824	5	14.3867808	2.94903659	1.07E-06	NA
rs185139807	22	40594781	G	A	421	0.00593824	5	14.4330526	2.95312686	1.02E-06	NA
rs141127122	22	40604439	G	A	421	0.00475059	4	15.3824875	3.30083549	3.16E-06	NA
rs148998974	22	40620530	A	G	421	0.00593824	5	14.5129532	2.94976024	8.65E-07	NA
rs555040883	22	40631476	G	A	421	0.00475059	4	15.4273069	3.30542717	3.05E-06	NA
rs182959028	22	45823032	T	C	421	0.00831354	7	11.944461	2.53146	2.38E-06	NA
rs150946694	22	46853180	T	C	421	0.00475059	4	15.3082479	3.29125059	3.30E-06	NA

Supplementary Table S7. Independent Replication

Chennai-2 cohort, case-control data, comparing to discovery 12M quantitative trait (QT) data

Threshold for replicative significance =	5.00E-02
Total SNPs found:	41
Total risk loci found:	22

Thick boxed: SNPs that attained adjusted p-values of replication significance
NA: not applicable

rsid	chr	pos_37	REF	ALT	n.obs	caf	MAC	Est	Est.SE	Score.pval	pval.chennai	adj pval SNPs	adj pval loci	Closest gene	Prioritized gene
rs2427460	20	61590782	T	C	439	0.48633257	420	-2.574044	0.51325892	5.30E-07	2.80E-04	1.15E-02	6.17E-03	SLC17A9	SLC17A9
rs9636964	21	41304765	A	G	439	0.88496583	115	-4.2543192	0.82744371	2.73E-07	3.07E-02	1.00E+00	6.76E-01	PCP4	PCP4
rs147944608	10	110423709	C	T	439	0.01252847	11	11.2205243	2.29229161	9.84E-07	5.91E-02	1.00E+00	1.00E+00	XPNPEP1	XPNPEP1
rs144138711	9	99922419	T	C	439	0.04555809	40	6.08547131	1.26091918	1.39E-06	5.95E-02	1.00E+00	1.00E+00	ANKRD18CP	CCDC180
rs117591241	3	190584679	A	G	439	0.00341686	4	19.9014028	4.33331389	4.38E-06	8.12E-02	1.00E+00	1.00E+00	GMNC	GMNC
rs1472243465	3	190584679	A	G	439	0.00341686	4	19.9014028	4.33331389	4.38E-06	8.12E-02	1.00E+00	1.00E+00	GMNC	GMNC
rs74572772	22	34492649	A	G	439	0.01822323	16	10.2225183	1.94126774	1.40E-07	1.09E-01	1.00E+00	1.00E+00	LARGE1	LARGE1
rs61918041	12	6681868	C	T	439	0.023918	21	7.97264643	1.73636056	4.40E-06	1.93E-01	1.00E+00	1.00E+00	CHD4	CHD4
rs56821264	6	148703901	C	T	439	0.00683371	6	14.7509213	3.11419566	2.17E-06	2.05E-01	1.00E+00	1.00E+00	SASH1	SASH1
rs917335559	6	148703901	C	T	439	0.00683371	6	14.7509213	3.11419566	2.17E-06	2.05E-01	1.00E+00	1.00E+00	SASH1	SASH1
rs1162141034	2	67825685	C	T	439	0.07972665	71	4.55991411	0.98044721	3.31E-06	2.13E-01	1.00E+00	1.00E+00	ETAA1	ETAA1
rs146465493	2	67825685	C	T	439	0.07972665	71	4.55991411	0.98044721	3.31E-06	2.13E-01	1.00E+00	1.00E+00	ETAA1	ETAA1
rs2902021	2	67825685	C	T	439	0.07972665	71	4.55991411	0.98044721	3.31E-06	2.13E-01	1.00E+00	1.00E+00	ETAA1	ETAA1
rs75082290	2	67831153	T	G	439	0.0785877	69	4.69395387	0.98072733	1.70E-06	2.58E-01	1.00E+00	1.00E+00	ETAA1	ETAA1
rs9974985	21	41307573	G	A	439	0.88610478	114	-4.3459395	0.82924282	1.60E-07	3.20E-01	1.00E+00	1.00E+00	PCP4	PCP4
rs7534177	1	65965720	A	G	439	0.05694761	49	4.96123378	1.0598616	2.85E-06	3.28E-01	1.00E+00	1.00E+00	LEPR	LEPR
rs4896997	6	148683924	C	T	439	0.00683371	6	14.4320923	3.09188376	3.05E-06	3.29E-01	1.00E+00	1.00E+00	SASH1	SASH1
rs62515405	8	57055978	G	A	439	0.023918	21	8.07691204	1.76428755	4.69E-06	3.29E-01	1.00E+00	1.00E+00	PLAG1	PLAG1
rs17078283	6	148696920	C	T	439	0.00683371	6	14.4302821	3.0918048	3.05E-06	3.42E-01	1.00E+00	1.00E+00	SASH1	SASH1
rs17543620	4	169934725	T	C	439	0.0501139	44	5.42796755	1.17982097	4.21E-06	3.47E-01	1.00E+00	1.00E+00	CBR4	CBR4
rs1005412	21	41308948	A	G	439	0.88724374	112	-4.4521096	0.85237138	1.76E-07	3.96E-01	1.00E+00	1.00E+00	PCP4	PCP4
rs386818616	21	41308948	A	G	439	0.88724374	112	-4.4521096	0.85237138	1.76E-07	3.96E-01	1.00E+00	1.00E+00	PCP4	PCP4
rs117699122	12	560262	T	C	439	0.00341686	4	21.4238256	4.40952328	1.18E-06	4.03E-01	1.00E+00	1.00E+00	CCDC77	CCDC77
rs9305683	21	41305720	G	A	439	0.88952164	112	-4.1163679	0.83634363	8.57E-07	4.08E-01	1.00E+00	1.00E+00	PCP4	PCP4
rs12114488	8	62672723	G	A	439	0.30523918	266	2.62307914	0.54257702	1.33E-06	4.11E-01	1.00E+00	1.00E+00	MIR4470	ADPH
rs139816293	20	30921343	C	T	439	0.00569476	6	17.1384248	3.52537223	1.17E-06	4.23E-01	1.00E+00	1.00E+00	KIF3B	KIF3B
rs74683551	1	112354418	G	A	439	0.01594533	14	9.54722832	2.04640679	3.08E-06	4.23E-01	1.00E+00	1.00E+00	KCND3	KCND3
rs76356799	3	179593768	G	A	439	0.00455581	4	19.2867007	3.76039319	2.91E-07	5.66E-01	1.00E+00	1.00E+00	PEX5L	PEX5L
rs143686474	7	16291999	A	C	439	0.01252847	11	10.8531498	2.37513184	4.89E-06	5.72E-01	1.00E+00	1.00E+00	CRPPA	SOSTDC1
rs62192733	20	1928157	A	G	439	0.00683371	7	15.744618	3.35866563	2.76E-06	5.76E-01	1.00E+00	1.00E+00	PDYN	SIRPA
rs147669485	18	26603650	G	T	439	0.00341686	4	22.1107639	4.14923198	9.88E-08	5.80E-01	1.00E+00	1.00E+00	CDH2	CDH2
rs7275595	21	41307923	G	A	439	0.88838269	113	-4.2609754	0.83407868	3.25E-07	6.02E-01	1.00E+00	1.00E+00	PCP4	PCP4
rs2606194	17	77210823	A	G	439	0.94874715	44	-5.6956979	1.16435773	1.00E-06	6.65E-01	1.00E+00	1.00E+00	RBFOX3	RBFOX3
rs17127656	1	65943471	C	T	439	0.05580866	49	5.07046538	1.05780641	1.64E-06	6.74E-01	1.00E+00	1.00E+00	LEPR	LEPR
rs77297738	22	34473543	C	T	439	0.01822323	16	10.2480572	1.97167592	2.02E-07	6.78E-01	1.00E+00	1.00E+00	LARGE1	LARGE1
rs17615362	4	169934087	G	A	439	0.0501139	44	5.41974472	1.17918425	4.30E-06	7.04E-01	1.00E+00	1.00E+00	CBR4	CBR4
rs7518849	1	65948791	T	C	439	0.05580866	49	5.07456956	1.05964469	1.68E-06	7.24E-01	1.00E+00	1.00E+00	LEPR	LEPR
rs9981433	21	41309565	G	T	439	0.88154897	116	-4.1220124	0.84715422	1.14E-06	8.07E-01	1.00E+00	1.00E+00	LEPR	LEPR
rs80019988	22	34493647	G	A	439	0.01822323	16	10.0623306	1.91665407	1.52E-07	8.18E-01	1.00E+00	1.00E+00	LARGE1	LARGE1
rs4131286	6	148688963	G	T	439	0.00683371	6	14.4284672	3.09172532	3.06E-06	9.26E-01	1.00E+00	1.00E+00	SASH1	SASH1
rs11579567	1	65957141	C	A	439	0.05580866	49	5.02973793	1.06097379	2.13E-06	9.78E-01	1.00E+00	1.00E+00	LEPR	LEPR
rs142934021	5	52662672	G	A	439	0.00797267	7	18.824715	3.31820294	1.40E-08	NA				
rs138164904	16	6858239	C	T	439	0.00341686	3	23.892641	4.3369669	3.61E-08	NA				

rs138138661	3	147657814	C	T	439	0.00455581	4	23.1347457	4.25695836	5.49E-08	NA
rs148997617	3	147755576	C	T	439	0.00455581	4	20.8774277	3.92648902	1.05E-07	NA
rs148157126	20	30948839	C	T	439	0.00455581	5	19.7865001	3.76042103	1.43E-07	NA
rs186792608	2	43096699	A	G	439	0.00341686	3	22.4512499	4.28172841	1.58E-07	NA
rs113154814	2	68289497	T	C	439	0.00569476	5	19.8310188	3.7892894	1.66E-07	NA
rs193041547	20	30771880	T	C	439	0.00455581	4	19.6791358	3.77081146	1.80E-07	NA
rs146249289	20	30682231	C	T	439	0.00455581	4	19.6109527	3.77306035	2.02E-07	NA
rs192855100	20	31108741	G	A	439	0.00455581	4	20.1986097	3.99111726	4.17E-07	NA
rs562831582	19	51234310	A	C	439	0.00341686	4	21.0856548	4.21624838	5.70E-07	NA
rs149859280	20	30678170	C	T	439	0.00455581	5	18.3057707	3.66075889	5.72E-07	NA
rs138733283	20	30675687	C	T	439	0.00455581	5	18.2744744	3.65830138	5.87E-07	NA
rs80212581	16	6412967	C	T	439	0.00341686	3	22.2762003	4.47237333	6.33E-07	NA
rs148248743	3	136134595	C	T	439	0.0022779	3	27.1548587	5.46352726	6.69E-07	NA
rs188353596	17	46078602	C	T	439	0.00341686	3	21.8057947	4.39141941	6.85E-07	NA
rs113791989	13	61918888	G	A	439	0.0022779	3	25.2049582	5.08402789	7.13E-07	NA
rs56324718	15	40280073	G	A	439	0.01025057	9	12.5117374	2.5288888	7.52E-07	NA
rs191298981	2	68110046	C	T	439	0.00455581	3	24.0078714	4.85703224	7.70E-07	NA
rs146919974	2	115252863	T	C	439	0.00455581	5	19.7280466	3.99554232	7.91E-07	NA
rs145791959	20	30722429	G	A	439	0.00569476	5	17.9091953	3.64034112	8.67E-07	NA
rs142956968	10	23490165	C	T	439	0.01594533	14	10.4167992	2.11909083	8.85E-07	NA
rs532269430	13	74054452	T	C	439	0.00455581	5	18.6173752	3.7963004	9.39E-07	NA
rs113063005	4	23509067	T	C	439	0.00683371	7	16.61763	3.39113209	9.57E-07	NA
rs113537164	2	68234930	A	C	439	0.00455581	4	20.3890413	4.17097432	1.02E-06	NA
rs138055631	20	30780644	G	A	439	0.00569476	5	17.6647762	3.62004915	1.06E-06	NA
rs566724618	13	23463839	C	T	439	0.00341686	3	22.3433705	4.58061605	1.07E-06	NA
rs145764464	10	23601847	G	A	439	0.01708428	15	9.5967306	1.96976348	1.10E-06	NA
rs529110230	7	117175907	T	C	439	0.00341686	3	20.8583633	4.30235088	1.25E-06	NA
rs182868205	3	177712448	C	T	439	0.00341686	3	22.8544303	4.73215662	1.37E-06	NA
rs372194899	13	74357510	A	G	439	0.00341686	3	21.3012101	4.41516106	1.40E-06	NA
rs368187808	13	74357508	A	C	439	0.00341686	3	21.2991251	4.41501637	1.41E-06	NA
rs145804766	1	202122499	C	T	439	0.01025057	9	12.5864465	2.61118962	1.43E-06	NA
rs182211730	7	116003615	G	A	439	0.00455581	4	18.7746033	3.8971981	1.45E-06	NA
rs558784715	7	117043294	A	G	439	0.00341686	3	20.7591787	4.31689475	1.52E-06	NA
rs189709453	3	177574308	G	A	439	0.00341686	3	23.2009936	4.82954374	1.56E-06	NA
rs142721557	7	117218328	A	C	439	0.00341686	3	20.7329975	4.32245894	1.61E-06	NA
rs576047962	7	117109021	A	G	439	0.00341686	3	20.7261549	4.32140182	1.62E-06	NA
rs142215699	7	117199874	T	C	439	0.00341686	3	20.7292287	4.32245361	1.62E-06	NA
rs143281973	7	116007996	C	T	439	0.00455581	4	18.2750778	3.81098198	1.62E-06	NA
rs201355675	7	117225781	G	A	439	0.00341686	3	20.718124	4.32246722	1.64E-06	NA
rs186767531	3	177639777	T	C	439	0.00341686	3	23.0749977	4.81664642	1.66E-06	NA
rs181132315	4	129647369	C	T	439	0.00341686	3	22.1324679	4.62266164	1.69E-06	NA
rs145421321	20	30862126	C	T	439	0.00569476	6	17.0212611	3.55829648	1.72E-06	NA
rs143432612	20	30892775	C	T	439	0.00569476	6	16.9387232	3.54318154	1.75E-06	NA
rs140276610	16	6433735	C	T	439	0.00341686	4	22.1806577	4.64062401	1.76E-06	NA
rs559152067	18	66029612	G	A	439	0.0022779	3	24.7443692	5.18343862	1.81E-06	NA
rs147221953	13	23485722	G	A	439	0.00341686	3	22.5809676	4.7360336	1.86E-06	NA
rs556274646	11	439012	C	T	439	0.01025057	9	12.8914592	2.70847728	1.94E-06	NA
rs190251199	14	105590577	T	C	439	0.00341686	3	22.6385061	4.76043817	1.98E-06	NA
rs188993522	7	117274731	T	C	439	0.00341686	3	21.2984827	4.47867266	1.98E-06	NA
rs568321148	2	29870348	T	G	439	0.00341686	3	20.7400203	4.36652717	2.04E-06	NA
rs148815783	11	1013992	C	T	439	0.00797267	8	13.5980313	2.8647171	2.07E-06	NA
rs76220567	2	13059945	T	C	439	0.00341686	4	20.6828868	4.35776216	2.07E-06	NA

rs35145334	1	23465122	A	G	439	0.01708428	14	10.6552656	2.24620792	2.10E-06	NA
rs114067899	1	85191044	G	A	439	0.00797267	7	13.7760673	2.90836365	2.17E-06	NA
rs536781978	2	29733681	A	G	439	0.00341686	3	20.5561882	4.34258099	2.21E-06	NA
rs147267707	4	43026558	G	A	439	0.00455581	4	18.0094385	3.80818408	2.25E-06	NA
rs77618729	4	43050968	T	C	439	0.00455581	4	18.0130153	3.80914173	2.26E-06	NA
rs547186621	20	6106741	A	G	439	0.00341686	4	21.5510829	4.5690881	2.40E-06	NA
rs200198574	20	30946654	G	A	439	0.00569476	6	16.5282735	3.50702674	2.44E-06	NA
rs532464521	4	43571039	T	C	439	0.00455581	4	17.9895528	3.81820438	2.46E-06	NA
rs79252854	3	157523157	C	T	439	0.00455581	5	17.5464336	3.72541417	2.48E-06	NA
rs79935606	2	177338635	T	C	439	0.00569476	5	16.3396833	3.47376974	2.55E-06	NA
rs556435089	4	43551033	G	A	439	0.00455581	4	17.939079	3.81772146	2.62E-06	NA
rs139390630	1	84866196	G	A	439	0.01138952	13	11.6975971	2.4994863	2.87E-06	NA
rs149706477	3	66148228	A	G	439	0.00341686	3	20.9828687	4.4842531	2.88E-06	NA
rs189094663	4	11623450	G	A	439	0.0022779	3	23.895416	5.10822708	2.90E-06	NA
rs181182636	7	115654558	A	G	439	0.00341686	3	21.9472639	4.69244743	2.91E-06	NA
rs76098744	1	112349372	C	T	439	0.01594533	14	9.5685657	2.04635193	2.93E-06	NA
rs73202425	7	109155716	T	C	439	0.01252847	12	10.761844	2.30582166	3.05E-06	NA
rs139055031	3	158586849	T	C	439	0.00569476	6	16.2853761	3.4955337	3.18E-06	NA
rs138873576	7	116025359	G	T	439	0.00455581	4	17.6151711	3.78331079	3.22E-06	NA
rs148483098	3	157382051	C	A	439	0.00569476	5	15.7357844	3.38211024	3.28E-06	NA
rs187281112	15	33113387	C	T	439	0.00569476	5	14.7343356	3.16793649	3.30E-06	NA
rs189912648	5	134765439	C	T	439	0.00341686	4	19.8604449	4.2716652	3.33E-06	NA
rs557276277	4	19013698	T	C	439	0.00569476	5	19.0146344	4.09137627	3.36E-06	NA
rs147869916	1	227307652	A	G	439	0.00569476	5	17.5964551	3.78625881	3.36E-06	NA
rs367732718	19	35918004	G	A	439	0.0022779	3	23.8784923	5.13995752	3.39E-06	NA
rs62515436	8	57141203	G	T	439	0.01708428	15	9.27545902	1.99804812	3.45E-06	NA
rs189234695	3	147658158	T	C	439	0.00341686	3	23.9697134	5.16542129	3.48E-06	NA
rs186893139	7	116096970	C	T	439	0.00455581	4	18.602598	4.01258383	3.55E-06	NA
rs73384619	6	20458179	C	T	439	0.02277904	21	8.3916702	1.81241389	3.65E-06	NA
rs114280794	3	132842203	G	A	439	0.01594533	14	8.76296877	1.89632016	3.82E-06	NA
rs112148840	17	66233774	C	T	439	0.0022779	3	24.0241075	5.1994476	3.83E-06	NA
rs147455971	4	140931712	T	C	439	0.00797267	5	17.2160142	3.72793057	3.87E-06	NA
rs144788248	3	19586745	T	C	439	0.01138952	10	11.9086237	2.57879124	3.88E-06	NA
rs111512950	5	153680427	C	T	439	0.00911162	8	13.567443	2.94598493	4.12E-06	NA
rs149616342	12	99178813	C	T	439	0.01138952	10	11.2978214	2.454657	4.17E-06	NA
rs190354334	7	116075846	G	A	439	0.00455581	5	17.3392581	3.7718663	4.29E-06	NA
rs113244573	12	6684385	G	A	439	0.023918	21	7.97095746	1.73766156	4.49E-06	NA
rs116897913	17	16839162	C	T	439	0.00341686	3	20.1396765	4.40294288	4.78E-06	NA
rs143669489	3	159392885	G	A	439	0.00341686	3	24.2253199	5.30400674	4.94E-06	NA

Supplementary Table S7. Independent Replication

Chennai-2 cohort, case-control data, comparing to discovery 3M quantitative trait (QT) data

Threshold for replicative significance:	5.00E-02
Total SNPs found:	123
Total risk loci found:	41

Gray shading: SNPs and target genes of genome-wide significance in the discovery cohort
Thick boxed: SNPs that attained adjusted p-values of replication significance
NA: not applicable

rsid	chr	pos_37	REF	ALT	n.obs	caf	MAC	Est	Est.SE	Score.pval	pval.chennai	adj pval SNPs	adj pval loci	Closest gene	Prioritized gene
rs7822082	8	55690220	T	C	421	0.33016627	277	2.3570878	0.44879289	1.50E-07	8.66E-06	1.07E-03	3.55E-04	RP1	SOX17
rs1561297	8	55678538	A	C	421	0.33254157	279	2.28315237	0.44743146	3.35E-07	7.41E-05	9.11E-03	3.04E-03	RP1	SOX17
rs193153124	3	148330710	A	G	421	0.00593824	5	14.3657313	3.06514594	2.78E-06	3.56E-04	4.38E-02	1.46E-02	AGTR1	AGTR1
rs12678939	8	55705021	A	G	421	0.39429929	331	2.01975618	0.44220051	4.94E-06	1.66E-03	2.04E-01	6.81E-02	RP1	SOX17
rs446222	8	55574960	G	A	421	0.66627078	288	-2.247819	0.45397995	7.37E-07	1.95E-03	2.40E-01	8.00E-02	RP1	SOX17
rs2375537	8	55619508	C	T	421	0.33254157	280	2.23876848	0.44938849	6.30E-07	3.33E-03	4.10E-01	1.37E-01	RP1	SOX17
rs2375536	8	55640722	T	C	421	0.347981	292	2.12635806	0.44857091	2.13E-06	3.38E-03	4.16E-01	1.39E-01	RP1	SOX17
rs1812506	8	55676101	A	G	421	0.3456057	291	2.12258868	0.4466178	2.01E-06	5.06E-03	6.22E-01	2.07E-01	RP1	SOX17
rs858397	8	55614690	A	G	421	0.33135392	278	2.2301348	0.44990341	7.16E-07	1.03E-02	1.00E+00	4.22E-01	RP1	SOX17
rs382476	8	55590975	G	A	421	0.66627078	288	-2.2481938	0.4540554	7.37E-07	1.07E-02	1.00E+00	4.39E-01	RP1	SOX17
rs1595406	8	55630615	A	G	421	0.3456057	291	2.10018889	0.44830064	2.80E-06	1.27E-02	1.00E+00	5.21E-01	RP1	SOX17
rs13278605	8	55688171	C	T	421	0.32897862	277	2.27377348	0.44593165	3.42E-07	1.37E-02	1.00E+00	5.62E-01	RP1	SOX17
rs13276543	8	55688174	G	T	421	0.32897862	276	2.29263157	0.44823093	3.14E-07	1.37E-02	1.00E+00	5.62E-01	RP1	SOX17
rs405226	8	55592336	A	G	421	0.66270784	291	-2.1881305	0.45101874	1.23E-06	1.41E-02	1.00E+00	5.78E-01	RP1	SOX17
rs1391462	8	55699781	C	A	421	0.39786223	334	1.9984766	0.43696027	4.79E-06	1.84E-02	1.00E+00	7.54E-01	RP1	SOX17
rs1437782	8	55632762	C	T	421	0.33016627	278	2.2579585	0.45098781	5.54E-07	1.98E-02	1.00E+00	8.12E-01	RP1	SOX17
rs2375219	8	55698295	C	T	421	0.39311164	330	2.05700167	0.44073069	3.05E-06	2.18E-02	1.00E+00	8.94E-01	RP1	SOX17
rs16920698	8	55678434	G	A	421	0.33016627	277	2.30492695	0.44908177	2.86E-07	2.28E-02	1.00E+00	9.35E-01	RP1	SOX17
rs4737676	8	55679546	G	A	421	0.33016627	277	2.30492057	0.44908192	2.86E-07	2.53E-02	1.00E+00	1.00E+00	RP1	SOX17
rs1396896	8	55695310	A	G	421	0.39786223	334	1.99853327	0.43690452	4.78E-06	2.66E-02	1.00E+00	1.00E+00	RP1	SOX17
rs9643828	8	55529073	C	T	421	0.67695962	279	-2.4236294	0.46846911	2.30E-07	2.99E-02	1.00E+00	1.00E+00	RP1	SOX17
rs1437781	8	55629852	T	C	421	0.33254157	280	2.23854673	0.44940766	6.32E-07	3.11E-02	1.00E+00	1.00E+00	RP1	SOX17
rs12548593	8	55674617	G	T	421	0.33254157	279	2.28269961	0.44752402	3.38E-07	3.14E-02	1.00E+00	1.00E+00	RP1	SOX17
rs2083123	8	55680318	C	T	421	0.33254157	279	2.27910794	0.44707491	3.44E-07	3.14E-02	1.00E+00	1.00E+00	RP1	SOX17
rs720372	8	55628637	G	A	421	0.34679335	292	2.09872734	0.44886259	2.93E-06	3.58E-02	1.00E+00	1.00E+00	RP1	SOX17
rs7843693	8	55692112	G	A	421	0.39786223	334	1.99856756	0.43695394	4.79E-06	3.58E-02	1.00E+00	1.00E+00	RP1	SOX17
rs13277510	8	55674149	G	A	421	0.33016627	277	2.30494752	0.44909942	2.86E-07	3.62E-02	1.00E+00	1.00E+00	RP1	SOX17
rs983248	8	55680792	C	T	421	0.33016627	277	2.30260428	0.44871461	2.87E-07	3.75E-02	1.00E+00	1.00E+00	RP1	SOX17
rs432393	8	55580298	C	T	421	0.66270784	291	-2.1875382	0.45096346	1.23E-06	4.10E-02	1.00E+00	1.00E+00	RP1	SOX17
rs12024557	1	229812357	A	C	421	0.04275534	35	5.56930969	1.12195729	6.91E-07	4.44E-02	1.00E+00	1.00E+00	URB2	URB2
rs16823323	3	153657202	G	A	421	0.01662708	14	9.48464065	1.71769795	3.36E-08	5.30E-02	1.00E+00	1.00E+00	ARHGEF26	ARHGEF26
rs79182806	7	18433827	T	C	421	0.00831354	7	12.0972603	2.38956889	4.14E-07	5.30E-02	1.00E+00	1.00E+00	HDAC9	HDAC9
rs369623	8	55571940	A	C	421	0.66627078	287	-2.2359815	0.45415557	8.51E-07	6.33E-02	1.00E+00	1.00E+00	RP1	SOX17
rs384127	8	55597489	G	A	421	0.66627078	288	-2.2481559	0.45404811	7.37E-07	6.35E-02	1.00E+00	1.00E+00	RP1	SOX17
rs184613584	17	48508221	A	C	421	0.00712589	6	14.1377974	2.8212234	5.41E-07	6.48E-02	1.00E+00	1.00E+00	ACSF2	ACSF2
rs182959028	22	45823032	T	C	421	0.00831354	7	11.944461	2.53146	2.38E-06	6.48E-02	1.00E+00	1.00E+00	RIBC2	RIBC2
rs433324	8	55564609	A	G	421	0.66627078	287	-2.2483462	0.45814252	9.22E-07	6.83E-02	1.00E+00	1.00E+00	RP1	SOX17
rs1301444047	7	36574504	G	A	421	0.04275534	43	5.12842209	1.08835549	2.45E-06	7.23E-02	1.00E+00	1.00E+00	AOAH	AOAH
rs62447184	7	36574504	G	A	421	0.04275534	43	5.12842209	1.08835549	2.45E-06	7.23E-02	1.00E+00	1.00E+00	AOAH	AOAH
rs541288561	19	18869445	T	G	421	0.00475059	5	15.6119012	3.13183483	6.20E-07	7.23E-02	1.00E+00	1.00E+00	CRTC1	CRTC1
rs570407448	19	18880030	G	A	421	0.00475059	5	15.4977984	3.12412555	7.02E-07	7.23E-02	1.00E+00	1.00E+00	CRTC1	CRTC1
rs423841	8	55556069	G	A	421	0.66270784	291	-2.1495726	0.45846846	2.75E-06	7.39E-02	1.00E+00	1.00E+00	RP1	SOX17
rs117913371	10	102918486	G	A	421	0.02494062	21	7.86609555	1.40785512	2.31E-08	7.46E-02	1.00E+00	1.00E+00	LINC01514	KAZALD1

rs147601511	8	22322319	A	G	421	0.00475059	4	16.4471181	3.31777038	7.15E-07	7.96E-02	1.00E+00	1.00E+00	PPP3CC	PPP3CC
rs1391463	8	55681876	T	G	421	0.33016627	277	2.30258865	0.44871307	2.87E-07	9.01E-02	1.00E+00	1.00E+00	RP1	SOX17
rs78225611	7	18407464	A	C	421	0.00950119	8	11.0294387	2.26622466	1.13E-06	1.05E-01	1.00E+00	1.00E+00	HDAC9	HDAC9
rs77300464	7	18408761	A	G	421	0.00712589	6	14.6659794	2.6169516	2.09E-08	1.10E-01	1.00E+00	1.00E+00	HDAC9	HDAC9
rs4737674	8	55661654	C	A	421	0.33016627	277	2.30559554	0.44921988	2.86E-07	1.16E-01	1.00E+00	1.00E+00	RP1	SOX17
rs11987234	8	55669829	A	G	421	0.32897862	276	2.29599259	0.44884532	3.13E-07	1.20E-01	1.00E+00	1.00E+00	RP1	SOX17
rs17169602	7	18446741	G	A	421	0.00950119	8	12.7273717	2.25993984	1.78E-08	1.25E-01	1.00E+00	1.00E+00	HDAC9	HDAC9
rs3098298	8	55582838	C	T	421	0.66270784	291	-2.1875635	0.45094791	1.23E-06	1.25E-01	1.00E+00	1.00E+00	RP1	SOX17
rs61434999	7	18418351	A	G	421	0.00831354	7	11.8411511	2.41284027	9.22E-07	1.28E-01	1.00E+00	1.00E+00	HDAC9	HDAC9
rs77346868	7	18406599	A	G	421	0.00950119	8	11.0210129	2.26481172	1.14E-06	1.31E-01	1.00E+00	1.00E+00	HDAC9	HDAC9
rs139493286	18	28816019	G	A	421	0.00356295	3	17.4603093	3.75076428	3.24E-06	1.33E-01	1.00E+00	1.00E+00	DSG1	DSG1
rs1877768	6	16534923	C	T	421	0.01781473	15	8.28754685	1.76830748	2.78E-06	1.60E-01	1.00E+00	1.00E+00	ATXN1	ATXN1
rs1686289	14	46260982	G	A	421	0.67814727	277	-2.2551539	0.48625976	3.52E-06	1.63E-01	1.00E+00	1.00E+00	LINC02303	MIS18BP1
rs150586237	6	24491348	C	T	421	0.00356295	3	22.0461443	3.78094793	5.51E-09	1.64E-01	1.00E+00	1.00E+00	GPLD1	GPLD1
rs10105693	8	55640472	C	T	421	0.32897862	276	2.2928391	0.44965282	3.41E-07	1.68E-01	1.00E+00	1.00E+00	RP1	SOX17
rs79539453	11	125266353	C	T	421	0.0023753	3	20.8220616	4.34542406	1.65E-06	1.73E-01	1.00E+00	1.00E+00	PKNOX2	PKNOX2
rs10279777	7	18441589	G	A	421	0.00950119	8	12.8681335	2.27961694	1.65E-08	1.97E-01	1.00E+00	1.00E+00	HDAC9	HDAC9
rs367179	8	55587616	T	C	421	0.66270784	291	-2.1875635	0.45094791	1.23E-06	2.03E-01	1.00E+00	1.00E+00	RP1	SOX17
rs384543	8	55591609	G	A	421	0.66627078	288	-2.2481938	0.4540554	7.37E-07	2.18E-01	1.00E+00	1.00E+00	RP1	SOX17
rs2385790	1	229807492	C	T	421	0.0415677	35	5.58224822	1.12394723	6.81E-07	2.21E-01	1.00E+00	1.00E+00	URB2	URB2
rs78907958	7	18425017	T	G	421	0.00831354	7	12.0901322	2.40514961	4.99E-07	2.36E-01	1.00E+00	1.00E+00	HDAC9	HDAC9
rs2274997	1	229804646	A	G	421	0.0415677	35	5.58731228	1.1238603	6.64E-07	2.43E-01	1.00E+00	1.00E+00	URB2	URB2
rs1498183	8	55716905	C	T	421	0.39429929	332	2.0245746	0.44255141	4.77E-06	2.53E-01	1.00E+00	1.00E+00	RP1	SOX17
rs10958428	8	55685641	A	G	421	0.33372922	280	2.23996885	0.44568673	5.01E-07	2.78E-01	1.00E+00	1.00E+00	RP1	SOX17
rs80156375	7	18443215	A	C	421	0.00950119	8	11.5406255	2.25971308	3.27E-07	2.83E-01	1.00E+00	1.00E+00	HDAC9	HDAC9
rs79213709	11	99093455	G	A	421	0.03206651	26	6.17081884	1.23453654	5.78E-07	2.83E-01	1.00E+00	1.00E+00	CNTN5	CNTN5
rs12502861	4	2426305	T	C	421	0.01068884	9	11.1588472	2.36961887	2.49E-06	3.29E-01	1.00E+00	1.00E+00	CFAP99	CFAP99
rs557092705	20	34189911	C	T	421	0.00356295	3	19.4769546	4.2480073	4.54E-06	3.29E-01	1.00E+00	1.00E+00	FER1L4	ERGIC3
rs75773869	7	18410845	G	T	421	0.00831354	7	11.8642716	2.41902322	9.36E-07	3.31E-01	1.00E+00	1.00E+00	HDAC9	HDAC9
rs74455595	7	18431784	A	G	421	0.00712589	6	14.8397312	2.5778114	8.58E-09	3.40E-01	1.00E+00	1.00E+00	HDAC9	HDAC9
rs79602997	7	18410250	G	A	421	0.00831354	7	11.8608211	2.41901626	9.43E-07	3.50E-01	1.00E+00	1.00E+00	HDAC9	HDAC9
rs75090694	7	18447436	A	G	421	0.00831354	7	13.8314005	2.41427081	1.01E-08	3.59E-01	1.00E+00	1.00E+00	HDAC9	HDAC9
rs4737201	8	55691458	C	T	421	0.33016627	277	2.30197734	0.44869179	2.89E-07	3.65E-01	1.00E+00	1.00E+00	RP1	SOX17
rs17746486	2	95722609	C	T	421	0.03206651	29	6.90851358	1.43477175	1.47E-06	3.80E-01	1.00E+00	1.00E+00	MAL	MAL
rs118040657	10	3472846	C	T	421	0.00831354	8	12.1147712	2.64434694	4.62E-06	3.80E-01	1.00E+00	1.00E+00	LOC10537636[illegible]	KLF6
rs113651406	4	990967	C	T	421	0.00356295	3	19.4054026	3.7635826	2.52E-07	3.88E-01	1.00E+00	1.00E+00	IDUA	IDUA
rs75689761	7	18406573	C	T	421	0.00831354	7	11.8482917	2.41320258	9.12E-07	3.91E-01	1.00E+00	1.00E+00	HDAC9	HDAC9
rs147171192	4	89135588	A	G	421	0.00712589	5	13.3044047	2.89758492	4.40E-06	4.15E-01	1.00E+00	1.00E+00	ABCG2	ABCG2
rs10486295	7	18446807	G	A	421	0.00950119	8	12.76229	2.26480456	1.75E-08	4.22E-01	1.00E+00	1.00E+00	HDAC9	HDAC9
rs74683551	1	112354418	G	A	421	0.01662708	14	8.38252043	1.72705812	1.21E-06	4.23E-01	1.00E+00	1.00E+00	KCND3	KCND3
rs147630370	4	87450675	T	C	421	0.00475059	4	16.0013633	3.23339786	7.47E-07	4.23E-01	1.00E+00	1.00E+00	MAPK10	MAPK10
rs111391231	7	89497045	T	C	421	0.01425178	12	8.31717755	1.80720714	4.18E-06	4.23E-01	1.00E+00	1.00E+00	STEAP2	STEAP2
rs112007361	11	99188380	A	C	421	0.03087886	26	6.35658468	1.22825758	2.28E-07	4.23E-01	1.00E+00	1.00E+00	CNTN5	CNTN5
rs10494861	1	205331874	G	A	421	0.00356295	3	19.1820498	4.0729134	2.48E-06	4.27E-01	1.00E+00	1.00E+00	KLHDC8A	KLHDC8A
rs142894171	2	151438271	G	T	421	0.00356295	3	18.0710394	3.74586836	1.41E-06	4.27E-01	1.00E+00	1.00E+00	LINC02612	RND3
rs176786	14	46282970	T	C	421	0.32304038	268	2.28475641	0.49171936	3.38E-06	4.32E-01	1.00E+00	1.00E+00	LINC02303	MIS18BP1
rs76327548	12	101182966	G	A	421	0.01306413	11	9.17842714	1.93235231	2.04E-06	4.39E-01	1.00E+00	1.00E+00	ANO4	ANO4
rs12045643	1	229834050	C	T	421	0.04275534	36	5.48149152	1.10708948	7.37E-07	4.45E-01	1.00E+00	1.00E+00	URB2	URB2
rs140782222	9	84028894	T	C	421	0.0023753	3	21.791526	4.31928461	4.53E-07	4.53E-01	1.00E+00	1.00E+00	TLE1	TLE1
rs12266995	10	24852783	T	C	421	0.03087886	26	5.96782535	1.29311959	3.93E-06	4.91E-01	1.00E+00	1.00E+00	ARHGAP21	ARHGAP21
rs176783	14	46280913	A	G	421	0.32185273	267	2.28010147	0.49376374	3.88E-06	5.10E-01	1.00E+00	1.00E+00	LINC02303	MIS18BP1
rs77867199	7	18442275	G	T	421	0.01068884	9	10.8221282	2.13363365	3.93E-07	5.14E-01	1.00E+00	1.00E+00	HDAC9	HDAC9

rs2891865	1	229806368	A	G	421	0.0415677	35	5.58181376	1.12378916	6.80E-07	5.18E-01	1.00E+00	1.00E+00	URB2	URB2
rs16850124	1	229831331	T	C	421	0.04394299	37	5.18095662	1.09435188	2.20E-06	5.34E-01	1.00E+00	1.00E+00	URB2	URB2
rs75606013	7	18414613	G	A	421	0.00712589	6	14.6617119	2.61692421	2.11E-08	5.47E-01	1.00E+00	1.00E+00	HDAC9	HDAC9
rs290120	5	163268244	T	G	421	0.00712589	6	13.3587764	2.83260801	2.40E-06	5.69E-01	1.00E+00	1.00E+00	MAT2B	MAT2B
rs73085348	3	42711221	A	G	421	0.01187648	11	10.139317	2.03504571	6.28E-07	5.76E-01	1.00E+00	1.00E+00	STEAP2	STEAP2
rs111900874	7	89516669	G	A	421	0.01425178	12	8.42424013	1.82258334	3.80E-06	5.76E-01	1.00E+00	1.00E+00	STEAP2	STEAP2
rs148556485	9	84023826	A	C	421	0.0023753	3	21.3800152	4.26618316	5.40E-07	5.76E-01	1.00E+00	1.00E+00	TLE1	TLE1
rs180765647	13	114427311	G	T	421	0.0023753	3	20.1246521	4.37397806	4.20E-06	5.76E-01	1.00E+00	1.00E+00	GRK1	ATP4B
rs2327968	20	15813491	C	T	421	0.02494062	21	6.15899856	1.34390809	4.59E-06	5.83E-01	1.00E+00	1.00E+00	MACROD2	MACROD2
rs2365739	1	62484462	G	A	421	0.02137767	18	6.86815917	1.43887996	1.81E-06	5.88E-01	1.00E+00	1.00E+00	PATJ	PATJ
rs142993106	4	90957372	G	A	421	0.01781473	15	8.08463611	1.73306162	3.09E-06	5.88E-01	1.00E+00	1.00E+00	CCSER1	CCSER1
rs180828621	10	124533409	G	A	421	0.00712589	6	15.1380574	2.79957627	6.40E-08	5.90E-01	1.00E+00	1.00E+00	DMBT1L1	CUZD1
rs12315614	12	64920957	C	A	421	0.0760095	64	3.77710662	0.8201959	4.12E-06	6.20E-01	1.00E+00	1.00E+00	TBK1	TBK1
rs4562666	1	229824770	T	C	421	0.04275534	36	5.51922599	1.11457457	7.35E-07	6.58E-01	1.00E+00	1.00E+00	URB2	URB2
rs2876414	20	15813704	G	T	421	0.02256532	19	6.81910907	1.48530907	4.41E-06	6.60E-01	1.00E+00	1.00E+00	MACROD2	MACROD2
rs12036586	1	229826378	G	A	421	0.04750594	40	5.22212176	1.07114093	1.09E-06	6.73E-01	1.00E+00	1.00E+00	URB2	URB2
rs75334617	10	102956152	G	A	421	0.03800475	32	5.84603405	1.18027345	7.30E-07	7.59E-01	1.00E+00	1.00E+00	LINC01514	KAZALD1
rs78547898	22	32824278	G	A	421	0.00356295	3	20.9098395	4.15538274	4.85E-07	7.68E-01	1.00E+00	1.00E+00	BPIFC	FOX07
rs796777817	22	32824278	G	A	421	0.00356295	3	20.9098395	4.15538274	4.85E-07	7.68E-01	1.00E+00	1.00E+00	BPIFC	FOX07
rs2274996	1	229804538	C	T	421	0.0415677	35	5.58527433	1.12406363	6.74E-07	7.87E-01	1.00E+00	1.00E+00	URB2	URB2
rs143048774	14	46294660	A	C	421	0.31710214	265	2.28482299	0.49630858	4.15E-06	8.12E-01	1.00E+00	1.00E+00	LINC02303	MIS18BP1
rs428110	14	46294660	A	C	421	0.31710214	265	2.28482299	0.49630858	4.15E-06	8.12E-01	1.00E+00	1.00E+00	LINC02303	MIS18BP1
rs76526501	7	18431110	G	A	421	0.00712589	6	14.8397312	2.5778114	8.58E-09	8.23E-01	1.00E+00	1.00E+00	HDAC9	HDAC9
rs146526206	4	90993018	T	C	421	0.01781473	15	8.09176656	1.69535286	1.82E-06	8.30E-01	1.00E+00	1.00E+00	CCSER1	CCSER1
rs17017794	4	91825885	T	C	421	0.0391924	33	5.83240545	1.14450838	3.47E-07	9.15E-01	1.00E+00	1.00E+00	CCSER1	CCSER1
rs147559909	2	237051523	T	C	421	0.00593824	5	17.0840975	3.03209195	1.76E-08	9.53E-01	1.00E+00	1.00E+00	AGAP1	AGAP1
rs74521112	11	99089147	G	T	421	0.03206651	27	6.25632387	1.22787586	3.48E-07	9.53E-01	1.00E+00	1.00E+00	CNTN5	CNTN5
rs115348382	1	9655903	G	A	421	0.00356295	3	18.2107495	3.84721698	2.21E-06	NA				
rs186532456	1	18948328	C	T	421	0.00356295	3	20.1730343	4.11737745	9.61E-07	NA				
rs562032622	1	18959333	A	C	421	0.00356295	3	21.0267115	4.3197503	1.13E-06	NA				
rs149493615	1	79880089	G	A	421	0.00831354	8	11.750632	2.43451244	1.39E-06	NA				
rs143811231	1	79968131	T	C	421	0.00831354	8	11.5747409	2.42066027	1.74E-06	NA				
rs34270375	1	89370702	G	A	421	0.02612827	21	7.71147017	1.57216792	9.34E-07	NA				
rs187518659	1	99455745	T	G	421	0.00950119	8	11.6819623	2.46564537	2.16E-06	NA				
rs140420703	1	102803484	T	G	421	0.00475059	5	16.487654	3.46213891	1.91E-06	NA				
rs563167766	1	102866365	G	A	421	0.0023753	3	19.415739	4.24240395	4.73E-06	NA				
rs77180278	1	102961882	T	C	421	0.02850356	24	7.33619996	1.37404112	9.34E-08	NA				
rs112351653	1	103220360	T	C	421	0.02850356	24	7.42386947	1.33286772	2.55E-08	NA				
rs180926150	1	103226326	C	T	421	0.0023753	3	19.641918	4.24793649	3.77E-06	NA				
rs114413507	1	103419168	T	C	421	0.02850356	24	7.42216029	1.33294954	2.57E-08	NA				
rs116672066	1	103472916	G	A	421	0.02731591	23	8.04769476	1.37187844	4.46E-09	NA				
rs111928960	1	103633635	G	A	421	0.02850356	22	8.60257208	1.45827516	3.65E-09	NA				
rs113221952	1	103753974	A	G	421	0.02137767	19	7.92350704	1.61421682	9.17E-07	NA				
rs1856085	1	104114545	G	A	421	0.0023753	3	21.51144	4.26747898	4.64E-07	NA				
rs143597860	1	104157143	A	G	421	0.0023753	3	21.5748154	4.2760379	4.52E-07	NA				
rs144541665	1	104310729	G	A	421	0.0023753	3	21.8734611	4.30339839	3.72E-07	NA				
rs76098744	1	112349372	C	T	421	0.01662708	14	8.38612452	1.72704116	1.20E-06	NA				
rs76617932	1	180930424	T	C	421	0.01187648	9	10.5449608	2.25040726	2.79E-06	NA				
rs183180157	1	181247121	A	C	421	0.00950119	7	12.61386	2.68921121	2.72E-06	NA				
rs375790303	1	184530482	G	A	421	0.00831354	7	11.4240393	2.49719192	4.77E-06	NA				
rs138480898	1	184955657	C	T	421	0.00356295	3	16.9873974	3.66972373	3.67E-06	NA				
rs145766563	1	185129502	G	A	421	0.00356295	4	16.8682102	3.54127008	1.90E-06	NA				

rs147032554	1	186148864	T	G	421	0.00356295	3	18.162609	3.65402821	6.68E-07	NA
rs180989936	1	193044178	A	G	421	0.00475059	4	17.1793579	3.70215494	3.48E-06	NA
rs559559983	1	246977132	C	A	421	0.00712589	5	13.811132	2.89725588	1.87E-06	NA
rs550763536	2	7452347	T	G	421	0.00593824	5	14.4871647	3.10349768	3.04E-06	NA
rs558553658	2	15682724	C	T	421	0.00475059	3	17.1099107	3.29108061	2.01E-07	NA
rs536781978	2	29733681	A	G	421	0.00356295	3	17.5870824	3.70259451	2.03E-06	NA
rs568321148	2	29870348	T	G	421	0.00356295	3	17.7125046	3.7201251	1.92E-06	NA
rs76777840	2	48312950	G	A	421	0.00593824	5	14.3500784	2.8927894	7.03E-07	NA
rs145080832	2	48473143	G	A	421	0.00475059	4	17.1618477	3.25709537	1.37E-07	NA
rs184220112	2	48631743	C	A	421	0.00475059	4	16.4879043	3.22267606	3.12E-07	NA
rs189890455	2	48655397	C	T	421	0.00593824	5	15.1467391	2.99355083	4.20E-07	NA
rs181193202	2	52527354	T	C	421	0.00356295	3	19.6312088	3.74776015	1.62E-07	NA
rs183378658	2	52563371	C	T	421	0.00356295	3	19.7446169	3.72634921	1.17E-07	NA
rs190193113	2	53085778	G	A	421	0.00356295	3	19.9533602	3.7619559	1.13E-07	NA
rs187520610	2	53360041	G	A	421	0.00475059	4	18.8010396	3.19364595	3.93E-09	NA
rs149421869	2	53483429	G	T	421	0.00831354	8	11.7382186	2.47204376	2.05E-06	NA
rs146479102	2	65825759	G	A	421	0.00593824	5	15.4846768	3.26980199	2.18E-06	NA
rs528288879	2	65875930	C	T	421	0.00593824	6	14.7878085	2.95121929	5.42E-07	NA
rs11690187	2	67565909	A	C	421	0.00593824	5	13.6310532	2.9751322	4.61E-06	NA
rs151272830	2	67682724	G	T	421	0.00712589	6	13.3323479	2.84806076	2.85E-06	NA
rs186142189	2	67702707	G	A	421	0.00712589	6	12.7361927	2.71405368	2.70E-06	NA
rs184200893	2	69260913	C	T	421	0.00356295	3	19.9580356	4.21905487	2.24E-06	NA
rs111927235	2	74483954	A	G	421	0.00475059	4	17.0468955	3.46111889	8.43E-07	NA
rs111838310	2	74673491	C	A	421	0.00475059	5	17.7963938	3.48429066	3.26E-07	NA
rs112983626	2	74697150	G	A	421	0.00475059	4	17.7848079	3.48532494	3.35E-07	NA
rs113006316	2	74802360	A	G	421	0.0023753	3	22.8886792	4.92411335	3.35E-06	NA
rs76554191	2	95967628	G	A	421	0.04038005	34	5.72148039	1.2389174	3.87E-06	NA
rs140352232	2	108038112	G	A	421	0.0023753	3	21.0576511	4.56013129	3.88E-06	NA
rs116189766	2	126393864	T	C	421	0.0023753	3	21.190764	4.36037289	1.17E-06	NA
rs139877408	2	129606273	A	G	421	0.0023753	3	20.3067129	4.43052497	4.58E-06	NA
rs541508507	2	170023265	G	A	421	0.00475059	4	15.4630033	3.27389618	2.32E-06	NA
rs142549310	2	170030506	C	T	421	0.00475059	4	15.2501713	3.24983634	2.70E-06	NA
rs556293455	2	176950420	G	A	421	0.00356295	3	14.3860463	2.96998402	1.27E-06	NA
rs184098071	2	177116420	G	A	421	0.00356295	3	13.947724	2.86782452	1.15E-06	NA
rs532416695	2	177486790	G	A	421	0.00356295	3	19.8726656	3.76737683	1.33E-07	NA
rs112557251	2	188375400	T	C	421	0.0023753	3	20.6510064	4.44850106	3.45E-06	NA
rs185158855	2	223650026	C	A	421	0.00475059	4	16.6678864	3.60266524	3.72E-06	NA
rs185510569	2	223814861	G	A	421	0.00356295	3	19.5886906	3.60490977	5.51E-08	NA
rs181217257	2	239989119	C	T	421	0.00475059	4	21.5677368	3.47296989	5.29E-10	NA
rs188076929	2	239993719	T	C	421	0.00475059	4	19.9775614	3.27246746	1.03E-09	NA
rs112475378	3	1626661	T	C	421	0.01662708	14	9.55085929	1.97628138	1.35E-06	NA
rs145676540	3	2044635	C	T	421	0.00593824	5	13.980658	2.88447377	1.25E-06	NA
rs146007933	3	28227423	T	C	421	0.02137767	18	7.51149453	1.55442805	1.35E-06	NA
rs73057656	3	33940571	A	G	421	0.0368171	33	5.9238329	1.27589711	3.44E-06	NA
rs142684595	3	55319301	T	C	421	0.00593824	5	16.7729431	3.11364755	7.17E-08	NA
rs80203220	3	122722331	C	T	421	0.00593824	6	13.426042	2.84218656	2.31E-06	NA
rs192443987	3	135532345	G	A	421	0.00475059	4	17.7540285	3.67564302	1.36E-06	NA
rs148248743	3	136134595	C	T	421	0.0023753	3	22.839379	4.62044689	7.69E-07	NA
rs576124203	3	141841196	T	G	421	0.00356295	3	19.4039925	3.84392582	4.47E-07	NA
rs545552231	3	141864350	C	T	421	0.00356295	3	19.8641332	3.94582563	4.80E-07	NA
rs188720948	3	150069880	T	C	421	0.00356295	3	17.7032653	3.59722874	8.59E-07	NA
rs139943877	3	155451289	G	A	421	0.00712589	7	13.0535902	2.76041594	2.26E-06	NA

rs187047882	3	164285621	G	A	421	0.00356295	3	18.5641361	3.99725417	3.41E-06	NA
rs141169929	3	164808462	A	G	421	0.00475059	5	14.8454178	3.1738839	2.91E-06	NA
rs186649043	3	174932293	C	T	421	0.00356295	3	19.2134209	4.06588786	2.30E-06	NA
rs189709453	3	177574308	G	A	421	0.00356295	3	21.6581488	4.05916142	9.52E-08	NA
rs186767531	3	177639777	T	C	421	0.00356295	3	21.2013243	4.04780758	1.63E-07	NA
rs182868205	3	177712448	C	T	421	0.00356295	3	20.9548768	3.97841839	1.39E-07	NA
rs191792521	3	195646605	G	A	421	0.00831354	6	13.9515484	2.96443599	2.52E-06	NA
rs189765693	4	4324793	T	C	421	0.00475059	4	15.5191341	3.29924607	2.55E-06	NA
rs183962155	4	21259643	A	C	421	0.00593824	6	13.7646008	2.97247759	3.64E-06	NA
rs113751774	4	23428854	C	T	421	0.00712589	7	14.9103274	2.96657459	5.01E-07	NA
rs113063005	4	23509067	T	C	421	0.00593824	6	21.2073894	3.15520096	1.80E-11	NA
rs145875128	4	32073136	G	A	421	0.00356295	3	24.479986	4.60639213	1.07E-07	NA
rs143287889	4	35572280	C	T	421	0.0023753	3	19.96741	4.34406	4.30E-06	NA
rs77141817	4	37053759	T	C	421	0.00356295	3	20.6110292	3.64214866	1.52E-08	NA
rs190822761	4	37099356	G	T	421	0.00356295	3	20.5846354	3.64346559	1.61E-08	NA
rs999769259	4	62511965	G	A	421	0.00356295	3	22.1246725	4.77597881	3.61E-06	NA
rs145116559	4	112677596	T	C	421	0.00356295	4	18.2441224	3.9932536	4.91E-06	NA
rs181415102	4	112689266	T	C	421	0.00356295	4	18.2631019	3.99387944	4.81E-06	NA
rs191423619	4	126516627	G	T	421	0.0023753	3	21.694611	4.20667337	2.51E-07	NA
rs149298750	4	127158701	A	C	421	0.00356295	3	22.184052	4.26573246	1.99E-07	NA
rs112679237	4	139140860	T	C	421	0.01781473	18	8.71202752	1.67680671	2.04E-07	NA
rs531769270	4	154411043	T	C	421	0.00356295	3	16.5945338	3.51936092	2.41E-06	NA
rs116651654	4	163238743	C	T	421	0.00712589	6	15.6223694	3.06624451	3.49E-07	NA
rs567982164	5	25631297	G	A	421	0.0023753	3	21.8356286	4.36706703	5.73E-07	NA
rs191986449	5	25745687	C	T	421	0.00475059	3	18.4978793	4.03679831	4.60E-06	NA
rs185771987	5	73285489	T	C	421	0.00356295	3	18.2344824	3.97675014	4.53E-06	NA
rs139360368	5	73372109	A	C	421	0.00356295	4	18.3590487	3.78701272	1.25E-06	NA
rs181933850	5	91465647	A	G	421	0.00831354	7	11.0436586	2.37544216	3.33E-06	NA
rs190190051	5	91475485	G	A	421	0.00831354	7	11.0589887	2.38317975	3.48E-06	NA
rs182531466	5	91530073	C	A	421	0.00712589	6	12.6804978	2.75685261	4.23E-06	NA
rs187236873	5	91530447	G	A	421	0.00712589	6	12.44907	2.61780937	1.98E-06	NA
rs183816745	5	91631888	A	G	421	0.00593824	5	17.3487909	3.20195556	6.02E-08	NA
rs111407636	5	95080029	C	T	421	0.00356295	3	18.642774	3.74168052	6.28E-07	NA
rs111676272	5	95094298	C	A	421	0.00356295	3	18.6068625	3.74429748	6.72E-07	NA
rs75848314	5	95098340	T	C	421	0.00356295	3	20.0998481	3.78700451	1.11E-07	NA
rs111846247	5	95111643	T	C	421	0.00356295	3	20.4865772	3.85467205	1.07E-07	NA
rs137873790	5	97087041	A	G	421	0.01306413	11	9.22240881	2.01095379	4.52E-06	NA
rs189912648	5	134765439	C	T	421	0.00356295	4	16.9732599	3.60846227	2.55E-06	NA
rs191006910	5	154216009	G	A	421	0.00356295	3	17.6173803	3.79049689	3.36E-06	NA
rs74343174	5	161493182	C	A	421	0.00593824	5	14.7671824	3.13913408	2.55E-06	NA
rs775626702	5	162630067	A	C	421	0.00356295	3	18.9484137	4.07023372	3.23E-06	NA
rs371245624	5	162880956	T	C	421	0.00356295	3	19.4795931	4.06572011	1.66E-06	NA
rs545428520	5	167821266	T	C	421	0.00356295	4	21.566035	3.66407988	3.96E-09	NA
rs528404963	5	167852025	T	C	421	0.00356295	4	21.1393977	3.65303517	7.17E-09	NA
rs72832764	5	170004673	G	A	421	0.00356295	3	18.2159757	3.88196743	2.70E-06	NA
rs72837643	5	170188955	T	C	421	0.00356295	4	16.9302874	3.67743479	4.15E-06	NA
rs142311947	5	177384469	G	A	421	0.00712589	8	12.0339013	2.57618116	2.99E-06	NA
rs151015676	5	177390937	T	G	421	0.0023753	3	20.1421367	4.37074482	4.06E-06	NA
rs571986619	6	85678832	A	G	421	0.0023753	3	20.7858067	4.34301983	1.70E-06	NA
rs56224400	6	98092675	T	C	421	0.01662708	14	8.4433064	1.72251471	9.50E-07	NA
rs147627638	6	99173116	A	G	421	0.00712589	6	13.0785306	2.79364254	2.85E-06	NA
rs141326851	6	134833127	A	C	421	0.01662708	14	8.57849688	1.81053268	2.16E-06	NA

rs146048121	6	142198955	G	A	421	0.0023753	3	20.0214379	4.30086025	3.24E-06	NA
rs142106992	6	142269885	C	A	421	0.00356295	4	22.8345886	3.6758699	5.23E-10	NA
rs72983831	6	142307100	T	G	421	0.00593824	6	15.6733239	3.17294911	7.83E-07	NA
rs72986533	6	142611258	T	C	421	0.00831354	8	13.2812726	2.53063629	1.54E-07	NA
rs73586304	6	142839425	C	T	421	0.00356295	3	18.2405116	3.99428202	4.96E-06	NA
rs148532212	6	165499857	T	C	421	0.00356295	3	19.3535457	3.70945693	1.81E-07	NA
rs117498042	6	165514281	C	T	421	0.00356295	3	19.335471	3.70913521	1.86E-07	NA
rs148153037	6	167501386	G	A	421	0.00831354	8	14.3359246	2.42128102	3.20E-09	NA
rs184487573	6	167513471	A	G	421	0.00712589	7	13.5009635	2.59268014	1.92E-07	NA
rs17776100	7	6426479	G	A	421	0.02969121	25	6.63697397	1.32177185	5.13E-07	NA
rs187978759	7	11711845	G	A	421	0.00356295	3	20.4555522	4.3637719	2.76E-06	NA
rs117166500	7	17052778	G	T	421	0.00712589	7	12.5146865	2.62694198	1.90E-06	NA
rs55844051	7	23360363	T	C	421	0.00356295	3	18.3875896	3.86268704	1.93E-06	NA
rs574076561	7	49544747	A	G	421	0.00356295	3	19.6434766	4.25225723	3.85E-06	NA
rs181259864	7	97488823	C	A	421	0.00356295	3	17.1360821	3.68242789	3.26E-06	NA
rs192750513	7	97577830	A	G	421	0.00356295	3	17.1892469	3.72514218	3.94E-06	NA
rs539713344	7	100474786	G	A	421	0.0023753	3	20.044756	4.21957545	2.03E-06	NA
rs188028357	7	100668425	C	T	421	0.0023753	3	21.8425578	4.35791809	5.38E-07	NA
rs536023430	7	146865072	T	C	421	0.00356295	3	19.4038683	4.13084856	2.64E-06	NA
rs187384541	8	1632241	A	G	421	0.03562945	26	6.88745117	1.47776668	3.15E-06	NA
rs575473987	8	5577494	C	T	421	0.00356295	3	22.2248653	3.88625754	1.07E-08	NA
rs139062456	8	13251991	C	T	421	0.01781473	15	7.83515809	1.69687423	3.89E-06	NA
rs185874707	8	18030674	C	T	421	0.01187648	10	9.13934765	1.94918644	2.75E-06	NA
rs140797780	8	22087792	C	T	421	0.00356295	3	19.2908396	3.69147785	1.73E-07	NA
rs188415494	8	25613298	C	T	421	0.00356295	3	18.5169478	3.9914865	3.50E-06	NA
rs117816016	8	103751262	C	T	421	0.00356295	3	19.8807023	4.26960212	3.22E-06	NA
rs567383525	8	115029494	C	T	421	0.00356295	3	17.7455227	3.71970627	1.84E-06	NA
rs545550279	8	115552429	G	T	421	0.00356295	3	19.1812421	4.19212988	4.75E-06	NA
rs536803366	8	123001411	T	C	421	0.0023753	3	19.2098639	4.00952057	1.66E-06	NA
rs555249476	8	123001411	T	C	421	0.0023753	3	19.2098639	4.00952057	1.66E-06	NA
rs532513136	8	135813748	C	A	421	0.00356295	3	19.9490594	4.03118599	7.47E-07	NA
rs532730683	9	1784492	G	T	421	0.00356295	3	18.726838	3.8314675	1.02E-06	NA
rs540065886	9	2770228	T	C	421	0.00356295	3	19.9489151	4.18802805	1.90E-06	NA
rs543844012	9	30107023	C	T	421	0.00356295	3	18.1160485	3.9446103	4.38E-06	NA
rs188034471	9	85937714	G	A	421	0.00475059	4	14.7559999	3.14925113	2.79E-06	NA
rs190294315	9	85945465	C	T	421	0.00593824	5	14.5664276	2.88126649	4.29E-07	NA
rs545690161	9	93030699	G	A	421	0.00593824	6	17.1436874	3.00318779	1.14E-08	NA
rs565682685	9	93222328	T	C	421	0.00475059	4	18.938397	3.7206174	3.58E-07	NA
rs183737367	9	93330047	T	C	421	0.00356295	3	23.3452306	4.24471431	3.80E-08	NA
rs187213609	9	93415465	C	T	421	0.00356295	3	23.1975304	4.27412656	5.72E-08	NA
rs150027952	9	103385416	A	G	421	0.0023753	3	21.8616648	4.51464643	1.28E-06	NA
rs146207930	9	129042336	A	G	421	0.00712589	6	11.8624321	2.51502465	2.40E-06	NA
rs78296164	9	135266715	C	T	421	0.00831354	7	11.6095338	2.33917557	6.94E-07	NA
rs77871739	9	138552309	G	A	421	0.00475059	4	15.0724603	3.23606181	3.20E-06	NA
rs184425183	10	13457520	A	G	421	0.00356295	3	22.6683174	3.77549648	1.92E-09	NA
rs184458518	10	13471195	T	G	421	0.00356295	3	22.8015789	3.77123942	1.48E-09	NA
rs117998251	10	13497976	C	T	421	0.00356295	3	22.9543311	3.77480488	1.19E-09	NA
rs117025967	10	20207052	C	A	421	0.01306413	11	9.66973563	1.927439	5.25E-07	NA
rs529011661	10	20372316	G	A	421	0.00475059	4	15.6393469	3.3125339	2.34E-06	NA
rs138249376	10	63515829	T	G	421	0.00475059	4	16.4300711	3.59466791	4.86E-06	NA
rs140277951	10	82359100	G	A	421	0.00831354	6	13.7463158	2.64333932	1.99E-07	NA
rs566018180	10	86755052	C	T	421	0.00356295	3	18.563552	4.04215862	4.38E-06	NA

rs140706881	10	96217535	G	A	421	0.00475059	4	18.974805	3.93288931	1.40E-06	NA
rs752259256	10	104925319	T	C	421	0.00356295	3	20.4258639	4.45411591	4.52E-06	NA
rs147393020	10	124779274	A	G	421	0.00593824	5	14.1853505	2.95980896	1.65E-06	NA
rs193093906	10	126705489	G	A	421	0.00950119	9	10.5219537	2.25840517	3.18E-06	NA
rs151115079	11	18655741	T	C	421	0.00475059	5	17.8273587	3.2680205	4.89E-08	NA
rs138414342	11	18679398	G	A	421	0.00475059	5	18.1582505	3.28214943	3.16E-08	NA
rs541653703	11	18701786	G	A	421	0.00475059	4	18.3169499	3.27243418	2.18E-08	NA
rs118093638	11	18718324	C	T	421	0.00593824	5	14.2657259	2.91296506	9.72E-07	NA
rs181812512	11	66665729	C	T	421	0.00356295	3	19.5093168	4.24219816	4.25E-06	NA
rs529345909	11	67110852	A	G	421	0.00356295	3	18.70153	4.09206035	4.87E-06	NA
rs544042801	11	68460243	G	A	421	0.00475059	4	15.8869866	3.40519019	3.08E-06	NA
rs149949098	11	95099866	G	A	421	0.01662708	13	8.55927625	1.81883308	2.53E-06	NA
rs148781275	11	103640603	A	G	421	0.00475059	5	15.7111853	3.32183832	2.25E-06	NA
rs141281289	11	123693024	A	G	421	0.00593824	6	15.811518	3.0889522	3.08E-07	NA
rs528140343	11	125719227	A	C	421	0.00356295	3	18.2835521	3.72738029	9.33E-07	NA
rs546409459	11	125754989	A	G	421	0.00356295	3	18.9195474	4.00286836	2.28E-06	NA
rs528609331	11	125842195	C	T	421	0.00356295	3	19.5812418	3.94832705	7.07E-07	NA
rs7104959	11	129846126	C	T	421	0.00356295	3	17.9204611	3.84366851	3.13E-06	NA
rs189360484	12	1870510	A	G	421	0.00475059	4	16.8078794	3.31424855	3.95E-07	NA
rs141754456	12	20151132	T	C	421	0.00712589	7	12.1460333	2.32842921	1.82E-07	NA
rs118184666	12	20424749	G	A	421	0.00712589	6	10.9509803	2.32477242	2.47E-06	NA
rs549931083	12	20516286	A	C	421	0.00356295	3	14.3282694	2.79891395	3.07E-07	NA
rs151323346	12	21012024	T	C	421	0.00593824	4	15.8903888	3.38034868	2.59E-06	NA
rs371879555	12	23015962	T	C	421	0.00356295	3	20.1033765	4.20002323	1.70E-06	NA
rs183466664	12	26821687	A	G	421	0.00475059	4	16.0181882	3.45609016	3.57E-06	NA
rs77353774	12	28248852	G	A	421	0.00712589	6	13.1054362	2.67954103	1.00E-06	NA
rs113167689	12	28435962	C	T	421	0.00712589	6	14.1364135	2.73335629	2.32E-07	NA
rs17510814	12	28468969	A	C	421	0.00712589	6	14.0387924	2.71409689	2.31E-07	NA
rs141756120	12	28511096	A	C	421	0.00831354	6	14.278403	2.76458538	2.41E-07	NA
rs117991215	12	28511473	T	C	421	0.00831354	6	14.333666	2.77918736	2.50E-07	NA
rs191930622	12	48284655	G	A	421	0.0023753	3	19.6783579	4.30610368	4.88E-06	NA
rs56302696	12	48292830	G	A	421	0.0023753	3	19.8959041	4.31584707	4.03E-06	NA
rs185620578	12	48569399	C	T	421	0.0023753	3	20.7232267	4.39505442	2.42E-06	NA
rs190806532	12	48862147	G	T	421	0.0023753	3	21.326955	4.43856043	1.55E-06	NA
rs568658857	12	49853998	G	A	421	0.00593824	5	14.2361193	3.09423422	4.21E-06	NA
rs137880949	12	63306297	T	C	421	0.00475059	3	21.6084034	4.13589168	1.75E-07	NA
rs191053292	12	63445280	T	C	421	0.00356295	3	22.9266488	4.11439732	2.51E-08	NA
rs182437250	12	63608466	T	C	421	0.00475059	4	18.8083451	3.79343554	7.12E-07	NA
rs76904423	12	101188744	G	A	421	0.01068884	10	9.80576231	2.11568908	3.57E-06	NA
rs180764936	12	101498621	T	C	421	0.00356295	3	19.3473053	4.0200238	1.49E-06	NA
rs185855183	12	101505126	C	T	421	0.00356295	3	18.7397727	3.86137343	1.22E-06	NA
rs139598422	13	23887014	A	G	421	0.00356295	4	20.4886522	3.64167194	1.84E-08	NA
rs143371352	13	47383834	C	T	421	0.00356295	3	20.5341625	3.87928964	1.20E-07	NA
rs75186966	13	47395758	A	C	421	0.00356295	3	20.2281345	3.83225523	1.30E-07	NA
rs150077525	13	57979549	A	G	421	0.00712589	7	12.9984875	2.71312414	1.66E-06	NA
rs534845494	13	58213864	A	G	421	0.00475059	5	15.9431765	3.18484007	5.56E-07	NA
rs140062526	13	59286033	G	A	421	0.00593824	5	14.0926088	3.02052903	3.08E-06	NA
rs546286713	13	91411079	G	A	421	0.00475059	4	15.7027841	3.3611216	2.98E-06	NA
rs567080482	13	95075405	T	C	421	0.00475059	4	15.4999561	3.31620837	2.95E-06	NA
rs142928734	13	101601082	G	A	421	0.00356295	3	17.009593	3.70044989	4.29E-06	NA
rs556680896	13	101602415	C	T	421	0.00356295	3	17.0254659	3.70286799	4.27E-06	NA
rs184265355	13	108011352	A	C	421	0.00593824	6	14.4271719	2.91829792	7.67E-07	NA

rs572961122	13	108012985	C	T	421	0.00593824	6	13.3354896	2.85827607	3.08E-06	NA
rs528809914	13	113041256	G	A	421	0.00356295	3	19.5048552	3.76867111	2.27E-07	NA
rs138215817	14	22641516	A	G	421	0.00475059	4	16.311019	3.27367022	6.28E-07	NA
rs74704551	14	30161887	C	T	421	0.00356295	3	24.1990705	4.31064693	1.98E-08	NA
rs116862847	14	64141677	C	T	421	0.00712589	7	15.3160369	2.95194262	2.12E-07	NA
rs569916471	14	75891342	G	A	421	0.00475059	5	15.6404088	3.3537133	3.11E-06	NA
rs113767990	14	81717563	G	A	421	0.00475059	4	14.5811155	3.19363095	4.98E-06	NA
rs190251199	14	105590577	T	C	421	0.00356295	3	20.0125292	4.01627173	6.27E-07	NA
rs185155853	15	41244100	C	T	421	0.00475059	5	15.9164657	3.40838631	3.02E-06	NA
rs144026361	15	41248669	C	T	421	0.00475059	5	16.1372994	3.43734659	2.67E-06	NA
rs558614420	15	41810870	C	T	421	0.00593824	6	13.9750831	3.02772732	3.92E-06	NA
rs138109686	15	42051442	A	G	421	0.00593824	6	13.5956705	2.96761718	4.62E-06	NA
rs145896760	15	42119222	G	A	421	0.00593824	6	13.3688825	2.92346809	4.81E-06	NA
rs140642138	15	42125165	G	A	421	0.00593824	6	13.5832221	2.96274049	4.55E-06	NA
rs6080	15	58837933	C	A	421	0.04394299	36	5.66476745	1.2393253	4.86E-06	NA
rs145439370	15	58879765	T	C	421	0.03087886	26	6.89328891	1.40339117	9.02E-07	NA
rs149425014	15	58951660	T	C	421	0.02612827	22	7.55224792	1.58320604	1.84E-06	NA
rs146442492	15	58982115	C	T	421	0.02731591	24	7.05925858	1.51661316	3.25E-06	NA
rs193253461	15	59229353	A	G	421	0.01306413	12	10.5374468	2.07404763	3.76E-07	NA
rs184117160	15	59404306	C	T	421	0.01425178	11	10.3417236	2.13366338	1.25E-06	NA
rs80292573	15	59435086	T	G	421	0.03444181	30	6.55323491	1.32696583	7.87E-07	NA
rs182303755	15	59634792	A	C	421	0.01306413	12	10.5479917	2.09359291	4.70E-07	NA
rs138217865	15	94392882	C	T	421	0.00475059	4	16.709641	3.45193896	1.29E-06	NA
rs553840536	16	25697895	A	G	421	0.00356295	3	19.6203442	4.23547954	3.61E-06	NA
rs183817723	16	59302775	C	T	421	0.00356295	4	17.5860507	3.7341919	2.48E-06	NA
rs144954214	16	76179362	A	G	421	0.0023753	3	21.8113263	4.43942529	8.96E-07	NA
rs529523094	16	77715551	A	G	421	0.00356295	3	18.1584346	3.72617419	1.10E-06	NA
rs146728064	17	19265440	G	A	421	0.00712589	6	12.6400411	2.73467115	3.80E-06	NA
rs191271637	17	52123260	A	G	421	0.00356295	3	18.379836	3.92083355	2.76E-06	NA
rs185819304	18	27001580	G	A	421	0.00356295	3	19.9401113	3.94501953	4.32E-07	NA
rs187942235	18	27030430	C	T	421	0.00356295	3	20.3968658	3.96963025	2.77E-07	NA
rs143538552	18	29050262	A	G	421	0.00356295	3	19.1457218	3.63679703	1.41E-07	NA
rs373746073	18	29058384	C	A	421	0.00356295	3	19.0847197	3.63798064	1.55E-07	NA
rs146333745	18	55497457	C	T	421	0.00356295	4	17.4215784	3.56782335	1.04E-06	NA
rs185464792	19	18797371	C	T	421	0.0023753	3	22.5896015	4.50191473	5.23E-07	NA
rs186768950	19	18806124	C	A	421	0.0023753	3	22.7152061	4.50776479	4.68E-07	NA
rs559008174	19	18876059	C	T	421	0.00475059	5	15.6025661	3.12812006	6.11E-07	NA
rs546144116	19	19563339	C	T	421	0.0023753	3	23.1354815	4.47162758	2.29E-07	NA
rs560206697	19	20729098	C	T	421	0.0023753	3	24.9989943	4.55350673	4.02E-08	NA
rs111285015	19	23123198	G	A	421	0.00356295	3	27.3347642	4.62083983	3.31E-09	NA
rs1008091735	19	31090099	T	C	421	0.00356295	3	18.4689243	3.69079909	5.61E-07	NA
rs148433854	19	31096478	G	A	421	0.00356295	3	18.6267585	3.68806754	4.41E-07	NA
rs140788628	20	15858501	C	A	421	0.01068884	8	11.8039284	2.2826396	2.33E-07	NA
rs559228693	20	15963329	G	A	421	0.00593824	5	14.3643551	3.08196497	3.15E-06	NA
rs184785969	21	17171431	C	A	421	0.00356295	3	19.8736486	3.70683937	8.26E-08	NA
rs117280553	21	17207163	T	C	421	0.00356295	3	20.087417	3.70274826	5.80E-08	NA
rs79486609	21	17245006	G	A	421	0.00356295	3	20.6624886	3.77824957	4.53E-08	NA
rs73227413	21	23136973	G	A	421	0.03444181	28	5.96205514	1.27150176	2.75E-06	NA
rs75024143	21	23156546	G	T	421	0.01425178	12	9.81254241	2.0684542	2.10E-06	NA
rs192134381	21	23450714	T	C	421	0.00356295	3	21.788812	3.73125357	5.23E-09	NA
rs397836601	21	23450714	T	C	421	0.00356295	3	21.788812	3.73125357	5.23E-09	NA
rs118183140	21	35477486	C	T	421	0.02019002	17	7.5893248	1.56122232	1.17E-06	NA

rs183586634	21	38763032	G	A	421	0.00593824	5	14.3132202	2.99210048	1.72E-06	NA
rs117185941	21	38766484	G	A	421	0.00593824	5	15.3808786	3.17143013	1.24E-06	NA
rs1329159859	21	38766484	G	A	421	0.00593824	5	15.3808786	3.17143013	1.24E-06	NA
rs118084887	21	38863820	T	C	421	0.00593824	5	14.9382616	3.13674741	1.91E-06	NA
rs150539922	21	43276916	T	C	421	0.00356295	3	16.6696937	3.64477381	4.79E-06	NA
rs113625788	22	19969182	C	T	421	0.00831354	7	11.8084829	2.42941561	1.17E-06	NA
rs541680196	22	40528090	G	A	421	0.00593824	5	14.3867808	2.94903659	1.07E-06	NA
rs185139807	22	40594781	G	A	421	0.00593824	5	14.4330526	2.95312686	1.02E-06	NA
rs141127122	22	40604439	G	A	421	0.00475059	4	15.3824875	3.30083549	3.16E-06	NA
rs148998974	22	40620530	A	G	421	0.00593824	5	14.5129532	2.94976024	8.65E-07	NA
rs555040883	22	40631476	G	A	421	0.00475059	4	15.4273069	3.30542717	3.05E-06	NA
rs150946694	22	46853180	T	C	421	0.00475059	4	15.3082479	3.29125059	3.30E-06	NA
