# Supplementary material for: Pharmacogenomics of steroid-induced ocular hypertension: relationship to high-tension glaucomas and new pathophysiologic insight
Source: medRxiv. 2025 Aug 13:2025.08.11.25333245. Preprint. [Version 1] doi: 10.1101/2025.08.11.25333245 (PMC12363710; doi:10.1101/2025.08.11.25333245)
Supplement: Supplement 9 — Table S8. Independent Replication [file media-9.pdf]

Supplementary Table S8. GWAS Results Indianapolis-2 Replication Cohort
12 month quantitative trait (QT), P-value ordered

ID	chr	POS_37	freq	MAC	Score	Score.SE	Score.Stat	Score.pval	Func.refGene	Gene.refGene	GeneDetail.refGene	rsID	gnomAD_ genome_ALL	gnomAD_ genome_ NFE	Rsq	hwe	chr_38	POS_38
18:6045905:T:A	18	6045905	0.049893204	10	2.439599988	0.449001371	5.43339095	5.52931E-08	intronic	L3MBTL4	.	rs74649788	0.0811	0.0475	0.923243	1	chr18	6045906
13:30768424:G:A	13	30768424	0.029165049	6	1.909228275	0.360021719	5.303091934	1.13858E-07	intergenic	LINC00365;KATNAL1	dist=85412;dist=8343	rs74833295	0.0153	0.0232	0.9983	1	chr13	30194287
3:146048618:G:A	3	146048618	0.023815534	5	1.481987222	0.287749854	5.150262284	2.60122E-07	intergenic	PLSCR4;PLSCR2	dist=79652;dist=102457	rs73150883	0.0163	0.0252	0.848249	1	chr3	146330831
8:23542722:T:A	8	23542722	0.016383495	3	1.2982161	0.255687499	5.077354608	3.82726E-07	intergenic	NKX3-1;NKX2-6	dist=2272;dist=17242	rs189490695	0.0070	0.0092	0.874937	1	chr8	23685209
8:23462708:A:G	8	23462708	0.016941748	3	1.341800652	0.264601135	5.071031363	3.95666E-07	intergenic	SLC25A37;NKX3-1	dist=32645;dist=73498	rs539324287	0.0033	0.0057	0.907302	1	chr8	23605195
17:75157900:C:T	17	75157900	0.033300971	7	1.815437814	0.358406655	5.065301631	4.07754E-07	intronic	SEC14L1	.	rs113199364	0.0230	0.0364	0.893794	1	chr17	77161818
3:75482532:G:C	3	75482532	0.011626214	2	0.80016771	0.159019081	5.031897471	4.85649E-07	ncRNA_intronic	FAM86DP	.	rs185293133	0.0054	0.0078	0.492536	1	chr3	75433381
18:6049551:C:A	18	6049551	0.044349515	9	2.087223432	0.417798884	4.995761149	5.86042E-07	intronic	L3MBTL4	.	rs58503971	0.0786	0.0441	0.959099	1	chr18	6049552
18:6049026:T:A	18	6049026	0.044359223	9	2.087445553	0.417849208	4.995691057	5.86254E-07	intronic	L3MBTL4	.	rs79381542	0.0807	0.0470	0.959092	1	chr18	6049027
5:115979852:T:C	5	115979852	0.029092233	6	1.782650366	0.361233296	4.934900476	8.01916E-07	intergenic	SEMA6A;LOC102467223	dist=69230;dist=99146	rs114241096	0.0185	0.0229	0.998458	1	chr5	116644156
5:115980038:G:C	5	115980038	0.029092233	6	1.782650366	0.361233296	4.934900476	8.01916E-07	intergenic	SEMA6A;LOC102467223	dist=69416;dist=98960	rs114587121	0.0185	0.0229	0.998458	1	chr5	116644342
5:115980724:A:G	5	115980724	0.029097087	6	1.782403345	0.361231089	4.934246796	8.04606E-07	intergenic	SEMA6A;LOC102467223	dist=70102;dist=98274	rs79516960	0.0171	0.0229	0.998286	1	chr5	116645028
5:115980901:A:C	5	115980901	0.029097087	6	1.782403345	0.361231089	4.934246796	8.04606E-07	intergenic	SEMA6A;LOC102467223	dist=70279;dist=98097	rs78085382	0.0186	0.0228	0.998286	1	chr5	116645205
5:115981236:C:G	5	115981236	0.029097087	6	1.782403345	0.361231089	4.934246796	8.04606E-07	intergenic	SEMA6A;LOC102467223	dist=70614;dist=97762	rs79794218	0.0170	0.0229	0.998286	1	chr5	116645540
5:115981990:C:G	5	115981990	0.029097087	6	1.782403345	0.361231089	4.934246796	8.04606E-07	intergenic	SEMA6A;LOC102467223	dist=71368;dist=97008	rs114108437	0.0170	0.0229	0.998286	1	chr5	116646294
5:115991913:A:T	5	115991913	0.029087379	6	1.782276135	0.361233864	4.933856736	8.06216E-07	intergenic	SEMA6A;LOC102467223	dist=81291;dist=87085	rs76006987	0.0185	0.0225	0.998629	1	chr5	116656217
5:115988180:G:A	5	115988180	0.029126214	6	1.784300998	0.361724673	4.932759998	8.10758E-07	intergenic	SEMA6A;LOC102467223	dist=77558;dist=90818	rs77857559	0.0165	0.0221	1	1	chr5	116652484
5:115988843:C:T	5	115988843	0.029126214	6	1.784300998	0.361724673	4.932759998	8.10758E-07	intergenic	SEMA6A;LOC102467223	dist=78221;dist=90155	rs115917626	0.0167	0.0223	1	1	chr5	116653147
13:52703883:C:G	13	52703883	0.036063107	7	1.896523445	0.386307062	4.90936778	9.13705E-07	ncRNA_exonic	LOC101929657	.	rs148654242	0.0202	0.0302	0.890541	1	chr13	52129747
5:115976909:A:C	5	115976909	0.029834951	6	1.764770451	0.360470372	4.895743415	9.79348E-07	intergenic	SEMA6A;LOC102467223	dist=66287;dist=102089	rs76433992	0.0168	0.0226	0.974211	1	chr5	116641213
2:50062774:T:G	2	50062774	0.030864078	6	1.721021507	0.356977463	4.821092882	1.42774E-06	intergenic	FSHR;NRXN1	dist=681108;dist=82869	rs138665118	0.0114	0.0171	0.892953	1	chr2	49835636
5:162653096:T:C	5	162653096	0.02438835	5	1.593867941	0.333478221	4.779526333	1.75709E-06	intergenic	GABRG2;CCNG1	dist=1070551;dist=211481	rs116626718	0.0249	0.0325	0.995221	1	chr5	163226090
2:126913427:C:G	2	126913427	0.033174757	7	1.808450116	0.378512536	4.777781303	1.7724E-06	intergenic	CNTNAP5;GYPC	dist=1240473;dist=500084	rs28798228	0.0111	0.0144	0.961063	1	chr2	126155850
2:74482548:G:T	2	74482548	0.032208738	7	1.780987998	0.373137772	4.77300378	1.81498E-06	intronic	SLC4A5	.	rs12621729	0.1479	0.0335	0.942493	1	chr2	74255421
5:162646718:G:C	5	162646718	0.024461165	5	1.583713858	0.331858007	4.772263512	1.82167E-06	intergenic	GABRG2;CCNG1	dist=1064173;dist=217859	rs115331238	0.0236	0.0306	0.983743	1	chr5	163219712
15:27636953:T:G	15	27636953	0.067961165	14	2.448622014	0.514411414	4.760046044	1.93549E-06	intronic	GABRG3	.	rs72705714	0.0522	0.0496	1	1	chr15	27391807
15:27636387:G:A	15	27636387	0.067961166	14	2.444243265	0.513892292	4.756333777	1.9714E-06	intronic	GABRG3	.	rs72705712	0.0463	0.0496	0.998164	1	chr15	27391241
15:27637194:T:C	15	27637194	0.067961166	14	2.444243265	0.513892292	4.756333777	1.9714E-06	intronic	GABRG3	.	rs72705715	0.0428	0.0496	0.998164	1	chr15	27392048
15:27635356:C:G	15	27635356	0.067970874	14	2.443670474	0.513826908	4.75582426	1.97638E-06	intronic	GABRG3	.	rs72705711	0.0601	0.0498	0.997858	1	chr15	27390210
2:126955214:G:A	2	126955214	0.032441747	7	1.7618909	0.370731447	4.752472211	2.00944E-06	intergenic	CNTNAP5;GYPC	dist=1282260;dist=458297	rs28796094	0.0111	0.0144	0.939443	1	chr2	126197637
7:9489611:T:G	7	9489611	0.019781553	4	1.317750936	0.277733732	4.744655705	2.08861E-06	intergenic	NXPH1;PER4	dist=697018;dist=184289	rs17206867	0.0067	0.0108	0.886061	1	chr7	9449981
15:31383118:A:C	15	31383118	0.019359223	4	1.431828899	0.302121941	4.739241691	2.1452E-06	intronic	TRPM1	.	rs75968509	0.0124	0.0149	0.979554	1	chr15	31090915
5:162661735:C:T	5	162661735	0.025213592	5	1.5722456	0.331875986	4.73744913	2.16425E-06	intergenic	GABRG2;CCNG1	dist=1079190;dist=202842	rs114008181	0.0256	0.0337	0.958521	1	chr5	163234729
15:27633984:A:C	15	27633984	0.068470875	14	2.432058671	0.513440775	4.736785214	2.17135E-06	intronic	GABRG3	.	rs72705708	0.0450	0.0491	0.990527	1	chr15	27388838
5:162629778:G:A	5	162629778	0.024538835	5	1.5321512	0.32364261	4.734083693	2.20047E-06	intergenic	GABRG2;CCNG1	dist=1047233;dist=234799	rs116189826	0.0218	0.0286	0.937899	1	chr5	163202772
15:27634215:C:T	15	27634215	0.068349515	14	2.429618929	0.513388602	4.732514355	2.21756E-06	intronic	GABRG3	.	rs72705709	0.0448	0.0489	0.992399	1	chr15	27389069
15:27634405:G:C	15	27634405	0.068320389	14	2.428373305	0.513251522	4.731351398	2.2303E-06	intronic	GABRG3	.	rs72705710	0.0448	0.0489	0.992701	1	chr15	27389259
8:133999440:A:C	8	133999440	0.014558252	3	1.200642195	0.254591569	4.715954269	2.4058E-06	intronic	TG	.	rs74591804	0.0132	0.0217	0.999662	1	chr8	132987195
12:11046142:C:A	12	11046142	0.03042233	6	1.581110295	0.336660743	4.696449854	2.64722E-06	ncRNA_intronic	PRH1-PRR4	.	rs111621289	0.0242	0.0300	0.810976	1	chr12	10893543
6:126490324:C:T	6	126490324	0.016917476	3	1.203746268	0.259271271	4.642806213	3.43709E-06	intergenic	MIR5695;CENPW	dist=46562;dist=170611	rs148294287	0.0107	0.0165	0.861892	1	chr6	126169178
6:31248568:G:A	6	31248568	0.024305825	5	1.53280058	0.332764823	4.606257864	4.0998E-06	intergenic	HLA-C;HLA-B	dist=8655;dist=73081	rs9380234	0.0429	0.0402	0.995714	1	chr6	31280791
6:31238801:C:G	6	31238801	0.024349515	5	1.533218592	0.332869958	4.606058777	4.10372E-06	intronic	HLA-C	.	rs41544614	0.0428	0.0404	0.994693	1	chr6	31271024
6:158989974:G:A	6	158989974	0.015582524	3	1.102833648	0.239463028	4.60544434	4.11586E-06	intronic	TMEM181	.	rs142006494	0.0339	0.0408	0.909469	1	chr6	158568942
6:31240096:G:A	6	31240096	0.024271845	5	1.534745688	0.333295989	4.604752947	4.12956E-06	upstream	HLA-C	dist=183	rs9366775	0.0429	0.0404	1	1	chr6	31272319
6:31240479:T:G	6	31240479	0.024271845	5	1.534745688	0.333295989	4.604752947	4.12956E-06	upstream	HLA-C	dist=566	rs9357121	0.0431	0.0406	1	1	chr6	31272702
6:31245080:G:A	6	31245080	0.024271845	5	1.534745688	0.333295989	4.604752947	4.12956E-06	intergenic	HLA-C;HLA-B	dist=5167;dist=76569	rs9391714	0.0430	0.0403	1	1	chr6	31277303
6:31247267:C:T	6	31247267	0.024237864	5	1.532381559	0.332833918	4.60404267	4.14368E-06	intergenic	HLA-C;HLA-B	dist=7354;dist=74382	rs56356836	0.0429	0.0402	0.998566	1	chr6	31279490
6:31247998:T:C	6	31247998	0.024237864	5	1.532381559	0.332833918	4.60404267	4.14368E-06	intergenic	HLA-C;HLA-B	dist=8085;dist=73651	rs9405016	0.0430	0.0403	0.998566	1	chr6	31280221
6:31248262:G:A	6	31248262	0.024237864	5	1.532381559	0.332833918	4.60404267	4.14368E-06	intergenic	HLA-C;HLA-B	dist=8349;dist=73387	rs12529015	0.0429	0.0401	0.998566	1	chr6	31280485
6:31248493:T:C	6	31248493	0.024237864	5	1.532381559	0.332833918	4.60404267	4.14368E-06	intergenic	HLA-C;HLA-B	dist=8580;dist=73156	rs9368669	0.0429	0.0402	0.998566	1	chr6	31280716
6:31256026:C:G	6	31256026	0.024237864	5	1.532381559	0.332833918	4.60404267	4.14368E-06	intergenic	HLA-C;HLA-B	dist=16113;dist=65623	rs17198734	0.0253	0.0303	0.998566	1	chr6	31288249
6:31256058:G:T	6	31256058	0.024237864	5	1.532381559	0.332833918	4.60404267	4.14368E-06	intergenic	HLA-C;HLA-B	dist=16145;dist=65591	rs17192386	0.0358	0.0305	0.998566	1	chr6	31288281
6:31238068:C:T	6	31238068	0.024432039	5	1.532787887	0.332980302	4.603238922	4.15971E-06	exonic	HLA-C	.	rs1050276	0.0426	0.0400	0.991508	1	chr6	31270291
6:31239727:C:A	6	31239727	0.024334952	5	1.529466801	0.332272783	4.603045682	4.16357E-06	intronic	HLA-C	.	rs29029490	0.0429	0.0404	0.991645	1	chr6	31271950
6:31240818:G:A	6	31240818	0.024203884	5	1.529478709	0.332384994	4.601527555	4.19404E-06	upstream	HLA-C	dist=905	rs4298345	0.0429	0.0404	0.997144	1	chr6	31273041

3:196625842:T:C	3	196625842	0.97179126	6	-1.52139796	0.331189308	-4.593741177	4.35369E-06	intronic	SENP5	.	rs6583185	0.9845	0.9781	0.853106	1	chr3	196898971
5:162496418:T:C	5	162496418	0.028330097	6	1.557183414	0.34021336	4.577078964	4.71514E-06	intergenic	GABRG2;CCNG1	dist=913873;dist=368159	rs114500719	0.0243	0.0327	0.909911	1	chr5	163069412
2:134644599:A:G	2	134644599	0.014786408	3	1.137571373	0.248782585	4.572552272	4.81819E-06	intergenic	NCKAP5;MIR3679	dist=318568;dist=240097	rs189440995	0.0060	0.0093	0.899627	1	chr2	133887028
10:72790290:G:A	10	72790290	0.038834951	8	1.881493073	0.411573362	4.571464641	4.84327E-06	intergenic	PCBD1;UNC5B	dist=141747;dist=182002	rs77364739	0.0865	0.0588	1	1	chr10	71030533
4:125382184:C:G	4	125382184	0.019456311	4	1.343334873	0.29403913	4.568558189	4.91091E-06	intergenic	LINC01091;LOC101927087	dist=530666;dist=38913	rs72678487	0.0073	0.0115	0.987952	1	chr4	124461029

Supplementary Table S8. GWAS Results Indianapolis-2 Replication Cohort
12 month quantitative trait (QT), P-value ordered

ID	chr	POS_37	freq	MAC	Score	Score.SE	Score.Stat	Score.pval	Func.refGene	Gene.refGene	GeneDetail.refGene	rsID	gnomAD_ genome_ALL	gnomAD_ genome_ NFE	Rsq	hwe	chr_38	POS_38
13:71228466:C:A	13	71228466	0.066820388	14	3.693410575	0.700908722	5.269460143	1.36826E-07	intergenic	ATXN8OS;LINC00348	dist=514581;dist=360807	rs9592740	0.0651	0.0859	0.979474	1	chr13	70654334
10:115449191:G:A	10	115449191	0.01631068	3	1.860675373	0.358119043	5.195689556	2.03962E-07	intronic	CASP7	.	rs150977401	0.0073	0.0111	0.870763	1	chr10	113689432
13:71242428:T:G	13	71242428	0.065	13	3.560256036	0.685315257	5.195063146	2.0465E-07	intergenic	ATXN8OS;LINC00348	dist=528543;dist=346845	rs9599709	0.0654	0.0860	0.958512	1	chr13	70668296
2:130563530:G:A	2	130563530	0.248378641	51	6.844310493	1.325444312	5.163785781	2.42005E-07	intergenic	LOC151121;LOC389033	dist=532066;dist=116905	rs4337430	0.2131	0.2653	0.973034	0.279636	chr2	129805957
2:151347581:A:G	2	151347581	0.013723301	3	1.731904782	0.335798226	5.157575737	2.50168E-07	intergenic	RND3;LOC101929260	dist=3372;dist=61465	rs145531574	0.0088	0.0141	0.8698	1	chr2	150491067
15:61531695:C:G	15	61531695	0.014752427	3	1.816785354	0.35534236	5.112774488	3.17461E-07	intergenic	RORA;VPS13C	dist=10193;dist=612895	rs143862437	0.0083	0.0139	0.9521	1	chr15	61239496
15:61534825:G:A	15	61534825	0.014762136	3	1.816594932	0.35534099	5.112258325	3.1833E-07	intergenic	RORA;VPS13C	dist=13323;dist=609765	rs182462684	0.0084	0.0138	0.951481	1	chr15	61242626
12:78530991:C:T	12	78530991	0.028985437	6	2.517383543	0.494187455	5.093985122	3.50614E-07	exonic	NAV3	.	rs61754236	0.0085	0.0125	0.995116	1	chr12	78137211
12:898065:G:T	12	898065	0.01511165	3	1.72959212	0.339602326	5.092992566	3.52456E-07	intronic	WNK1	.	rs2014160	0.0005	0.0007	0.853667	1	chr12	788899
12:78545400:G:A	12	78545400	0.02904369	6	2.519911214	0.495194249	5.088732794	3.60464E-07	intronic	NAV3	.	rs140686116	0.0063	0.0103	0.997108	1	chr12	78151620
8:39944917:T:A	8	39944917	0.030781554	6	2.577314922	0.506628696	5.087187008	3.63413E-07	intergenic	IDO2;C8orf4	dist=71007;dist=66070	rs138697513	0.0130	0.0201	0.884291	1	chr8	40087398
8:39944783:C:T	8	39944783	0.030776699	6	2.576605742	0.50654249	5.086652731	3.64438E-07	intergenic	IDO2;C8orf4	dist=70873;dist=66204	rs145892996	0.0131	0.0201	0.884119	1	chr8	40087264
2:130565338:C:A	2	130565338	0.253538835	52	6.70870463	1.334874447	5.025719568	5.01548E-07	intergenic	LOC151121;LOC389033	dist=533874;dist=115097	rs34017523	0.2123	0.2644	0.978903	0.420041	chr2	129807765
2:130567425:T:A	2	130567425	0.253538835	52	6.70870463	1.334874447	5.025719568	5.01548E-07	intergenic	LOC151121;LOC389033	dist=535961;dist=113010	rs6720381	0.2065	0.2652	0.978903	0.420041	chr2	129809852
2:130573252:C:A	2	130573252	0.254174757	52	6.714995733	1.337157087	5.021845077	5.11775E-07	intergenic	LOC151121;LOC389033	dist=541788;dist=107183	rs34895266	0.2140	0.2654	0.980963	0.420041	chr2	129815679
19:46480407:T:C	19	46480407	0.019417476	4	2.066073669	0.41173362	5.017986311	5.22159E-07	intergenic	NOVA2;CCDC61	dist=3750;dist=18312	rs4239537	0.0835	0.0109	1	1	chr19	45977149
22:19080386:G:A	22	19080386	0.024252427	5	2.149293074	0.429334633	5.006102254	5.55433E-07	intronic	DGCR2	.	rs150428353	0.0172	0.0230	0.783413	1	chr22	19092873
15:61567547:C:T	15	61567547	0.01557767	3	1.784212495	0.357897115	4.985266493	6.18765E-07	intergenic	RORA;VPS13C	dist=46045;dist=577043	rs144327698	0.0097	0.0159	0.916644	1	chr15	61275348
15:61555055:G:C	15	61555055	0.015490291	3	1.774737275	0.356244746	4.98179214	6.29981E-07	intergenic	RORA;VPS13C	dist=33553;dist=589535	rs143096638	0.0093	0.0154	0.913405	1	chr15	61262856
3:64559845:T:C	3	64559845	0.014699029	3	1.72126431	0.347407762	4.954593705	7.24816E-07	ncRNA_intronic	ADAMTS9-AS1	.	rs146268777	0.0046	0.0068	0.94425	1	chr3	64574169
2:130584142:C:T	2	130584142	0.222898058	46	6.124040336	1.238717369	4.943856031	7.65923E-07	intergenic	LOC151121;LOC389033	dist=552678;dist=96293	rs35748618	0.1481	0.2177	0.96226	1	chr2	129826569
5:73263379:A:G	5	73263379	0.024257281	5	2.247608363	0.454880737	4.941093741	7.76855E-07	intergenic	ARHGEF28;LINC01335	dist=25561;dist=338856	rs190652742	0.0104	0.0094	0.999387	1	chr5	73967554
2:130592585:C:T	2	130592585	0.258529127	53	6.558142102	1.329599407	4.932419546	8.12172E-07	intergenic	LOC151121;LOC389033	dist=561121;dist=87850	rs6742395	0.2225	0.2681	0.976222	0.430075	chr2	129835012
X:86181688:A:G	X	86181688	0.013970874	3	1.634757242	0.331433352	4.932386049	8.12312E-07	intergenic	DACH2;KLHL4	dist=94083;dist=591027	rs186620466	0.0162	0.0191	0.873035	1	chrX	86926685
10:21883439:G:A	10	21883439	0.015543689	3	1.645849191	0.333793955	4.93073396	8.19212E-07	intronic	MLLT10	.	rs191906004	0.0058	0.0077	0.843604	1	chr10	21594510
10:21970382:G:A	10	21970382	0.014849515	3	1.594035309	0.323294895	4.930592272	8.19807E-07	intronic	MLLT10	.	rs188790792	0.0052	0.0080	0.823859	1	chr10	21681453
2:130586642:C:T	2	130586642	0.257281553	53	6.683699411	1.358232147	4.920881476	8.61553E-07	intergenic	LOC151121;LOC389033	dist=555178;dist=93793	rs4277471	0.1953	0.2581	1	0.300358	chr2	129829069
1:53720723:T:C	1	53720723	0.040257281	8	2.387313586	0.485212014	4.920145248	8.648E-07	intronic	LRP8	.	rs12116501	0.0311	0.0437	0.807097	1	chr1	53255051
1:240998165:G:A	1	240998165	0.019203883	4	1.815512107	0.369826911	4.909085989	9.15019E-07	intronic	RGS7	.	rs72754890	0.0051	0.0073	0.806371	1	chr1	240834865
7:89853957:C:T	7	89853957	0.018815534	4	1.879423001	0.384569904	4.887077696	1.02344E-06	intronic	STEAP2	.	rs149916697	0.0065	0.0095	0.928405	1	chr7	90224643
7:89988476:T:C	7	89988476	0.018800971	4	1.89404647	0.387759417	4.8845918	1.03643E-06	intronic	GTPBP10	.	rs79847565	0.0069	0.0100	0.945469	1	chr7	90359162
7:90013529:G:A	7	90013529	0.018776699	4	1.910374378	0.392033885	4.872982793	1.09926E-06	intronic	GTPBP10	.	rs187685448	0.0067	0.0098	0.967919	1	chr7	90384215
2:130589408:A:T	2	130589408	0.263592233	54	6.506401141	1.335621499	4.871440856	1.10787E-06	intergenic	LOC151121;LOC389033	dist=557944;dist=91027	rs1882610	0.2367	0.2795	0.977378	0.601073	chr2	129831835
7:90047115:A:G	7	90047115	0.018825242	4	1.913164032	0.392804365	4.870526408	1.11301E-06	intergenic	CLDN12;CDK14	dist=1847;dist=178561	rs143425398	0.0066	0.0097	0.970376	1	chr7	90417801
2:130582408:G:C	2	130582408	0.264009709	54	6.522687428	1.33928955	4.87025933	1.11452E-06	intergenic	LOC151121;LOC389033	dist=550944;dist=98027	rs12621133	0.2291	0.2789	0.979973	0.601073	chr2	129824835
7:90253061:G:A	7	90253061	0.01892233	4	1.921233543	0.394605718	4.868742276	1.12311E-06	intronic	CDK14	.	rs150148213	0.0066	0.0097	0.973808	1	chr7	90623747
1:12215283:T:C	1	12215283	0.013373786	3	1.580945998	0.324859599	4.866551587	1.13562E-06	intergenic	TNFRSF8;MIR7846	dist=11019;dist=11717	rs147985761	0.0081	0.0132	0.894028	1	chr1	12155226
9:36357606:C:A	9	36357606	0.023582524	5	2.18417045	0.448849347	4.866154904	1.1379E-06	intronic	RNF38	.	rs4879986	0.0112	0.0117	0.970686	1	chr9	36357609
9:36381902:C:T	9	36381902	0.97573301	5	-2.242692534	0.461738278	-4.857064356	1.19139E-06	intronic	RNF38	.	rs2248297	0.9692	0.9879	0.999795	1	chr9	36381905
4:163976364:T:C	4	163976364	0.028898058	6	2.023347127	0.417548067	4.845782525	1.26114E-06	intergenic	FSTL5;MIR4454	dist=891178;dist=38362	rs146154846	0.0182	0.0245	0.711952	1	chr4	163055212
2:117348669:G:A	2	117348669	0.020179612	4	2.005609812	0.414444146	4.839276486	1.30313E-06	intergenic	DPP10;DDX18	dist=746343;dist=1223586	rs75685045	0.0065	0.0111	0.944983	1	chr2	116591093
9:36360845:T:C	9	36360845	0.024014563	5	2.210438405	0.456810098	4.838856266	1.30588E-06	intronic	RNF38	.	rs79319828	0.0116	0.0120	0.9887	1	chr9	36360848
9:36386865:T:G	9	36386865	0.024029126	5	2.210045419	0.456790677	4.838201677	1.31019E-06	intronic	RNF38	.	rs76774745	0.0117	0.0120	0.988086	1	chr9	36386868
17:43780479:T:C	17	43780479	0.0145	3	1.703938501	0.352883611	4.828613304	1.37487E-06	intronic	MGC57346-CRHR1	.	rs79985283	0.0211	0.0077	0.994938	1	chr17	45703113
10:72790290:G:A	10	72790290	0.038834951	8	2.690625403	0.557664123	4.824813526	1.40134E-06	intergenic	PCBD1;UNC5B	dist=141747;dist=182002	rs77364739	0.0865	0.0588	1	1	chr10	71030533
1:70886329:A:G	1	70886329	0.038961165	8	2.731688539	0.567885471	4.81028073	1.50718E-06	intronic	CTH	.	rs79934683	0.0306	0.0416	0.993133	1	chr1	70420646
20:6955818:G:T	20	6955818	0.017223301	4	1.82640918	0.380591181	4.798874146	1.5956E-06	intergenic	BMP2;LINC01428	dist=194893;dist=171296	rs146812516	0.0161	0.0210	0.862977	1	chr20	6975171
14:24001662:C:T	14	24001662	0.024456311	5	1.768121176	0.369052815	4.790970568	1.65976E-06	intronic	ZFHX2	.	rs41307102	0.0265	0.0375	0.636713	1	chr14	23532453
8:40049692:T:G	8	40049692	0.044912622	9	2.785140072	0.582245322	4.783447747	1.72314E-06	intergenic	C8orf4;ZMAT4	dist=36865;dist=338419	rs150470417	0.0259	0.0383	0.960377	1	chr8	40192173
8:40027101:T:C	8	40027101	0.04368932	9	2.810807743	0.588520937	4.776053945	1.78768E-06	intergenic	C8orf4;ZMAT4	dist=14274;dist=361010	rs116259399	0.0402	0.0401	1	1	chr8	40169582
5:5233724:G:C	5	5233724	0.014830097	3	1.706394641	0.358423256	4.760836832	1.92792E-06	intronic	ADAMTS16	.	rs115149137	0.0104	0.0175	0.967403	1	chr5	5233611
7:89801861:C:T	7	89801861	0.017533981	4	1.680077446	0.353443095	4.753459521	1.99965E-06	ncRNA_intronic	STEAP2-AS1	.	rs7800716	0.0069	0.0101	0.830555	1	chr7	90172547
4:40948164:G:A	4	40948164	0.037621359	8	2.919015192	0.615000485	4.746362423	2.07107E-06	intronic	APBB2	.	rs113189296	0.0591	0.0852	0.970482	1	chr4	40946147
1:240988921:C:T	1	240988921	0.020087379	4	1.824932693	0.38452179	4.74597992	2.07499E-06	intronic	RGS7	.	rs72754888	0.0047	0.0067	0.836433	1	chr1	240825621
5:5241203:C:A	5	5241203	0.014868932	3	1.68070787	0.35442199	4.742109459	2.11504E-06	intronic	ADAMTS16	.	rs145155685	0.0080	0.0129	0.944326	1	chr5	5241090
7:48396066:G:A	7	48396066	0.014519418	3	1.695949216	0.358675926	4.728360877	2.2634E-06	intronic	ABCA13	.	rs189164061	0.0077	0.0119	0.996283	1	chr7	48356469
11:44085975:C:A	11	44085975	0.014893204	3	1.699510358	0.360057984	4.720101852	2.35727E-06	intergenic	ACCSL;ACCS	dist=4448;dist=1754	rs76143420	0.0134	0.0215	0.971325	1	chr11	44064425

13:102628784:G:A	13	102628784	0.00926699	2	1.077369422	0.228342774	4.718211147	2.37927E-06	intronic	FGF14	.	rs75197268	0.0122	0.0178	0.53936	1	chr13	101976434
18:39394472:G:C	18	39394472	0.023024272	5	1.975849294	0.419524828	4.709731495	2.48043E-06	intergenic	KC6;PIK3C3	dist=293911;dist=140691	rs113484345	0.0229	0.0286	0.890728	1	chr18	41814507
4:173193727:G:A	4	173193727	0.014470874	3	1.644356357	0.349394212	4.706306808	2.52245E-06	intronic	GALNTL6	.	rs187567621	0.0019	0.0033	0.94795	1	chr4	172272576
10:98558540:C:A	10	98558540	0.013485437	3	1.29789842	0.276292266	4.697556103	2.63293E-06	intergenic	PIK3AP1;MIR607	dist=78261;dist=29886	rs61856855	0.0113	0.0141	0.63836	1	chr10	96798783
13:61672508:T:C	13	61672508	0.027004854	6	2.031117161	0.432525467	4.695948131	2.65373E-06	intergenic	LINC00378;MIR3169	dist=402574;dist=101424	rs147220861	0.0080	0.0082	0.826016	1	chr13	61098374
13:109580621:C:T	13	109580621	0.022786408	5	2.002149238	0.426532153	4.694017139	2.67892E-06	intronic	MYO16	.	rs145442123	0.0106	0.0163	0.930524	1	chr13	108928273
12:62144998:G:A	12	62144998	0.01723301	4	1.744080472	0.37159489	4.693499608	2.68571E-06	intronic	FAM19A2	.	rs76854078	0.0089	0.0135	0.904535	1	chr12	61751217
11:44073753:C:T	11	44073753	0.01565534	3	1.691379055	0.360755196	4.688439897	2.75296E-06	intronic	ACCSL	.	rs149030097	0.0134	0.0213	0.931546	1	chr11	44052203
4:164000229:C:T	4	164000229	0.02361165	5	1.814612257	0.387671579	4.680797751	2.85761E-06	intergenic	FSTL5;MIR4454	dist=915043;dist=14497	rs145034465	0.0184	0.0231	0.732815	1	chr4	163079077
10:72806820:G:A	10	72806820	0.036461165	8	2.445966686	0.523449162	4.672787474	2.97139E-06	intergenic	PCBD1;UNC5B	dist=158277;dist=165472	rs75505956	0.0366	0.0553	0.953209	1	chr10	71047063
17:52366146:G:A	17	52366146	0.023776699	5	1.836346208	0.394018804	4.660554743	3.15358E-06	intergenic	KIF2B;TOM1L1	dist=463573;dist=611906	rs62072692	0.0232	0.0371	0.819939	1	chr17	54288785
2:130568556:T:C	2	130568556	0.297684466	61	6.436745057	1.381972449	4.657650781	3.19838E-06	intergenic	LOC151121;LOC389033	dist=537092;dist=111879	rs4355062	0.2389	0.2895	0.981992	0.478458	chr2	129810983
2:72629234:T:G	2	72629234	0.018174757	4	1.80424283	0.387623961	4.654621515	3.24576E-06	intronic	EXOC6B	.	rs185879164	0.0118	0.0192	0.929494	1	chr2	72402105
10:21749029:G:A	10	21749029	0.011912621	2	1.22791161	0.263818829	4.654374424	3.24966E-06	intergenic	NEBL-AS1;CASC10	dist=285177;dist=34392	rs183818481	0.0059	0.0081	0.674237	1	chr10	21460100
15:45198007:A:C	15	45198007	0.048796117	10	2.632234639	0.565845842	4.651858229	3.28957E-06	intergenic	TRIM69;C15orf43	dist=137980;dist=50893	rs28631454	0.3216	0.1096	0.89064	1	chr15	44905809
20:45720309:A:G	20	45720309	0.032533981	7	2.316388446	0.498848234	4.643473279	3.426E-06	intronic	EYA2	.	rs146684353	0.0232	0.0301	0.918602	1	chr20	47091670
6:77472188:A:G	6	77472188	0.019490291	4	1.93070443	0.415892439	4.642316741	3.44524E-06	intergenic	IMPG1;HTR1B	dist=689793;dist=698377	rs55850402	0.0070	0.0099	0.988316	1	chr6	76762471
6:77460389:C:T	6	77460389	0.019490291	4	1.930446837	0.415888081	4.641745998	3.45477E-06	intergenic	IMPG1;HTR1B	dist=677994;dist=710176	rs191563430	0.0071	0.0101	0.988316	1	chr6	76750672
2:72852782:C:A	2	72852782	0.018898058	4	1.818657772	0.391871559	4.640953721	3.46805E-06	intronic	EXOC6B	.	rs115540332	0.0116	0.0188	0.914669	1	chr2	72625653
5:53390040:G:C	5	53390040	0.306907767	63	6.436279804	1.389496475	4.632095094	3.61984E-06	intronic	ARL15	.	rs42872	0.2490	0.2995	0.980711	0.812933	chr5	54094210
X:20935027:G:A	X	20935027	0.015194175	3	1.517881729	0.327834067	4.630030494	3.65612E-06	intergenic	RPS6KA3;CNKSR2	dist=650277;dist=457509	rs144178739	0.0231	0.0199	0.814532	1	chrX	20916909
5:33636035:T:C	5	33636035	0.155339806	32	5.112931608	1.104845474	4.627734581	3.69687E-06	intronic	ADAMTS12	.	rs13164825	0.1577	0.1968	1	0.705556	chr5	33635930
4:31782754:A:G	4	31782754	0.053990291	11	3.25806796	0.70424346	4.626337545	3.72189E-06	intergenic	LOC102723778;LOC102723828	dist=569458;dist=216247	rs79231845	0.0271	0.0314	0.964283	1	chr4	31781132
6:77429812:T:G	6	77429812	0.019640777	4	1.923001054	0.415738055	4.625511258	3.73676E-06	intergenic	IMPG1;HTR1B	dist=647417;dist=740753	rs77029592	0.0083	0.0100	0.980847	1	chr6	76720095
10:22083617:A:G	10	22083617	0.011519417	2	1.283845844	0.277566907	4.625356308	3.73955E-06	intronic	DNAJC1	.	rs181139900	0.0064	0.0085	0.775781	1	chr10	21794688
5:33646456:G:C	5	33646456	0.164631068	34	4.973632977	1.076119596	4.621821771	3.80385E-06	intronic	ADAMTS12	.	rs10057508	0.1583	0.2157	0.991904	1	chr5	33646351
6:77420028:C:A	6	77420028	0.019699029	4	1.920385778	0.415757928	4.618999777	3.85594E-06	intergenic	IMPG1;HTR1B	dist=637633;dist=750537	rs78549750	0.0160	0.0101	0.978334	1	chr6	76710311
6:77414670:G:C	6	77414670	0.019820388	4	1.925532437	0.417298507	4.614280674	3.94459E-06	intergenic	IMPG1;HTR1B	dist=632275;dist=755895	rs56232065	0.0103	0.0099	0.980026	1	chr6	76704953
5:33647634:G:A	5	33647634	0.159796116	33	4.948363002	1.073508457	4.609524006	4.03592E-06	intronic	ADAMTS12	.	rs13176485	0.1519	0.2149	0.991564	1	chr5	33647529
5:33644123:G:T	5	33644123	0.15973301	33	4.946113148	1.073435357	4.60774197	4.07065E-06	intronic	ADAMTS12	.	rs35388476	0.1520	0.2154	0.992101	1	chr5	33644018
21:35375126:G:A	21	35375126	0.007194175	1	0.846200633	0.183711704	4.606133495	4.10225E-06	intergenic	LINC00649;MRPS6	dist=38864;dist=70697	rs144499025	0.0048	0.0076	0.538813	1	chr21	34002825
11:21775222:T:A	11	21775222	0.012529126	3	1.341788897	0.291618921	4.601172279	4.2012E-06	intergenic	NELL1;ANO5	dist=177993;dist=439500	rs147118669	0.0050	0.0085	0.79684	1	chr11	21753676
10:22068468:C:A	10	22068468	0.011684466	2	1.295186101	0.281543412	4.600306895	4.21869E-06	intronic	DNAJC1	.	rs184920540	0.0059	0.0079	0.788199	1	chr10	21779539
5:33644614:T:C	5	33644614	0.160194175	33	4.958247799	1.078142852	4.598878331	4.24772E-06	intronic	ADAMTS12	.	rs67267661	0.1519	0.2153	1	1	chr5	33644509
4:31766001:G:A	4	31766001	0.053223301	11	3.28160902	0.713701131	4.598015718	4.26534E-06	intergenic	LOC102723778;LOC102723828	dist=552705;dist=233000	rs16885796	0.0382	0.0334	0.994282	1	chr4	31764379
2:66301595:G:A	2	66301595	0.024257282	5	2.107857413	0.458486846	4.597421782	4.27751E-06	intergenic	SPRED2;MIR4778	dist=641939;dist=283786	rs11676376	0.0435	0.0423	0.998566	1	chr2	66074461
10:22216057:G:C	10	22216057	0.013985437	3	1.470044503	0.319890854	4.595456497	4.31803E-06	intronic	DNAJC1	.	rs45496395	0.0063	0.0084	0.8731	1	chr10	21927128
4:31779863:T:C	4	31779863	0.053402913	11	3.29083276	0.717091233	4.589140975	4.45074E-06	intergenic	LOC102723778;LOC102723828	dist=566567;dist=219138	rs115916744	0.0283	0.0312	0.999904	0.247516	chr4	31778241
4:31772884:C:T	4	31772884	0.053398058	11	3.290738644	0.717096412	4.588976583	4.45424E-06	intergenic	LOC102723778;LOC102723828	dist=559588;dist=226117	rs116957173	0.0270	0.0312	1	0.245321	chr4	31771262
5:3562095:T:A	5	3562095	0.00734466	2	0.983175268	0.214354222	4.58668487	4.5034E-06	intergenic	LINC01019;IRX1	dist=25887;dist=34073	rs143503046	0.0027	0.0041	0.686588	1	chr5	3561981
4:31777594:G:A	4	31777594	0.053165049	11	3.264991759	0.71189775	4.586321223	4.51124E-06	intergenic	LOC102723778;LOC102723828	dist=564298;dist=221407	rs116168704	0.0285	0.0314	0.991203	1	chr4	31775972
1:18614493:G:T	1	18614493	0.014364078	3	1.46894969	0.320848564	4.578327141	4.68709E-06	intronic	IGSF21	.	rs115263712	0.0101	0.0137	0.8337	1	chr1	18287999
4:189389929:T:C	4	189389929	0.010296117	2	1.359541054	0.297050321	4.576803849	4.72134E-06	ncRNA_intronic	LINC01060	.	rs560030667	0.0037	0.0047	0.944465	1	chr4	188468775
4:189389927:T:C	4	189389927	0.010276699	2	1.359541791	0.297112351	4.575850811	4.74288E-06	ncRNA_intronic	LINC01060	.	rs544973300	0.0035	0.0050	0.946406	1	chr4	188468773
5:112013914:G:A	5	112013914	0.980427185	4	-1.381862916	0.302073383	-4.574593434	4.77146E-06	ncRNA_intronic	LOC102467216	.	rs12153142	0.9143	0.9991	0.499216	1	chr5	112678217

Supplementary Table S8. GWAS Results Indianapolis-2 Replication Cohort
12 month quantitative trait (QT), risk locus ordered

Shaded cells in Gene.refGene column indicate risk loci that co-localize with risk loci identified in the Indianapolis-1 GWAS

ID_37	chr_37	POS_37	rsID	chr_38	POS_38	freq	MAC	Score	Score.SE	Score.Stat	Score.pval	Func.refGene	Gene.refGene	GeneDetail.refGene	AOP-expressed refGene (protein coding only)	gnomAD_genome_ALL	gnomAD_genome_NFE	Rsq	hwe	AOP-expressed gene name
2:50062774:T:G	2	50062774	rs138665118	chr2	49835636	0.03086408	6	1.721021507	0.35697746	4.821092882	1.42774E-06	intergenic	FSHR;NRXN1	dist=681108;dist=82869	NRXN1	0.0114	0.0171	0.892953	1	Neurexin 1
2:74482548:G:T	2	74482548	rs12621729	chr2	74255421	0.03220874	7	1.780987998	0.37313777	4.77300378	1.81498E-06	intronic	SLC4A5	.	SLC4A5	0.1479	0.0335	0.942493	1	Solute Carrier Family 4 Member 5 (sodium bicarbonate transporter)
2:126913427:C:G	2	126913427	rs28798228	chr2	126155850	0.03317476	7	1.808450116	0.37851254	4.777781303	1.7724E-06	intergenic	CNTNAP5;GYPC	dist=1240473;dist=500084	GYPC	0.0111	0.0144	0.961063	1	Glycophorin C (Gerbich Blood Group)
2:126955214:G:A	2	126955214	rs28796094	chr2	126197637	0.03244175	7	1.7618909	0.37073145	4.752472211	2.00944E-06	intergenic	CNTNAP5;GYPC	dist=1282260;dist=458297		0.0111	0.0144	0.939443	1	
2:134644599:A:G	2	134644599	rs189440995	chr2	133887028	0.01478641	3	1.137571373	0.24878258	4.572552272	4.81819E-06	intergenic	NCKAP5;MIR3679	dist=318568;dist=240097	NCKAP5	0.0060	0.0093	0.899627	1	NCK Associated Protein 5
3:75482532:G:C	3	75482532	rs185293133	chr3	75433381	0.01162621	2	0.80016771	0.15901908	5.031897471	4.85649E-07	ncRNA_intronic	FAM86DP	.		0.0054	0.0078	0.492536	1	
3:146048618:G:A	3	146048618	rs73150883	chr3	146330831	0.02381553	5	1.481987222	0.28774985	5.150262284	2.60122E-07	intergenic	PLSCR4;PLSCR2	dist=79652;dist=102457	PLSCR4;PLSCR2	0.0163	0.0252	0.848249	1	Phospholipid Scramblase 4;Phospholipid Scramblase 2
3:196625842:T:C	3	196625842	rs6583185	chr3	196898971	0.97179126	6	-1.52139796	0.33118931	-4.593741177	4.35369E-06	intronic	SENP5	.	SENP5	0.9845	0.9781	0.853106	1	SUMO Specific Peptidase 5
4:125382184:C:G	4	125382184	rs72678487	chr4	124461029	0.01945631	4	1.343334873	0.29403913	4.568558189	4.91091E-06	intergenic	LINC01091;LOC101927087	dist=530666;dist=38913		0.0073	0.0115	0.987952	1	
5:115976909:A:C	5	115976909	rs76433992	chr5	116641213	0.02983495	6	1.764770451	0.36047037	4.895743415	9.79348E-07	intergenic	SEMA6A;LOC102467223	dist=66287;dist=102089	SEMA6A	0.0168	0.0226	0.974211	1	Semaphorin 6A
5:115979852:T:C	5	115979852	rs114241096	chr5	116644156	0.02909223	6	1.782650366	0.3612333	4.934900476	8.01916E-07	intergenic	SEMA6A;LOC102467223	dist=69230;dist=99146		0.0185	0.0229	0.998458	1	
5:115980038:G:C	5	115980038	rs114587121	chr5	116644342	0.02909223	6	1.782650366	0.3612333	4.934900476	8.01916E-07	intergenic	SEMA6A;LOC102467223	dist=69416;dist=98960		0.0185	0.0229	0.998458	1	
5:115980724:A:G	5	115980724	rs79516960	chr5	116645028	0.02909709	6	1.782403345	0.36123109	4.934246796	8.04606E-07	intergenic	SEMA6A;LOC102467223	dist=70102;dist=98274		0.0171	0.0229	0.998286	1	
5:115980901:A:C	5	115980901	rs78085382	chr5	116645205	0.02909709	6	1.782403345	0.36123109	4.934246796	8.04606E-07	intergenic	SEMA6A;LOC102467223	dist=70279;dist=98097		0.0186	0.0228	0.998286	1	
5:115981236:C:G	5	115981236	rs79794218	chr5	116645540	0.02909709	6	1.782403345	0.36123109	4.934246796	8.04606E-07	intergenic	SEMA6A;LOC102467223	dist=70614;dist=97762		0.0170	0.0229	0.998286	1	
5:115981990:C:G	5	115981990	rs114108437	chr5	116646294	0.02909709	6	1.782403345	0.36123109	4.934246796	8.04606E-07	intergenic	SEMA6A;LOC102467223	dist=71368;dist=97008		0.0170	0.0229	0.998286	1	
5:115988180:G:A	5	115988180	rs77857559	chr5	116652484	0.02912621	6	1.784300998	0.36172467	4.932759998	8.10758E-07	intergenic	SEMA6A;LOC102467223	dist=77558;dist=90818		0.0165	0.0221	1	1	
5:115988843:C:T	5	115988843	rs115917626	chr5	116653147	0.02912621	6	1.784300998	0.36172467	4.932759998	8.10758E-07	intergenic	SEMA6A;LOC102467223	dist=78221;dist=90155		0.0167	0.0223	1	1	
5:115991913:A:T	5	115991913	rs76006987	chr5	116656217	0.02908738	6	1.782276135	0.36123386	4.933856736	8.06216E-07	intergenic	SEMA6A;LOC102467223	dist=81291;dist=87085		0.0185	0.0225	0.998629	1	
5:162496418:T:C	5	162496418	rs114500719	chr5	163069412	0.0283301	6	1.557183414	0.34021336	4.577078964	4.71514E-06	intergenic	GABRG2;CCNG1	dist=913873;dist=368159	GABRG2	0.0243	0.0327	0.909911	1	Gamma-Aminobutyric Acid Type A Receptor Subunit Gamma2
5:162629778:G:A	5	162629778	rs116189826	chr5	163202772	0.02453883	5	1.5321512	0.32364261	4.734083693	2.20047E-06	intergenic	GABRG2;CCNG1	dist=1047233;dist=234799	CCNG1	0.0218	0.0286	0.937899	1	Cyclin G1
5:162646718:G:C	5	162646718	rs115331238	chr5	163219712	0.02446116	5	1.583713858	0.33185801	4.772263512	1.82167E-06	intergenic	GABRG2;CCNG1	dist=1064173;dist=217859		0.0236	0.0306	0.983743	1	
5:162653096:T:C	5	162653096	rs116626718	chr5	163226090	0.02438835	5	1.593867941	0.33347822	4.779526333	1.75709E-06	intergenic	GABRG2;CCNG1	dist=1070551;dist=211481		0.0249	0.0325	0.995221	1	
5:162661735:C:T	5	162661735	rs114008181	chr5	163234729	0.02521359	5	1.5722456	0.33187599	4.73744913	2.16425E-06	intergenic	GABRG2;CCNG1	dist=1079190;dist=202842		0.0256	0.0337	0.958521	1	
6:31238068:C:T	6	31238068	rs1050276	chr6	31270291	0.02443204	5	1.532787887	0.3329803	4.603238922	4.15971E-06	exonic	HLA-C	.		0.0426	0.0400	0.991508	1	
6:31238801:C:G	6	31238801	rs41544614	chr6	31271024	0.02434951	5	1.533218592	0.33286996	4.606058777	4.10372E-06	intronic	HLA-C	.		0.0428	0.0404	0.994693	1	
6:31239727:C:A	6	31239727	rs29029490	chr6	31271950	0.02433495	5	1.529466801	0.33227278	4.603045682	4.16357E-06	intronic	HLA-C	.		0.0429	0.0404	0.991645	1	
6:31240096:G:A	6	31240096	rs9366775	chr6	31272319	0.02427184	5	1.534745688	0.33329599	4.604752947	4.12956E-06	upstream	HLA-C	dist=183		0.0429	0.0404	1	1	
6:31240479:T:G	6	31240479	rs9357121	chr6	31272702	0.02427184	5	1.534745688	0.33329599	4.604752947	4.12956E-06	upstream	HLA-C	dist=566		0.0431	0.0406	1	1	
6:31240818:G:A	6	31240818	rs4298345	chr6	31273041	0.02420388	5	1.529478709	0.33238499	4.601527555	4.19404E-06	upstream	HLA-C	dist=905		0.0429	0.0404	0.997144	1	
6:31245080:G:A	6	31245080	rs9391714	chr6	31277303	0.02427184	5	1.534745688	0.33329599	4.604752947	4.12956E-06	intergenic	HLA-C;HLA-B	dist=5167;dist=76569	HLA-C	0.0430	0.0403	1	1	Major Histocompatibility Complex, Class I, C
6:31247267:C:T	6	31247267	rs56356836	chr6	31279490	0.02423786	5	1.532381559	0.33283392	4.60404267	4.14368E-06	intergenic	HLA-C;HLA-B	dist=7354;dist=74382	HLA-B	0.0429	0.0402	0.998566	1	Major Histocompatibility Complex, Class I, B
6:31247998:T:C	6	31247998	rs9405016	chr6	31280221	0.02423786	5	1.532381559	0.33283392	4.60404267	4.14368E-06	intergenic	HLA-C;HLA-B	dist=8085;dist=73651		0.0430	0.0403	0.998566	1	
6:31248262:G:A	6	31248262	rs12529015	chr6	31280485	0.02423786	5	1.532381559	0.33283392	4.60404267	4.14368E-06	intergenic	HLA-C;HLA-B	dist=8349;dist=73387		0.0429	0.0401	0.998566	1	
6:31248493:T:C	6	31248493	rs9368669	chr6	31280716	0.02423786	5	1.532381559	0.33283392	4.60404267	4.14368E-06	intergenic	HLA-C;HLA-B	dist=8580;dist=73156		0.0429	0.0402	0.998566	1	
6:31248568:G:A	6	31248568	rs9380234	chr6	31280791	0.02430583	5	1.53280058	0.33276482	4.606257864	4.0998E-06	intergenic	HLA-C;HLA-B	dist=8655;dist=73081		0.0429	0.0402	0.995714	1	
6:31256026:C:G	6	31256026	rs17198734	chr6	31288249	0.02423786	5	1.532381559	0.33283392	4.60404267	4.14368E-06	intergenic	HLA-C;HLA-B	dist=16113;dist=65623		0.0253	0.0303	0.998566	1	
6:31256058:G:T	6	31256058	rs17192386	chr6	31288281	0.02423786	5	1.532381559	0.33283392	4.60404267	4.14368E-06	intergenic	HLA-C;HLA-B	dist=16145;dist=65591		0.0358	0.0305	0.998566	1	
6:126490324:C:T	6	126490324	rs148294287	chr6	126169178	0.01691748	3	1.203746268	0.25927127	4.642806213	3.43709E-06	intergenic	MIR5695;CENPW	dist=46562;dist=170611	CENPW	0.0107	0.0165	0.861892	1	Centromere Protein W
6:158989974:G:A	6	158989974	rs142006494	chr6	158568942	0.01558252	3	1.102833648	0.23946303	4.60544434	4.11586E-06	intronic	TMEM181	.	TMEM181	0.0339	0.0408	0.909469	1	Transmembrane Protein 181
7:9489611:T:G	7	9489611	rs17206867	chr7	9449981	0.01978155	4	1.317750936	0.27773373	4.744655705	2.08861E-06	intergenic	NXPH1;PER4	dist=697018;dist=184289		0.0067	0.0108	0.886061	1	
8:23462708:A:G	8	23462708	rs539324287	chr8	23605195	0.01694175	3	1.341800652	0.26460113	5.071031363	3.95666E-07	intergenic	SLC25A37;NKX3-1	dist=32645;dist=73498	SLC25A37	0.0033	0.0057	0.907302	1	Solute Carrier Family 25 Member 37 (mitochondrial)
8:23542722:T:A	8	23542722	rs189490695	chr8	23685209	0.0163835	3	1.2982161	0.2556875	5.077354608	3.82726E-07	intergenic	NKX3-1;NKX2-6	dist=2272;dist=17242	NKX3-1	0.0070	0.0092	0.874937	1	NK3 Homeobox 1
8:133999440:A:C	8	133999440	rs74591804	chr8	132987195	0.01455825	3	1.200642195	0.25459157	4.715954269	2.4058E-06	intronic	TG	.	TG	0.0132	0.0217	0.999662	1	Thyroglobulin
10:72790290:G:A	10	72790290	rs77364739	chr10	71030533	0.03883495	8	1.881493073	0.41157336	4.571464641	4.84327E-06	intergenic	PCBD1;UNC5B	dist=141747;dist=182002	PCBD1;UNC5B	0.0865	0.0588	1	1	Pterin-4 Alpha-Carbinolamine Dehydratase 1;Unc-5 Netrin Recepto
12:11046142:C:A	12	11046142	rs111621289	chr12	10893543	0.03042233	6	1.581110295	0.33666074	4.696449854	2.64722E-06	ncRNA_intronic	PRH1-PRR4	.		0.0242	0.0300	0.810976	1	
13:30768424:G:A	13	30768424	rs74833295	chr13	30194287	0.02916505	6	1.909228275	0.36002172	5.303091934	1.13858E-07	intergenic	LINC00365;KATNAL1	dist=85412;dist=8343	KATNAL1	0.0153	0.0232	0.9983	1	Katanin Catalytic Subunit A1 Like 1
13:52703883:C:G	13	52703883	rs148654242	chr13	52129747	0.03606311	7	1.896523445	0.38630706	4.90936778	9.13705E-07	ncRNA_exonic	LOC101929657	.		0.0202	0.0302	0.890541	1	
15:27633984:A:C	15	27633984	rs72705708	chr15	27388838	0.06847087	14	2.432058671	0.51344077	4.736785214	2.17135E-06	intronic	GABRG3	.	GABRG3	0.0450	0.0491	0.990527	1	Gamma-Aminobutyric Acid Type A Receptor Subunit Gamma3
15:27634215:C:T	15	27634215	rs72705709	chr15	27389069	0.06834952	14	2.429618929	0.5133886	4.732514355	2.21756E-06	intronic	GABRG3	.		0.0448	0.0489	0.992399	1	
15:27634405:G:C	15	27634405	rs72705710	chr15	27389259	0.06832039	14	2.428373305	0.51325152	4.731351398	2.2303E-06	intronic	GABRG3	.		0.0448	0.0489	0.992701	1	
15:27635356:C:G	15	27635356	rs72705711	chr15	27390210	0.06797087	14	2.443670474	0.51382691	4.75582426	1.97638E-06	intronic	GABRG3	.		0.0601	0.0498	0.997858	1	
15:27636387:G:A	15	27636387	rs72705712	chr15	27391241	0.06796117	14	2.444243265	0.51389229	4.756333777	1.9714E-06	intronic	GABRG3	.		0.0463	0.0496	0.998164	1	
15:27636953:T:G	15	27636953	rs72705714	chr15	27391807	0.06796117	14	2.448622014	0.51441141	4.760046044	1.93549E-06	intronic	GABRG3	.		0.0522	0.0496	1	1	
15:27637194:T:C	15	27637194	rs72705715	chr15	27392048	0.06796117	14	2.444243265	0.51389229	4.756333777	1.9714E-06	intronic	GABRG3	.		0.0428	0.0496	0.998164	1	
15:31383118:A:C	15	31383118	rs75968509	chr15	31090915	0.01935922	4	1.431828899	0.30212194	4.739241691	2.1452E-06	intronic	TRPM1	.	TRPM1	0.0124	0.0149	0.979554	1	Transient Receptor Potential Cation Channel Subfamily M Member
17:75157900:C:T	17	75157900	rs113199364	chr17	77161818	0.03330097	7	1.815437814	0.35840665	5.065301631	4.07754E-07	intronic	SEC14L1	.	SEC14L1	0.0230	0.0364	0.893794	1	SEC14 Like Lipid Binding 1
18:6045905:T:A	18	6045905	rs74649788	chr18	6045906	0.0498932	10	2.439599988	0.44900137	5.43339095	5.52931E-08	intronic	L3MBTL4	.	L3MBTL4	0.0811	0.0475	0.923243	1	L3MBTL Histone Methyl-Lysine Binding Protein 4
18:6049026:T:A	18	6049026	rs79381542	chr18	6049027	0.04435922	9	2.087445553	0.41784921	4.995691057	5.86254E-07	intronic	L3MBTL4	.		0.0807	0.0470	0.959092	1	
18:6049551:C:A	18	6049551	rs58503971	chr18	6049552	0.04434952	9	2.087223432	0.41779888	4.995761149	5.86042E-07	intronic	L3MBTL4	.		0.0786	0.0441	0.959099	1	

25 risk loci
one overlaps with 3 month: PCBD1;UNC5B
Thus 57+25-1 = 81 total risk loci
Gray cells = risk loci overlapping with Indianapolis-1 GWAS; 4/: 16.00%

Supplementary Table S8. GWAS Results Indianapolis-2 Replication Cohort
3 month quantitative trait (QT), risk locus ordered

Shaded cells in Gene.refGene column indicate risk loci that co-localize with risk loci identified in the Indianapolis-1 GWAS

ID_37	chr_37	POS_37	rsID	chr_38	POS_38	freq	MAC	Score	Score.SE	Score.Stat	Score.pval	Func.refGene	Gene.refGene	GeneDetail.refGene	AOP-expressed refGene (protein coding only)	gnomAD_ genome_ALL	gnomAD_ genome_ NFE	Rsq	hwe	AOP-expressed gene name
1:12215283:T:C	1	12215283	rs147985761	chr1	12155226	0.013373786	3	1.580945998	0.324859599	4.866551587	1.13562E-06	intergenic	TNFRSF8;MIR7846	dist=11019;dist=11717	TNFRSF8	0.0081	0.0132	0.894028	1	TNF Receptor Superfamily Member 8
1:18614493:G:T	1	18614493	rs115263712	chr1	18287999	0.014364078	3	1.46894969	0.320848564	4.578327141	4.68709E-06	intronic	IGSF21	.	IGSF21	0.0101	0.0137	0.8337	1	Immunoglobin Superfamily Member 21
1:53720723:T:C	1	53720723	rs12116501	chr1	53255051	0.040257281	8	2.387313586	0.485212014	4.920145248	8.648E-07	intronic	LRP8	.	LRP8	0.0311	0.0437	0.807097	1	LDL Receptor Related Protein 8
1:70886329:A:G	1	70886329	rs79934683	chr1	70420646	0.038981165	8	2.731688539	0.567885471	4.81028073	1.50718E-06	intronic	CTH	.	CTH	0.0306	0.0416	0.993133	1	Cystathionine Gamma-Lyase
1:240988921:C:T	1	240988921	rs72754888	chr1	240825621	0.020087379	4	1.824932693	0.38452179	4.74597992	2.07499E-06	intronic	RGS7	.	RGS7	0.0047	0.0067	0.836433	1	Regulator Of G Protein Signaling 7
1:240998165:G:A	1	240998165	rs72754890	chr1	240834865	0.019203883	4	1.815512107	0.369826911	4.909085989	9.15019E-07	intronic	RGS7	.		0.0051	0.0073	0.806371	1	
2:66301595:G:A	2	66301595	rs11676376	chr2	66074461	0.024257282	5	2.107857413	0.458486846	4.597421782	4.27751E-06	intergenic	SPRED2;MIR4778	dist=641939;dist=283786	SPRED2	0.0435	0.0423	0.998566	1	Sprouty Related EVH1 Domain Containing 2
2:72629234:T:G	2	72629234	rs185879164	chr2	72402105	0.018174757	4	1.80424283	0.387623961	4.654621515	3.24576E-06	intronic	EXOC6B	.	EXOC6B	0.0118	0.0192	0.929494	1	Exocyst Complex Component 6B (exocytosis)
2:72852782:C:A	2	72852782	rs115540332	chr2	72625653	0.018898058	4	1.818657772	0.391871559	4.640953721	3.46805E-06	intronic	EXOC6B	.		0.0116	0.0188	0.914669	1	
2:117348669:G:A	2	117348669	rs75685045	chr2	116591093	0.020179612	4	2.005609812	0.414444146	4.839276486	1.30313E-06	intergenic	DPP10;DDX18	dist=746343;dist=1223586	DPP10;DDX18	0.0065	0.0111	0.944983	1	Dipeptidyl Peptidase Like 10 (modifies KCND3); DEAD-Box Helicase 18
2:130563530:G:A	2	130563530	rs4337430	chr2	129805957	0.248378641	51	6.844310493	1.325444312	5.163785781	2.42005E-07	intergenic	LOC151121;LOC389033	dist=532066;dist=116905		0.2131	0.2653	0.973034	0.279636	
2:130565338:C:A	2	130565338	rs34017523	chr2	129807765	0.255338835	52	6.70870463	1.334874447	5.025719568	5.01548E-07	intergenic	LOC151121;LOC389033	dist=533874;dist=115097		0.2123	0.2644	0.978903	0.420041	
2:130567425:T:A	2	130567425	rs6720381	chr2	129809852	0.253538835	52	6.70870463	1.334874447	5.025719568	5.01548E-07	intergenic	LOC151121;LOC389033	dist=535961;dist=113010		0.2065	0.2652	0.978903	0.420041	
2:130568556:T:C	2	130568556	rs4355062	chr2	129810983	0.297684466	61	6.436745057	1.381972449	4.657650781	3.19838E-06	intergenic	LOC151121;LOC389033	dist=537092;dist=111879		0.2389	0.2895	0.981992	0.478458	
2:130573252:C:A	2	130573252	rs34895266	chr2	129815679	0.254174757	52	6.714995733	1.337157087	5.021845077	5.11775E-07	intergenic	LOC151121;LOC389033	dist=541788;dist=107183		0.2140	0.2654	0.980963	0.420041	
2:130582408:G:C	2	130582408	rs12621133	chr2	129824835	0.264009709	54	6.522687428	1.33928955	4.87025933	1.11452E-06	intergenic	LOC151121;LOC389033	dist=550944;dist=98027		0.2291	0.2789	0.979973	0.601073	
2:130584142:C:T	2	130584142	rs35748618	chr2	129826569	0.222898058	46	6.124040336	1.238717369	4.943856031	7.65923E-07	intergenic	LOC151121;LOC389033	dist=552678;dist=96293		0.1481	0.2177	0.96226	1	
2:130586642:C:T	2	130586642	rs4277471	chr2	129829069	0.257281553	53	6.683699411	1.358232147	4.920881476	8.61553E-07	intergenic	LOC151121;LOC389033	dist=555178;dist=93793		0.1953	0.2581	1	0.300358	
2:130589408:A:T	2	130589408	rs1882610	chr2	129831835	0.263592233	54	6.506401141	1.335621499	4.871440856	1.10787E-06	intergenic	LOC151121;LOC389033	dist=557944;dist=91027		0.2367	0.2795	0.977378	0.601073	
2:130592585:C:T	2	130592585	rs6742395	chr2	129835012	0.258529127	53	6.558142102	1.329599407	4.932419546	8.12172E-07	intergenic	LOC151121;LOC389033	dist=561121;dist=87850		0.2225	0.2681	0.976222	0.430075	
2:151347581:A:G	2	151347581	rs145531574	chr2	150491067	0.013723301	3	1.731904782	0.335798226	5.157575737	2.50168E-07	intergenic	RND3;LOC101929260	dist=3372;dist=61465	RND3	0.0088	0.0141	0.8698	1	Rho Family GTPase 3
3:64559845:T:C	3	64559845	rs146268777	chr3	64574169	0.014699029	3	1.72126431	0.347407762	4.954593705	7.24816E-07	ncRNA_intronic	ADAMTS9-AS1	.	ADAMTS9	0.0046	0.0068	0.94425	1	ADAMTS9
4:31766001:G:A	4	31766001	rs16885796	chr4	31764379	0.053223301	11	3.28160902	0.713701131	4.598015718	4.26534E-06	intergenic	LOC102723778;LOC102723828	dist=552705;dist=233000		0.0382	0.0334	0.994282	1	
4:31772884:C:T	4	31772884	rs116957173	chr4	31771262	0.053398058	11	3.290738644	0.717096412	4.588976583	4.45424E-06	intergenic	LOC102723778;LOC102723828	dist=559588;dist=226117		0.0270	0.0312	1	0.245321	
4:31777594:G:A	4	31777594	rs116168704	chr4	31775972	0.053165049	11	3.264991759	0.71189775	4.586321223	4.51124E-06	intergenic	LOC102723778;LOC102723828	dist=564298;dist=221407		0.0285	0.0314	0.991203	1	
4:31779863:T:C	4	31779863	rs115916744	chr4	31778241	0.053402913	11	3.29083276	0.717091233	4.589140975	4.45074E-06	intergenic	LOC102723778;LOC102723828	dist=566567;dist=219138		0.0283	0.0312	0.999904	0.247516	
4:31782754:A:G	4	31782754	rs79231845	chr4	31781132	0.053990291	11	3.25806796	0.70424346	4.626337545	3.72189E-06	intergenic	LOC102723778;LOC102723828	dist=569458;dist=216247		0.0271	0.0314	0.964283	1	
4:40948164:G:A	4	40948164	rs113189296	chr4	40946147	0.037621359	8	2.919015192	0.615000485	4.746362423	2.07107E-06	intronic	APBB2	.	APBB2	0.0591	0.0852	0.970482	1	Amyloid Beta Precursor Protein Binding Family B Member 2
4:163976364:T:C	4	163976364	rs146154846	chr4	163055212	0.028898058	6	2.023347127	0.417548067	4.845782525	1.26114E-06	intergenic	FSTL5;MIR4454	dist=891178;dist=38362	FSTL5	0.0182	0.0245	0.711952	1	Follistatin-like 5
4:164000229:C:T	4	164000229	rs145034465	chr4	163079077	0.02361165	5	1.814612257	0.387671579	4.680797751	2.85761E-06	intergenic	FSTL5;MIR4454	dist=915043;dist=14497		0.0184	0.0231	0.732815	1	
4:173193727:G:A	4	173193727	rs187567621	chr4	172272576	0.014470874	3	1.644356357	0.349394212	4.706306808	2.52245E-06	intronic	GALNTL6	.	GALNTL6	0.0019	0.0033	0.94795	1	Polypeptide N-Acetylgalactosaminyltransferase Like 6
4:189389927:T:C	4	189389927	rs544973300	chr4	188468773	0.010276699	2	1.359541791	0.297112351	4.575850811	4.74288E-06	ncRNA_intronic	LINC01060	.		0.0035	0.0050	0.946406	1	
4:189389929:T:C	4	189389929	rs560030667	chr4	188468775	0.010296117	2	1.359541054	0.297050321	4.576803849	4.72134E-06	ncRNA_intronic	LINC01060	.		0.0037	0.0047	0.944465	1	
5:3562095:T:A	5	3562095	rs143503046	chr5	3561981	0.00734466	2	0.983175268	0.214354222	4.58668487	4.5034E-06	intergenic	LINC01019;IRX1	dist=25887;dist=34073	IRX1	0.0027	0.0041	0.686588	1	Iroquois Homeobox 1
5:5233724:G:C	5	5233724	rs115149137	chr5	5233611	0.014830097	3	1.706394641	0.358423256	4.760836832	1.92792E-06	intronic	ADAMTS16	.		0.0104	0.0175	0.967403	1	
5:5241203:C:A	5	5241203	rs145155685	chr5	5241090	0.014868932	3	1.68070787	0.35442199	4.742109459	2.11504E-06	intronic	ADAMTS16	.		0.0080	0.0129	0.944326	1	
5:33636035:T:C	5	33636035	rs13164825	chr5	33635930	0.155339806	32	5.112931608	1.104845474	4.627687454	3.69687E-06	intronic	ADAMTS12	.	ADAMTS12	0.1577	0.1968	1	0.705556	ADAMTS12
5:33644123:G:T	5	33644123	rs35388476	chr5	33644018	0.15973301	33	4.946113148	1.073435357	4.60774197	4.07065E-06	intronic	ADAMTS12	.		0.1520	0.2154	0.992101	1	
5:33644614:T:C	5	33644614	rs67267661	chr5	33644509	0.160194175	33	4.958247799	1.078142852	4.598878331	4.24772E-06	intronic	ADAMTS12	.		0.1519	0.2153	1	1	
5:33646456:G:C	5	33646456	rs10057508	chr5	33646351	0.164631068	34	4.973632977	1.076119596	4.621821771	3.80385E-06	intronic	ADAMTS12	.		0.1583	0.2157	0.991904	1	
5:33647634:G:A	5	33647634	rs13176485	chr5	33647529	0.159796116	33	4.948363002	1.073508457	4.609524006	4.03592E-06	intronic	ADAMTS12	.		0.1519	0.2149	0.991564	1	
5:53390040:G:C	5	53390040	rs42872	chr5	54094210	0.306907767	63	6.436279804	1.389496475	4.632095094	3.61984E-06	intronic	ARL15	.	ARL15	0.2490	0.2995	0.980711	0.812933	ARF Like GTPase 15
5:73263379:A:G	5	73263379	rs190652742	chr5	73967554	0.024257281	5	2.247608363	0.454880737	4.941093741	7.76855E-07	intergenic	ARHGEF28;LINC01335	dist=25561;dist=338856	ARHGEF28	0.0104	0.0094	0.999387	1	Rho Guanine Nucleotide Exchange Factor 2
5:112013914:G:A	5	112013914	rs12153142	chr5	112678217	0.980427185	4	-1.381862916	0.302073383	-4.574593434	4.77146E-06	ncRNA_intronic	LOC102467216	.		0.9143	0.9991	0.499216	1	
6:77414670:G:C	6	77414670	rs56232065	chr6	76704953	0.019820388	4	1.925532437	0.417298507	4.614280674	3.94459E-06	intergenic	IMPG1;HTR1B	dist=632275;dist=755895		0.0103	0.0099	0.980026	1	Interphotoreceptor Matrix Proteoglycan 1; 5-Hydroxytryptamine Receptor 1B (serat
6:77420028:C:A	6	77420028	rs78549750	chr6	76710311	0.019699029	4	1.920385778	0.415757928	4.618999777	3.85594E-06	intergenic	IMPG1;HTR1B	dist=637633;dist=750537		0.0160	0.0101	0.978334	1	
6:77429812:T:G	6	77429812	rs77029592	chr6	76720095	0.019640777	4	1.923001054	0.415738055	4.625511258	3.73676E-06	intergenic	IMPG1;HTR1B	dist=647417;dist=740753		0.0083	0.0100	0.980847	1	
6:77460389:C:T	6	77460389	rs191563430	chr6	76750672	0.019490291	4	1.930446587	0.415888081	4.641745998	3.45477E-06	intergenic	IMPG1;HTR1B	dist=677994;dist=710176		0.0071	0.0101	0.988316	1	
6:77472188:A:G	6	77472188	rs55850402	chr6	76762471	0.019490291	4	1.93070443	0.415892439	4.642316741	3.44524E-06	intergenic	IMPG1;HTR1B	dist=689793;dist=698377		0.0070	0.0099	0.988316	1	
7:48396066:G:A	7	48396066	rs189164061	chr7	48356469	0.014519418	3	1.695949216	0.358675926	4.728360877	2.2634E-06	intronic	ABCA13	.	ABCA13	0.0077	0.0119	0.996283	1	ATP Binding Cassette Subfamily A Member 13
7:89801861:C:T	7	89801861	rs7800716	chr7	90172547	0.017533981	4	1.680077446	0.353443095	4.753459521	1.99965E-06	ncRNA_intronic	STEAP2-AS1	.	STEAP2	0.0069	0.0101	0.830555	1	STEAP2 Metalloreductase
7:89853957:C:T	7	89853957	rs149916697	chr7	90224643	0.018815534	4	1.879423001	0.384569904	4.887077696	1.02344E-06	intronic	STEAP2	.		0.0065	0.0095	0.928405	1	
7:89988476:T:C	7	89988476	rs79847565	chr7	90359162	0.018800971	4	1.89404647	0.387759417	4.8845918	1.03643E-06	intronic	GTPBP10	.	GTPBP10	0.0069	0.0100	0.945469	1	GTP Binding Protein 10
7:90013529:G:A	7	90013529	rs187685448	chr7	90384215	0.018776699	4	1.910374378	0.392033885	4.872982793	1.09926E-06	intronic	GTPBP10	.		0.0067	0.0098	0.967919	1	
7:90047115:A:G	7	90047115	rs143425398	chr7	90417801	0.018825242	4	1.913164032	0.392804365	4.870526408	1.11301E-06	intergenic	CLDN12;CDK14	dist=1847;dist=178561	CDK14	0.0066	0.0097	0.970376	1	Cyclin Dependent Kinase 14
7:90253061:G:A	7	90253061	rs150148213	chr7	90623747	0.01892233	4	1.921233543	0.394605718	4.868742276	1.12311E-06	intronic	CDK14	.		0.0066	0.0097	0.973808	1	
8:39944783:C:T	8	39944783	rs145892996	chr8	40087264	0.030776699	6	2.576605742	0.50654249	5.086652731	3.64438E-07	intergenic	IDO2;C8orf4	dist=70873;dist=66204	C8orf4	0.0131	0.0201	0.884119	1	TCIM - Transcriptional And Immune Response Regulator
8:39944917:T:A	8	39944917	rs138697513	chr8	40087398	0.030781554	6	2.577314922	0.506628696	5.087187008	3.63413E-07	intergenic	IDO2;C8orf4	dist=71007;dist=66070		0.0130	0.0201	0.884291	1	
8:40027101:T:C	8	40027101	rs116259399	chr8	40169582	0.04368932	9	2.810807743	0.588520937	4.776053945	1.78768E-06	intergenic	C8orf4;ZMAT4	dist=14274;dist=361010	ZMAT4	0.0402	0.0401	1	1	Zinc Finger Matrin-Type 4
8:40049692:T:G	8	40049692	rs150470417	chr8	40192173	0.044912622	9	2.785140072	0.582245322	4.783447747	1.72314E-06	intergenic	C8orf4;ZMAT4	dist=36865;dist=338419		0.0259	0.0383	0.960377	1	
9:36357606:C:A	9	36357606	rs4879986	chr9	36357609	0.023582524	5	2.18417045	0.448849347	4.866154904	1.1379E-06	intronic	RNF38	.	RNF38	0.0112	0.0117	0.970686	1	Ring Finger Protein 38
9:36360845:T:C	9	36360845	rs79319828	chr9	36360848	0.024014563	5	2.210438405	0.456810098	4.838856266	1.30588E-06	intronic	RNF38	.		0.0116	0.0120	0.9887	1	
9:36381902:C:T	9	36381902	rs2248297	chr9	36381905	0.97573301	5	-2.242692534	0.461738278	-4.857064356	1.19139E-06	intronic	RNF38	.		0.9692	0.9879	0.999795	1	
9:36386865:T:G	9	36386865	rs76774745	chr9	36386868	0.024029126	5	2.210045419	0.456790677	4.838201677	1.31019E-06	intronic	RNF38	.		0.0117	0.0120	0.988086	1	
10:21749029:G:A	10	21749029	rs183818481	chr10	21460100	0.011912621	2	1.22791161	0.263818829	4.654374424	3.24966E-06	intergenic	NEBL-AS1;CASC10	dist=285177;dist=34392	NEBL	0.0059	0.0081	0.674237	1	Nebulette
10:21883439:G:A	10	21883439	rs191906004	chr10	21594510	0.015543689	3	1.645849191	0.333793955	4.93073396	8.19212E-07	intronic	MLLT10	.	MLLT10	0.0058	0.0077	0.843604	1	MLLT10 Histone Lysine Methyltransferase DOT1L Cofactor
10:21970382:G:A	10	21970382	rs188790792	chr10	21681453	0.014849515	3	1.594035309	0.323294895	4.930592272	8.19807E-07	intronic	MLLT10	.		0.0052	0.0080	0.823859	1	
10:22068468:C:A	10	22068468	rs184920540	chr10	21779539	0.011684466	2	1.295186101	0.281543412	4.600306855	4.21869E-06	intronic	DNAJC1	.	DNAJC1	0.0059	0.0079	0.788199	1	DnaJ Heat Shock Protein Family (Hsp40) Member C1
10:22083617:A:G	10	22083617	rs181139900	chr10	21794688	0.011519417	2	1.283845844	0.277566907	4.625356308	3.73955E-06	intronic	DNAJC1	.		0.0064	0.0085	0.775781	1	
10:22216057:G:C	10	22216057	rs45496395	chr10	21927128	0.013985437	3	1.470044503	0.319890854	4.595456497	4.31803E-06	intronic	DNAJC1	.		0.0063	0.0084	0.8731	1	
10:72790290:G:A	10	72790290	rs77364739	chr10	71030533	0.038834951	8	2.690625403	0.557664123	4.824813526	1.40134E-06	intergenic	PCBD1;UNC5B	dist=141747;dist=182002	PCBD1;UNC5B	0.0865	0.0588	1	1	Pterin-4 Alpha-Carbinolamine Dehydratase 1; Unc-5 Netrin Receptor B
10:72806820:G:A	10	72806820	rs75505956	chr10	71047063	0.036461165	8	2.445966686	0.523449162	4.672787474	2.97139E-06	intergenic	PCBD1;UNC5B	dist=158277;dist=165472		0.0366	0.0553	0.953209	1	
10:98558540:C:A	10	98558540	rs61856855	chr10	96798783	0.013485437	3	1.29789842	0.276292266	4.697556103	2.63293E-06	intergenic	PIK3AP1;MIR607	dist=78261;dist=29886	PIK3AP1	0.0113	0.0141	0.63836	1	Phosphoinositide-3-Kinase Adaptor Protein 1;
10:115449191:G:A	10	115449191	rs150977401	chr10	113689432	0.01631068	3	1.860675373	0.358119043	5.195689556	2.03962E-07	intronic	CASP7	.	CASP7	0.0073	0.0111	0.870763	1	Caspase 7
11:21775222:T:A	11	21775222	rs147118669	chr11	21753676	0.012529126	3	1.341788897	0.291618921	4.601172279	4.2012E-06	intergenic	NELL1;ANO5	dist=177993;dist=439500	NELL1;ANO5	0.0050	0.0085	0.79684	1	Neural EGFL Like 1; Anoctamin 5
11:44073753:C:T	11	44073753	rs149030097	chr11	44052203	0.01565534	3	1.691379055	0.360755196	4.688439897	2.75296E-06	intronic	ACCSL	.		0.0134	0.0213	0.931546	1	

11:44085975:C:A	11	44085975	rs76143420	chr11	44064425	0.014893204	3	1.699510358	0.360057984	4.720101852	2.35727E-06	intergenic	ACCSL;ACCS	dist=4448;dist=1754	ACCS	0.0134	0.0215	0.971325	1	1-Aminocyclopropane-1-Carboxylate Synthase Homolog (Inactive)
12:898065:G:T	12	898065	rs2014160	chr12	788899	0.01511165	3	1.72959212	0.339602326	5.092992566	3.52456E-07	intronic	WNK1	.	WNK1	0.0005	0.0007	0.853667	1	WNK Lysine Deficient Protein Kinase 1
12:62144998:G:A	12	62144998	rs76854078	chr12	61751217	0.01723301	4	1.744080472	0.37159489	4.693499608	2.68571E-06	intronic	FAM19A2	.	FAM19A2	0.0089	0.0135	0.904535	1	TAFA2 - TAFA Chemokine Like Family Member 2
12:78530991:C:T	12	78530991	rs61754236	chr12	78137211	0.028985437	6	2.517383543	0.494187455	5.093985122	3.50614E-07	exonic	NAV3	.	NAV3	0.0085	0.0125	0.995116	1	Neuron Navigator 3
12:78545400:G:A	12	78545400	rs140686116	chr12	78151620	0.02904369	6	2.519911214	0.495194249	5.088732794	3.60464E-07	intronic	NAV3	.		0.0063	0.0103	0.997108	1	
13:61672508:T:C	13	61672508	rs147220861	chr13	61098374	0.027004854	6	2.031117161	0.432525467	4.695948131	2.65373E-06	intergenic	LINC00378;MIR3169	dist=402574;dist=101424		0.0080	0.0082	0.826016	1	
13:71228466:C:A	13	71228466	rs9592740	chr13	70654334	0.066820388	14	3.693410575	0.700908722	5.269460143	1.36826E-07	intergenic	ATXN8OS;LINC00348	dist=514581;dist=360807		0.0651	0.0859	0.979474	1	
13:71242428:T:G	13	71242428	rs9599709	chr13	70668296	0.065	13	3.560256036	0.685315257	5.195063146	2.0465E-07	intergenic	ATXN8OS;LINC00348	dist=528543;dist=346845		0.0654	0.0860	0.958512	1	
13:102628784:G:A	13	102628784	rs75197268	chr13	101976434	0.00926699	2	1.077369422	0.228342774	4.718211147	2.37927E-06	intronic	FGF14	.	FGF14	0.0122	0.0178	0.53936	1	Fibrobast Growth Factor 14
13:109580621:C:T	13	109580621	rs145442123	chr13	108928273	0.022786408	5	2.002149238	0.426532153	4.694017139	2.67892E-06	intronic	MYO16	.	MYO16	0.0106	0.0163	0.930524	1	Myosin XVI
14:24001662:C:T	14	24001662	rs41307102	chr14	23532453	0.024456311	5	1.768121176	0.369052815	4.790970568	1.65976E-06	intronic	ZFHX2	.	ZFHX2	0.0265	0.0375	0.636713	1	Zinc Finger Homeobox 2
15:45198007:A:C	15	45198007	rs28631454	chr15	44905809	0.048796117	10	2.632234639	0.565845842	4.651858229	3.28957E-06	intergenic	TRIM69;C15orf43	dist=137980;dist=50893	TRIM69	0.3216	0.1096	0.89064	1	Tripartite Motif Containing 69
15:61531695:C:G	15	61531695	rs143862437	chr15	61239496	0.014752427	3	1.816785354	0.35534236	5.112774488	3.17461E-07	intergenic	RORA;VPS13C	dist=10193;dist=612895	RORA;VPS13C	0.0083	0.0139	0.9521	1	RAR Related Orphan Receptor A; Vacuolar Protein Sorting 13 Homolog C
15:61534825:G:A	15	61534825	rs182462684	chr15	61242626	0.014762136	3	1.816594932	0.35534099	5.112258325	3.1833E-07	intergenic	RORA;VPS13C	dist=13323;dist=609765		0.0084	0.0138	0.951481	1	
15:61555055:G:C	15	61555055	rs143096638	chr15	61262856	0.015490291	3	1.774737275	0.356244746	4.98179214	6.29981E-07	intergenic	RORA;VPS13C	dist=33553;dist=589535		0.0093	0.0154	0.913405	1	
15:61567547:C:T	15	61567547	rs144327698	chr15	61275348	0.01557767	3	1.784212495	0.357897115	4.985266493	6.18765E-07	intergenic	RORA;VPS13C	dist=46045;dist=577043		0.0097	0.0159	0.916644	1	
17:43780479:T:C	17	43780479	rs79985283	chr17	45703113	0.0145	3	1.703938501	0.352883611	4.828613304	1.37487E-06	intronic	MGC57346-CRHR1	.		0.0211	0.0077	0.994938	1	
17:52366146:G:A	17	52366146	rs62072692	chr17	54288785	0.023776699	5	1.836346208	0.394018804	4.660554743	3.15358E-06	intergenic	KIF2B;TOM1L1	dist=463573;dist=611906	TOM1L1	0.0232	0.0371	0.819939	1	Target Of Myb1 Like 1 Membrane Trafficking Protein
18:39394472:G:C	18	39394472	rs113484345	chr18	41814507	0.023024272	5	1.975849294	0.419524828	4.709731495	2.48043E-06	intergenic	KC6;PIK3C3	dist=293911;dist=140691	PIK3C3	0.0229	0.0286	0.890728	1	Phosphatidylinositol 3-Kinase Catalytic Subunit Type 3
19:46480407:T:C	19	46480407	rs4239537	chr19	45977149	0.019417476	4	2.066073669	0.41173362	5.017986311	5.22159E-07	intergenic	NOVA2;CCDC61	dist=3750;dist=18312	NOVA2;CCDC61	0.0835	0.0109	1	1	NOVA Alternative Splicing Regulator 2; Coiled-Coil Domain Containing 61
20:6955818:G:T	20	6955818	rs146812516	chr20	6975171	0.017223301	4	1.82640918	0.380591181	4.798874146	1.5956E-06	intergenic	BMP2;LINC01428	dist=194893;dist=171296	BMP2	0.0161	0.0210	0.862977	1	Bone Morphogenetic Protein 2
20:45720309:A:G	20	45720309	rs146684353	chr20	47091670	0.032533981	7	2.316388446	0.498848234	4.643473279	3.426E-06	intronic	EYA2	.	EYA2	0.0232	0.0301	0.918602	1	Eyes Absent 2
21:35375126:G:A	21	35375126	rs144499025	chr21	34002825	0.007194175	1	0.846200633	0.183711704	4.606133495	4.10225E-06	intergenic	LINC00649;MRPS6	dist=38864;dist=70697	MRPS6	0.0048	0.0076	0.538813	1	Mitochondrial Ribosomal Protein S6
22:19080386:G:A	22	19080386	rs150428353	chr22	19092873	0.024252427	5	2.149293074	0.429334633	5.006102254	5.55433E-07	intronic	DGCR2	.	DGCR2	0.0172	0.0230	0.783413	1	DiGeorge Syndrome Critical Region Gene 2
X:20935027:G:A	X	20935027	rs144178739	chrX	20916909	0.015194175	3	1.517881729	0.327834067	4.630030494	3.65612E-06	intergenic	RPS6KA3;CNKSR2	dist=650277;dist=457509	CNKSR2	0.0231	0.0199	0.814532	1	Connector Enhancer Of Kinase Suppressor Of Ras 2
X:86181688:A:G	X	86181688	rs186620466	chrX	86926685	0.013970874	3	1.634757242	0.331433352	4.932386049	8.12312E-07	intergenic	DACH2;KLHL4	dist=94083;dist=591027	DACH2;KLHL4	0.0162	0.0191	0.873035	1	Dachshund Family Transcription Factor 2; Kelch Like Family Member 4
													57 risk loci							
													one overlaps with 12 month: PCBD1;UNC5B							
													Thus 57+25-1 = 81 total risk loci							
													Gray cells = risk loci overlapping with Indianapolis-1 GWAS; 8/57		14.04%					
