# Supplementary material for: Pharmacogenomics of steroid-induced ocular hypertension: relationship to high-tension glaucomas and new pathophysiologic insight
Source: medRxiv. 2025 Aug 13:2025.08.11.25333245. Preprint. [Version 1] doi: 10.1101/2025.08.11.25333245 (PMC12363710; doi:10.1101/2025.08.11.25333245)
Supplement: Supplement 10 — Table S9. Functional Annotation Prioritized Genes [file media-10.pdf]

Supplementary Table S9. Functional Annotation Prioritized Genes
Functional grouping of prioritized target genes

Functional category	Prioritized target genes associated with SNPs of genome-wide significance	References to GC effects, ocular hypertension, or glaucoma	Prioritized target genes associated with SNPs of suggestive significance
Chromatin remodeling	*AGAP1*<br>*HDAC4*<br>*HDAC9*<br>*SPTY2D1*	ArfGAP protein that functions as a TF, priming chromatin for GR binding, thus influencing gene expression. (BioRxiv)<br>HDAC4 regulates GC signaling (PMID: 40186004). HDAC4 plays a role in neuroprotection and may be a therapeutic target for optic nerve damage in glaucoma (US Patent Publication Number WO/2024/010709)<br>HDAC9, a histone deacetylase, plays a role in the development of endothelial cell dysfunction and changes in vessel permeability in glaucoma (PMID: 37806089)	*AEBP2*<br>*ARID5B*<br>*ATXN1*<br>*CHD4*<br>*GATAD2A*<br>*JMJD7*<br>*MACROD2*<br>*MACROH2A1*<br>*RCBTB1*<br>*SMARCA2*<br>*TBL1XR1*
Transcription	*BEND7*<br>*PPARGC1A*<br>*ZNF737*<br>*ZNF728*	PPARGC1A expression stimulated by GCs; PPARGC1A can potentiate GC-induced transcription (OMIM entry 604517)	*AHR*<br>*ASXL1*<br>*CRTC1*<br>*CTBP2*<br>*DCANP1*<br>*DCT*<br>*DMRT2*<br>*E2F3*<br>*EBF2*<br>*EVX2*<br>*FOXN2*<br>*GTF2B*<br>*HOXD13*<br>*ID2*<br>*JADE1*<br>*JDP2*<br>*KLF5*<br>*KLF6*<br>*KLF12*<br>*KRBOX1*<br>*MAML3*<br>*MEOX2*<br>*MGA*<br>*NR2F1*<br>*NRC32*<br>*PITX2*<br>*PKNOX2*<br>*PLAG1*<br>*PLAGL2*<br>*POU3F2*<br>*PRDM10*<br>*PRDM15*<br>*SALL2*<br>*SHOX2*<br>*SLTM*<br>*SOX17*<br>*SWT1*<br>*TBX18*<br>*TFDP2*<br>*TFEC*<br>*TLE1*<br>*TRPS1*<br>*TTF1*<br>*ZBTB47*<br>*ZBTB49*<br>*ZFAT*<br>*ZKSCAN2*<br>*ZMAT3*<br>*ZNF514*<br>*ZNF536*<br>ZNF641<br>ZNF804B

Alternative RNA splicing	*RBFOX1*		*RBFOX3*
GTPase pathways	*AGAP1*<br>*ARHGEF26*<br>*DIRAS2*<br>*PPM1H*	ArfGAP protein that functions as a TF, priming chromatin for GR binding, thus influencing gene expression. (BioRxiv)<br>ARHGEF26 knockdown ameliorated GC-induced myofibroblast transdifferentiation in TM cells, and prevented the development of GC-induced OH in mice (PMID: 39092889).<br>DIRAS2 is upregulated in lymphocytes following GC treatment. It is a negative regulator of mTOR2 signaling, triggering autophagy (PMID: 21733849).	*ARAP2*<br>*ARHGAP21*<br>*ARHGEF28*<br>*DLC1*<br>*RAC1*<br>*RASSF3*<br>*RND3*<br>*RTKN*<br>*TBC1D12*
Hippo-Yap signaling	*WWC1*	WWC1 activity is affected by the Hippo pathway and GC signaling (PMID: 30627107).	*SCHIP1*
TGFB superfamily signaling	*FST*	FST expression increased in TM of glaucoma patients, and expression is upregulated by TGFB2 (PMID: 23010638).	*FSTL5*<br>*SOSTDC1*<br>*VWC2*
cAMP signaling	*PRKD1*		
Energy metabolism	*PPARGC1A*	PPARGC1A expression stimulated by GCs; PPARGC1A can potentiate GC-induced transcription (OMIM entry 604517)	*ACADSB*<br>*ACSF2*<br>*ACSL3*<br>*ADIPOR2*<br>*ALDH5A1*<br>*ALDH8A1*<br>*ALK*<br>*AMY2A*<br>*AMY2B*<br>*ARID5B*<br>*ASNS*<br>*CBR4*<br>*CHCHD7*<br>*CMC1*<br>*CRTC1*<br>*CTBP2*<br>*ETF3*<br>*ETNK1*<br>*LEPR*<br>*LPROT*<br>*LIPC*<br>*MOGAT1*<br>*MSRB2*<br>*MTX2*<br>*NT5C2*<br>*PC*<br>*PHYHIP*<br>*TMEM26*<br>*TMEM86A*
Immunity and inflammatory response	*CCR6*		ENDOD1<br>HCK<br>TBK1<br>TLR2<br>TNFRSF13B
Ubiquitination	*ASB3*<br>*C4orf19*		*ASB8*<br>*BTRC*<br>*FBXO7*<br>*FBXO11*<br>*NEDD4L*<br>*RNF2*<br>*RNF111*<br>*RNF144A*<br>*SH3RF1*<br>*SOCS7*<br>*TBL1XR1*

De-ubiquitination	*USP25*		*OTUD3*<br>*USP18*
GPI Anchor Cleavage	*GPLD1*		
Complement Inhibition & EMT	*CSMD1*		*CSMD3*
Cell-ECM Adhesion	*SGCG*		*FERMT1*
ECM Assembly	*COL11A1*<br>*KAZALD1*		

Supplementary Table S9. Functional Annotation Prioritized Genes

Pathway Enrichment Analysis

NOTES

Events identified by Reactome; enrichment scores determined using DAVID Bioinformatics functional annotation tool

Ordered by GWAS prioritized target gene enrichment score

DEGs: genes differentially regulated by glucocorticoids (GCs)

Prioritized target genes: see Table S3

Paired eye study DEGs:see Table S4

Reactome Events	DAVID Enrichment Scores		
	Prioritized target genes	Paired eye study responder DEGs	Paired eye study non-responder DEGs
Signal transduction: signaling by NOTCH	1.86	Not scored	Not scored
Disease: diseases of signal transduction by growth factor receptors and second messengers [cancer]	1.86	0.51	Not scored
Muscle contraction	1.77	2.52	Not scored
Muscle contraction: cardiac conduction	1.77	2.52	Not scored
Muscle contraction: cardiac conduction: Phase 0 – rapid depolarisation	Not scored	0.99	Not scored
Muscle contraction: cardiac conduction: Phase 1 - inactivation of fast Na+ channels	1.77	Not scored	Not scored
Muscle contraction: cardiac conduction: Phase 2 - plateau phase	Not scored	Not scored	Not scored
Muscle contraction: cardiac conduction: Phase 3 - rapid repolorisation	Not scored	Not scored	Not scored
Metabolism: metabolism of carbohydrates	1.3	0.2	Not scored
Metabolism: metabolism of carbohydrates: glycosaminoglycan metabolism	1.3	0.2	Not scored
Developmental biology: adipogenesis	1.12	Not scored	Not scored
Transport of small molecules	0.92	Not scored	Not scored
Transport of small molecules: ion channel transport	0.92	Not scored	Not scored
Transport of small molecules: stimuli-sensing channels	0.92	Not scored	Not scored
Disease: infectious disease: HIV infection	0.89	Not scored	Not scored
Disease: diseases of metabolism	0.8	Not scored	Not scored
Disease: diseases of glycosylation	0.8	Not scored	Not scored
Metabolism of proteins: post-translational protein modification: O-linked glycosylation	0.8	Not scored	Not scored
Signal transduction: signaling by Rho GTPases, Miro GTPases and ROBTB3	0.8	0.14	0.69
Developmental biology	0.75	Not scored	0.74
Developmental biology: nervous system development	0.75	Not scored	0.74
Developmental biology: axon guidance	0.75	Not scored	Not scored
Vesicle-mediated transport	0.71	Not scored	Not scored
Vesicle-mediated transport: membrane trafficking	0.71	Not scored	Not scored
Vesicle-mediated transport: clathrin-mediated endocytosis	0.71	Not scored	Not scored
Immune system: cytokine signaling in immune system: signaling by interleukins	0.67	Not scored	Not scored
Hemostasis: platelet activation, signaling and aggregation	0.67	0.73	0.67
Signal transduction: signaling by VEGF	0.65	Not scored	Not scored
Cellular response to stimuli: cellular senescence	0.55	Not scored	Not scored
Circadian clock	0.49	Not scored	Not scored
Organelle biogenesis and maintenance	0.49	Not scored	Not scored
Organelle biogenesis and maintenance: mitochondrial biogenesis	0.49	Not scored	Not scored
Disease	0.45	0.19	Not scored
Disease: infectious disease	0.45	0.19	Not scored
Disease: viral infection	0.45	0.19	Not scored
Signal transduction: intracellular signaling by second messengers	0.45	0.15	Not scored
Signal transduction: signaling by GPCR	0.44	3.98	2.2
Signal transduction: signaling by TGFB family members	0.35	Not scored	Not scored
Cell-cell communication	0.31	1.7	0.45
Immune system: cytokine signaling in immune system	0.27	Not scored	Not scored
Gene expression (transcription): epigenetic regulation of gene expression	0.25	0	Not scored
Chromatin organization	0.22	Not scored	Not scored
Signal transduction: signaling by nuclear receptors	0.19	Not scored	Not scored
Signal transduction: MAPK family signaling cascades	0.18	0.48	Not scored
Immune system: adaptive immune system	0.16	Not scored	Not scored
Immune system: adaptive immune system: antigen processing & presentation	0.16	Not scored	Not scored
Metabolism of proteins: post-translational protein modification: neddylation	0.16	Not scored	Not scored

Gene expression (transcription)	0.15	0	0.4
Gene expression (transcription): RNA polymerase II transcription	0.15	0	0.4
Cell cycle	0.08	Not scored	Not scored
Metabolism of RNA	0.03	Not scored	Not scored
Metabolism	Not scored	Not scored	Not scored
Signal transduction: signaling by Wnt	Not scored	3.45	Not scored
Signal transduction	Not scored	3.45	Not scored
Extracellular matrix organization	Not scored	1.99	Not scored
Immune system	Not scored	1.26	Not scored
Immune system: innate immune system	Not scored	1.26	Not scored
Immune system: innate immune system: neutrophil degranulation	Not scored	1.26	Not scored
